# Vitamin D-deficient mice have more invasive urinary tract infection

**DOI:** 10.1371/journal.pone.0180810

**Published:** 2017-07-27

**Authors:** Olof Hertting, Petra Lüthje, Devin Sullivan, Pontus Aspenström, Annelie Brauner

**Affiliations:** 1 Department of Microbiology, Tumor and Cell Biology, Division of Clinical Mircrobiology, Karolinska University Hospital and Karolinska Institutet, Stockholm, Sweden; 2 Pediatric Infectious Diseases Unit, Astrid Lindgren Children´s Hospital, Karolinska University Hospital, Stockholm, Sweden; 3 KTH Royal Institute of Technology, Science for Life Laboratory, School of Biotechnology, Stockholm, Sweden; 4 Department of Microbiology, Tumor and Cell Biology, Karolinska Institutet, Stockholm, Sweden; Northwestern University, UNITED STATES

## Abstract

Vitamin D deficiency is a common health problem with consequences not limited to bone and calcium hemostasis. Low levels have also been linked to tuberculosis and other respiratory infections as well as autoimmune diseases. We have previously shown that supplementation with vitamin D can induce the antimicrobial peptide cathelicidin during *ex vivo* infection of human urinary bladder. In rodents, however, cathelicidin expression is not linked to vitamin D and therefore this vitamin D-related effect fighting bacterial invasion is not relevant. To determine if vitamin D had further protective mechanisms during urinary tract infections, we therefore used a mouse model. In vitamin D-deficient mice, we detected more intracellular bacterial communities in the urinary bladder, higher degree of bacterial spread to the upper urinary tract and a skewed cytokine response. Furthermore, we show that the vitamin D receptor was upregulated in the urinary bladder and translocated into the cell nucleus after *E*. *coli* infection. This study supports a more general role for vitamin D as a local immune response mediator in the urinary tract.

## Introduction

Urinary tract infection (UTI) is one of the most common bacterial infections in humans, mainly affecting women and children [1]. *E*. *coli* accounts for about 80% of all UTIs [2] and >50% of women have at least one UTI during their lifetime, with more than 25% risk of experiencing a recurrent UTI within six months after the initial infection [3]. These recurrences are often caused by the same bacterial strain, suggesting incomplete resolution of the infection. For UTI to occur, bacteria enter the urinary tract, proceed against the flow of urine, adhere and invade superficial facet cells of the uroepithelium. Intracellularly, they rapidly multiply and may form intracellular bacterial communities (IBC) [4, 5], tightly packed bacteria surrounded by a biofilm-like substance, which enable them to hide from eradication by the host innate immune defense. Bacteria released from such IBCs can re-activate the infection, spread to other parts of the urinary tract or can invade nearby cells where they may enter a dormant stage [6]. The host on the other hand has developed different mechanisms to respond to the bacterial invasion, where the uroepithelium constitutes the first line of defense. Upon bacterial attachment, these cells respond rapidly by secreting antimicrobial peptides. We have demonstrated that the antimicrobial peptide cathelicidin protects the urinary tract against severe UTI in mice and that the same mechanisms are of importance during *E*. *coli* UTI in humans [7]. In addition, cytokines and chemokines play an important role in the pathogenesis [8]. A tightly regulated activation is needed to attract immune cells to the site of infection and avoid excessive and harmful inflammatory activity [9].

The increasing understanding of vitamin D involvement in the immune system has established a strong link between vitamin D and innate immunity [10]. We previously showed that vitamin D could induce cathelicidin in human urinary bladder cells and tissue [11]. Further, vitamin D receptor (VDR) knock-out mice had more severe *Salmonella* infection than their wild-type counterparts [12] and dietary-induced vitamin D-deficient mice had more severe inflammation and significantly higher bacterial load in a model of colitis [13]. We therefore hypothesized that vitamin D could have additional effects also in the urinary tract and that such influence would impact bacterial clearance.

## Material and methods

### Bacteria

Uropathogenic *E*. *coli* strain CFT073 was isolated from a patient with acute pyelonephritis and expresses type 1-, P- and S fimbriae as well as alpha-hemolysin. Bacteria were grown overnight on blood agar at 37°C, and then in LB broth for another 4 hours to reach logarithmic phase of growth. Bacteria were harvested by centrifugation and then washed twice with PBS. The bacterial concentration was measured by spectrophotometry and confirmed by viable count. This strain was used for both *in vivo* and *in vitro* experiments.

### Mouse model of urinary tract infection

Mouse experiments were approved by the Northern Stockholm Animal Ethics Committee and experiments were carried out according to FELASA guidelines and in compliance with the Committee’s requirements. C57BL/6 mice (Harlan Laboratories) were used in a model of ascending UTI described previously [14]. Briefly, mice were housed in rooms with 12:12 hour light-dark cycle. Food and water were provided *ad libitum*. They were anesthetized with isoflurane and infected transurethrally with 50 μL suspension of the uropathogenic *E*. *coli* strain CFT073 (10^9^ CFU/ml). Sterile PBS was used for non-infected control mice. After 14, 24 and 48 hours (n = 4 for each time-point), they were sacrificed and the bladders were cut open, rinsed and fixed in 4% paraformaldehyde (PFA) in PBS. In additional experiments, 3 weeks old C57BL/6 mice were given vitamin D deficient diet TD.89123 for 7 weeks, starting directly after weaning and were kept behind UV protection film (Clear 1 UV, Data Solution, Sweden). The TD.89123 diet is free of vitamin D and control mice were fed a diet (TD.110133) supplemented with 1.5 IU/g of cholecalciferol (Harlan Laboratories). At 10 weeks of age, experimental UTI was performed.

At the indicated time points, the mice were sacrificed. To determine the total bacterial load, we homogenized the bladders (n = 7 vitamin D-deficient, n = 8 controls) and kidneys (n = 18 vitamin D-deficient, n = 16 controls) in 0.5 mL PBS and serial dilutions of the homogenate were plated on blood agar for viable count. Homogenates were then centrifuged at 10 000 x g and stored at -20°C, pending ELISA analyses. For microscopy, bladders (n = 4 deficient, n = 4 controls) were fixed in 4% PFA overnight at 4°C and processed for sectioning.

### Mouse multiplex cytokine assay

Bladder and kidney homogenates were assayed in the Bio-Plex system (Bio-Rad). This array has been useful in determining cytokine response in experimental UTI in mice [15, 16]. Nine cytokines were analysed, IL-1β, IL-17A, IL-6, MIP-2, MIP-1β, KC, MCP-1, G-CSF and RANTES. All samples were run on the same plate to avoid inter-assay variation. The intra-assay variation was less than 5% for all cytokines measured according to the manufacturer.

### Serum vitamin D measurement

At the end of the experiment just before sacrificing the animals, blood was sampled for measurement of serum levels of 25OHD_3_ (25D_3_). After clotting for 60 minutes at room temperature, tubes were spun at 800 x g for 10 minutes. Serum was protected from light and stored at -20°C pending 25D_3_ measurement. 25D_3_ analyses were performed using LIASION 25-OH Vitamin D TOTAL kit, an antibody-based chemiluminescence assay (DiaSorin Inc.).

### Human uroepithelial cells

Normal telomerase immortalized bladder cells (TERT-NHUC) [17] were kindly provided by Professor Margareth Knowles, Leeds University, UK. The original samples of normal urothelium were taken with informed consent of the patient or their guardian and in agreement of the Local Research Ethics Committee [18].

TERT-NHUC cells were propagated in Keratinocyte Serum Free Medium (Invitrogen) at 37°C. 5637 and T24 bladder epithelial cell lines (ATCC) were grown in RPMI and McCoy's 5a Medium (Invitrogen) respectively, both supplemented with 10% fetal bovine serum at 37°C. Cells were treated with 1,25D_3_ (Calbiochem) at indicated concentrations and infected with *E*. *coli* CFT073 when appropriate. To study the influence of vitamin D, TERT-NHUC treated with 10^−8^ M 1,25D_3_ (Calbiochem) for 24 hours when near confluent.

### Silencing of VDR gene

5637 bladder cells were seeded 2 x 10^5^ cells per well in a 24-well plate. After 24 hours, cells were transfected with 1 μL Dharmafect 1 (Thermo Fisher) per well and silenced with 100 nM VDR siRNA (Thermo Fisher) for 16 hours. This silencing step was repeated once after 24 hours [19]. Scrambled siRNA (Thermo Fisher) and mock treated cells were used as controls.

### Real-time PCR

For the measurement of gene induction in human bladder cells, real-time PCR was performed. RNA was extracted from cells after treatment and infection at different time points using the RNeasy Mini Kit (Qiagen). cDNA was then synthesized from 1 μg of RNA using a MuLV transcriptase (Finnzymes) according to the manufacturers description. PCR was performed using Applied biosystems TaqMan Gene Expression Assays on a Rotor-Gene cycler. For calculation of gene induction, the delta delta Ct method was used [20].

### Vitamin D responsive element firefly luciferase assay

To determine transcriptional activity of the vitamin D receptor in urinary bladder cells upon infection, 5637 bladder epithelial cells were grown in 96-well plates and transfected for 16 hours with Cignal Vitamin D Reporter Kit (SABioscience) according to the manufacturer’s instructions. Briefly, the change in the activity of the VDR is determined by comparing the normalized luciferase activities of the reporter in infected versus uninfected transfectants. The identically treated negative control transfectants serve as a specificity control. The positive control serves as a control for transfection efficiency, by monitoring firefly luciferase and Renilla expression. After transfection, fresh normal medium was added for 24 hours after which the cells were infected with *E*. *coli* CFT073 for 90 minutes. Cells were then lysed and luciferase activity was determined using Dual-Luciferase Reporter (DLR) Assay System (Promega) in a Wallac 1420 VICTOR microplate luminometer (Perkin Elmer).

### Tissue and cell imaging

After fixation in 4% PFA, organs were stored in 70% ethanol and then rinsed and embedded in paraffin. Next, 4 μm sections were cut and mounted on glass slides. After dewaxing in xylene, graded rehydration in 100%, 95% and 70% ethanol was performed followed by antigen retrieval by boiling in citrate buffer for 20 minutes. Blocking with 10% rabbit or donkey serum as appropriate in 0.1% Triton and with Image enhancer (Invitrogen) was followed by incubation overnight with primary antibodies for VDR (Thermo Fisher), uroplakin III and *E*. *coli* at 4°C and Alexa Fluor secondary antibodies (Invitrogen).

For immunocytochemistry, TERT-NHUC were seeded on coverslips (VWR) and grown until nearly confluent. After stimulation with vitamin D and/or *E*. *coli*, cells were washed in PBS, then fixed in 4% PFA and processed as described for tissue.

Confocal microscopy was performed using a Leica SP5 system. In order to visualize the actin filament system after infection with *E*. *coli*, human uroepithelial cells were stained with tetramethylrhodamine B isothiocyanate (TRITC)-labelled phalloidin (Sigma).

### NF-κB nuclear translocation

For NF-κB nuclear translocation, TERT-NHUCs were grown on 10-cm dishes and treated with 10^−8^ M 1,25D_3_ (Calbiochem) or vector for 24 hours. Thereafter, cells were infected with 0.4x10^8^ CFU *E*. *coli* CFT073 per dish for 2 hours; non-infected cells served as controls. Cells were washed with PBS/EDTA and extraction of the nuclear and cytoplasmic fraction was performed with the NE-PER Nuclear and Cytoplasmic Extraction Reagents following the manufacturer’s protocol (Thermo Scientific). Starting material was a cell pellet of 20 μl per sample. The protein concentration in the final extracts was determined using the BCA assay (Pierce, Thermo Scientific) and samples were stored at -80°C until analysis. For Western blots, equal amounts of protein in 15 μl buffer was mixed with an equal amount of 2x Laemmli sample buffer supplemented with β-mercaptoethanol as recommended by the supplier (Biorad). Samples were denaturated at 95μC for 5 min. Proteins were separated on 10% Tris-HCl polyacrylamide gels (Biorad) and then transferred to PVDF memebranes (Invitrogen). Membranes were blocked in 5% milk in Tris-buffered saline with 0.1% Tween 20 (TBS-T) and incubated with rabbit anti-NFκB (p65; 1:200; sc-372, Santa Cruz Biotehcnologies) overnight at 4°C or for 1 hour at room temperature, followed by secondary antibody incubation for 1 hour at room temperature. Signals were detected with the SuperSignal West Pico Chemiluminescent kit (Thermo Scientific). Band intensity was quantified using ImageJ.

### Image analysis

For the actin filament image processing, CellProfiler (v2.1.1) software was used [21]. Global thresholding was performed using RidlerCalvard segmentation to identify nuclei, which were used as seeds for adaptive propagation segmentation using Otsu’s method minimized on the weighted variance to segment the cell body based on the actin channel. The eccentricity of each cell was calculated using the MeasureObjectSizeShape feature calculation module within CellProfiler. Comparison of the AreaShape_Eccentricity feature of cells in each condition was performed using analysis of variance (ANOVA) in MATLAB R2016a. Both the CellProfiler and MATLAB scripts are freely available with this work and under the GNU public license (S1 File).

### Statistics

Results are shown as mean and standard deviation of the mean. Between-group comparisons were made using unpaired *t* test. For comparisons between multiple groups ANOVA was used. Data were analysed using Prism GraphPad. A *p*-level of <0.05 was considered significant.

## Results

### Dietary-induced vitamin D deficiency in mice caused more pronounced infection

**Fig 1:**
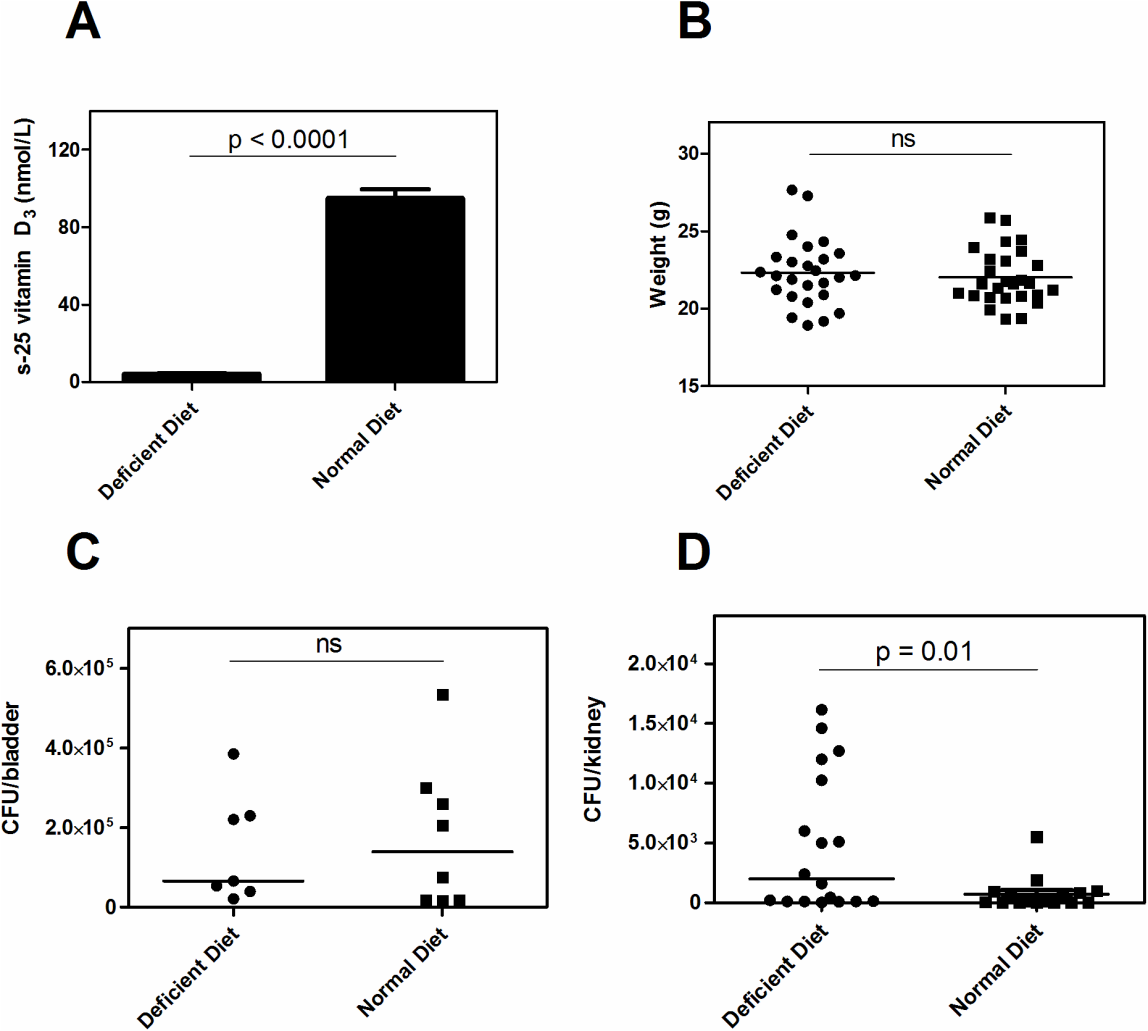
Higher bacterial burden in dietary-induced vitamin D deficiency. C57BL/6 mice were fed a vitamin D-deficient diet or control diet (n = 26 for each group) for 7 weeks directly after weaning. After 7 weeks mean serum levels were 4 nM in the deficient diet group vs. 96.7 nM in control mice (A). No difference in weight between the two groups was observed (n = 26 respectively). (B). More bacteria spread to the upper urinary tract in the vitamin D-deficient group as measured by bacterial count in the kidneys, (n = 9 in each group) (C) whereas no difference in bacterial load in the urinary bladder of the vitamin D-deficient and supplemented mice was observed, (n = 7 vitamin D deficient mice; n = 8 control mice) (D).

**Fig 2:**
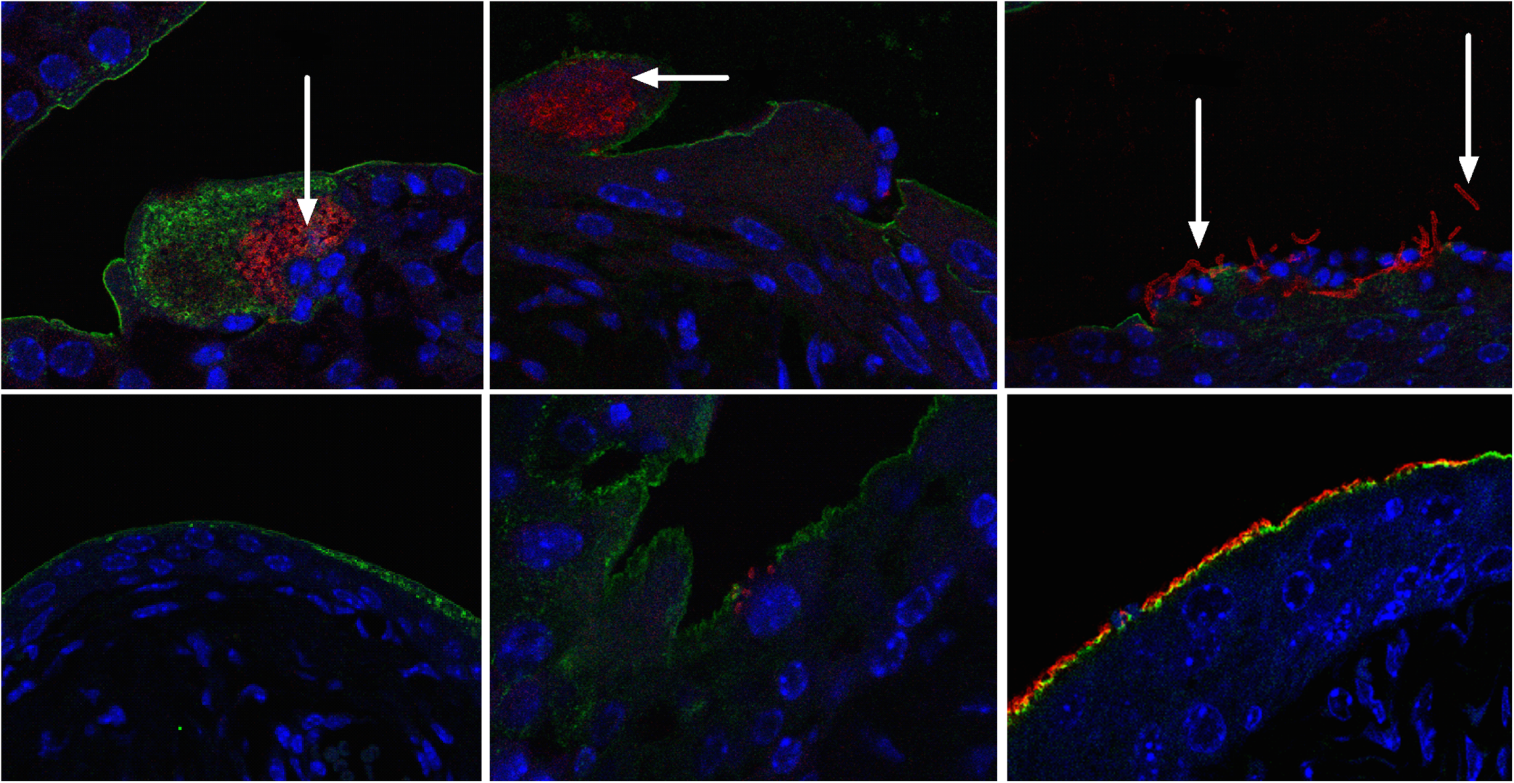
Altered bacterial invasion in vitamin D-deficient bladders. Uropathogenic *E*. *coli* strain CFT073 (red) form aggregates in vitamin D-deficient bladders both in the form of intracellular bacterial communities, IBC (upper panel, left and center, arrow) and large elongated bacterial complexes (upper panel right, arrow) not seen in the vitamin D-sufficient control mice (lower panel). Although also heavily infected, bacteria tended to respect the epithelial border lined with uroplakin (green) (n = 4 in each group). Three representative pictures from four vitamin D deficient and four vitamin D sufficient, control mice (Nuclei are stained with DAPI (blue). Objective used x 63.

To elucidate if vitamin D influences the urothelial immunity besides inducing cathelicidin, we used a mouse model of ascending UTI. Since cathelicidin expression is not affected by vitamin D in rodents, this is a suitable model. Mice were fed a diet either lacking 25D_3_ or supplemented with 1.5 IU/g of 25D_3._ After 7 weeks on a vitamin D-deficient diet, serum 25D_3_ levels from randomly selected mice were near the lower detection limit (median 4 nM in the deficient diet group vs. 96.7 nM in control mice, *p*<0.0001, Fig 1A), confirming an efficient model of severe vitamin D deficiency. There was no difference in weight between the two groups (median 22.12 vs. 21.62, *p* = 0.89) (Fig 1B). Mice were infected with *E*. *coli* and after 24 hours bladders and kidneys were analysed for the presence of bacteria (Fig 1C and 1D). Kidneys from vitamin D-deficient mice contained significantly more bacteria than in the vitamin D-sufficient control mice, indicating a more invasive spread of the infection (*p*<0.01) (Fig 1C). No difference in bacterial counts was observed in the bladder (Fig 1D). Interestingly however, bacteria formed multiple IBCs and large elongated bacteria released from their intracellular compartment were found in the vitamin D-deficient mice (Fig 2, upper panel). In contrast, in vitamin D-supplemented mice, the epithelium was intact, bacteria attached on the luminal side and only few IBCs were seen in the superficial cells and no elongated bacteria were detected (Fig 2, lower panel).

### Vitamin D protects human uroepithelial cells against cytoskeletal reorganization caused by *E*. *coli*

**Fig 3:**
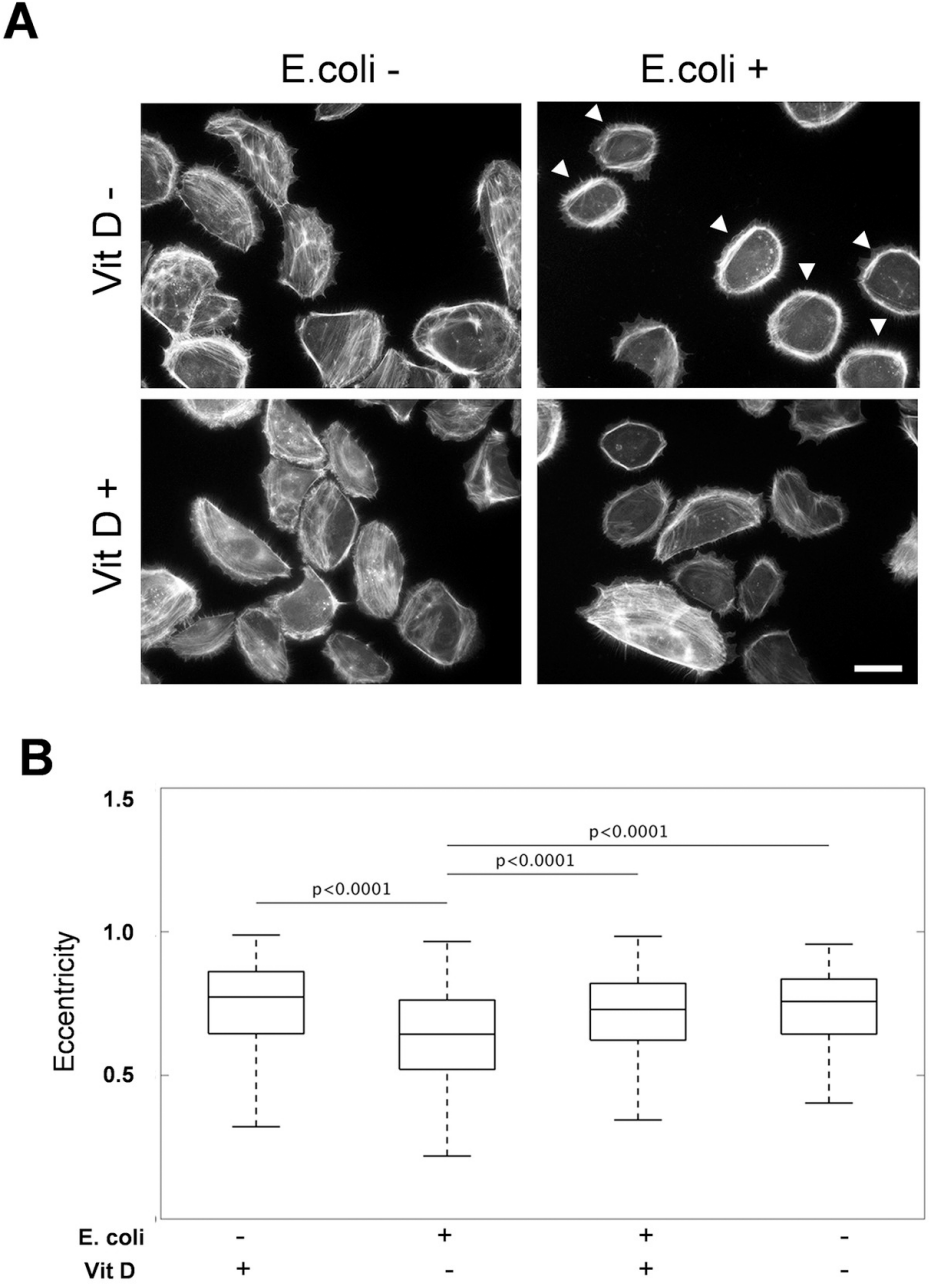
Vitamin D has a protective effect on the cytoskeletal reorganization caused by *E*. *coli* infection of human uroepithelial cells. The actin filament system of human uroepithelial cells was visualized with TRITC-labelled phalloidin. *E*. *coli* strain CFT073 infection caused the formation of cortical bundles of actin filaments (A, arrowheads). Image analysis shows that treatment with *E*. *coli* significantly altered cell morphology from control as measured by eccentricity (B). Hence, treatment of infected cells with vitamin D significantly recovered the wild-type phenotype while treatment with vitamin D in the absence of infection also showed wild-type cellular morphology and significant differences from infected phenotype.

In order to study the effect of *E*. *coli* infection on the cellular level, the actin filament system of human uroepithelial cells, not supplemented with vitamin D, was visualized after infection with *E*. *coli*. Non-infected cells had an uneven ellipsoid morphology. The actin filaments were mainly organized in short stress fibers and bundles. *E*. *coli* infection resulted in a dramatic reorganization of actin filaments into cortical bundles that entirely circumvented the cell periphery (Fig 3A, arrowheads). In addition, the cell shape became less elongated. Image analysis demonstrated a significant change (*p*<0.0001) in the eccentricity of the *E*. *coli-*infected cells (Fig 3B). In contrast, vitamin D treatment prior to *E*. *coli* infection abolished the actin reorganization (Fig 3A) and change in cell shape did not occur (*p* = 0.19) (Fig 3B). These findings suggest that vitamin D treatment can protect uroepithelial cells from cytoskeletal reorganization caused by bacterial infection.

### Vitamin D-deficient mice have a dysregulated bladder cytokine response

Further, we analyzed the cytokine response and measured the major neutrophil chemoattractants, KC, MIP-1β, MIP-2 and G-CSF, the monocyte and lymphocyte chemoattractants MCP-1 and RANTES, and the pro-inflammatory cytokines IL-1β, IL-17A and IL-6. In the urinary bladder, chemokines correlated well with bacterial load of the supplemented control group, KC (R = 0.79; *p*<0.05), MCP-1 (R = 0.76; *p*<0.05) and MIP-2 (R = 0.74; *p*<0.05). In the vitamin D-deficient group, however, this correlation was not seen. While no significant differences of cytokines/chemokines were observed between the control group and the vitamin D-deficient group, 7 out of the 9 tested cytokines appeared lower in the vitamin D-deficient mice. In kidneys, MIP-2 and G-CSF correlated with the bacterial load in both vitamin D-supplemented (MIP-2, R = 0.74, *p* = 0.001; G-CSF, R = 0.51, *p*<0.05) and deficient mice (MIP-2, R = 0.78, *p* = 0.0001; G-CSF, R = 0.61, *p*<0.01). No absolute differences of cytokines/chemokines were observed between the vitamin D-deficient and supplemented groups. Since NF-κB is a key regulator of inflammatory processes, we investigated its activation upon infection with *E*. *coli in vitro*. In line with the results *in vivo*, we could not detect any difference in nuclear translocation of NF-κB between vitamin D-treated and deficient cells (data not shown).

### *E*. *coli* increase vitamin D receptor levels and induce vitamin D receptor translocation

**Fig 4:**
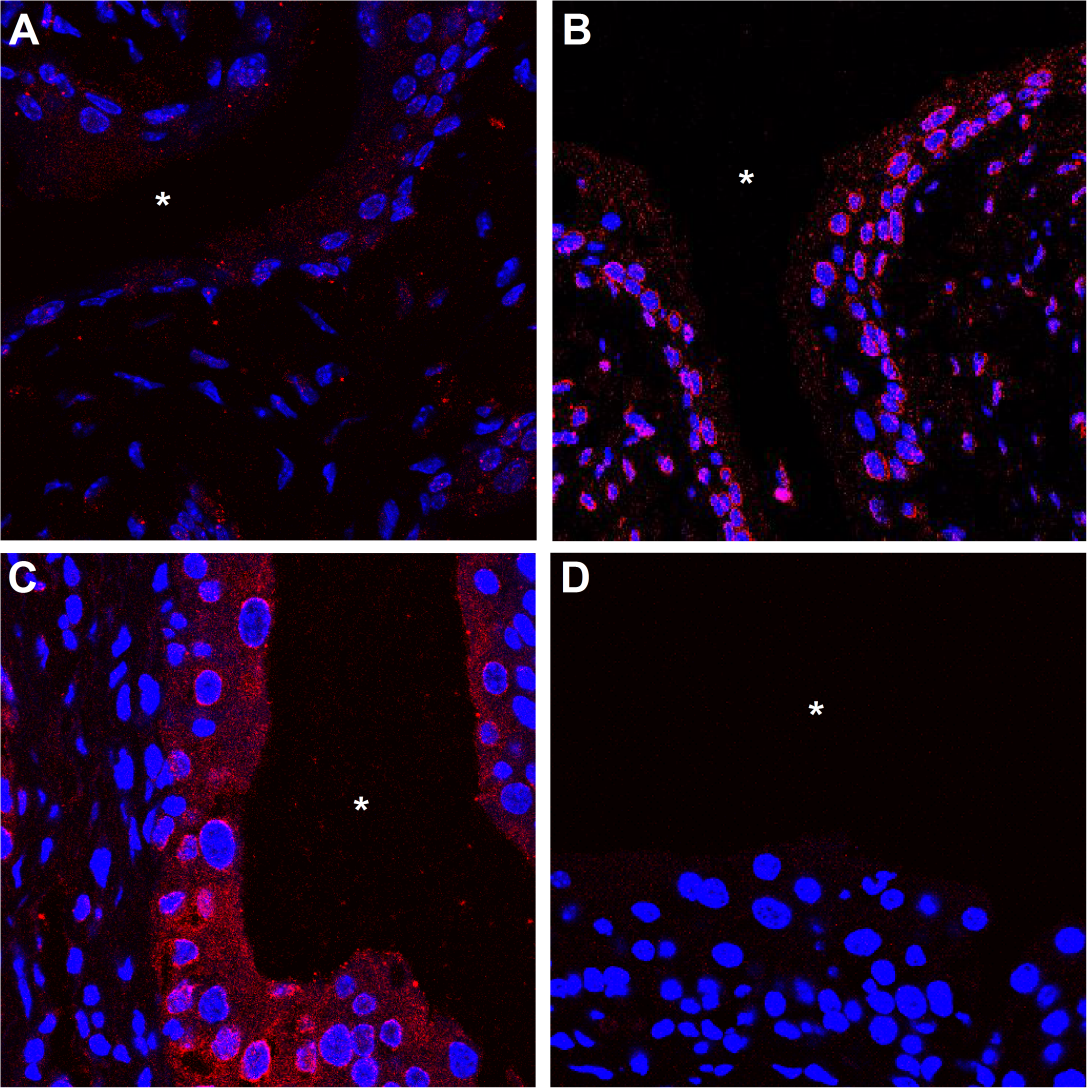
Vitamin D receptor (VDR) increases over time following *E*. *coli* infection of the uroeptithelium. C57BL/6 mice were infected with uropathogenic *E*. *coli* CFT073 for 14 (A), 24 (B) and 48 hours (C). Staining for VDR protein shows a time-dependent upregulation with initial patchy staining and progressive accumulation. Section without primary antibody is shown as control (D). Bladder lumen is marked with *. Four mice for each time-point.

To explore the underlying mechanisms of our findings, we investigated the kinetics of vitamin D action in the urinary bladder during infection and the induction and cellular localization of VDR in infected mice. Infection with *E*. *coli* CFT073 led to an increase of VDR in the urinary bladder in a time-dependent manner, as visualized by fluorescent immunohistochemistry. Only the uroepithelium was affected, suggesting a local VDR response to *E*. *coli* infection. By 14 hours, a slight increase in VDR in the cytoplasm was observed. At later time points, 24 and 48 hours, VDR was seen to assemble at the nuclear membrane and a distinct increase in cytoplasmic VDR was noted (Fig 4). These findings prompted us to investigate if similar mechanisms were present in human bladder cells.

**Fig 5:**
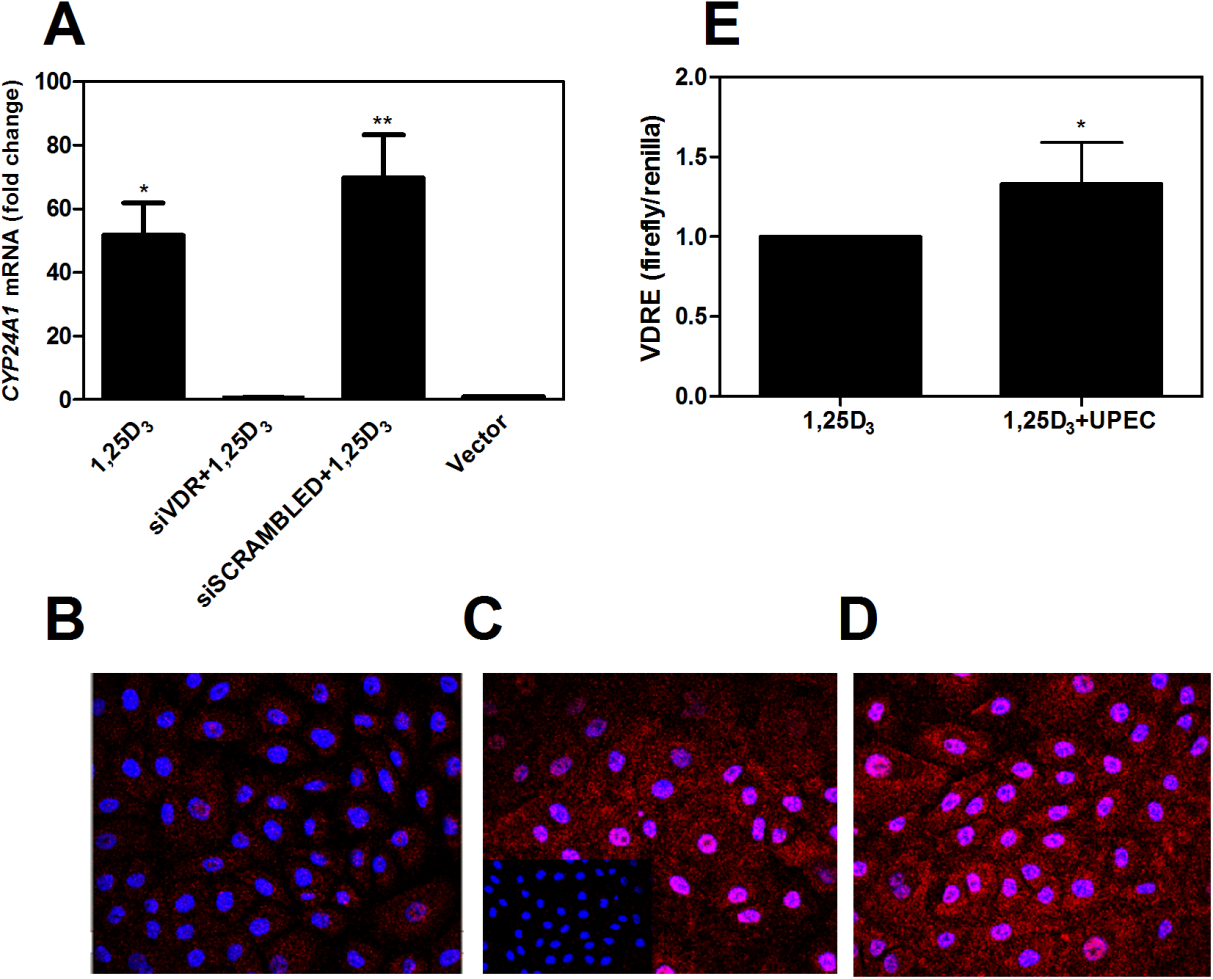
Human bladder cells show nuclear translocation and upregulation of VDR upon uropathogenic *E*. *coli* infection. To confirm that VDR conveys vitamin D signaling in the human urinary bladder, VDR silencing with siRNA was performed in bladder epithelial cells, TERT-NHUC. The expression of the vitamin D downstream target *CYP24A1* was almost completely abolished (A). Two independent experiments. Bladder cells were then infected with *E*. *coli* CFT073 for 45 (B), 90 (C) and 120 minutes (D). VDR increased and translocated into the nucleus at 90 minutes. Infected cells without primary antibody served as controls (inserted in B). Further, bladder cells were transfected with VDRE firefly constructs. In the presence of physiological levels of vitamin D, uropathogenic *E*. *coli* increased the transcriptional activity of VDR compared to uninfected cells. Pooled values from two independent experiments (n = 7 with 3 and 4 analyses in the respective experiments).

By silencing the VDR in human bladder cells, the prototypic vitamin D downstream target CYP24A1 was significantly decreased, demonstrating that the VDR is functionally responsible for downstream vitamin D effects (Fig 5A). Next, we investigated the effect of *E*. *coli* infection on VDR in human bladder cells. As observed in mouse bladder tissue, the overall VDR content in the cells increased in a time-dependent manner and VDR translocated into the nucleus (Fig 5B–5D). We then studied whether infection with uropathogenic *E*. *coli* could directly affect transcriptional activity of the VDR by employing a luciferase reporter assay in human bladder cells. Without any vitamin D in the culture medium, no increased activity was observed (data not shown). However, in the presence of physiological levels of vitamin D, VDRE activity increased significantly in the *E*. *coli*-infected cells (*p*<0.05, Fig 5E). This demonstrates that, in the presence of normal vitamin D levels, uropathogenic *E*. *coli* infection *per se* can induce increased receptor production and thus more vitamin D signaling in response to infection of uroepithelial cells.

## Discussion

In this study we sought to explore the role of vitamin D during UTI independently of the cathelicidin-mediated effect previously demonstrated [11]. Promoters of the cathelicidin genes in mice lack a VDRE, which makes this a suitable animal model. In vitamin D-deficient mice, more IBCs were observed compared to control mice. The exact mechanism remains unknown, but several cellular proteins and innate immune receptors are required for IBC formation [22], and may be influenced by vitamin D. In line with our hypothesis, we here show that vitamin D can abolish actin reorganization in human bladder cells, an important feature of uptake, intracellular growth and resurgence of uropathogenic *E*. *coli* [23]. Once bacteria hide within IBCs, they are no longer recognized by the immune system, with less activated immune response as a result [24]. In contrast, the same numbers of bacteria but fewer IBCs were observed in supplemented mice, suggesting a higher proportion of bacteria exposed to immune recognition and potential antibiotic treatment. Another means for the cell to reduce the bacterial burden is expulsion. We cannot exclude that vitamin D has an impact on expulsion of bacteria from the bladder cells, thereby decreasing the intracellular bacteria and development of IBCs.

After the initial IBC formation and maturation, the cells release bacteria into the lumen, where they can invade other uroepithelial cells or spread to other parts of the urinary tract [25]. In line with the higher number of IBCs, the vitamin D-deficient mice in the present study had significantly more elongated bacteria and more bacteria in the kidneys compared to the supplemented mice.

Cytokines and chemokines are crucial mediators of inflammatory signaling in UTI [26]. The neutrophil chemoattractants MIP-2 and KC and the monocyte chemoattractant MCP-1 correlated with bacterial load in supplemented control mice bladders. These findings are in line with other studies demonstrating a similar correlation in the mouse bladder [9]. Interestingly, such correlation was not found for any of the cytokines or chemokines tested in bladders of vitamin D-deficient mice. Our results therefore suggest either a direct or an indirect influence of vitamin D on the local immune response of the urothelium.

We speculate that the vitamin D-deficient mice elicit an earlier immune response compared to the supplemented control mice. This early peak is disrupted however, by more active bacterial invasion and thereby immune evasion into the superficial facet cells. In the supplemented mice on the other hand, the continuous adherence by *E*. *coli* to the luminal side of the epithelial surface will allow for constant immune stimulation. This is in line with the tendency to higher total cytokine and chemokine levels in this group.

Knowing that vitamin D can induce an immune response, one would expect the host to have developed means of increasing vitamin D signaling in response to infection. We therefore investigated if the vitamin D system itself could react to infectious stimuli to promote such an immune response. Uropathogenic *E*. *coli* induced increased VDR levels and caused nuclear translocation in mouse and human bladder cells independently of vitamin D, while vitamin D was necessary for VDR transcriptional activity upon bacterial infection. Observation in the gut of mice infected by *Salmonella typhimurium* showed a similar VDR increase, but in contrast to our study also VDR transcriptional activity independent of vitamin D [12]. This difference may be due to the high concentrations of bacterial metabolites in the gut, known to participate in the control of vitamin D signaling. Our findings are in line with our previously published data demonstrating increased cathelicidin, an indicator of VDR activation, after infection of human bladder biopsies, only when patients were previously supplemented with vitamin D [11].

In summary, mice with low serum levels of vitamin D had more intraepithelial bacteria in their bladders and a dysregulated cytokine response to infection. Compared with the vitamin D-supplemented group, they were more prone to ascending infection to the upper urinary tract.

In addition, VDR was upregulated in a time-dependent manner and increased translocation into the nucleus was observed in both human and mouse bladder cells. Our findings suggest that vitamin D has an additional protective role in the defense against invading *E*. *coli* in the urinary tract, which is not attributed to the antimicrobial peptide cathelicidin. We therefore conclude that patients with low serum levels of 25D_3_ may be at increased risk of recurrent UTI and should be supplemented until sufficient vitamin D levels are achieved.

## Supporting information

S1 File VitaminD_eColi reproducible Research Archive. This file contains all scripts (CellProfiler v2.1.1 and MATLAB2016a) and data necessary to reproduce the information shown in Fig 3.
(ZIP)
