# Supplementary figures and images for: Vitamin D-deficient mice have more invasive urinary tract infection (part 2 of 2)
Source: PLoS One. 2017 Jul 27;12(7):e0180810. doi: 10.1371/journal.pone.0180810 (PMC5531565; doi:10.1371/journal.pone.0180810)

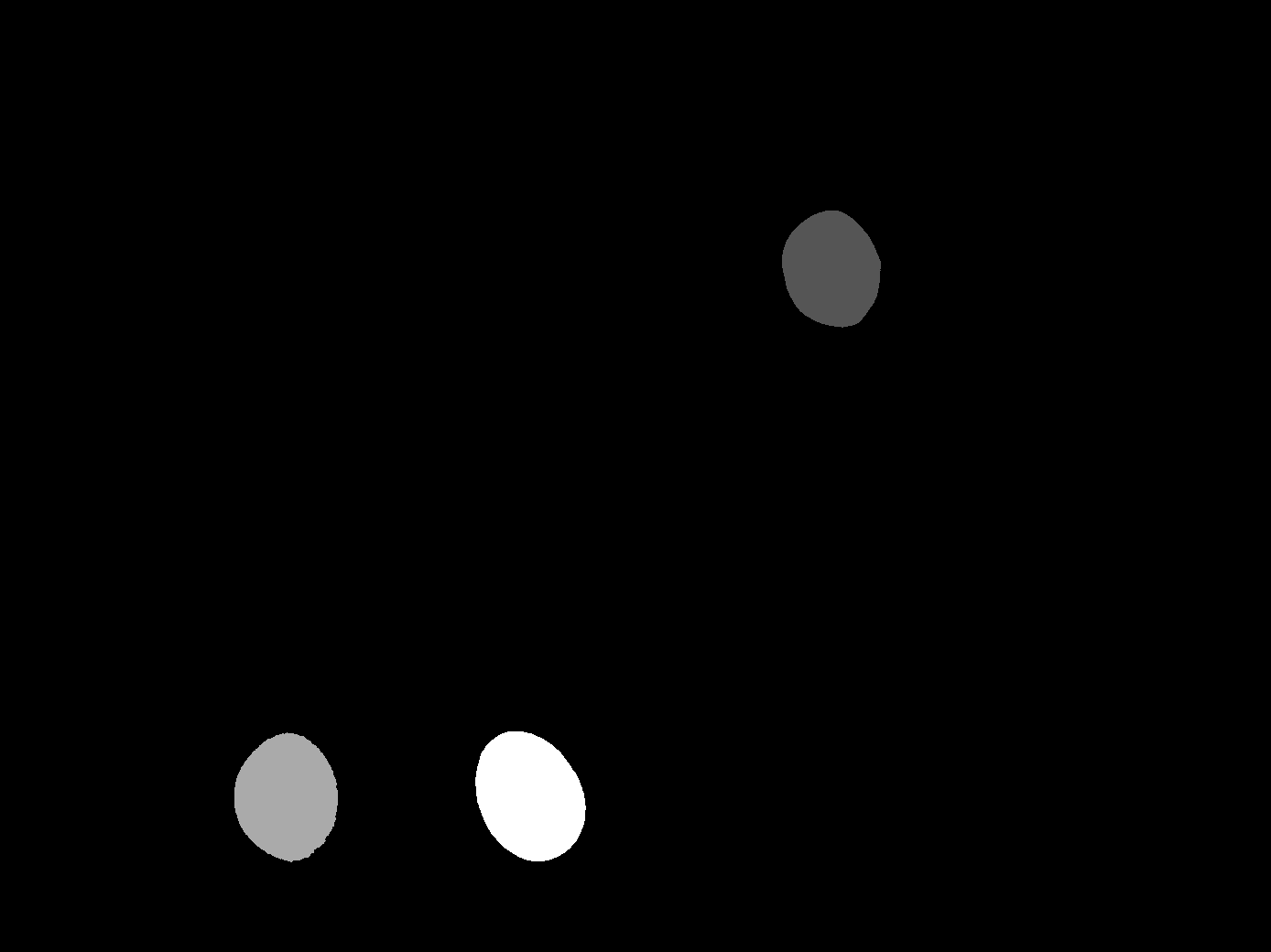

Supplement: S1 File — This file contains all scripts (CellProfiler v2.1.1 and MATLAB2016a) and data necessary to reproduce the information shown in Fig 3. (ZIP) [file pone.0180810.s001.zip › vitaminD_eColi_reproducibleResearchArchive/Results2016/B_19_c0_seg.tif]

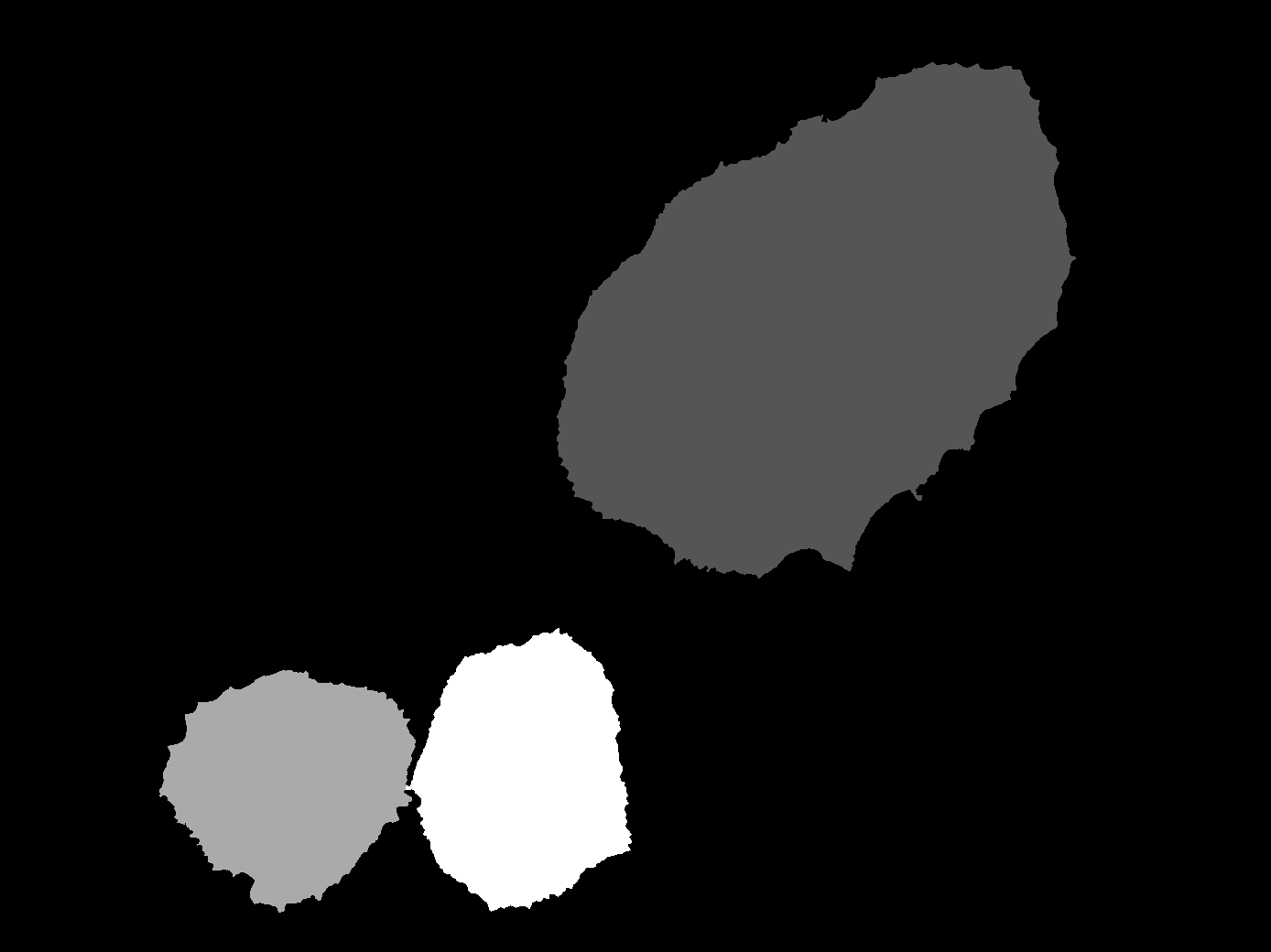

Supplement: S1 File — This file contains all scripts (CellProfiler v2.1.1 and MATLAB2016a) and data necessary to reproduce the information shown in Fig 3. (ZIP) [file pone.0180810.s001.zip › vitaminD_eColi_reproducibleResearchArchive/Results2016/B_19_c2_seg.tif]

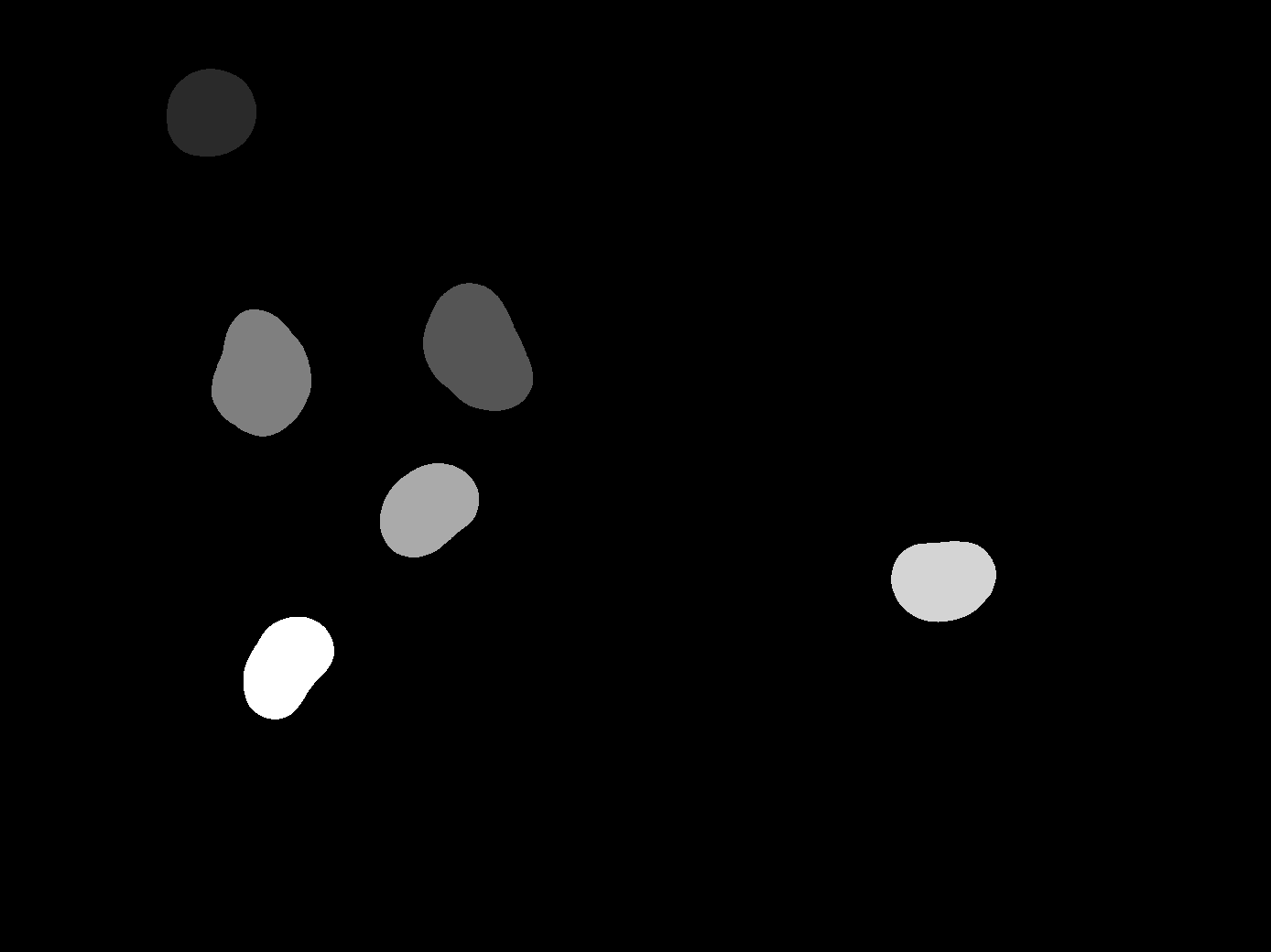

Supplement: S1 File — This file contains all scripts (CellProfiler v2.1.1 and MATLAB2016a) and data necessary to reproduce the information shown in Fig 3. (ZIP) [file pone.0180810.s001.zip › vitaminD_eColi_reproducibleResearchArchive/Results2016/B_1_c0_seg.tif]

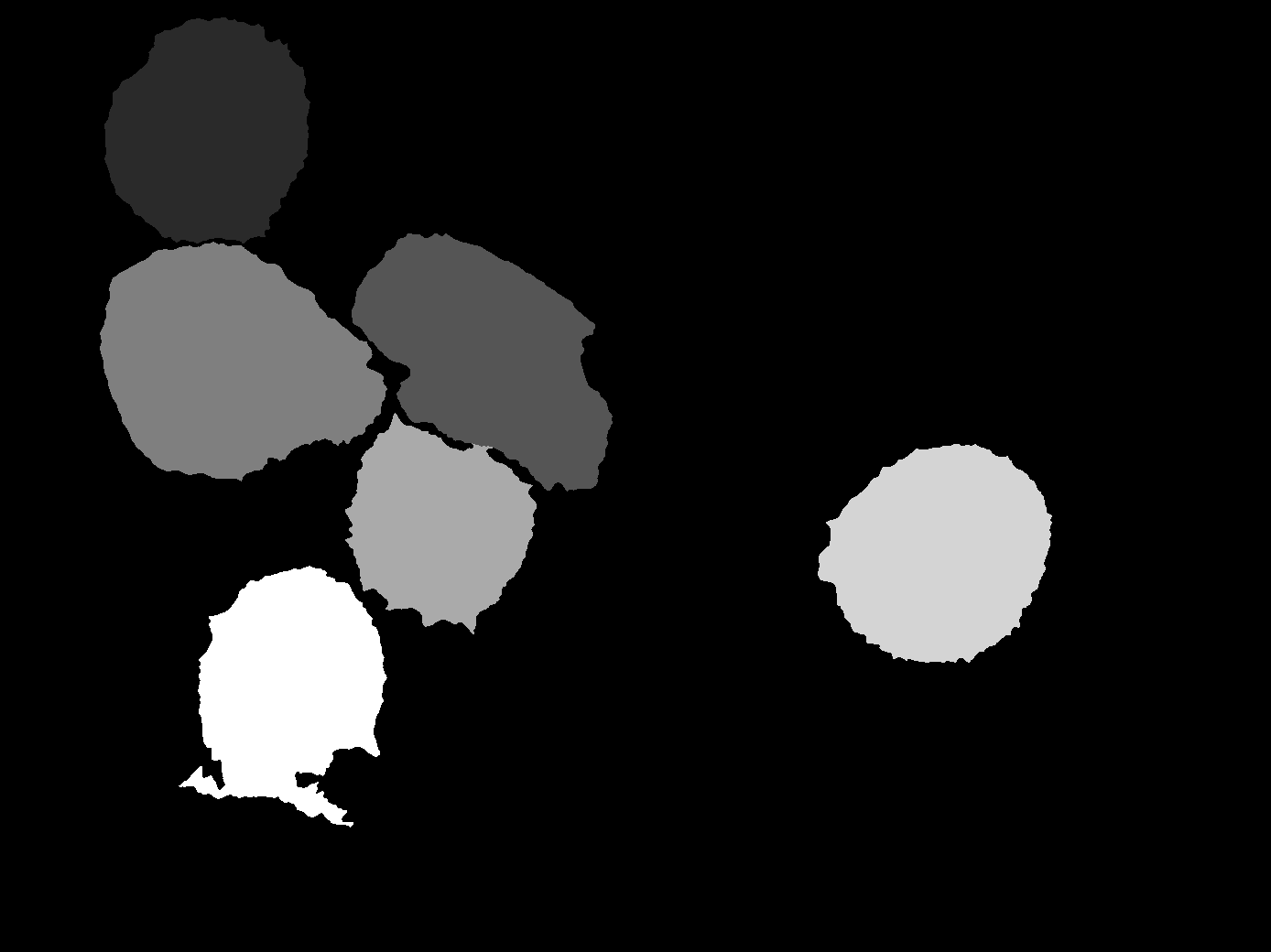

Supplement: S1 File — This file contains all scripts (CellProfiler v2.1.1 and MATLAB2016a) and data necessary to reproduce the information shown in Fig 3. (ZIP) [file pone.0180810.s001.zip › vitaminD_eColi_reproducibleResearchArchive/Results2016/B_1_c2_seg.tif]

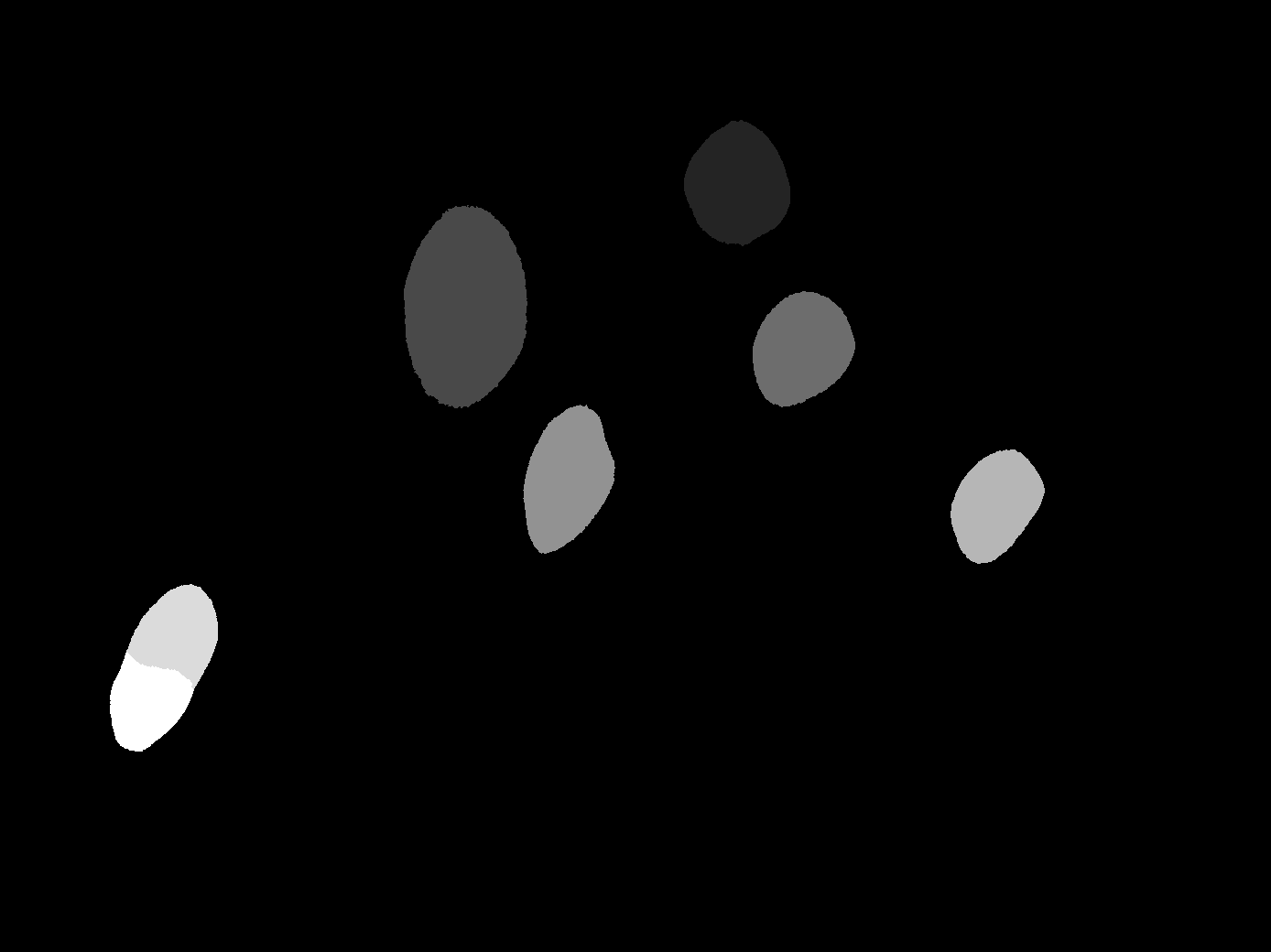

Supplement: S1 File — This file contains all scripts (CellProfiler v2.1.1 and MATLAB2016a) and data necessary to reproduce the information shown in Fig 3. (ZIP) [file pone.0180810.s001.zip › vitaminD_eColi_reproducibleResearchArchive/Results2016/B_20_c0_seg.tif]

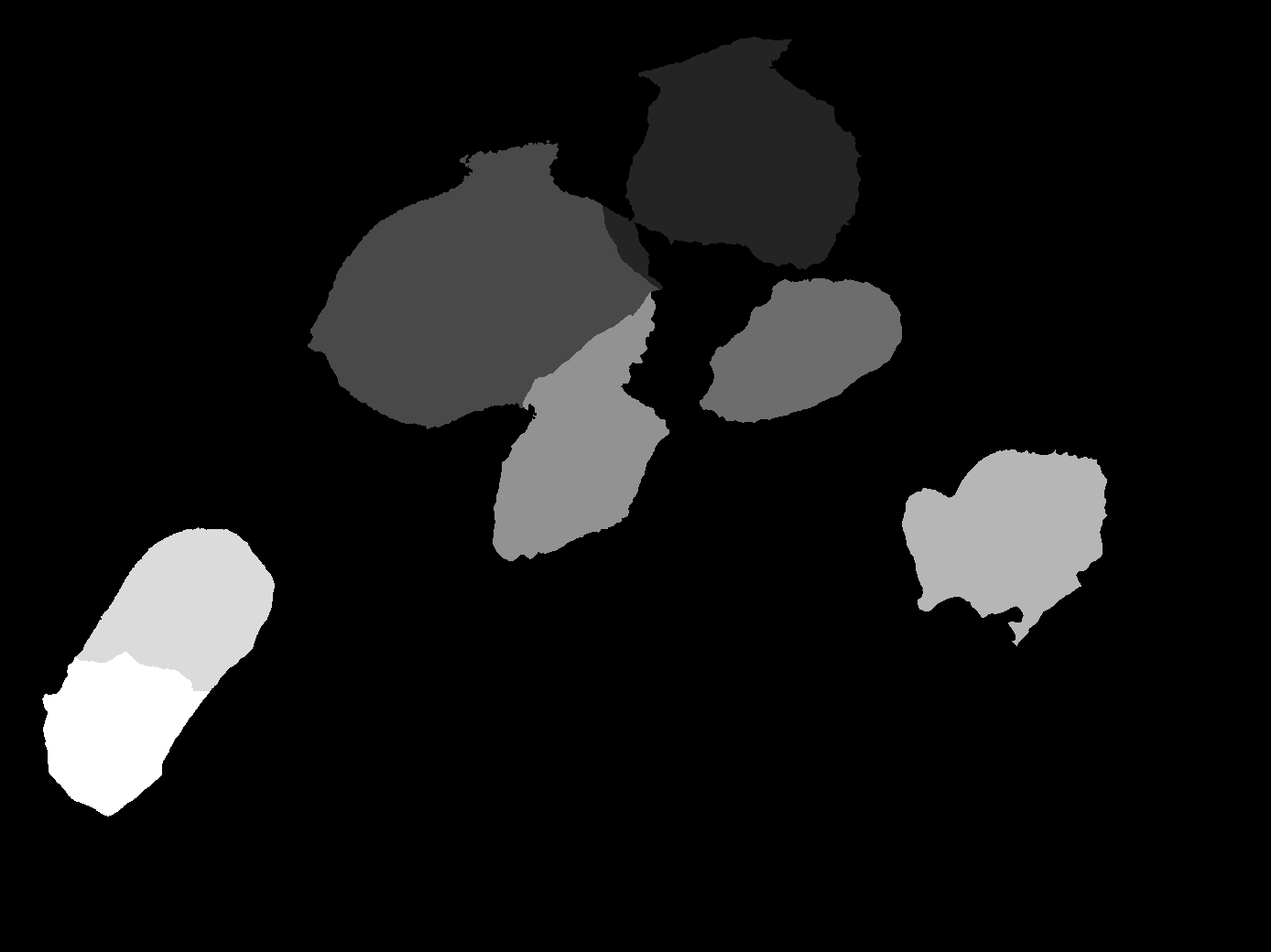

Supplement: S1 File — This file contains all scripts (CellProfiler v2.1.1 and MATLAB2016a) and data necessary to reproduce the information shown in Fig 3. (ZIP) [file pone.0180810.s001.zip › vitaminD_eColi_reproducibleResearchArchive/Results2016/B_20_c2_seg.tif]

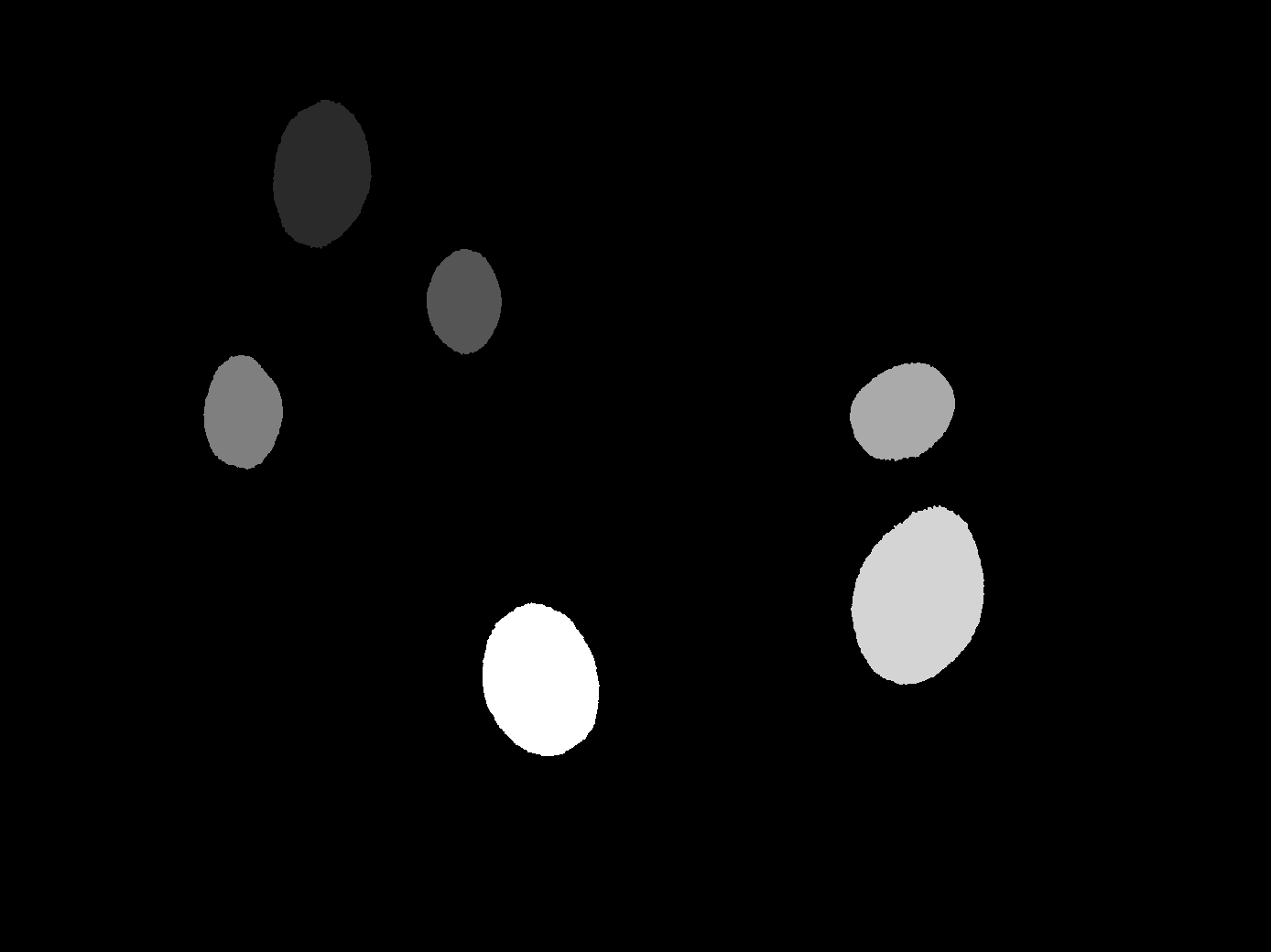

Supplement: S1 File — This file contains all scripts (CellProfiler v2.1.1 and MATLAB2016a) and data necessary to reproduce the information shown in Fig 3. (ZIP) [file pone.0180810.s001.zip › vitaminD_eColi_reproducibleResearchArchive/Results2016/B_21_c0_seg.tif]

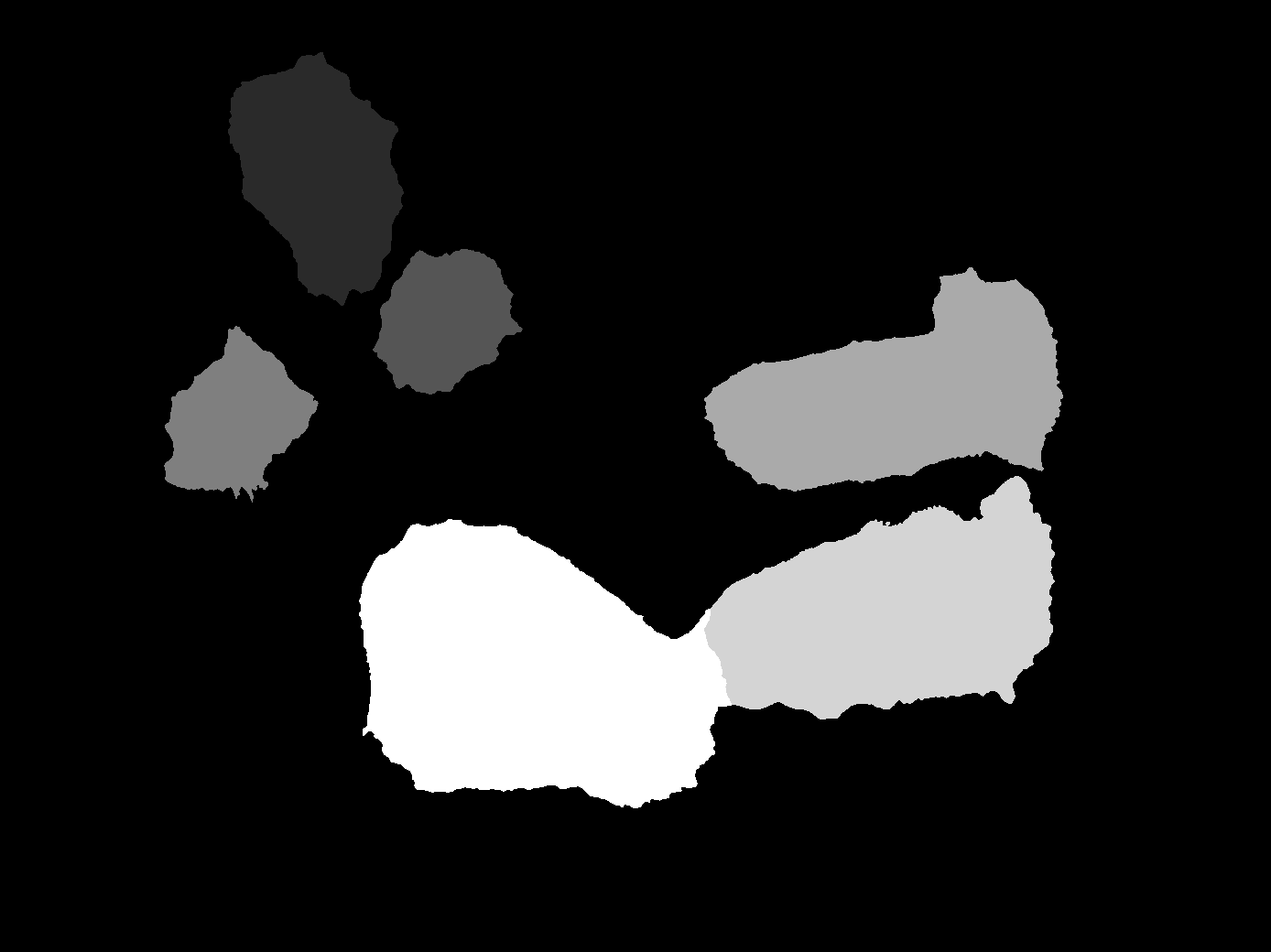

Supplement: S1 File — This file contains all scripts (CellProfiler v2.1.1 and MATLAB2016a) and data necessary to reproduce the information shown in Fig 3. (ZIP) [file pone.0180810.s001.zip › vitaminD_eColi_reproducibleResearchArchive/Results2016/B_21_c2_seg.tif]

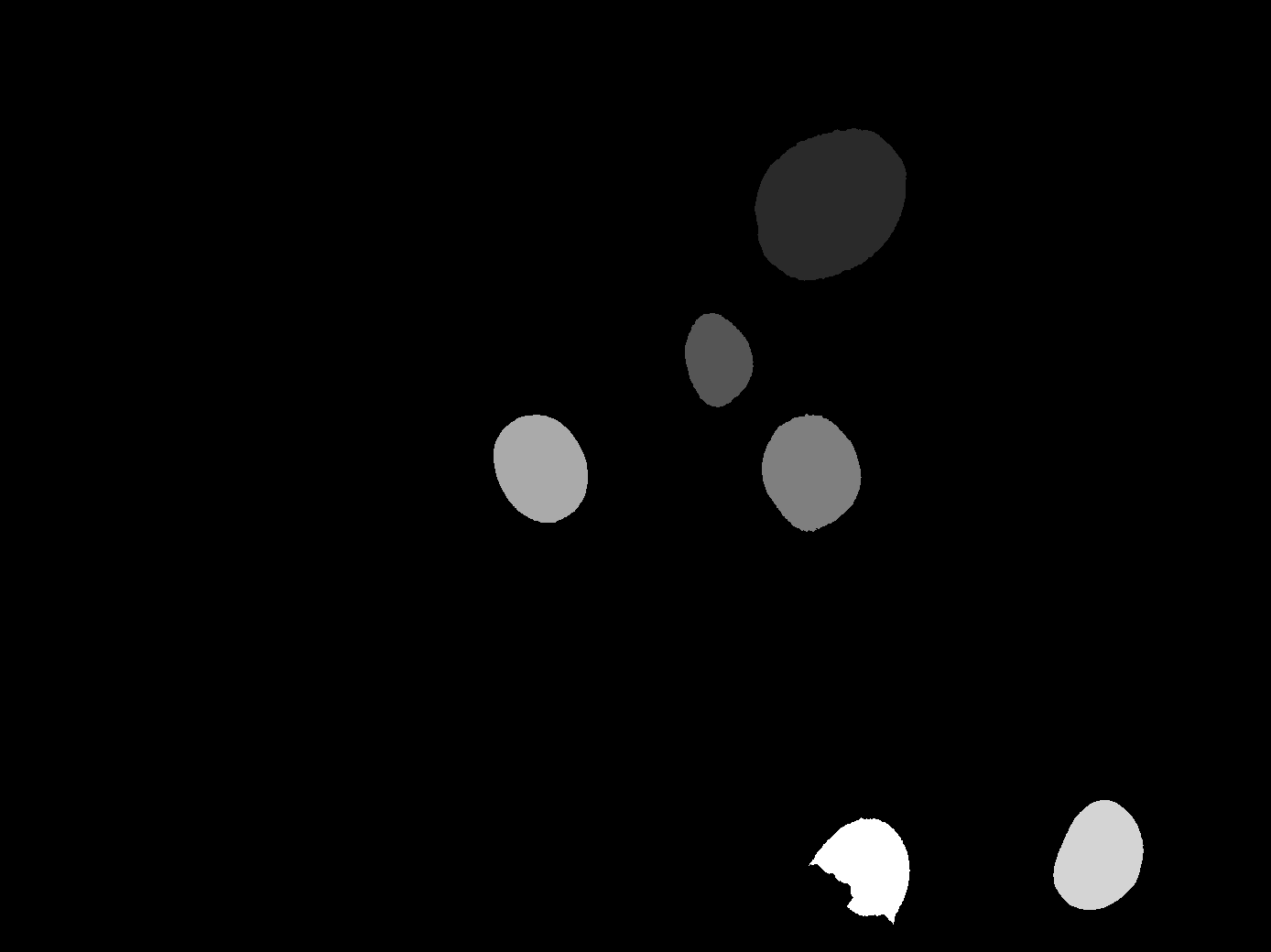

Supplement: S1 File — This file contains all scripts (CellProfiler v2.1.1 and MATLAB2016a) and data necessary to reproduce the information shown in Fig 3. (ZIP) [file pone.0180810.s001.zip › vitaminD_eColi_reproducibleResearchArchive/Results2016/B_22_c0_seg.tif]

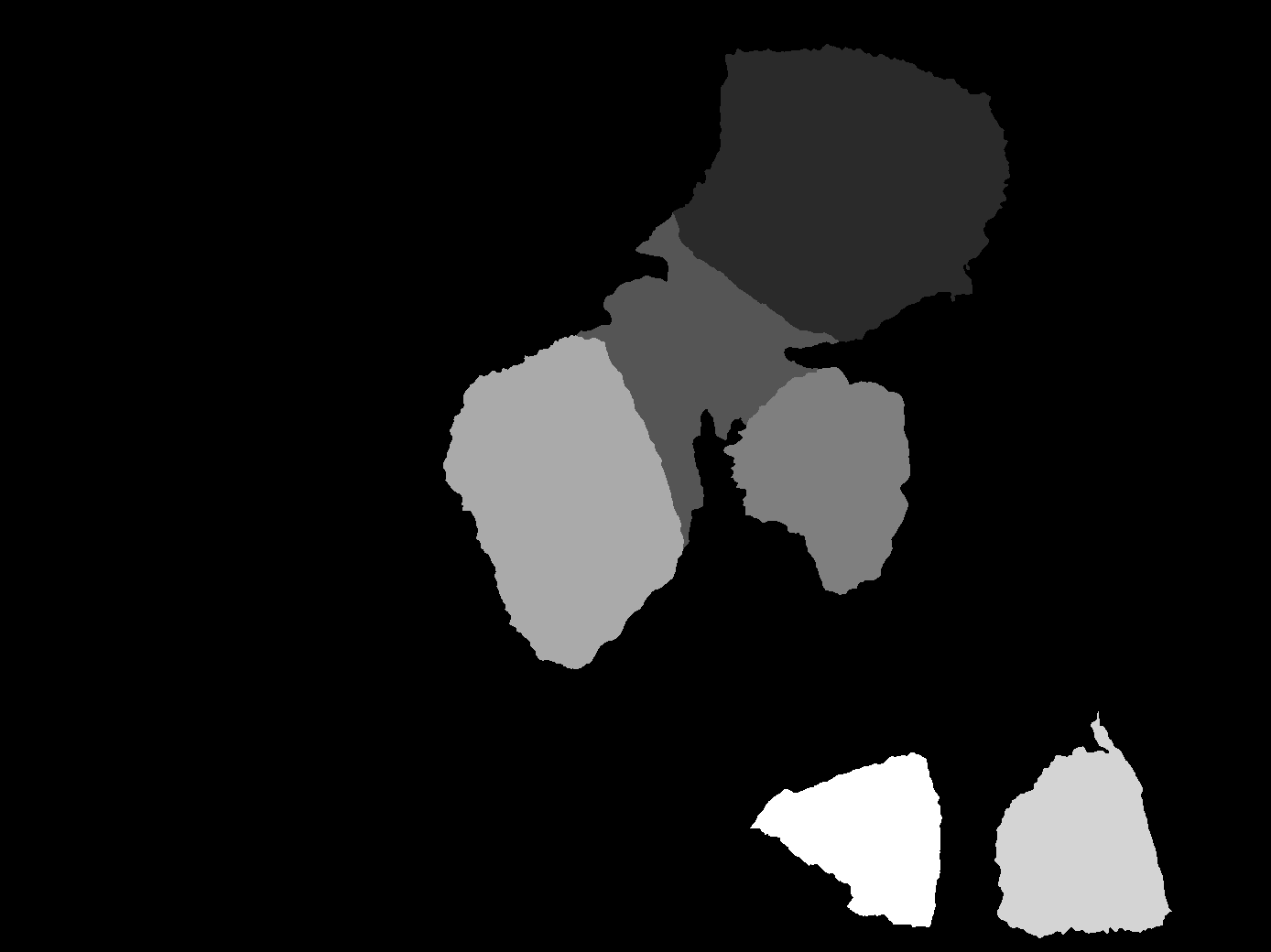

Supplement: S1 File — This file contains all scripts (CellProfiler v2.1.1 and MATLAB2016a) and data necessary to reproduce the information shown in Fig 3. (ZIP) [file pone.0180810.s001.zip › vitaminD_eColi_reproducibleResearchArchive/Results2016/B_22_c2_seg.tif]

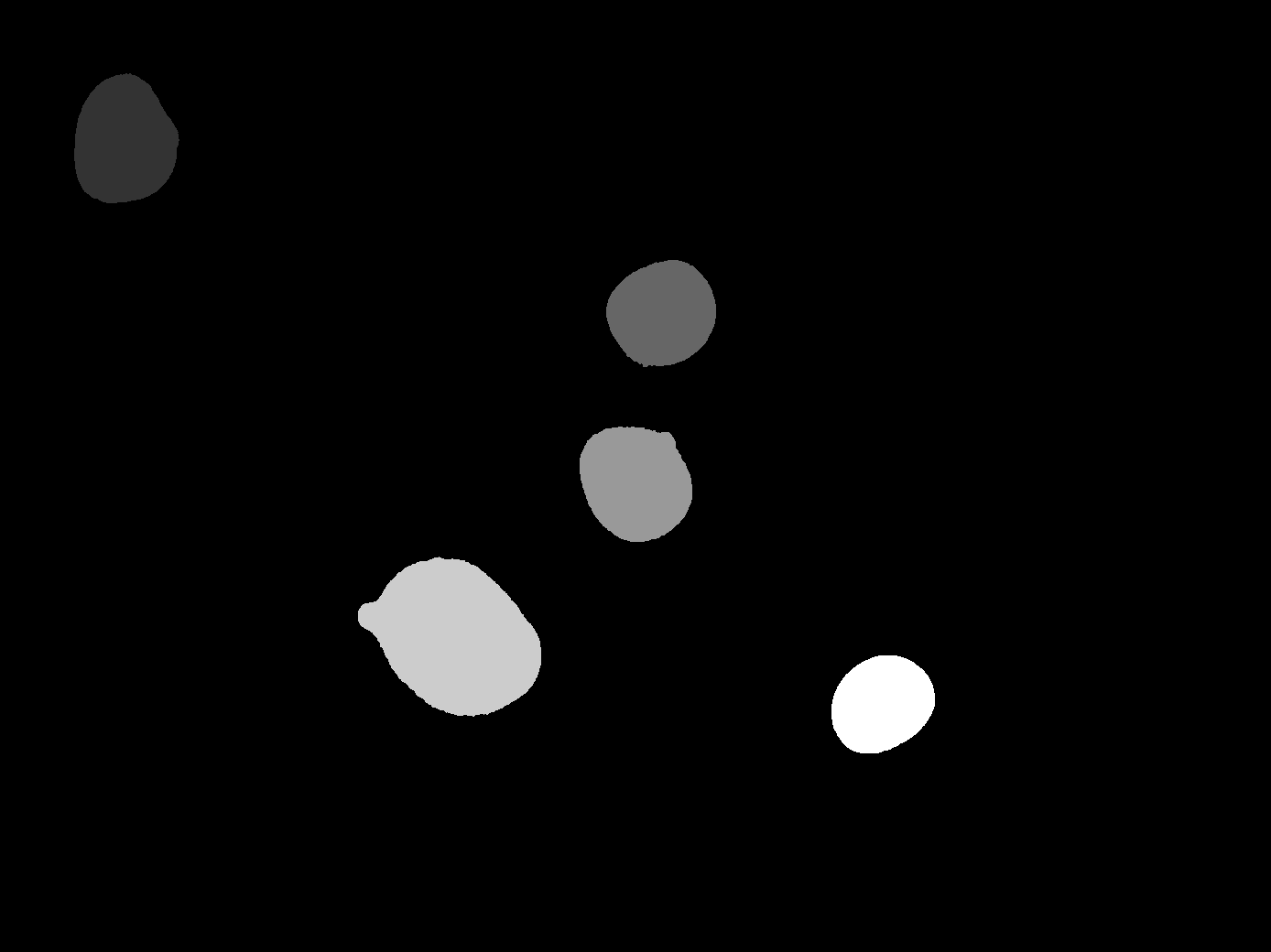

Supplement: S1 File — This file contains all scripts (CellProfiler v2.1.1 and MATLAB2016a) and data necessary to reproduce the information shown in Fig 3. (ZIP) [file pone.0180810.s001.zip › vitaminD_eColi_reproducibleResearchArchive/Results2016/B_23_c0_seg.tif]

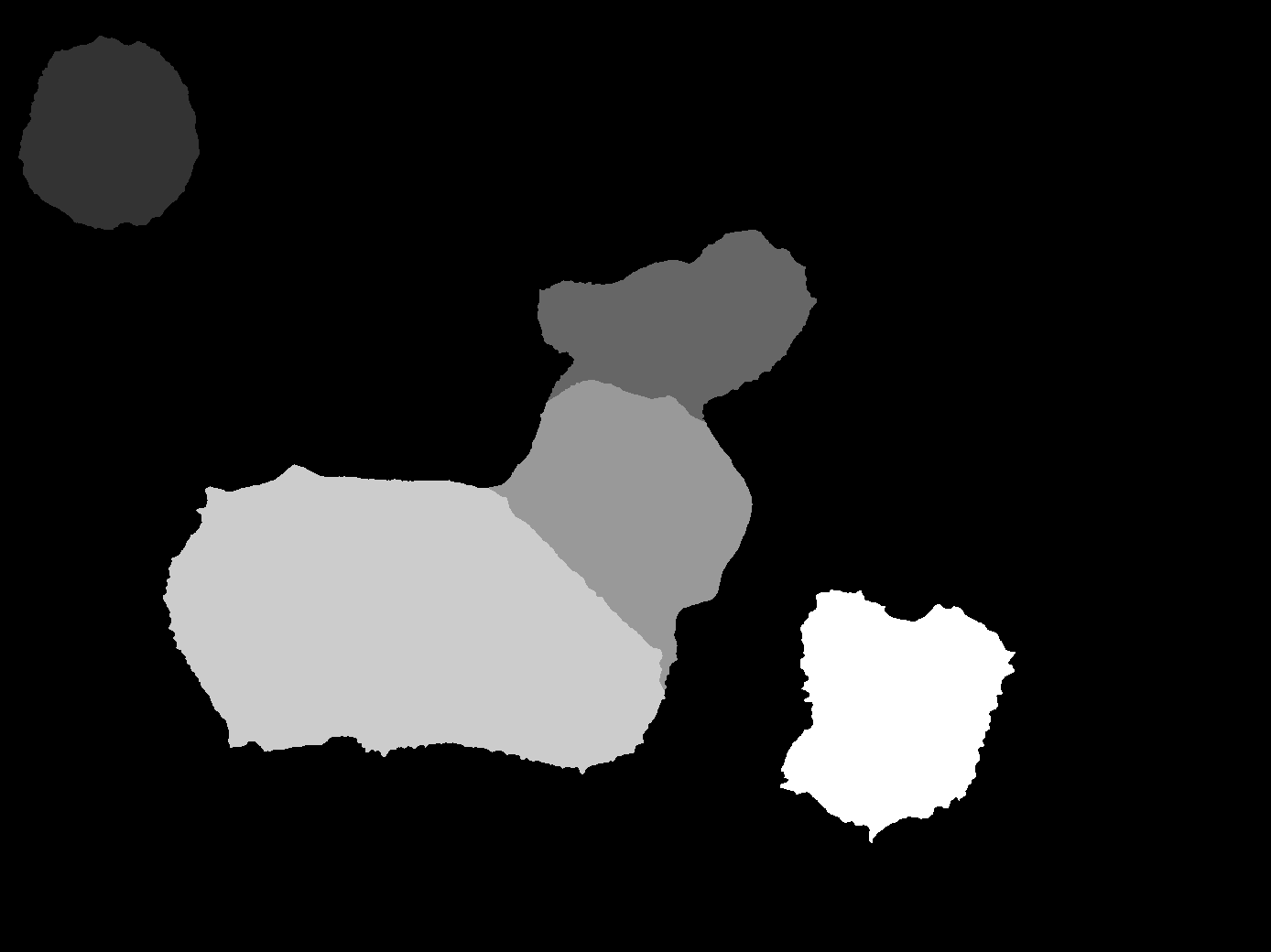

Supplement: S1 File — This file contains all scripts (CellProfiler v2.1.1 and MATLAB2016a) and data necessary to reproduce the information shown in Fig 3. (ZIP) [file pone.0180810.s001.zip › vitaminD_eColi_reproducibleResearchArchive/Results2016/B_23_c2_seg.tif]

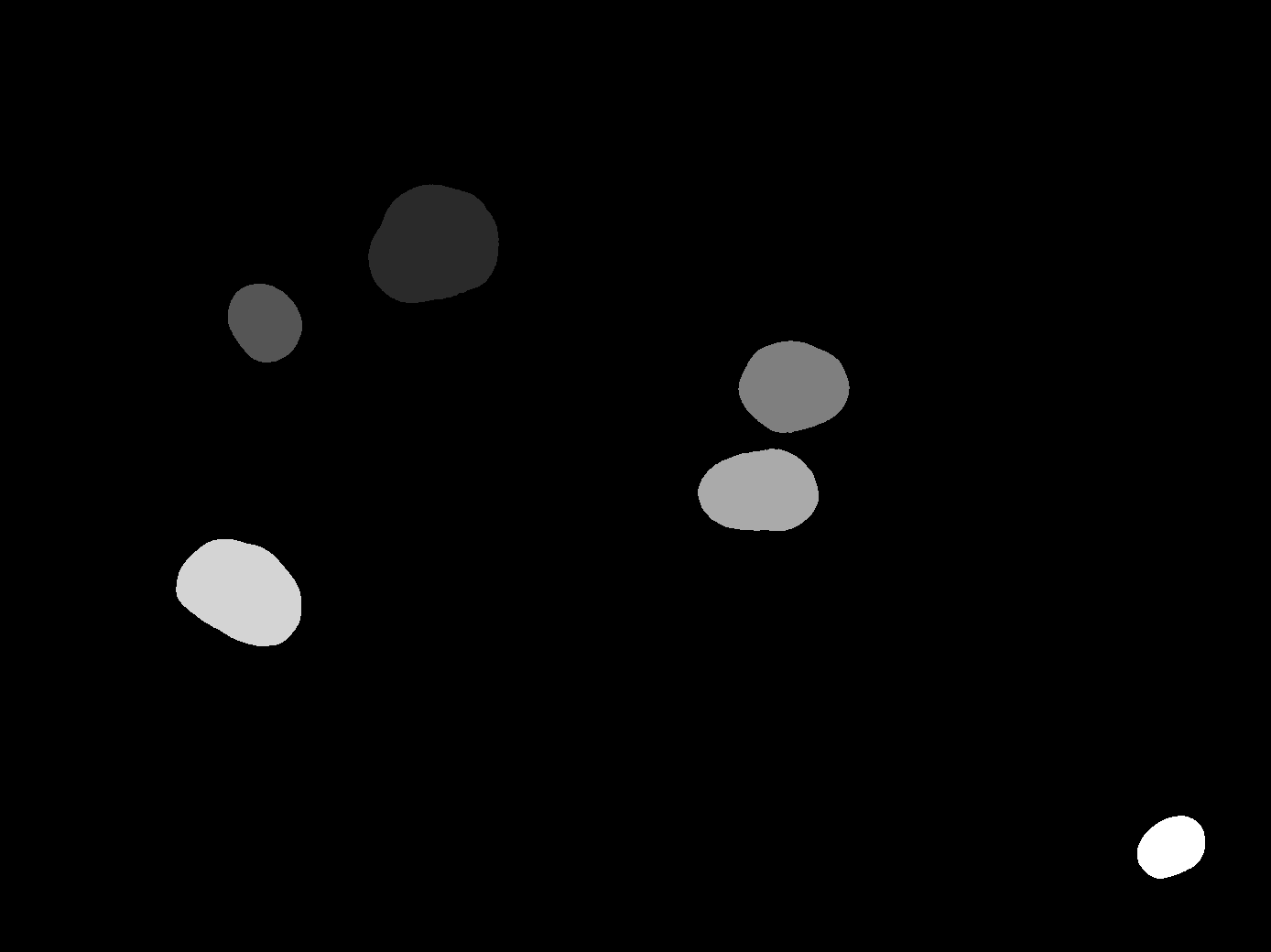

Supplement: S1 File — This file contains all scripts (CellProfiler v2.1.1 and MATLAB2016a) and data necessary to reproduce the information shown in Fig 3. (ZIP) [file pone.0180810.s001.zip › vitaminD_eColi_reproducibleResearchArchive/Results2016/B_24_c0_seg.tif]

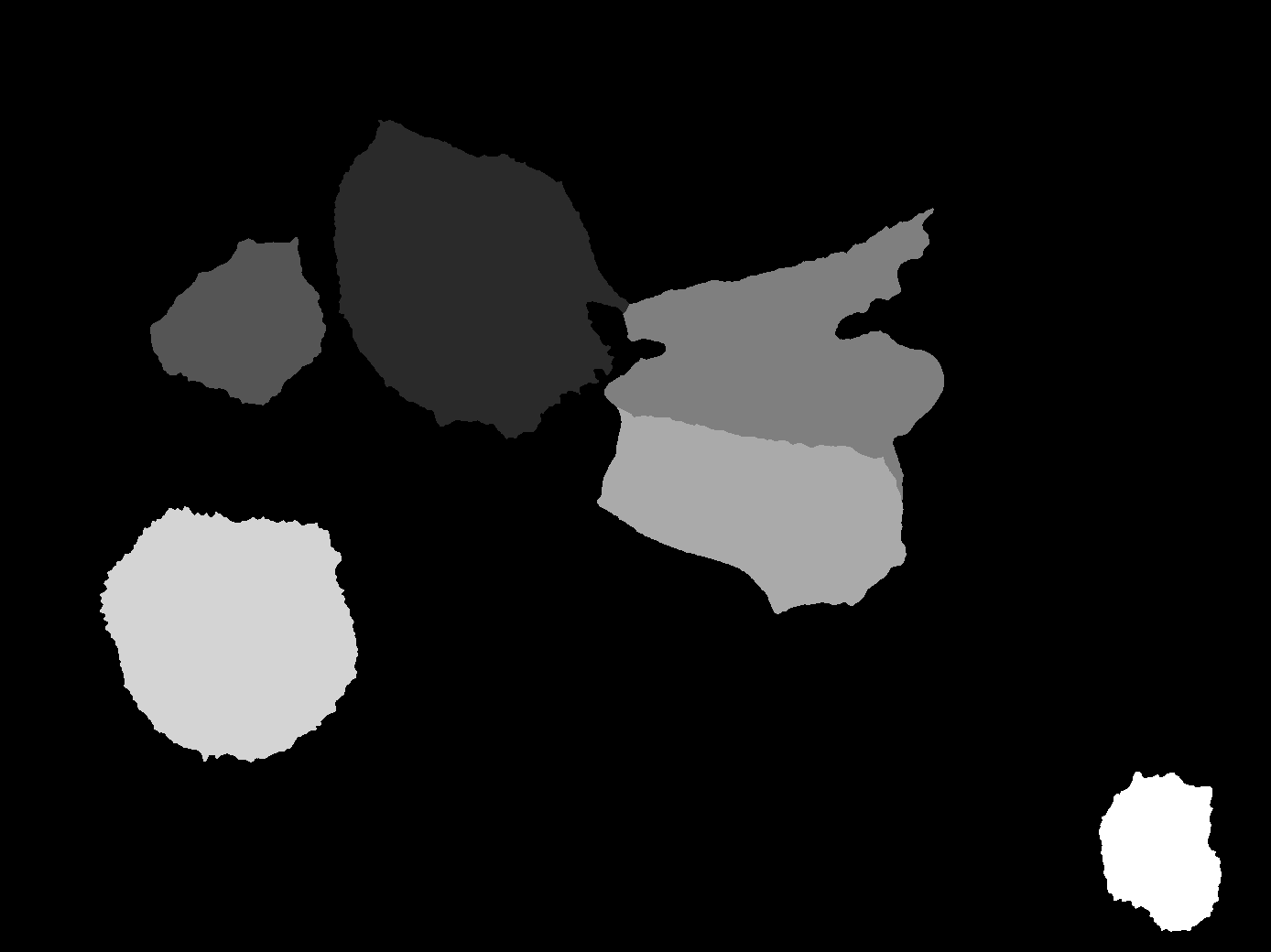

Supplement: S1 File — This file contains all scripts (CellProfiler v2.1.1 and MATLAB2016a) and data necessary to reproduce the information shown in Fig 3. (ZIP) [file pone.0180810.s001.zip › vitaminD_eColi_reproducibleResearchArchive/Results2016/B_24_c2_seg.tif]

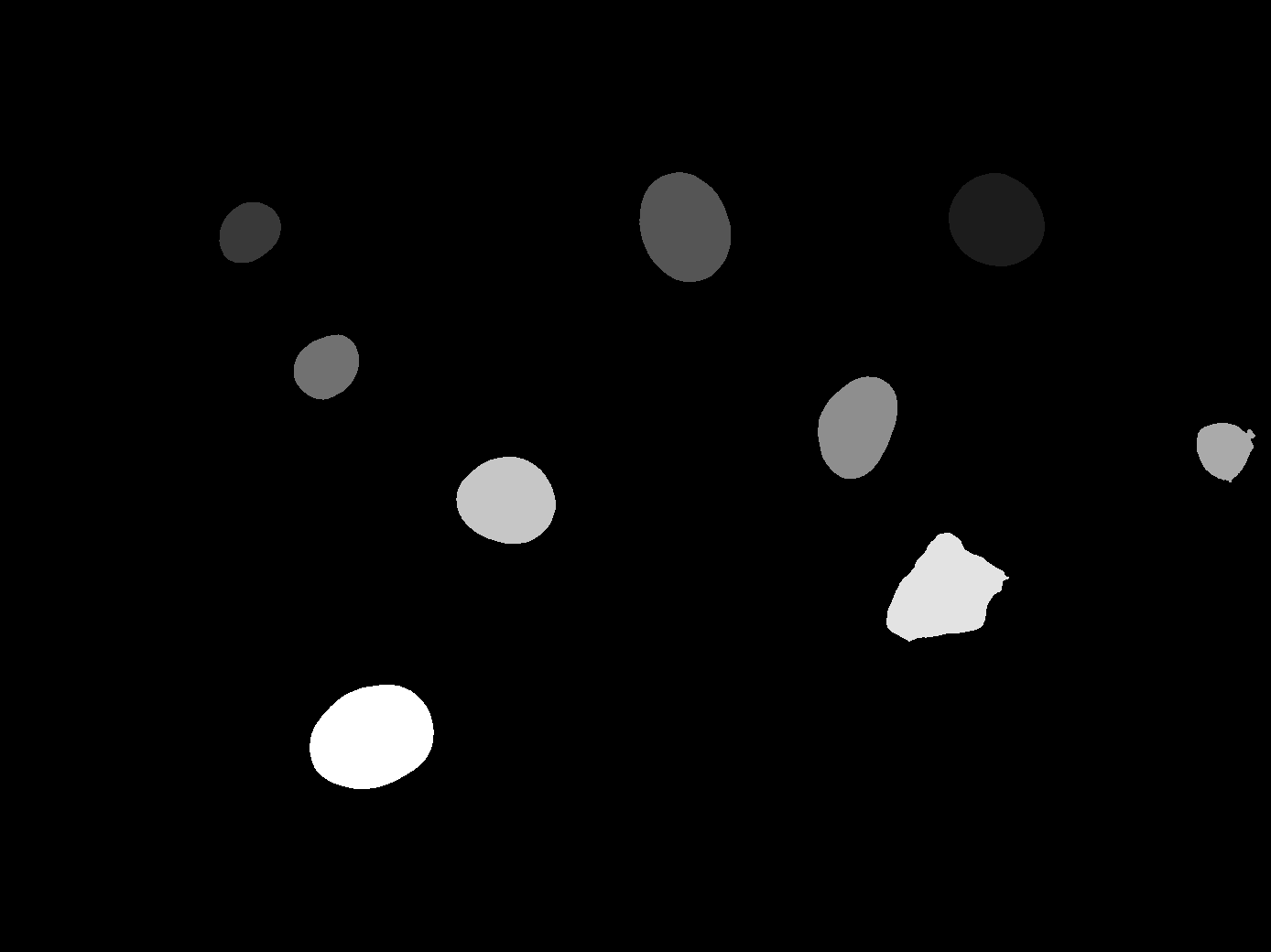

Supplement: S1 File — This file contains all scripts (CellProfiler v2.1.1 and MATLAB2016a) and data necessary to reproduce the information shown in Fig 3. (ZIP) [file pone.0180810.s001.zip › vitaminD_eColi_reproducibleResearchArchive/Results2016/B_25_c0_seg.tif]

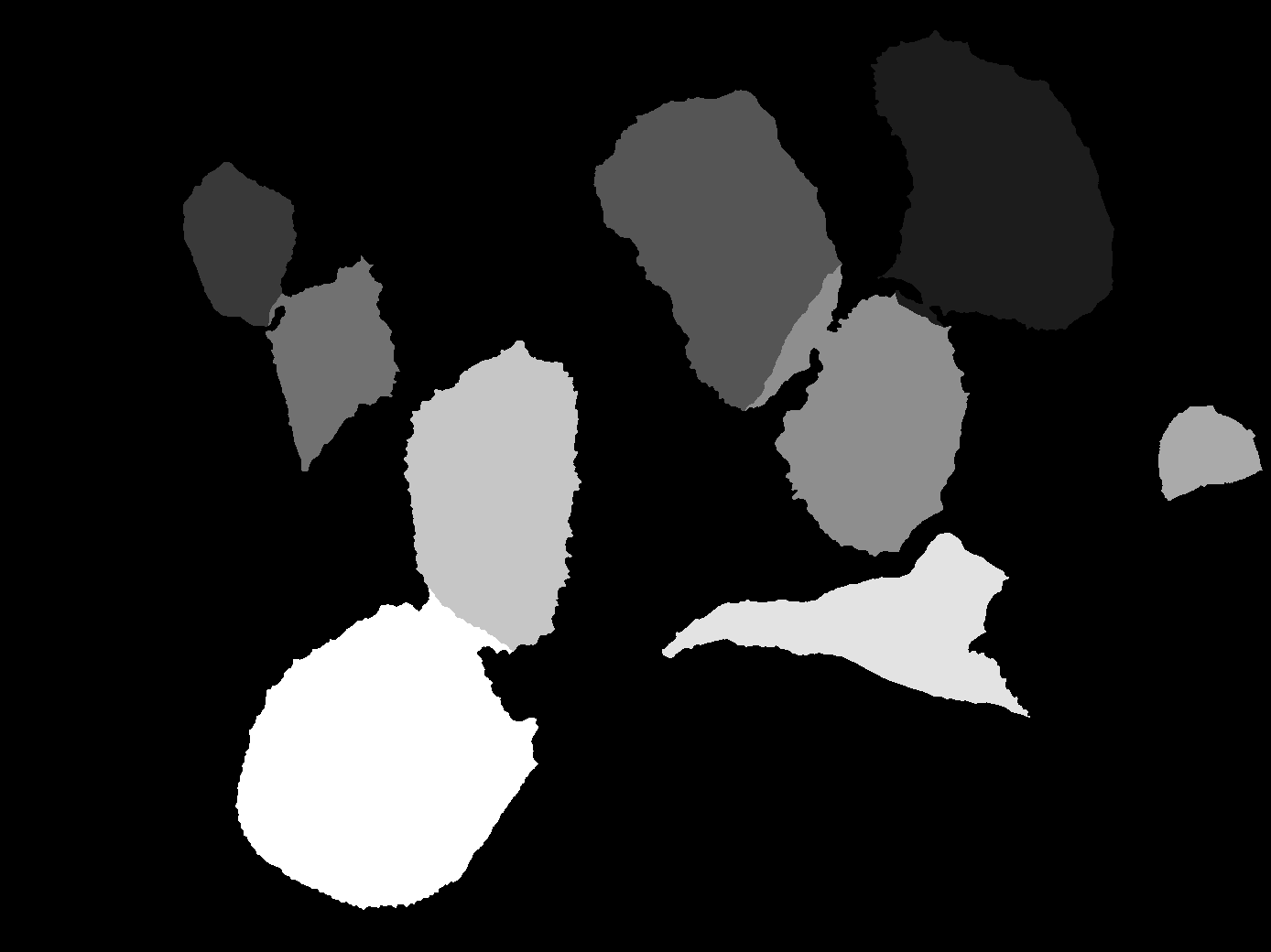

Supplement: S1 File — This file contains all scripts (CellProfiler v2.1.1 and MATLAB2016a) and data necessary to reproduce the information shown in Fig 3. (ZIP) [file pone.0180810.s001.zip › vitaminD_eColi_reproducibleResearchArchive/Results2016/B_25_c2_seg.tif]

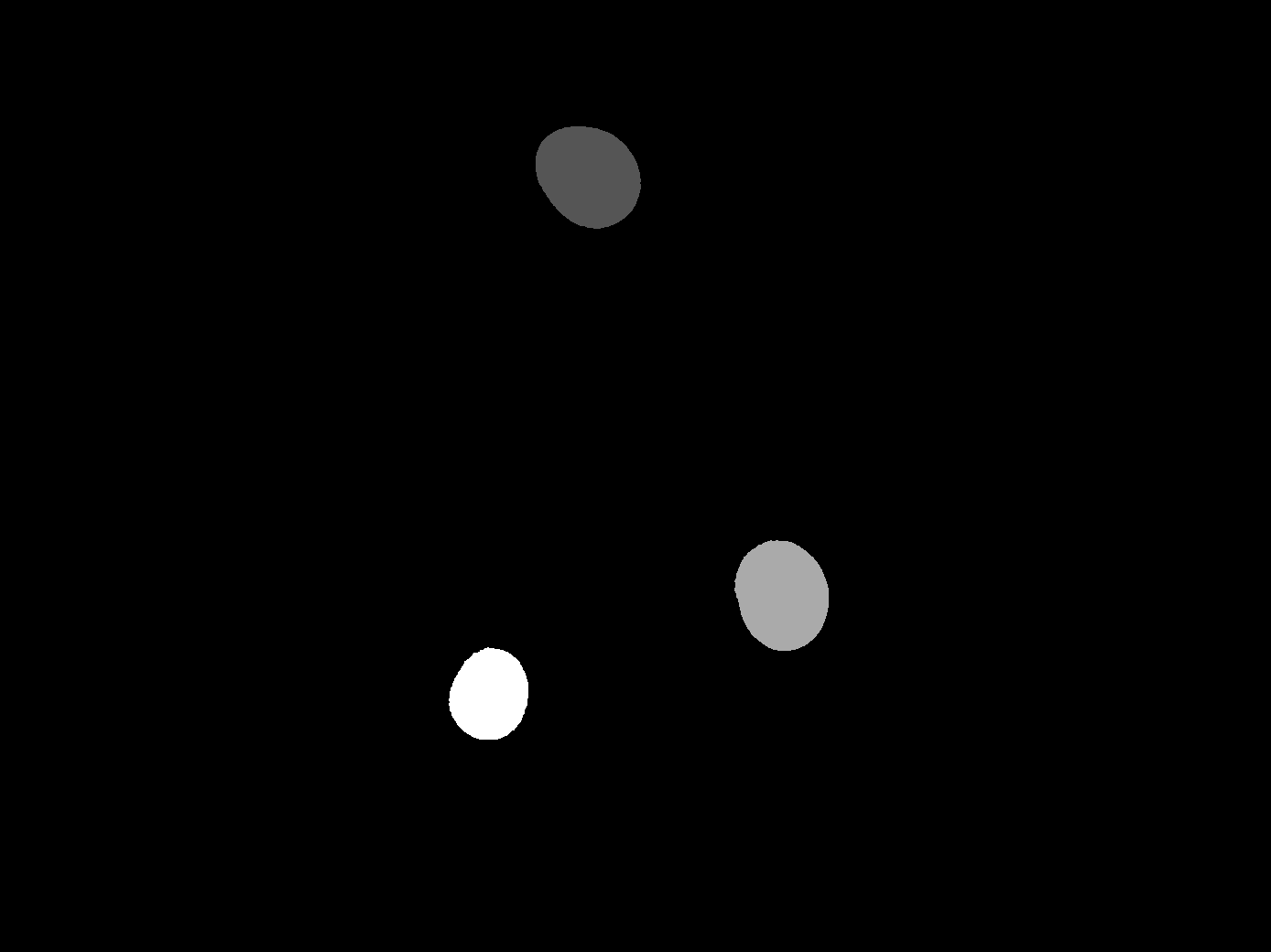

Supplement: S1 File — This file contains all scripts (CellProfiler v2.1.1 and MATLAB2016a) and data necessary to reproduce the information shown in Fig 3. (ZIP) [file pone.0180810.s001.zip › vitaminD_eColi_reproducibleResearchArchive/Results2016/B_26_c0_seg.tif]

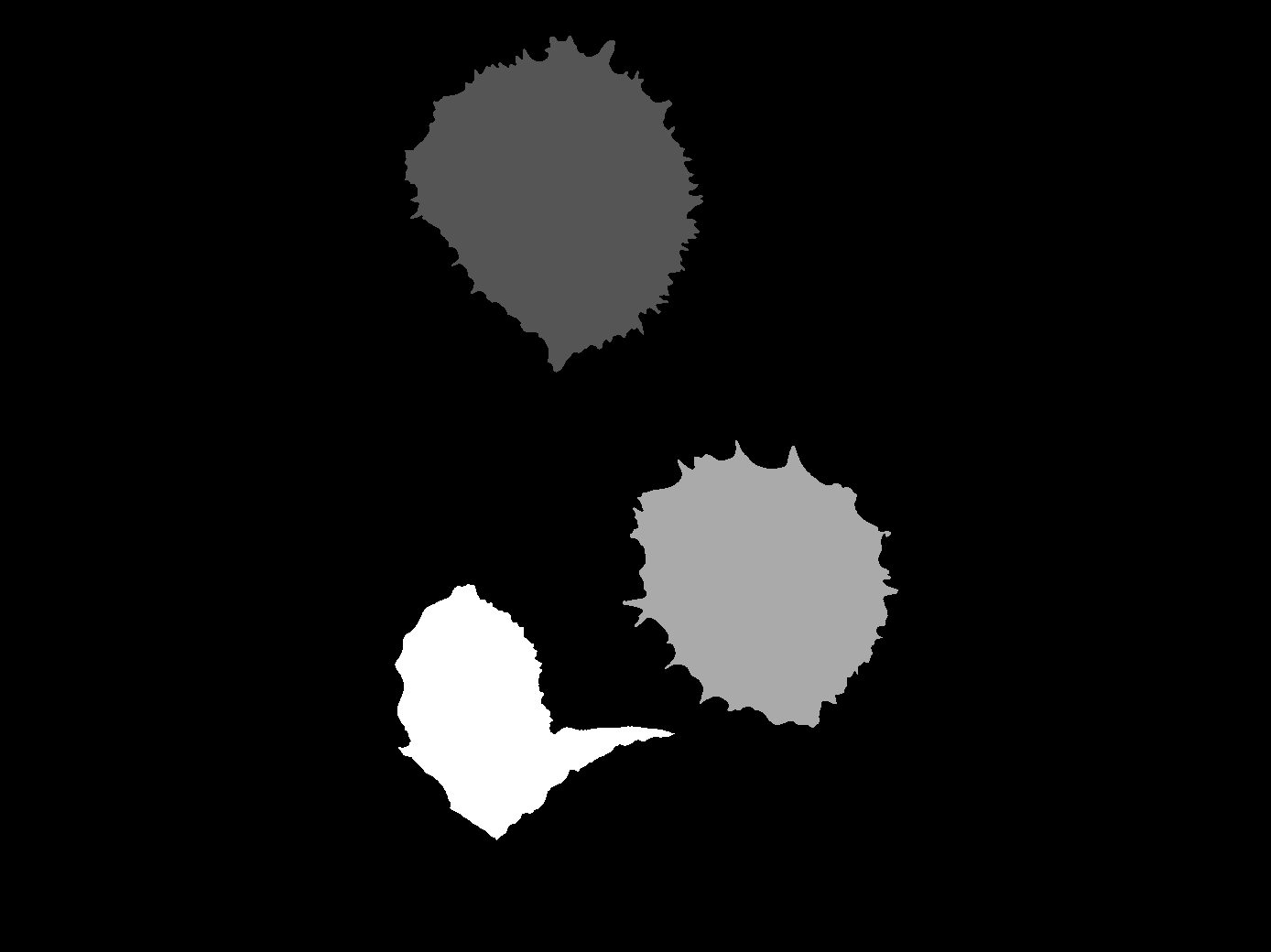

Supplement: S1 File — This file contains all scripts (CellProfiler v2.1.1 and MATLAB2016a) and data necessary to reproduce the information shown in Fig 3. (ZIP) [file pone.0180810.s001.zip › vitaminD_eColi_reproducibleResearchArchive/Results2016/B_26_c2_seg.tif]

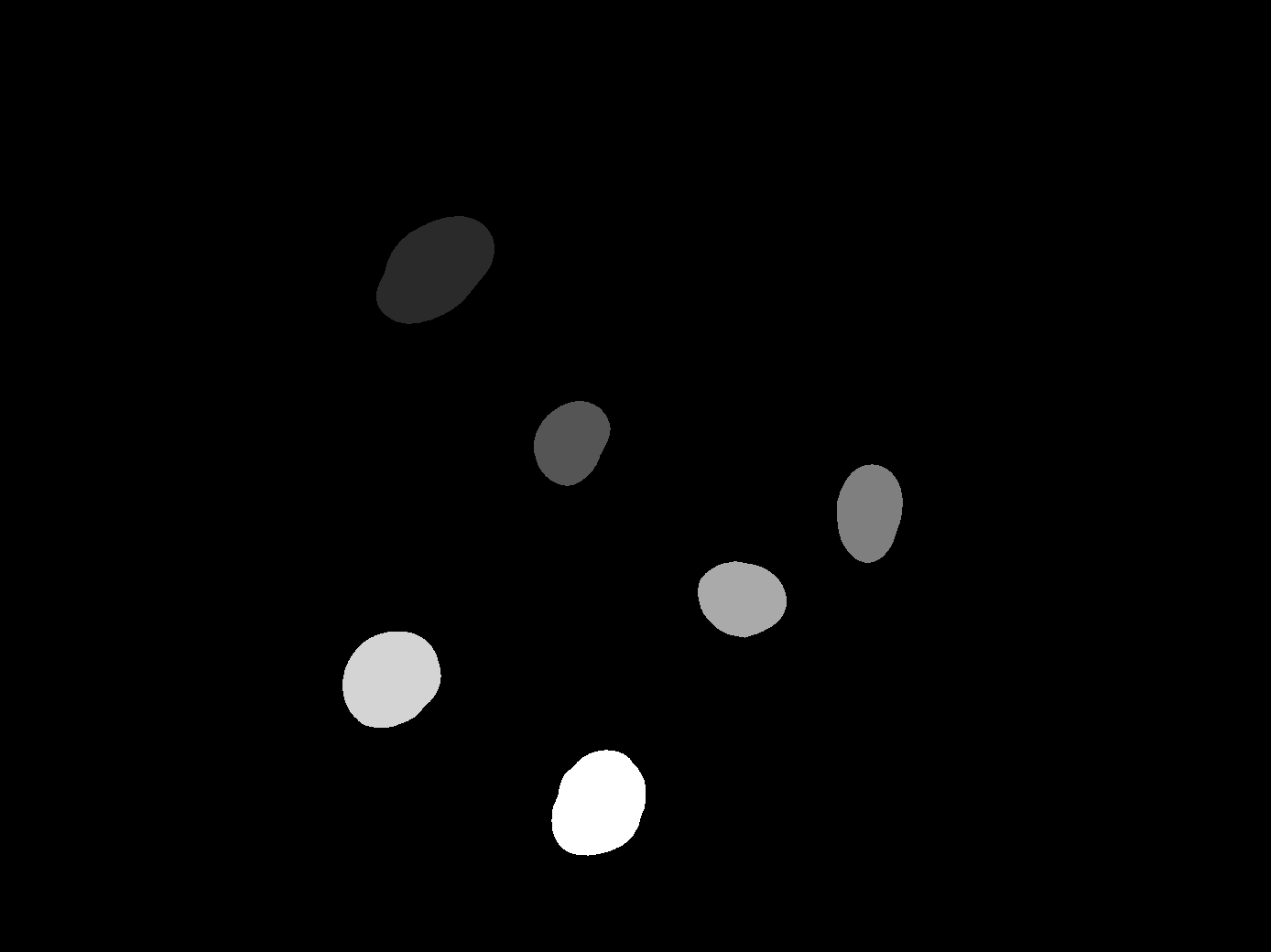

Supplement: S1 File — This file contains all scripts (CellProfiler v2.1.1 and MATLAB2016a) and data necessary to reproduce the information shown in Fig 3. (ZIP) [file pone.0180810.s001.zip › vitaminD_eColi_reproducibleResearchArchive/Results2016/B_27_c0_seg.tif]

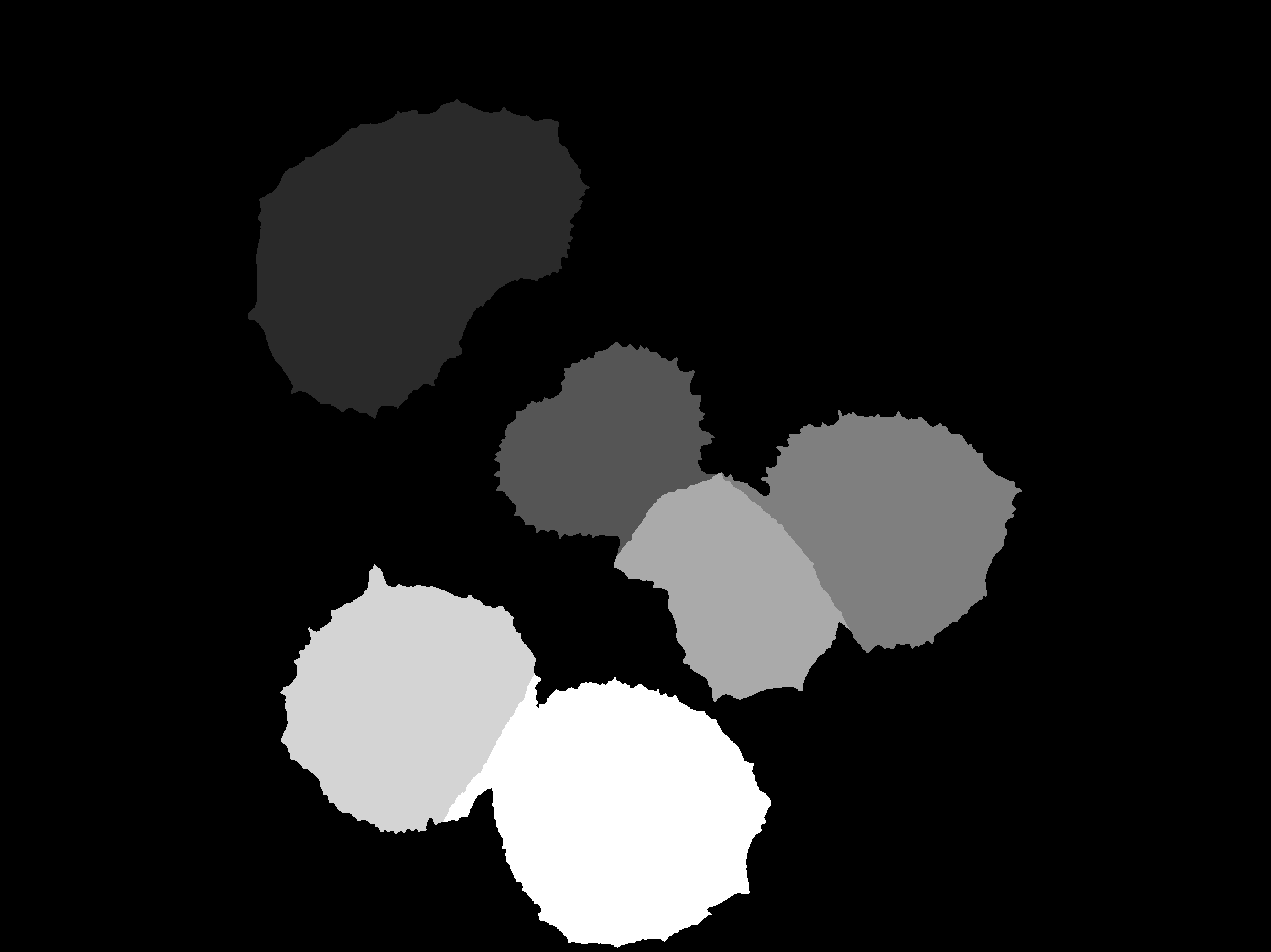

Supplement: S1 File — This file contains all scripts (CellProfiler v2.1.1 and MATLAB2016a) and data necessary to reproduce the information shown in Fig 3. (ZIP) [file pone.0180810.s001.zip › vitaminD_eColi_reproducibleResearchArchive/Results2016/B_27_c2_seg.tif]

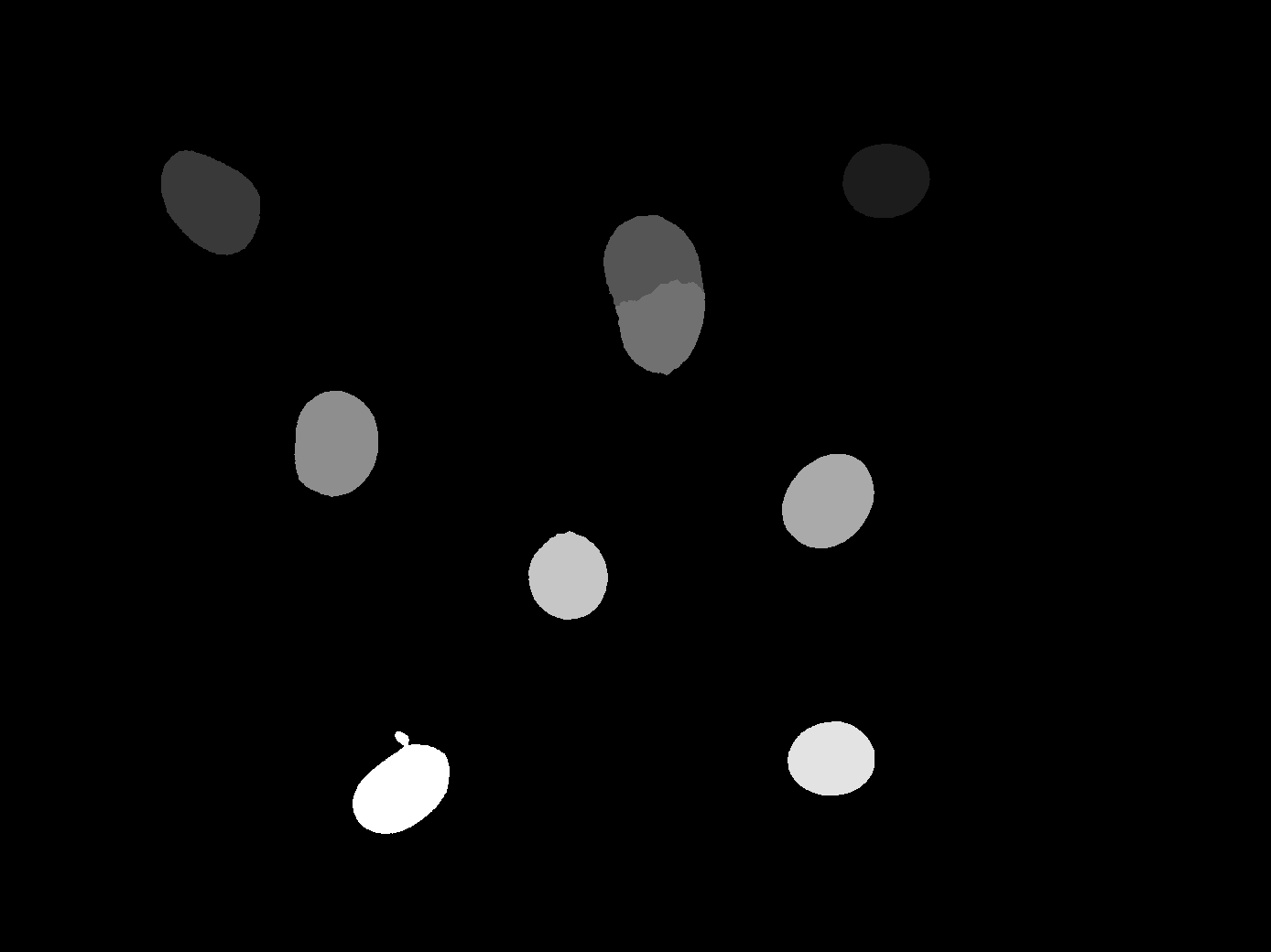

Supplement: S1 File — This file contains all scripts (CellProfiler v2.1.1 and MATLAB2016a) and data necessary to reproduce the information shown in Fig 3. (ZIP) [file pone.0180810.s001.zip › vitaminD_eColi_reproducibleResearchArchive/Results2016/B_28_c0_seg.tif]

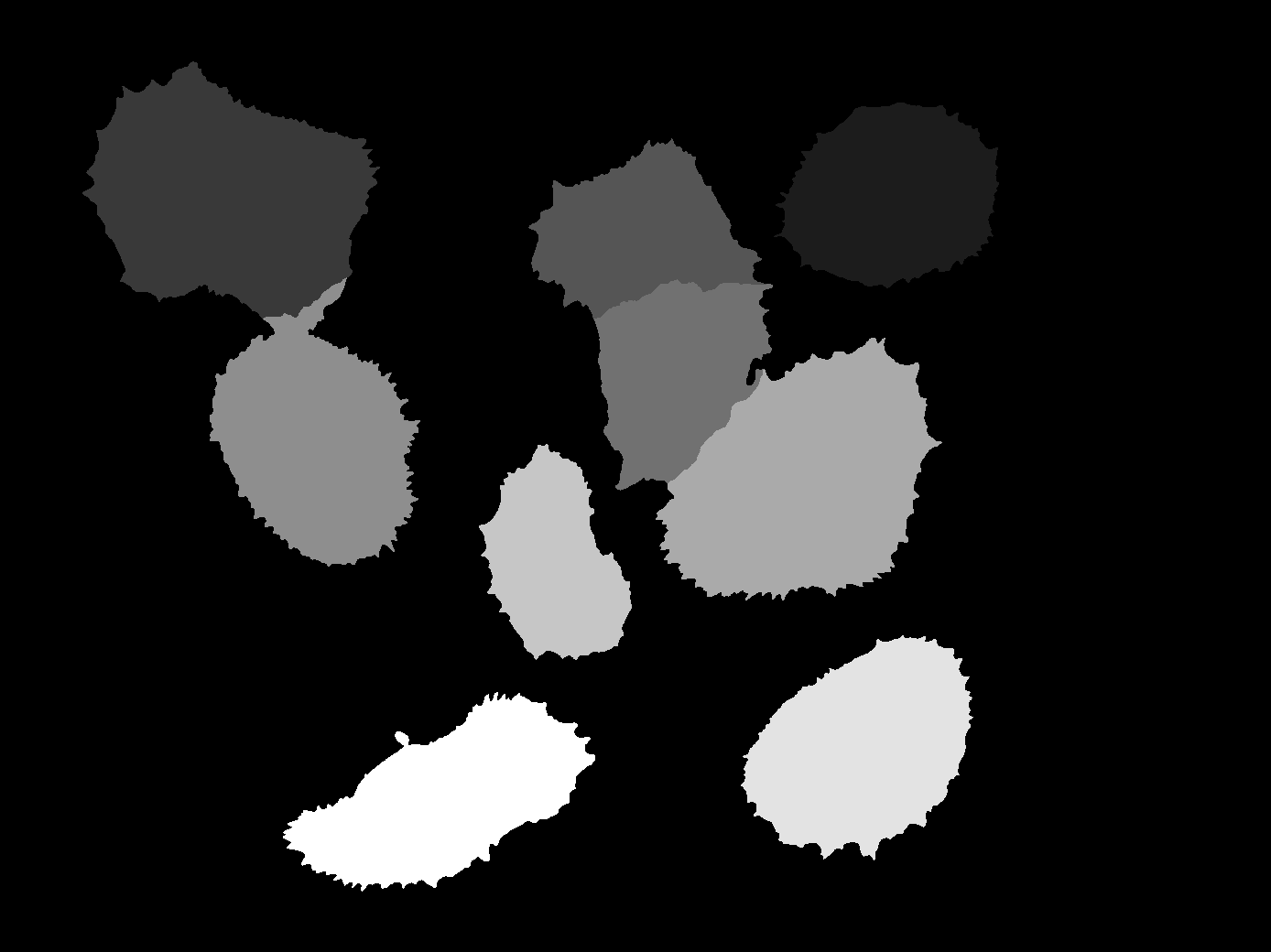

Supplement: S1 File — This file contains all scripts (CellProfiler v2.1.1 and MATLAB2016a) and data necessary to reproduce the information shown in Fig 3. (ZIP) [file pone.0180810.s001.zip › vitaminD_eColi_reproducibleResearchArchive/Results2016/B_28_c2_seg.tif]

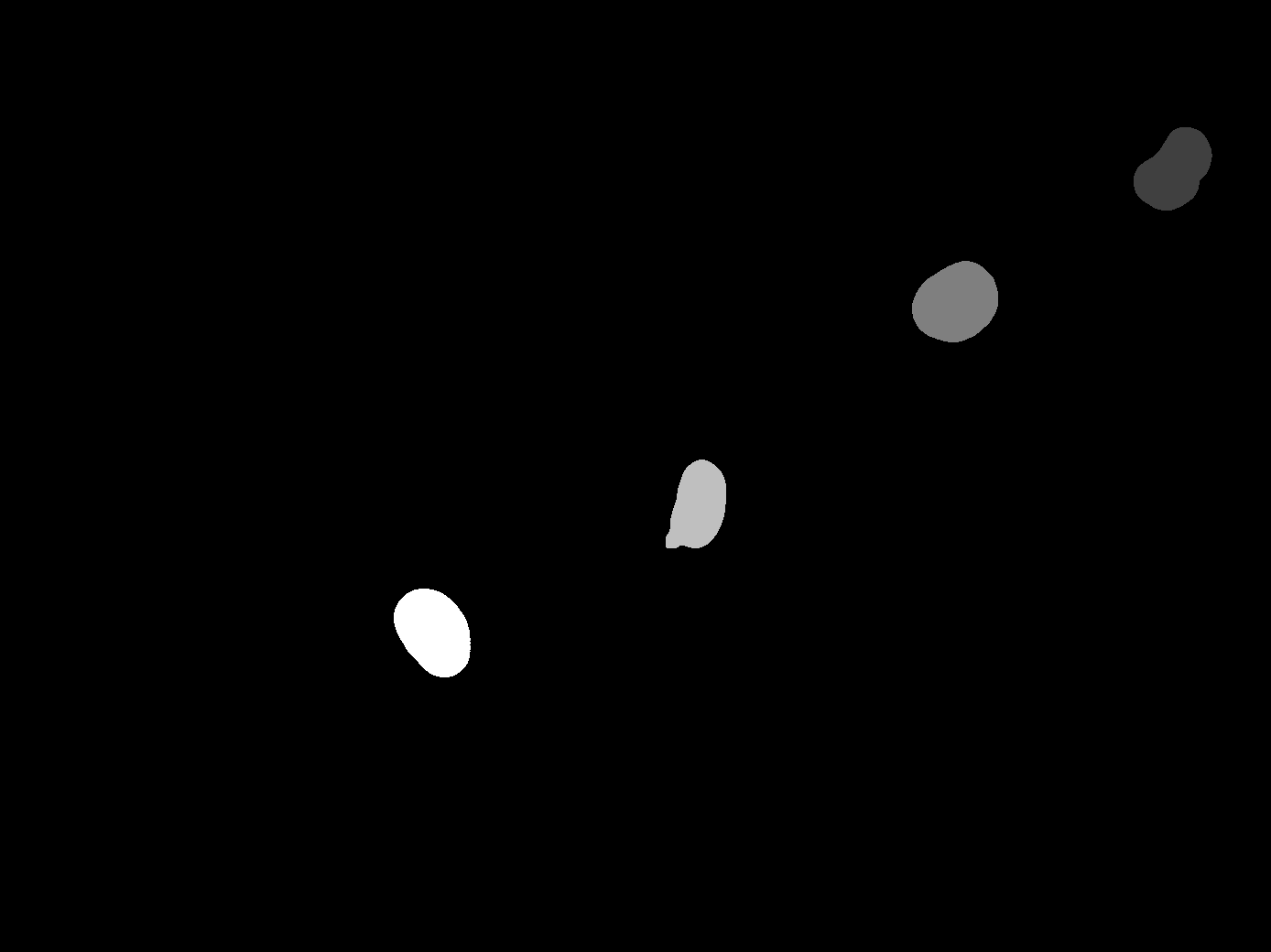

Supplement: S1 File — This file contains all scripts (CellProfiler v2.1.1 and MATLAB2016a) and data necessary to reproduce the information shown in Fig 3. (ZIP) [file pone.0180810.s001.zip › vitaminD_eColi_reproducibleResearchArchive/Results2016/B_29_c0_seg.tif]

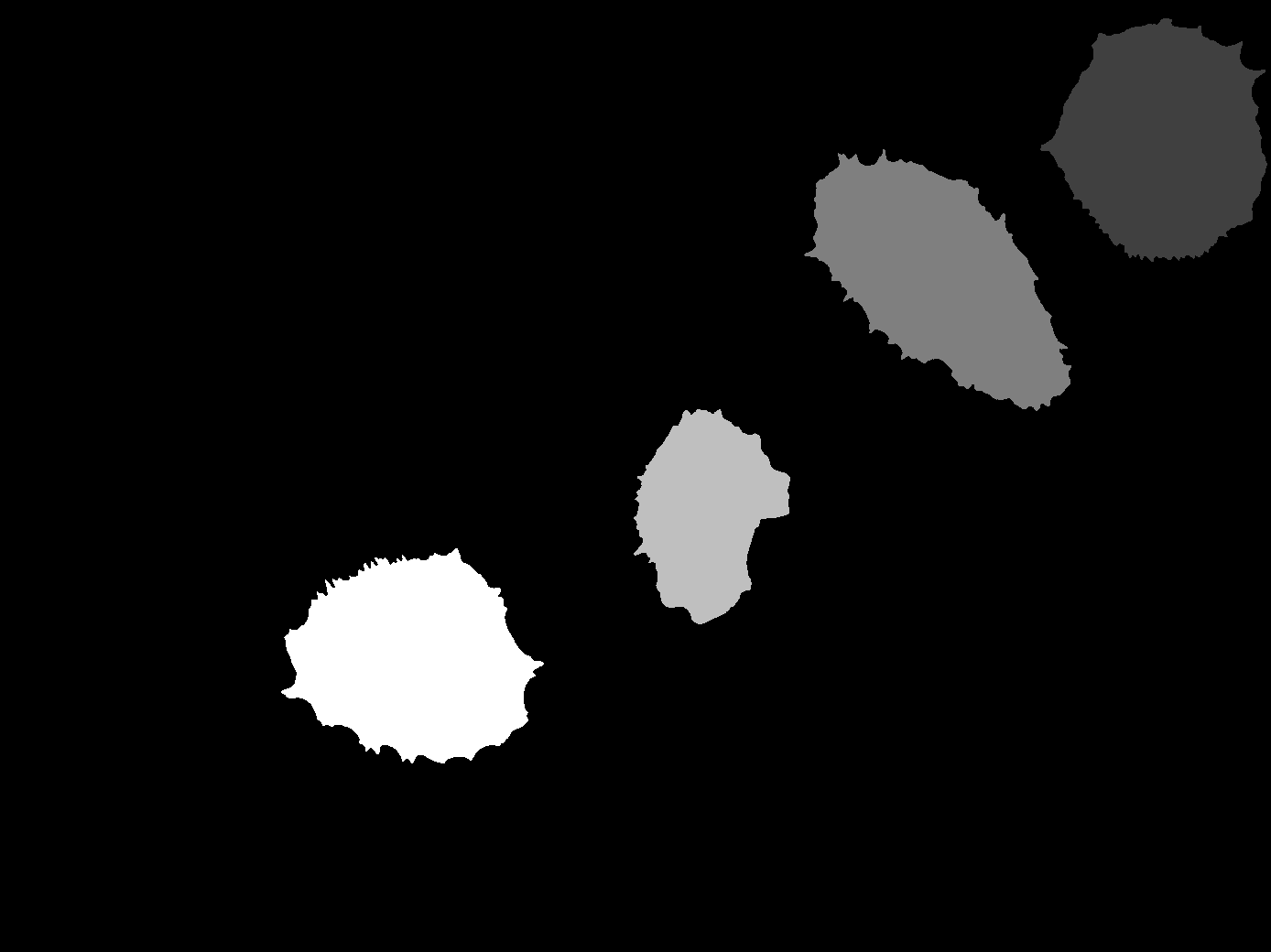

Supplement: S1 File — This file contains all scripts (CellProfiler v2.1.1 and MATLAB2016a) and data necessary to reproduce the information shown in Fig 3. (ZIP) [file pone.0180810.s001.zip › vitaminD_eColi_reproducibleResearchArchive/Results2016/B_29_c2_seg.tif]

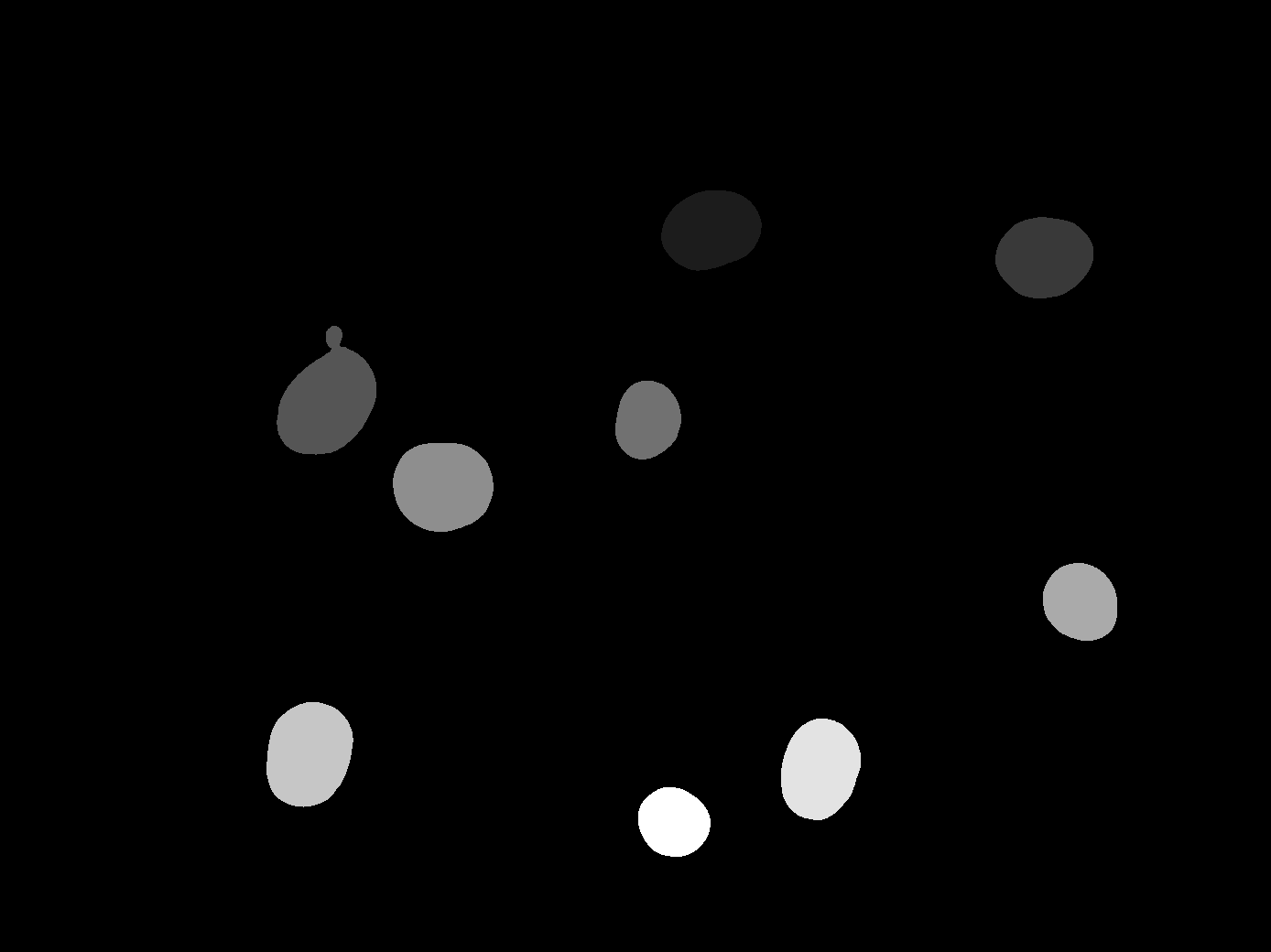

Supplement: S1 File — This file contains all scripts (CellProfiler v2.1.1 and MATLAB2016a) and data necessary to reproduce the information shown in Fig 3. (ZIP) [file pone.0180810.s001.zip › vitaminD_eColi_reproducibleResearchArchive/Results2016/B_2_c0_seg.tif]

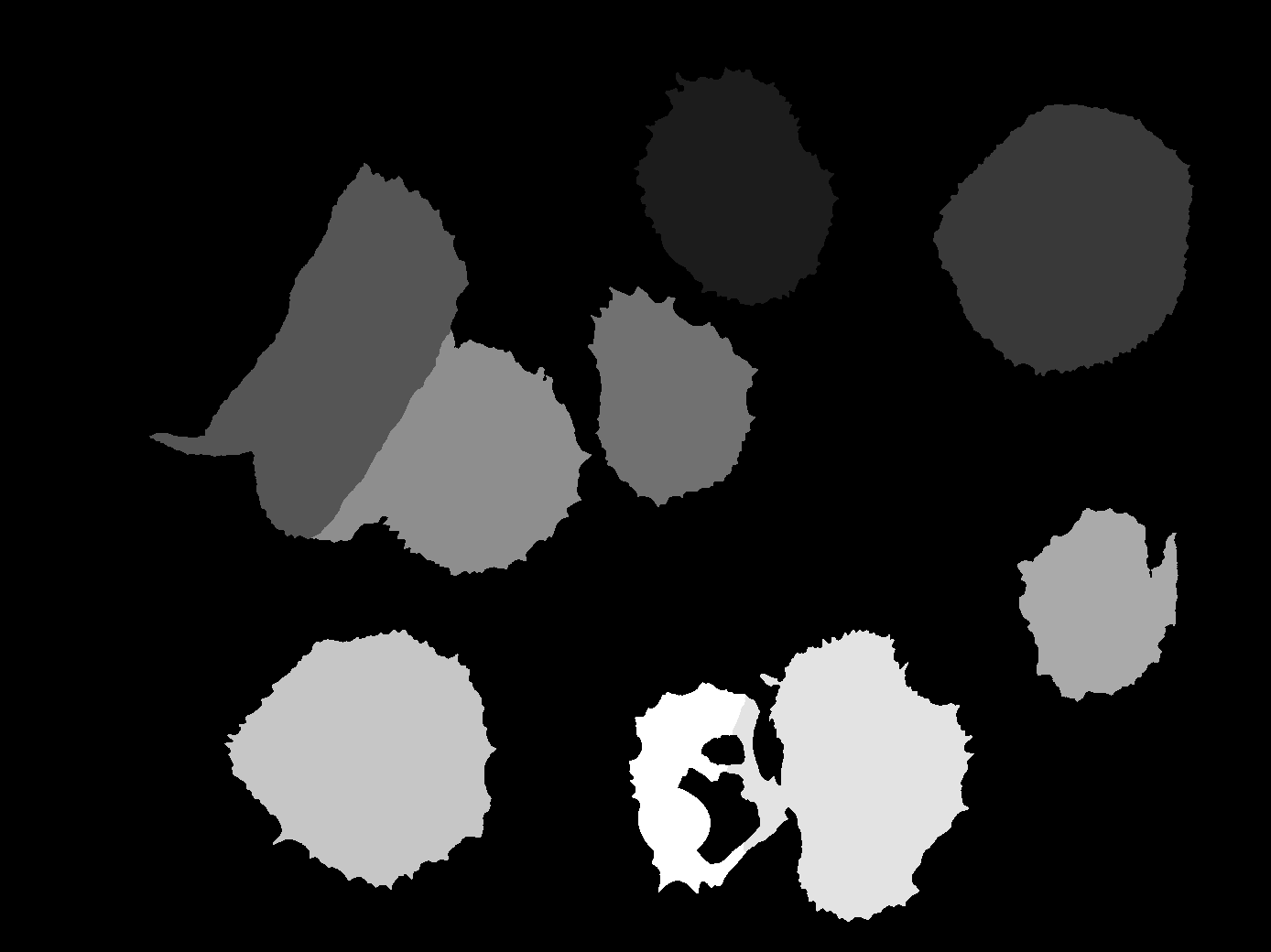

Supplement: S1 File — This file contains all scripts (CellProfiler v2.1.1 and MATLAB2016a) and data necessary to reproduce the information shown in Fig 3. (ZIP) [file pone.0180810.s001.zip › vitaminD_eColi_reproducibleResearchArchive/Results2016/B_2_c2_seg.tif]

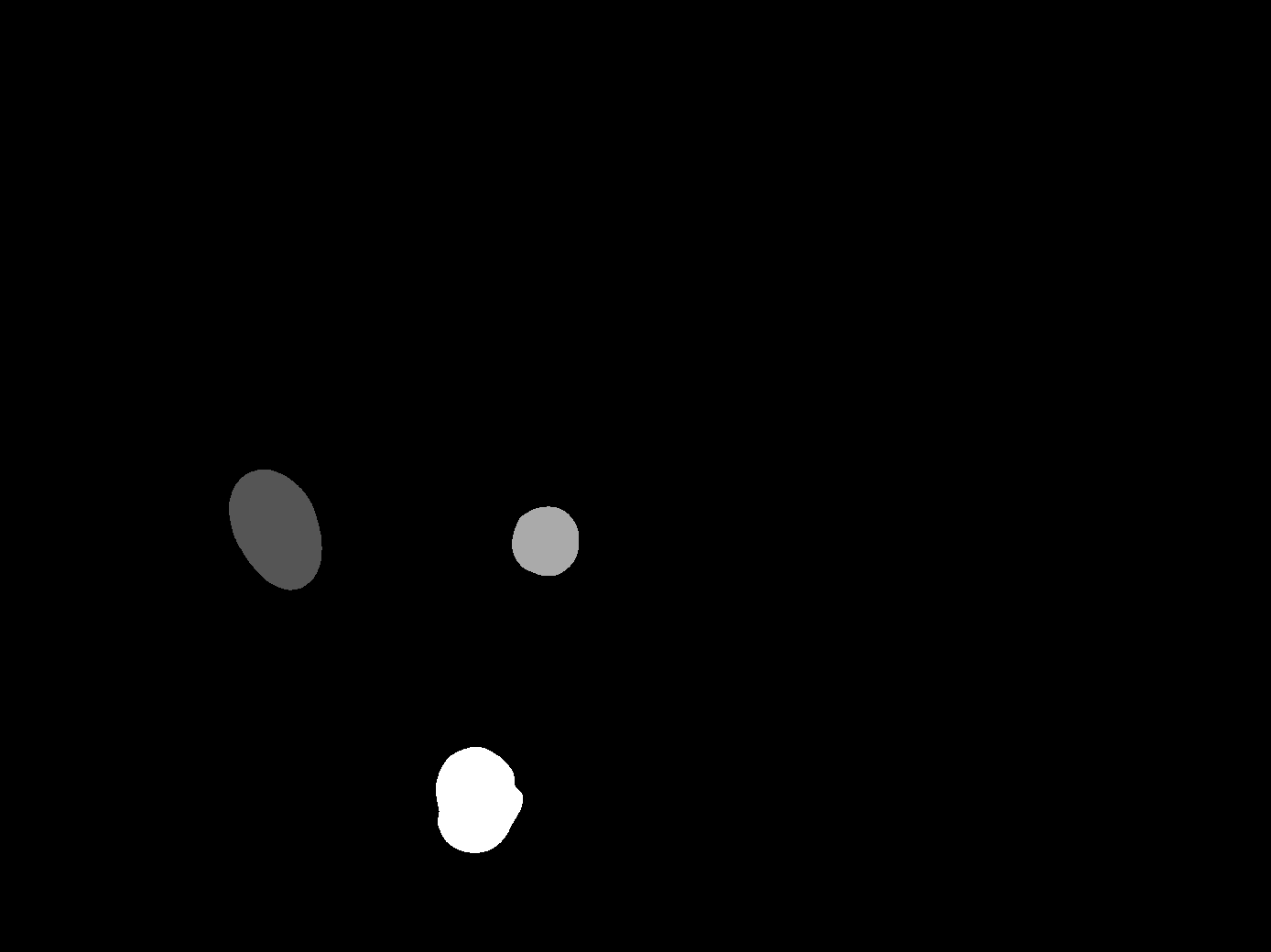

Supplement: S1 File — This file contains all scripts (CellProfiler v2.1.1 and MATLAB2016a) and data necessary to reproduce the information shown in Fig 3. (ZIP) [file pone.0180810.s001.zip › vitaminD_eColi_reproducibleResearchArchive/Results2016/B_30_c0_seg.tif]

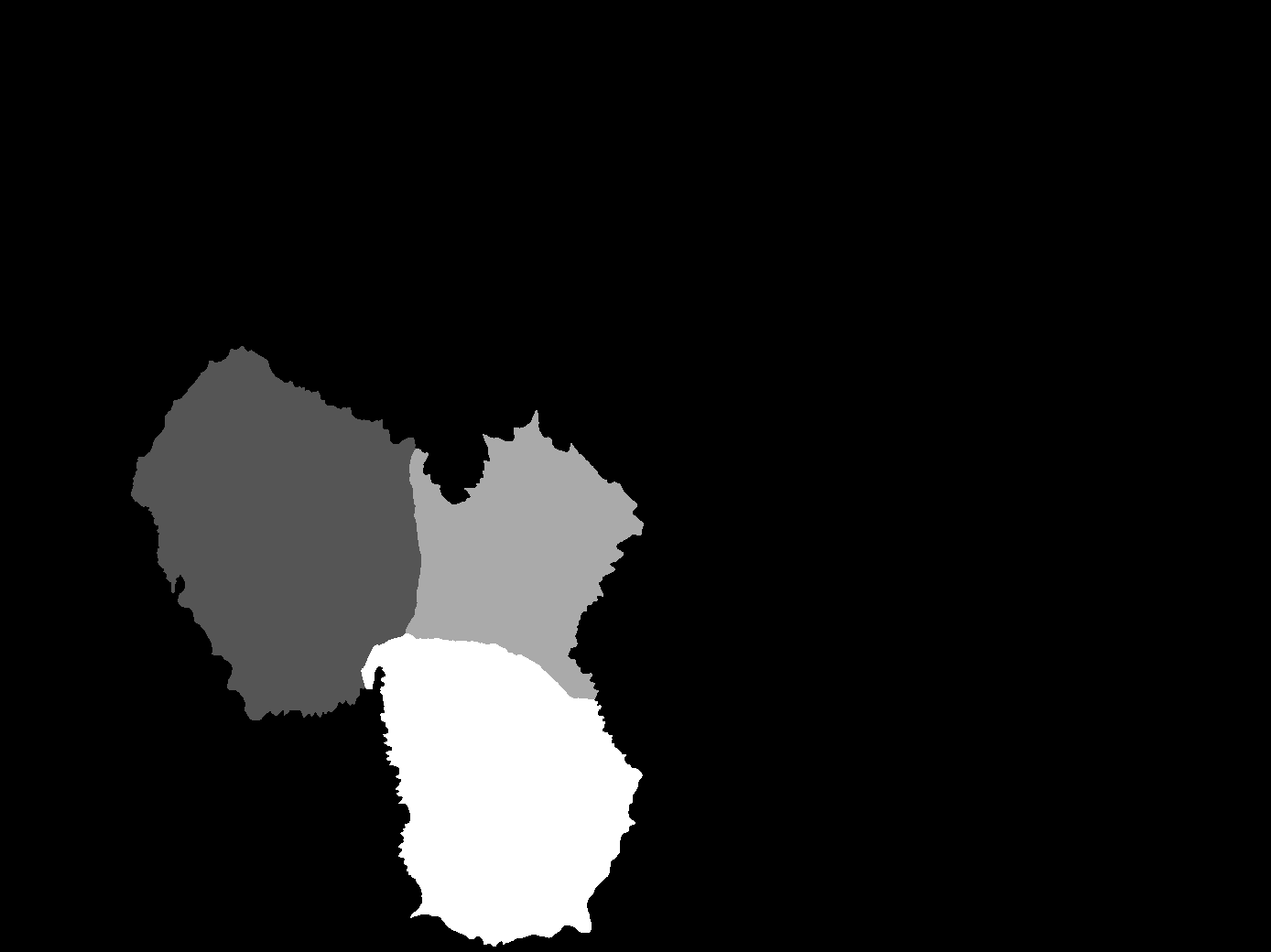

Supplement: S1 File — This file contains all scripts (CellProfiler v2.1.1 and MATLAB2016a) and data necessary to reproduce the information shown in Fig 3. (ZIP) [file pone.0180810.s001.zip › vitaminD_eColi_reproducibleResearchArchive/Results2016/B_30_c2_seg.tif]

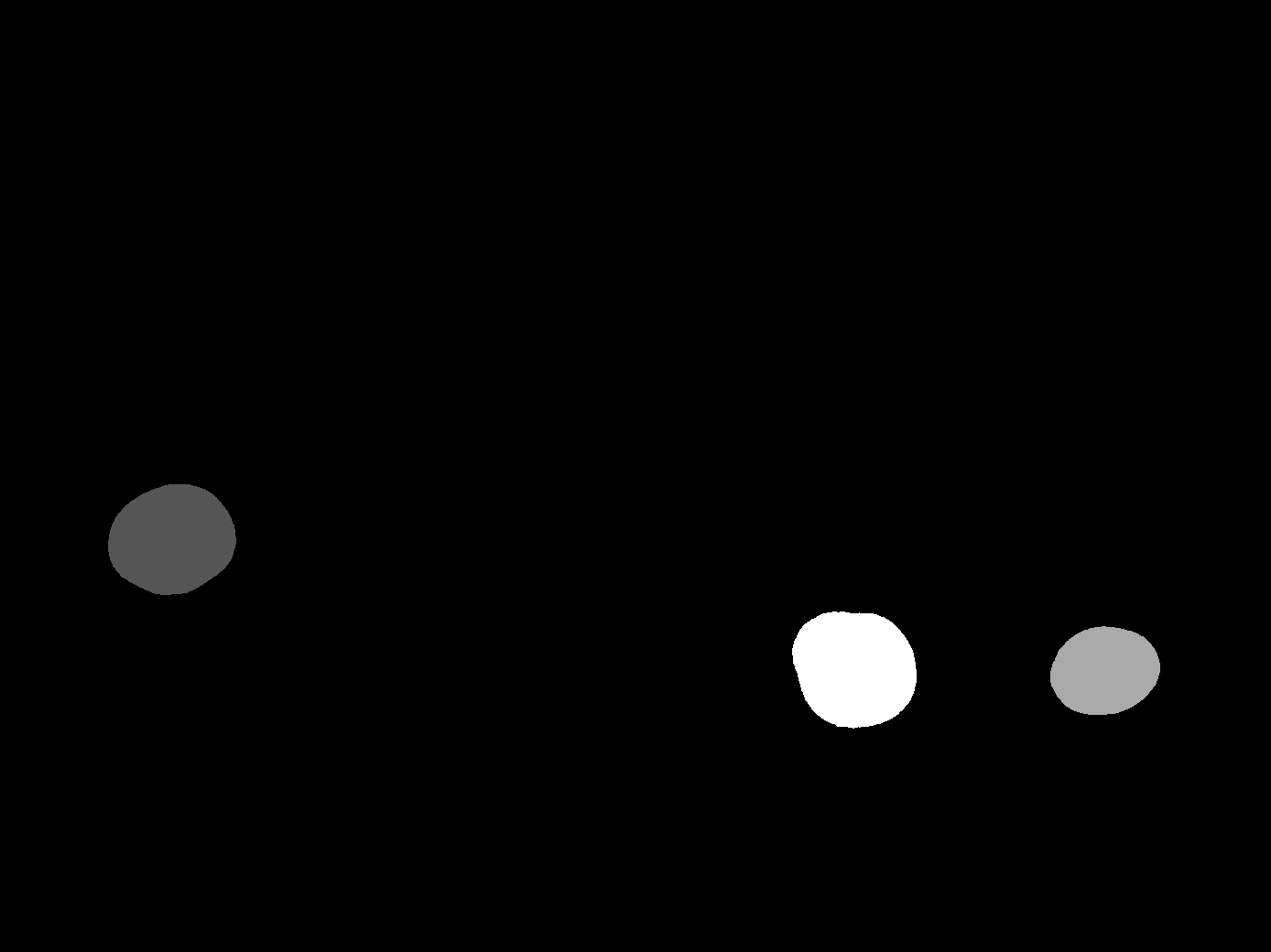

Supplement: S1 File — This file contains all scripts (CellProfiler v2.1.1 and MATLAB2016a) and data necessary to reproduce the information shown in Fig 3. (ZIP) [file pone.0180810.s001.zip › vitaminD_eColi_reproducibleResearchArchive/Results2016/B_31_c0_seg.tif]

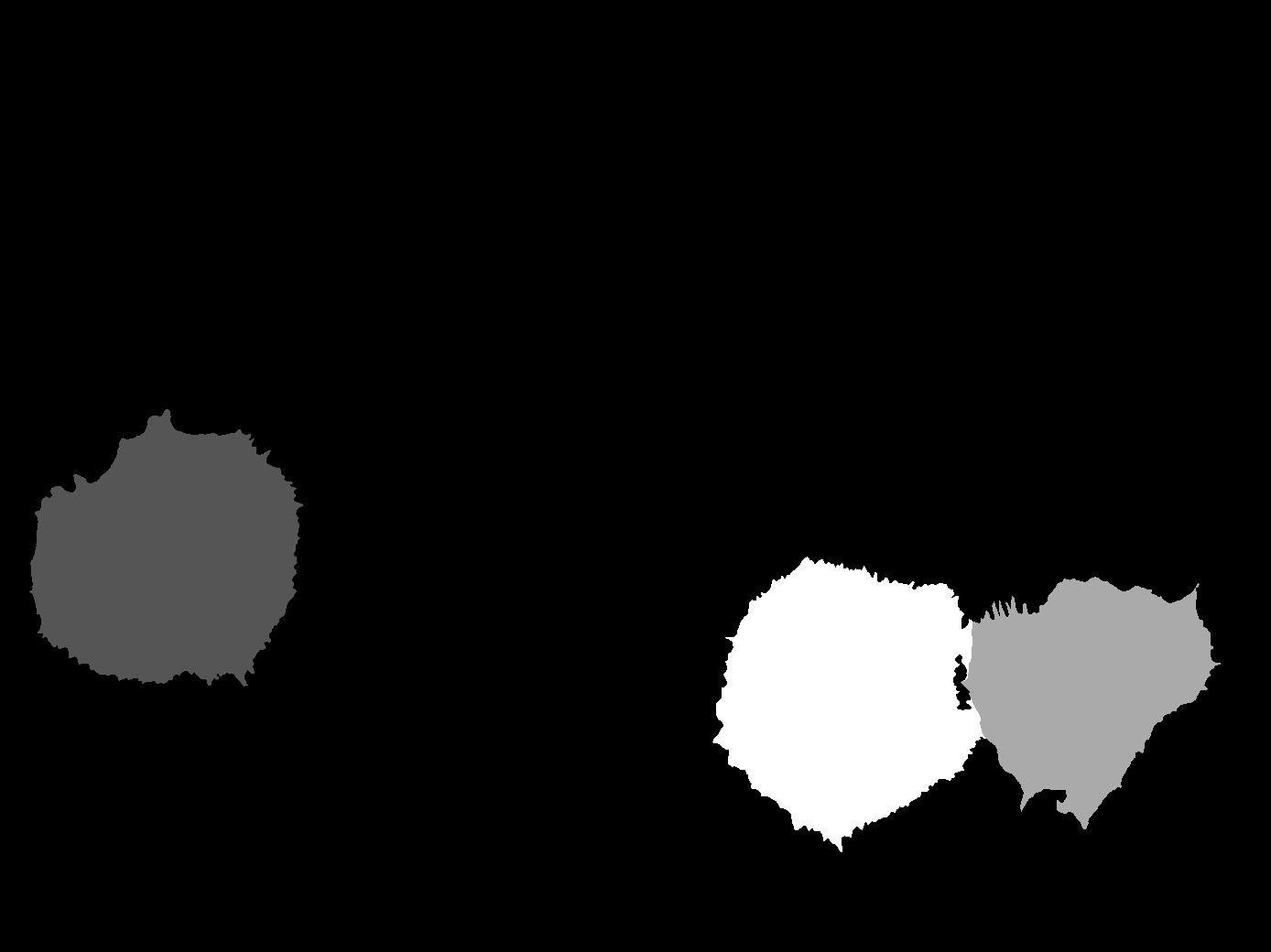

Supplement: S1 File — This file contains all scripts (CellProfiler v2.1.1 and MATLAB2016a) and data necessary to reproduce the information shown in Fig 3. (ZIP) [file pone.0180810.s001.zip › vitaminD_eColi_reproducibleResearchArchive/Results2016/B_31_c2_seg.tif]

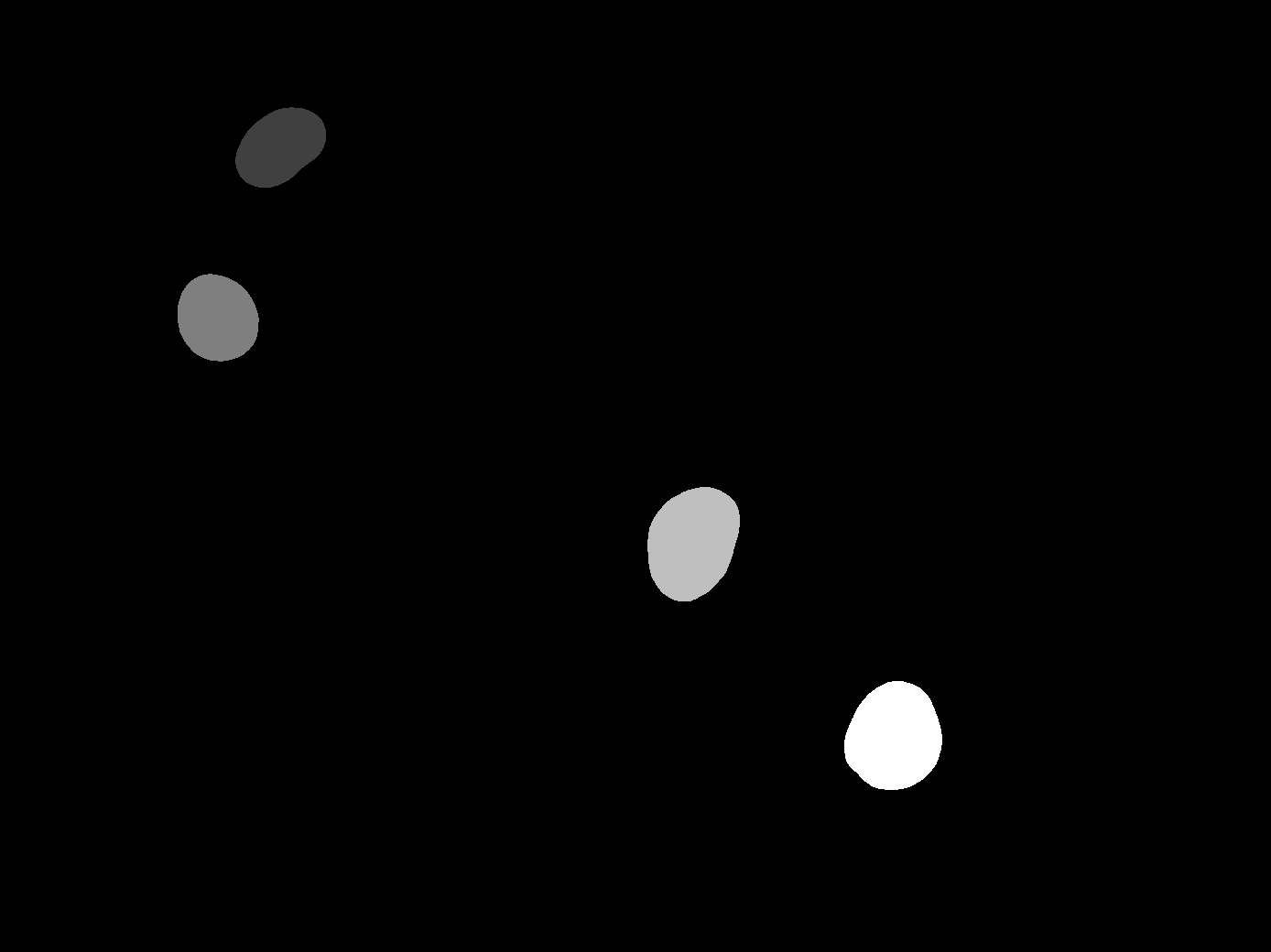

Supplement: S1 File — This file contains all scripts (CellProfiler v2.1.1 and MATLAB2016a) and data necessary to reproduce the information shown in Fig 3. (ZIP) [file pone.0180810.s001.zip › vitaminD_eColi_reproducibleResearchArchive/Results2016/B_32_c0_seg.tif]

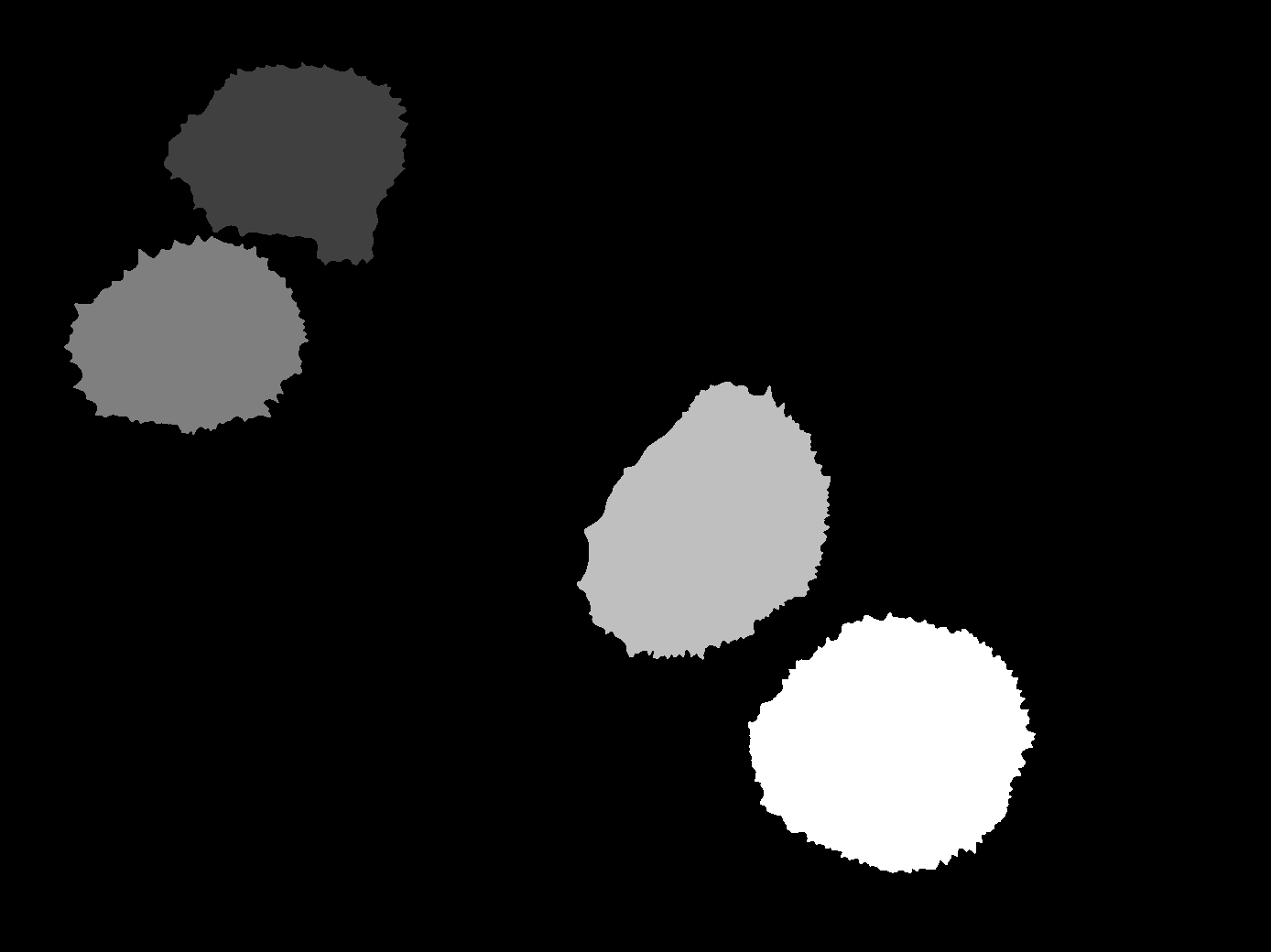

Supplement: S1 File — This file contains all scripts (CellProfiler v2.1.1 and MATLAB2016a) and data necessary to reproduce the information shown in Fig 3. (ZIP) [file pone.0180810.s001.zip › vitaminD_eColi_reproducibleResearchArchive/Results2016/B_32_c2_seg.tif]

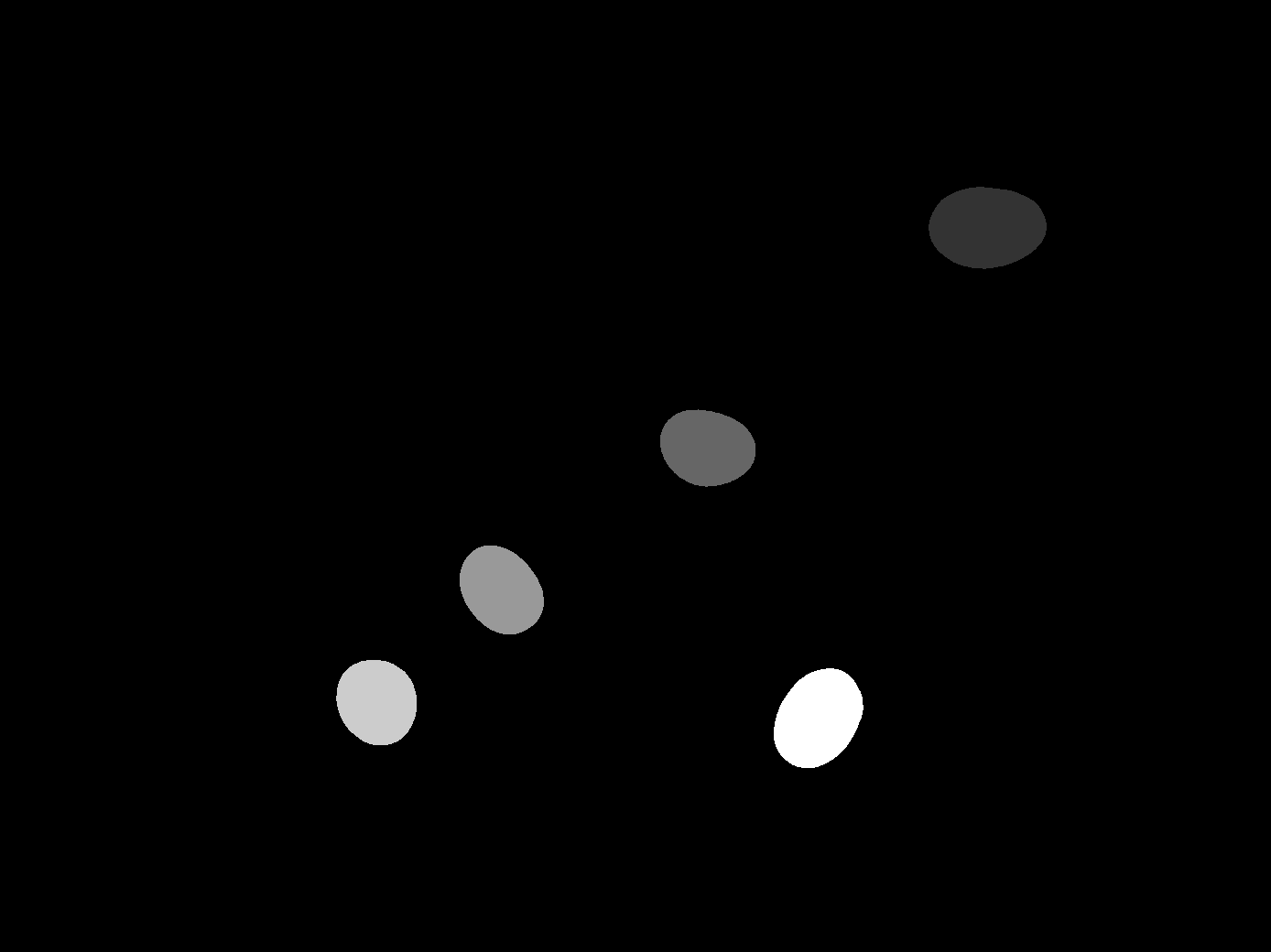

Supplement: S1 File — This file contains all scripts (CellProfiler v2.1.1 and MATLAB2016a) and data necessary to reproduce the information shown in Fig 3. (ZIP) [file pone.0180810.s001.zip › vitaminD_eColi_reproducibleResearchArchive/Results2016/B_33_c0_seg.tif]

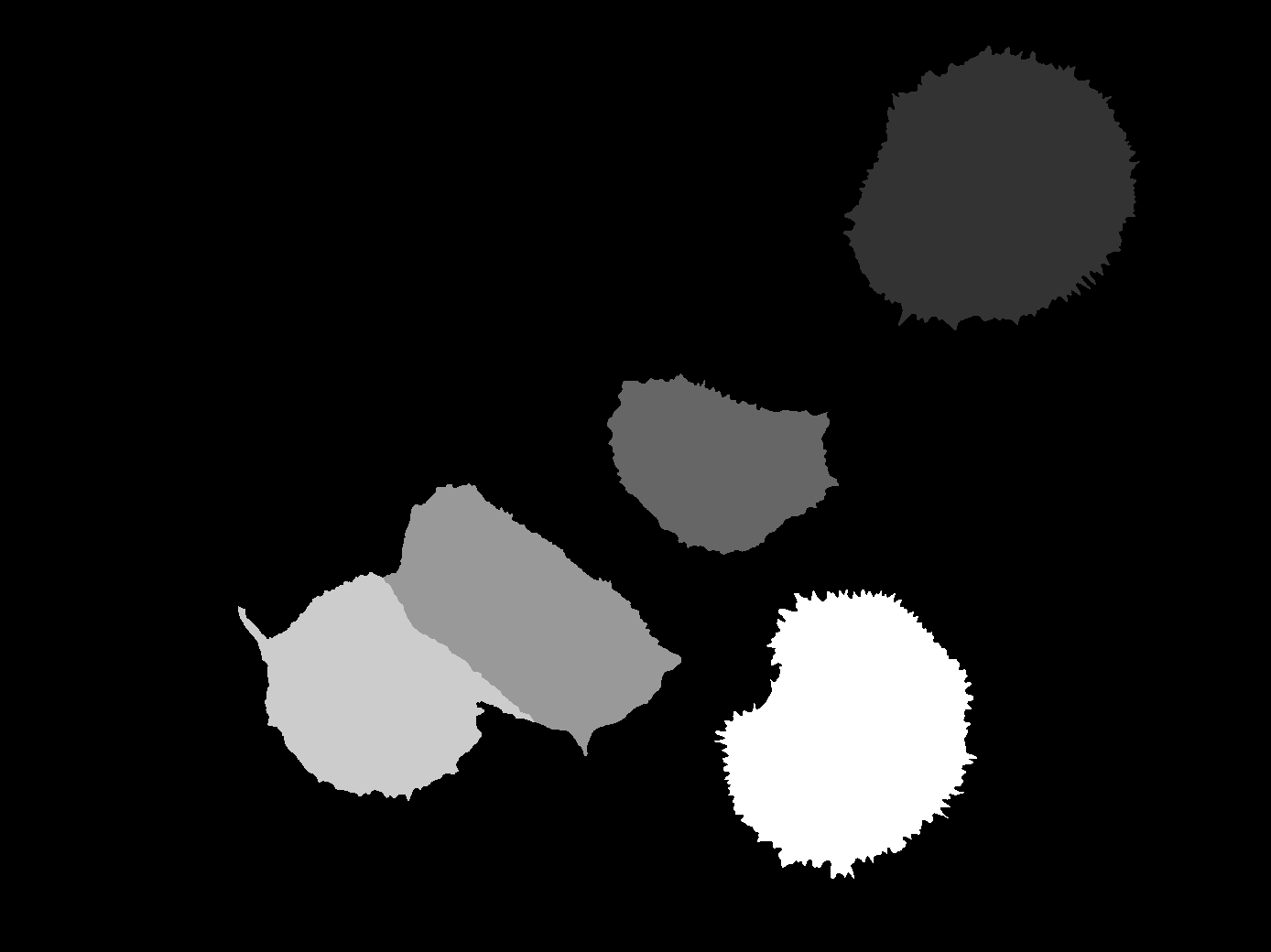

Supplement: S1 File — This file contains all scripts (CellProfiler v2.1.1 and MATLAB2016a) and data necessary to reproduce the information shown in Fig 3. (ZIP) [file pone.0180810.s001.zip › vitaminD_eColi_reproducibleResearchArchive/Results2016/B_33_c2_seg.tif]

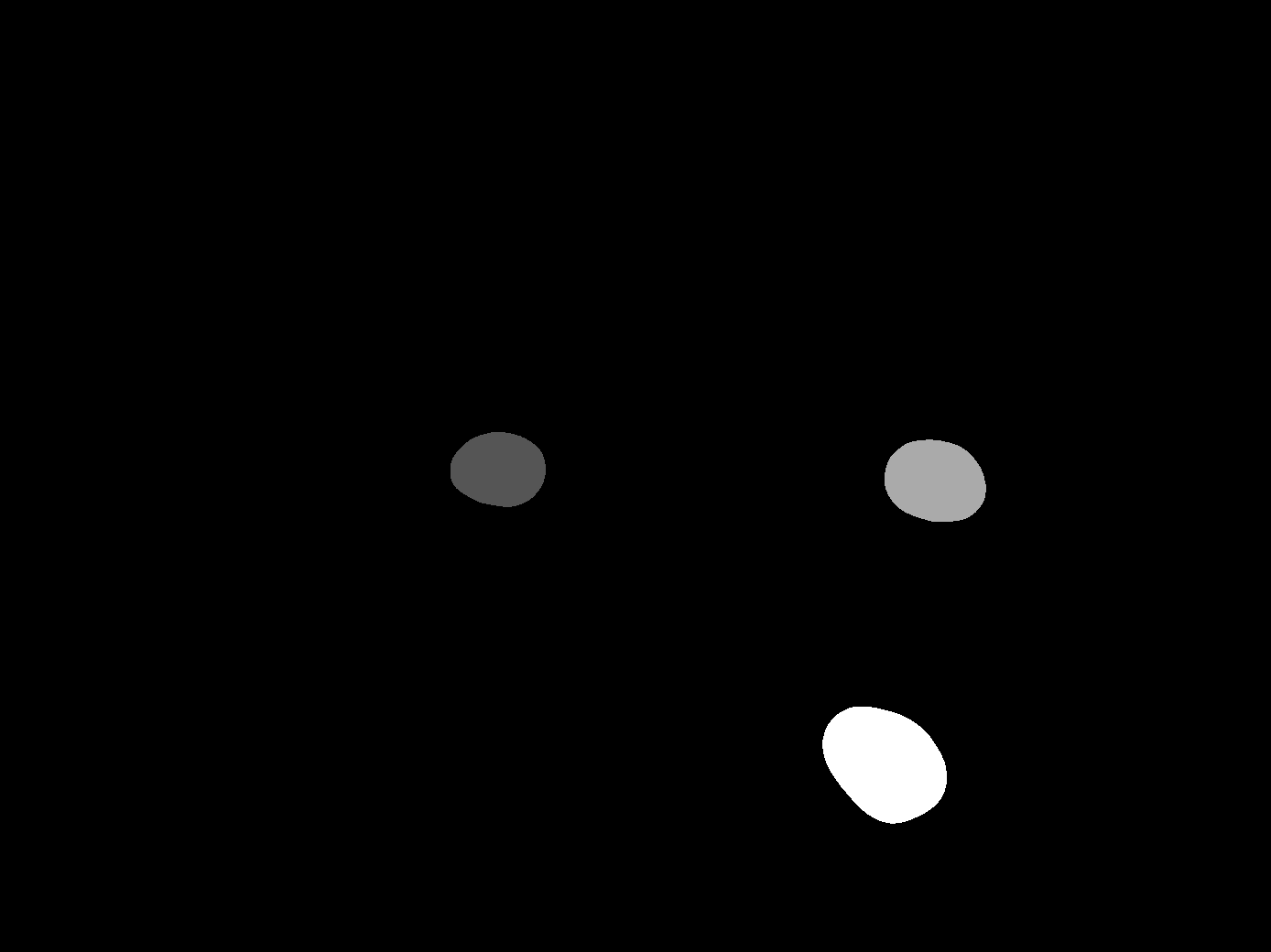

Supplement: S1 File — This file contains all scripts (CellProfiler v2.1.1 and MATLAB2016a) and data necessary to reproduce the information shown in Fig 3. (ZIP) [file pone.0180810.s001.zip › vitaminD_eColi_reproducibleResearchArchive/Results2016/B_34_c0_seg.tif]

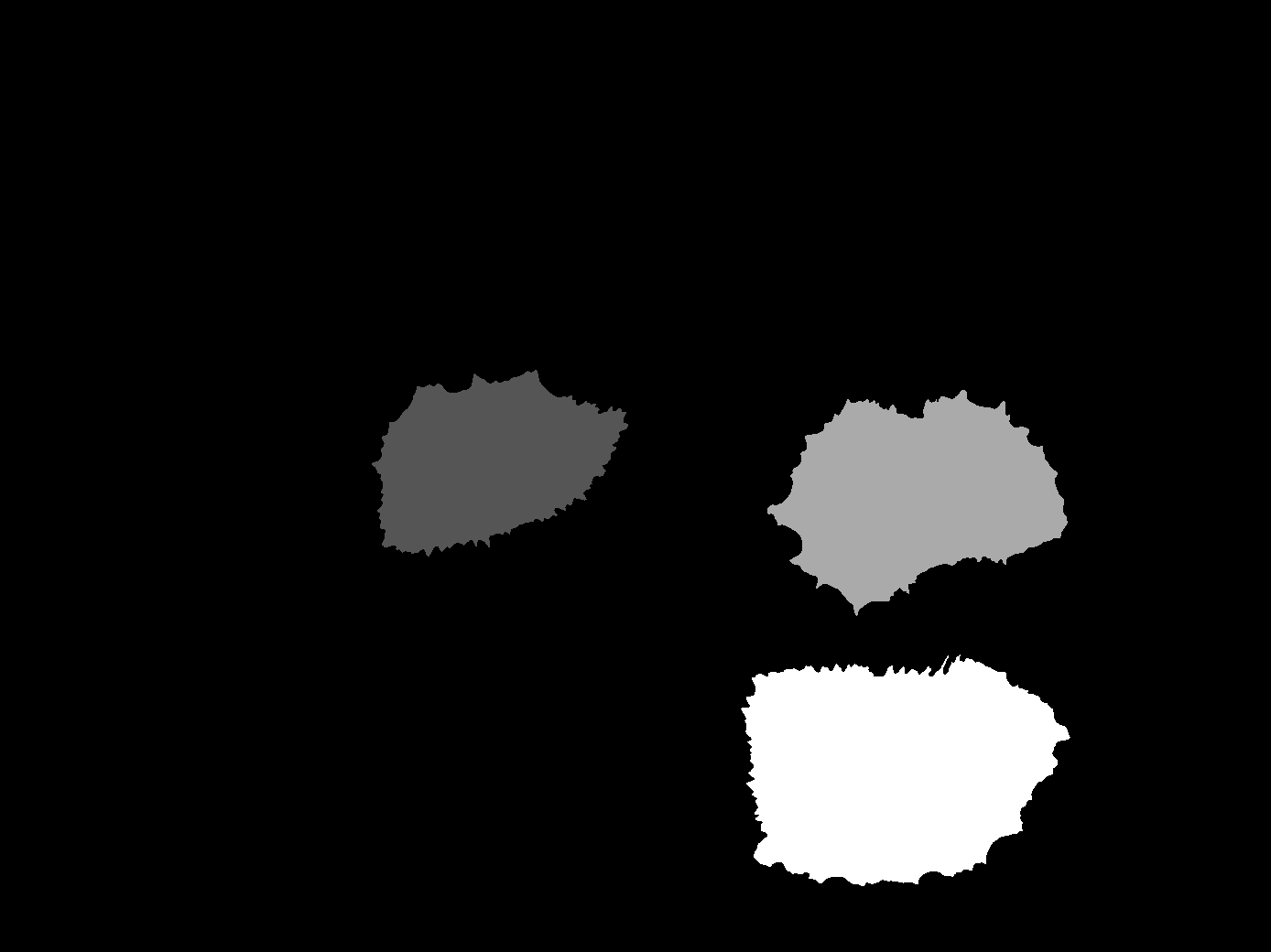

Supplement: S1 File — This file contains all scripts (CellProfiler v2.1.1 and MATLAB2016a) and data necessary to reproduce the information shown in Fig 3. (ZIP) [file pone.0180810.s001.zip › vitaminD_eColi_reproducibleResearchArchive/Results2016/B_34_c2_seg.tif]

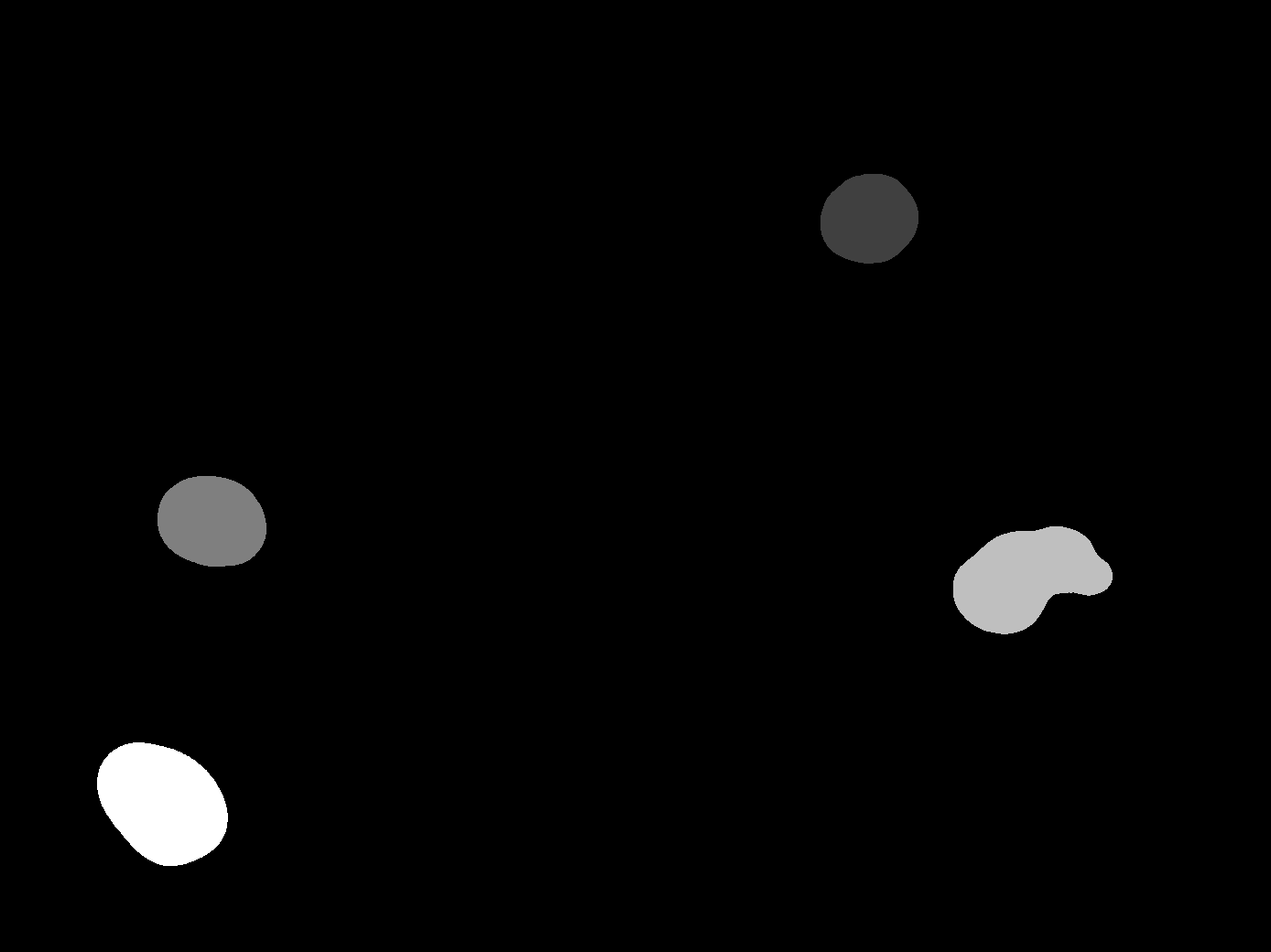

Supplement: S1 File — This file contains all scripts (CellProfiler v2.1.1 and MATLAB2016a) and data necessary to reproduce the information shown in Fig 3. (ZIP) [file pone.0180810.s001.zip › vitaminD_eColi_reproducibleResearchArchive/Results2016/B_35_c0_seg.tif]

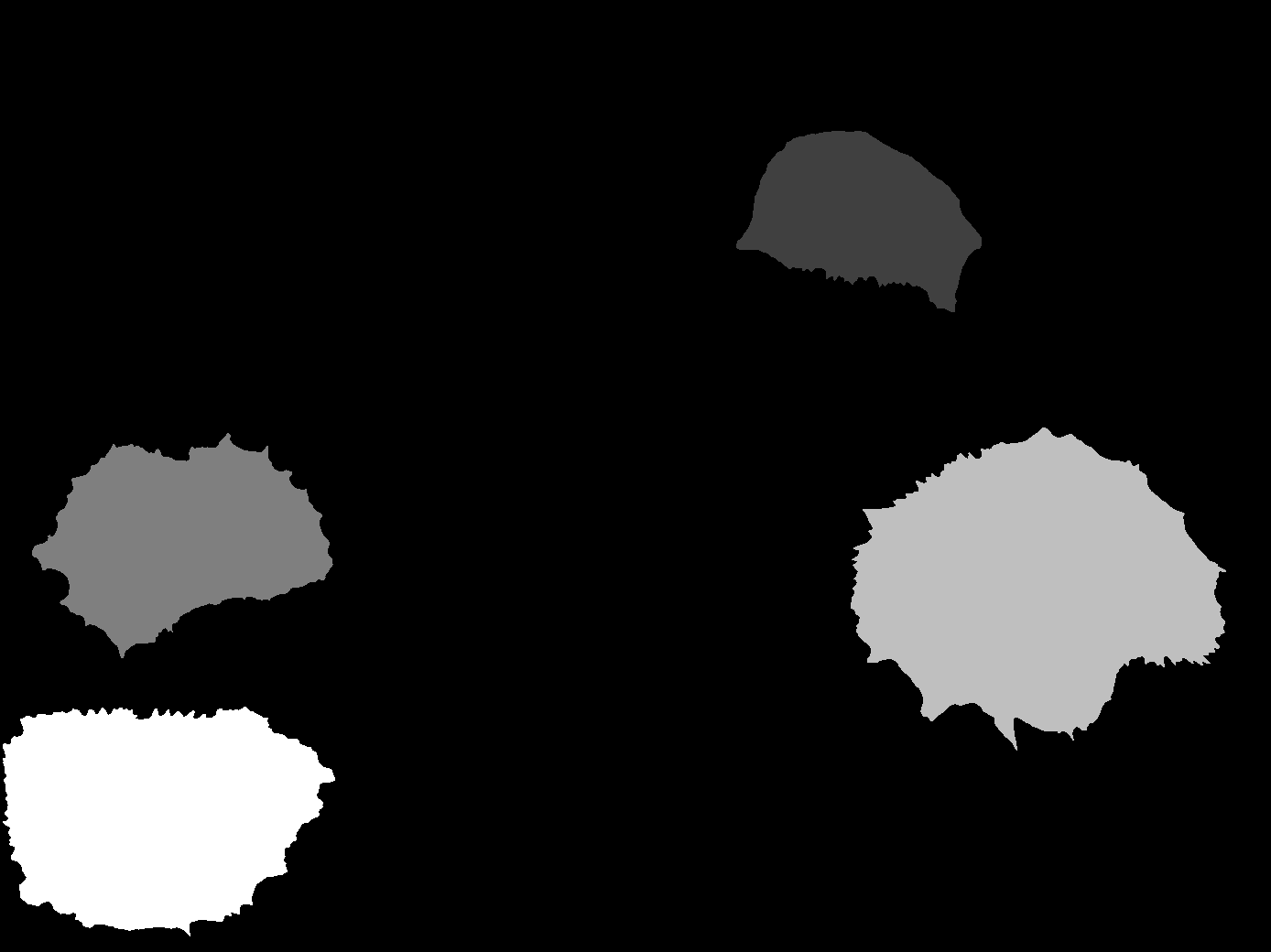

Supplement: S1 File — This file contains all scripts (CellProfiler v2.1.1 and MATLAB2016a) and data necessary to reproduce the information shown in Fig 3. (ZIP) [file pone.0180810.s001.zip › vitaminD_eColi_reproducibleResearchArchive/Results2016/B_35_c2_seg.tif]

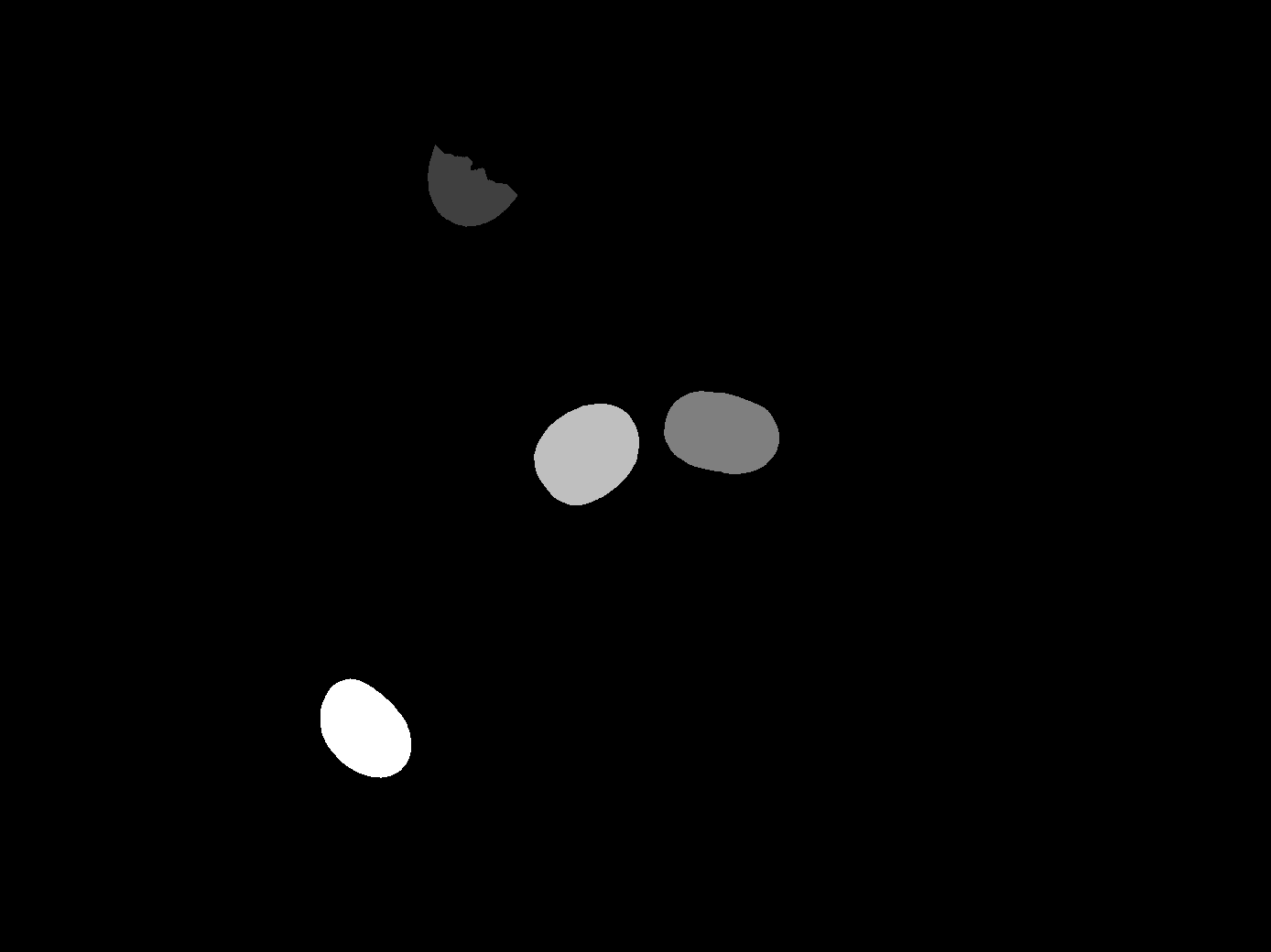

Supplement: S1 File — This file contains all scripts (CellProfiler v2.1.1 and MATLAB2016a) and data necessary to reproduce the information shown in Fig 3. (ZIP) [file pone.0180810.s001.zip › vitaminD_eColi_reproducibleResearchArchive/Results2016/B_36_c0_seg.tif]

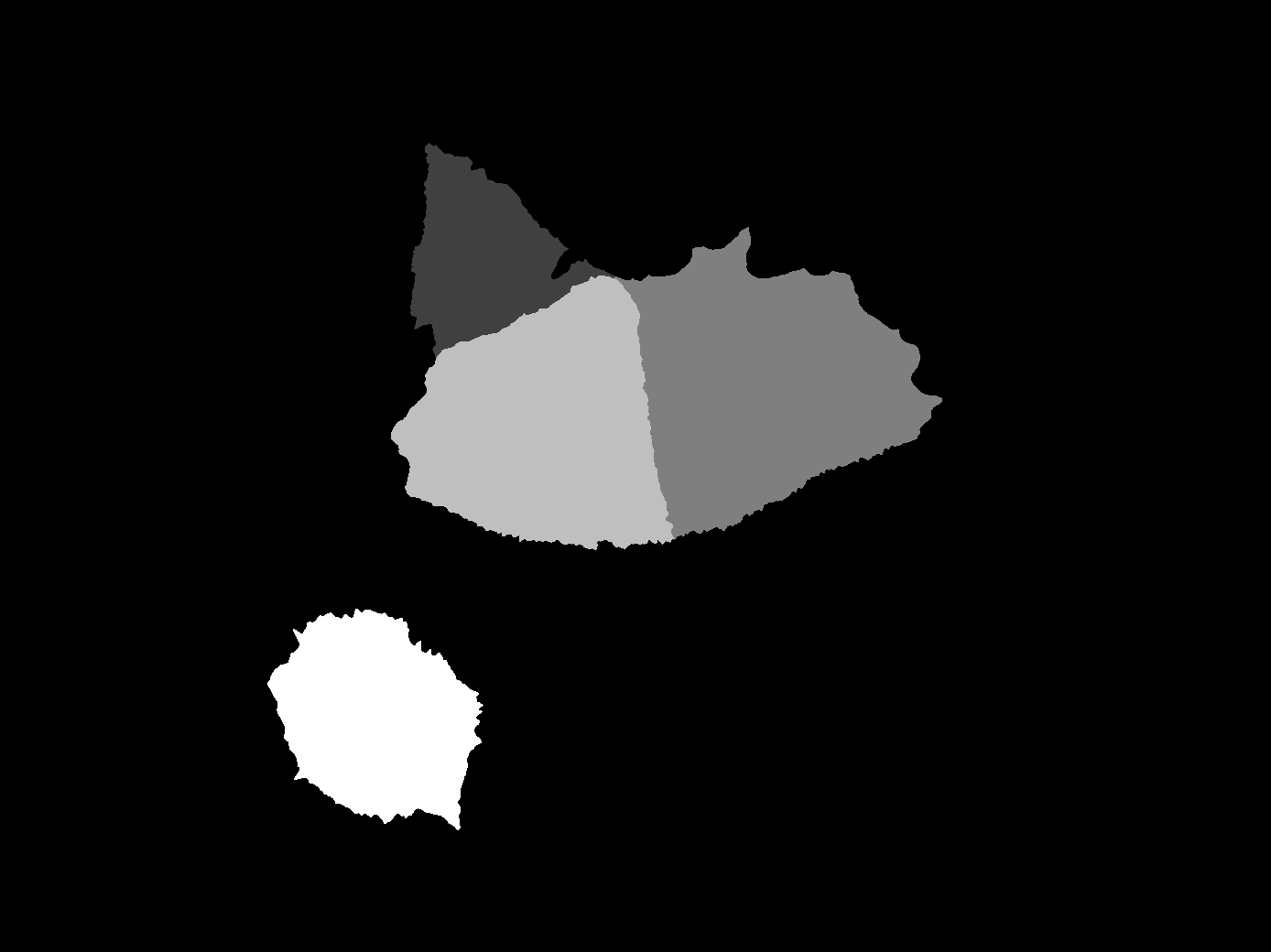

Supplement: S1 File — This file contains all scripts (CellProfiler v2.1.1 and MATLAB2016a) and data necessary to reproduce the information shown in Fig 3. (ZIP) [file pone.0180810.s001.zip › vitaminD_eColi_reproducibleResearchArchive/Results2016/B_36_c2_seg.tif]

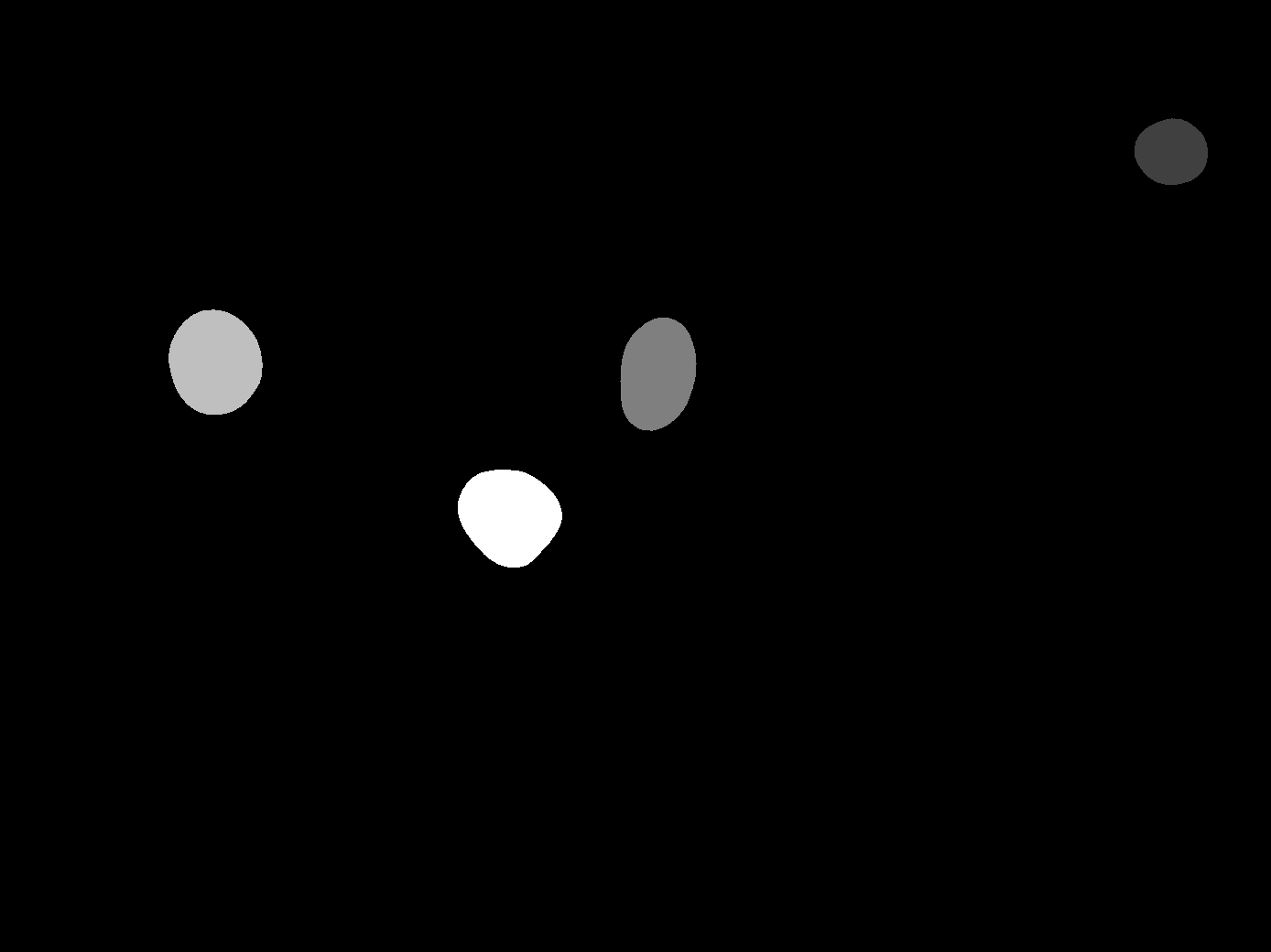

Supplement: S1 File — This file contains all scripts (CellProfiler v2.1.1 and MATLAB2016a) and data necessary to reproduce the information shown in Fig 3. (ZIP) [file pone.0180810.s001.zip › vitaminD_eColi_reproducibleResearchArchive/Results2016/B_37_c0_seg.tif]

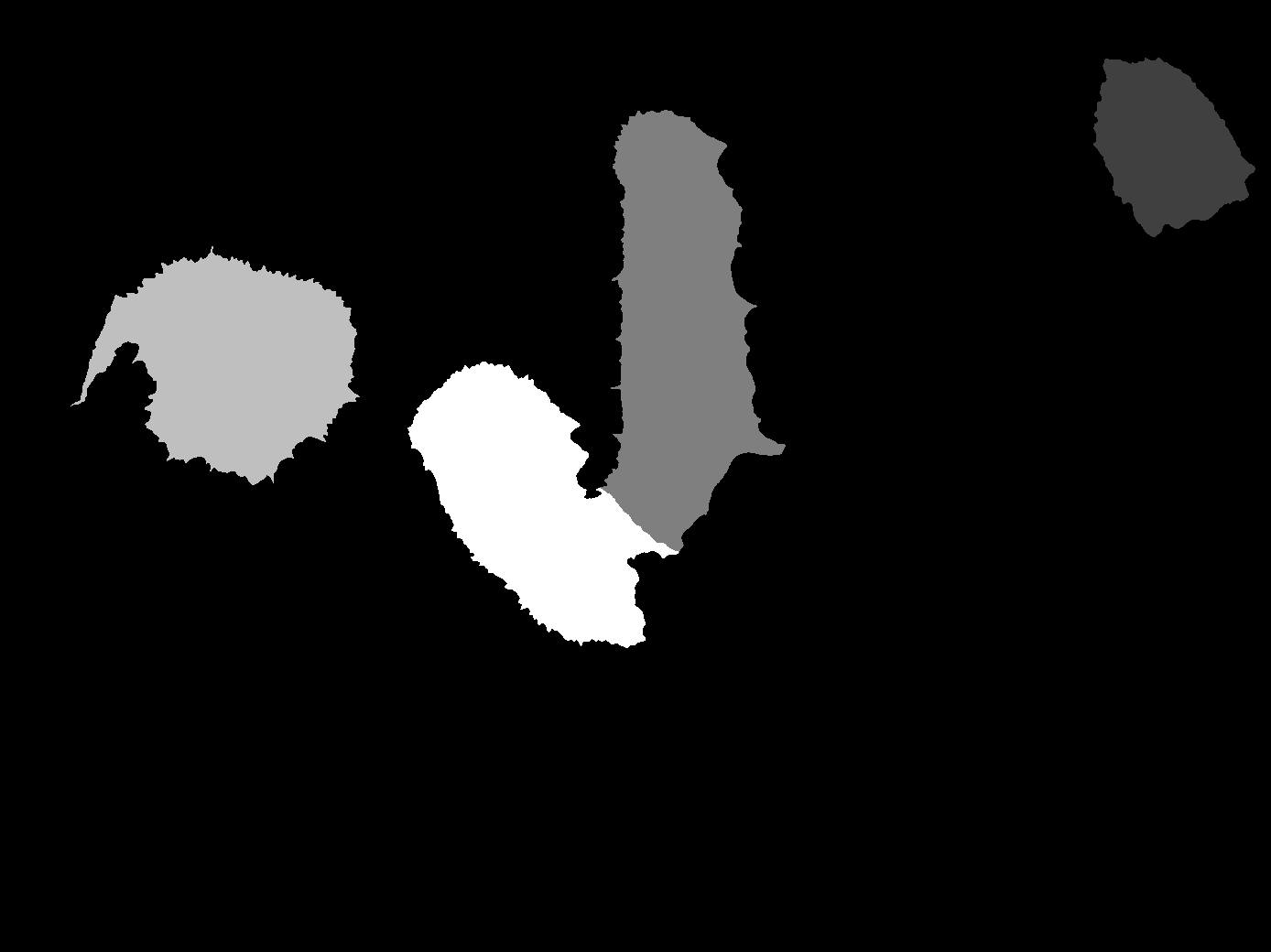

Supplement: S1 File — This file contains all scripts (CellProfiler v2.1.1 and MATLAB2016a) and data necessary to reproduce the information shown in Fig 3. (ZIP) [file pone.0180810.s001.zip › vitaminD_eColi_reproducibleResearchArchive/Results2016/B_37_c2_seg.tif]

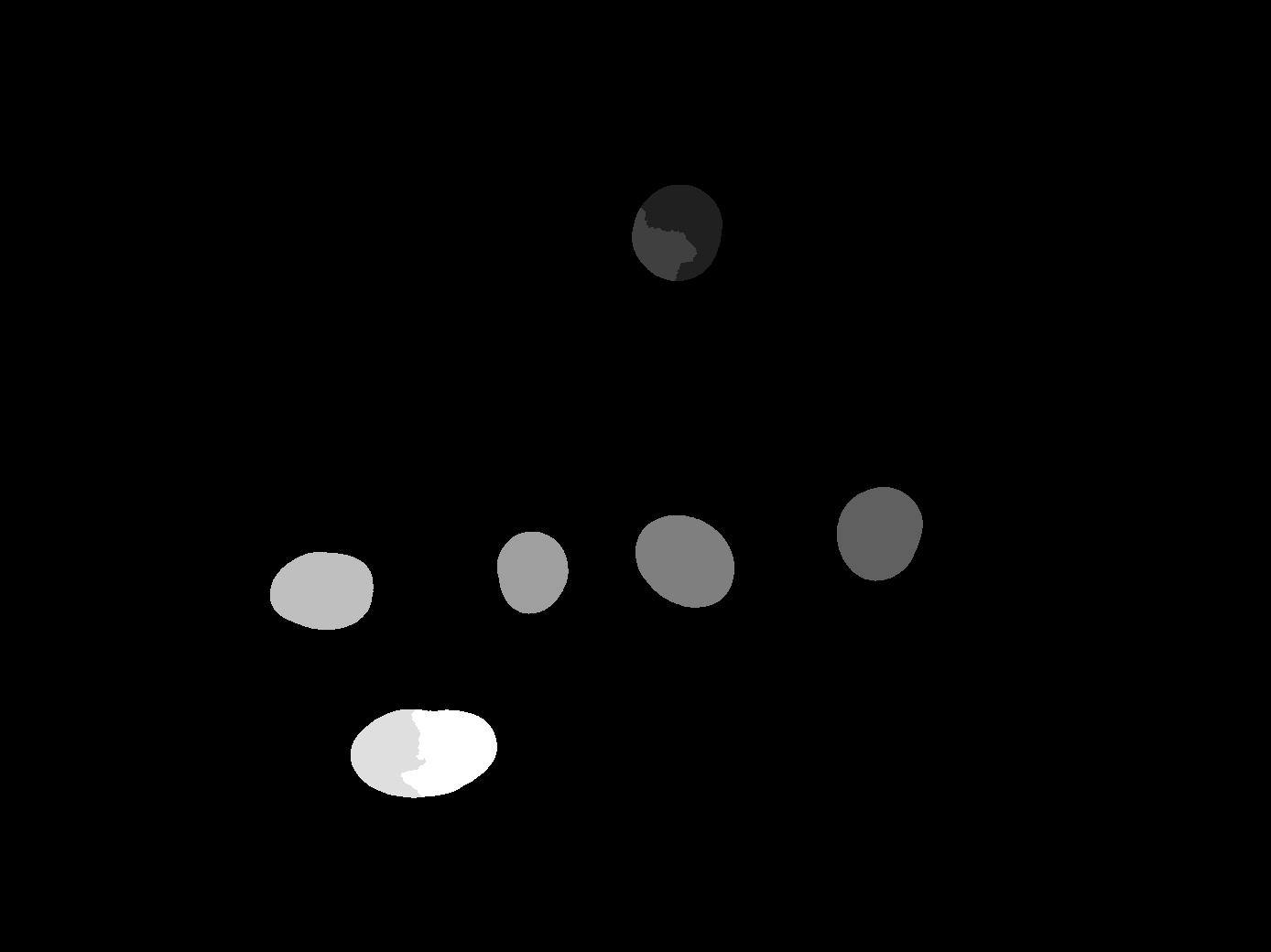

Supplement: S1 File — This file contains all scripts (CellProfiler v2.1.1 and MATLAB2016a) and data necessary to reproduce the information shown in Fig 3. (ZIP) [file pone.0180810.s001.zip › vitaminD_eColi_reproducibleResearchArchive/Results2016/B_38_c0_seg.tif]

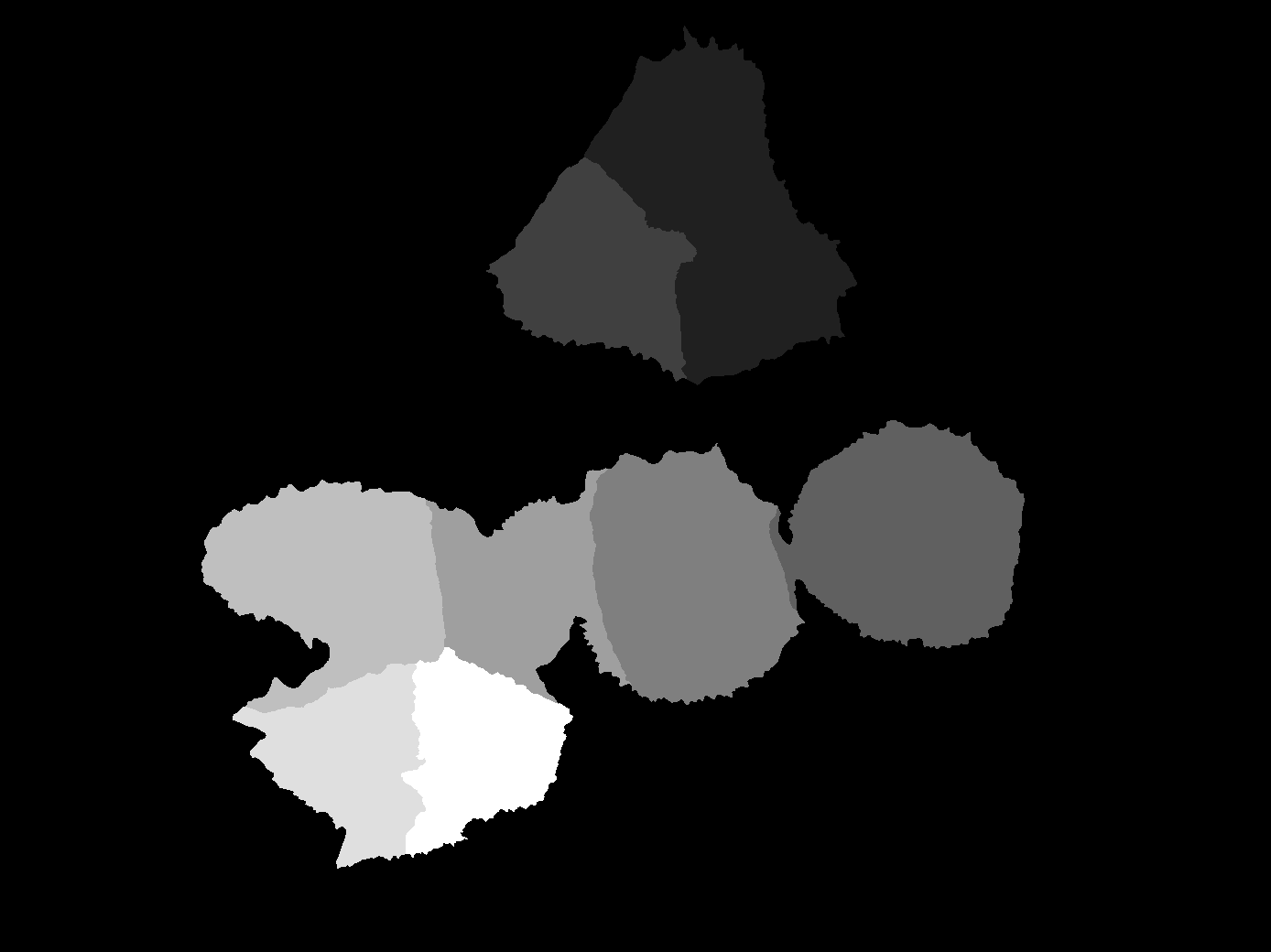

Supplement: S1 File — This file contains all scripts (CellProfiler v2.1.1 and MATLAB2016a) and data necessary to reproduce the information shown in Fig 3. (ZIP) [file pone.0180810.s001.zip › vitaminD_eColi_reproducibleResearchArchive/Results2016/B_38_c2_seg.tif]

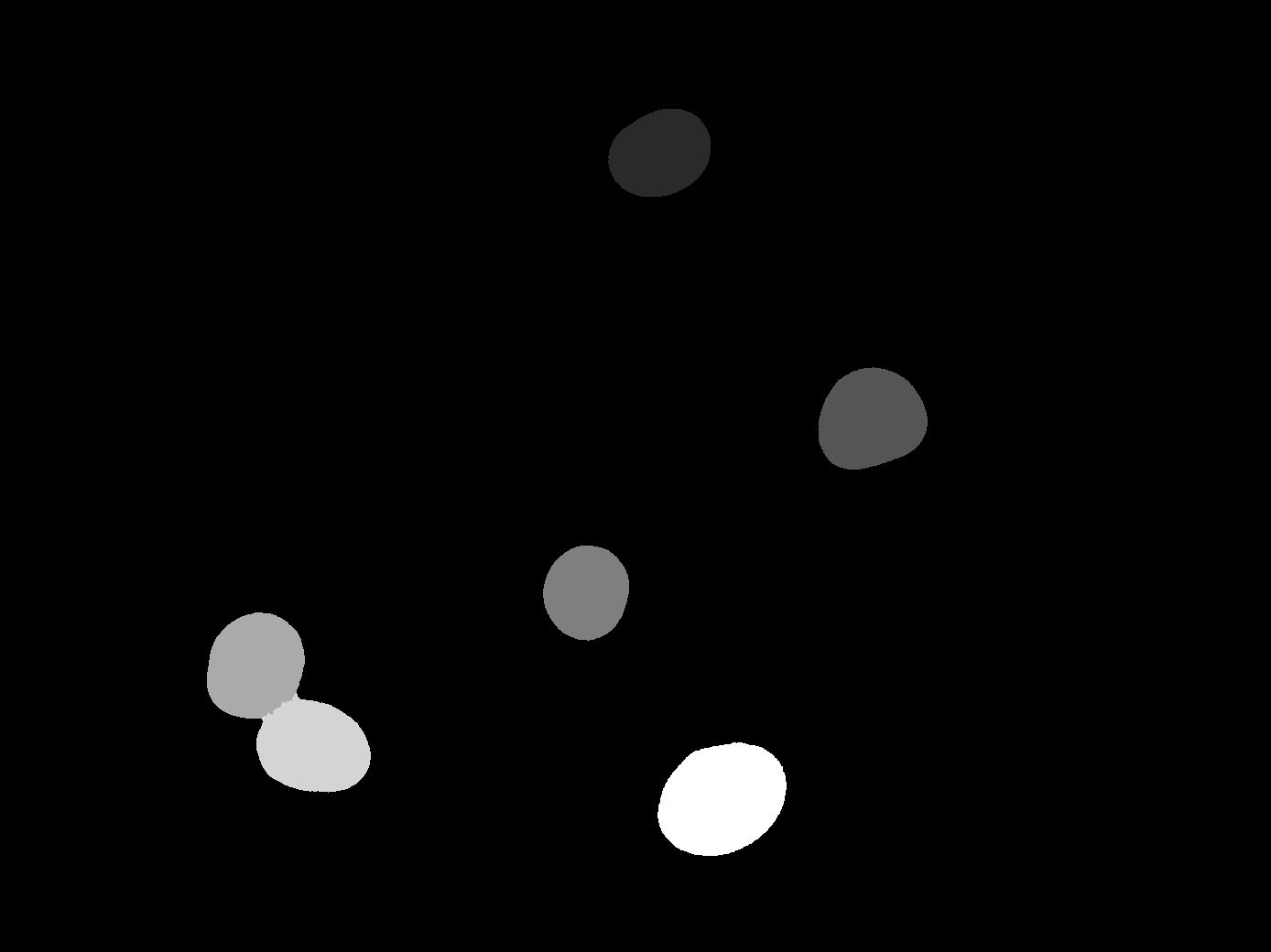

Supplement: S1 File — This file contains all scripts (CellProfiler v2.1.1 and MATLAB2016a) and data necessary to reproduce the information shown in Fig 3. (ZIP) [file pone.0180810.s001.zip › vitaminD_eColi_reproducibleResearchArchive/Results2016/B_39_c0_seg.tif]

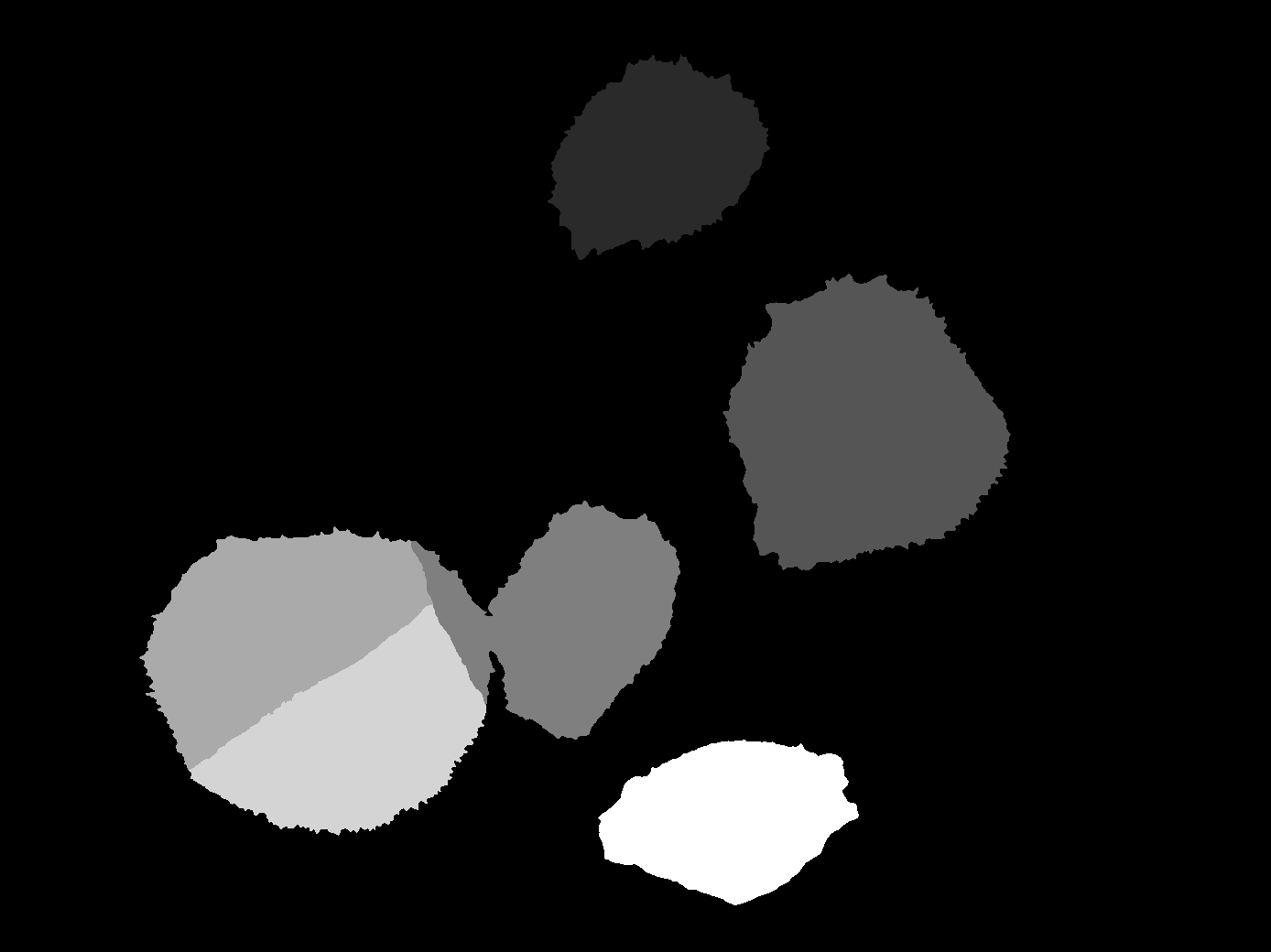

Supplement: S1 File — This file contains all scripts (CellProfiler v2.1.1 and MATLAB2016a) and data necessary to reproduce the information shown in Fig 3. (ZIP) [file pone.0180810.s001.zip › vitaminD_eColi_reproducibleResearchArchive/Results2016/B_39_c2_seg.tif]

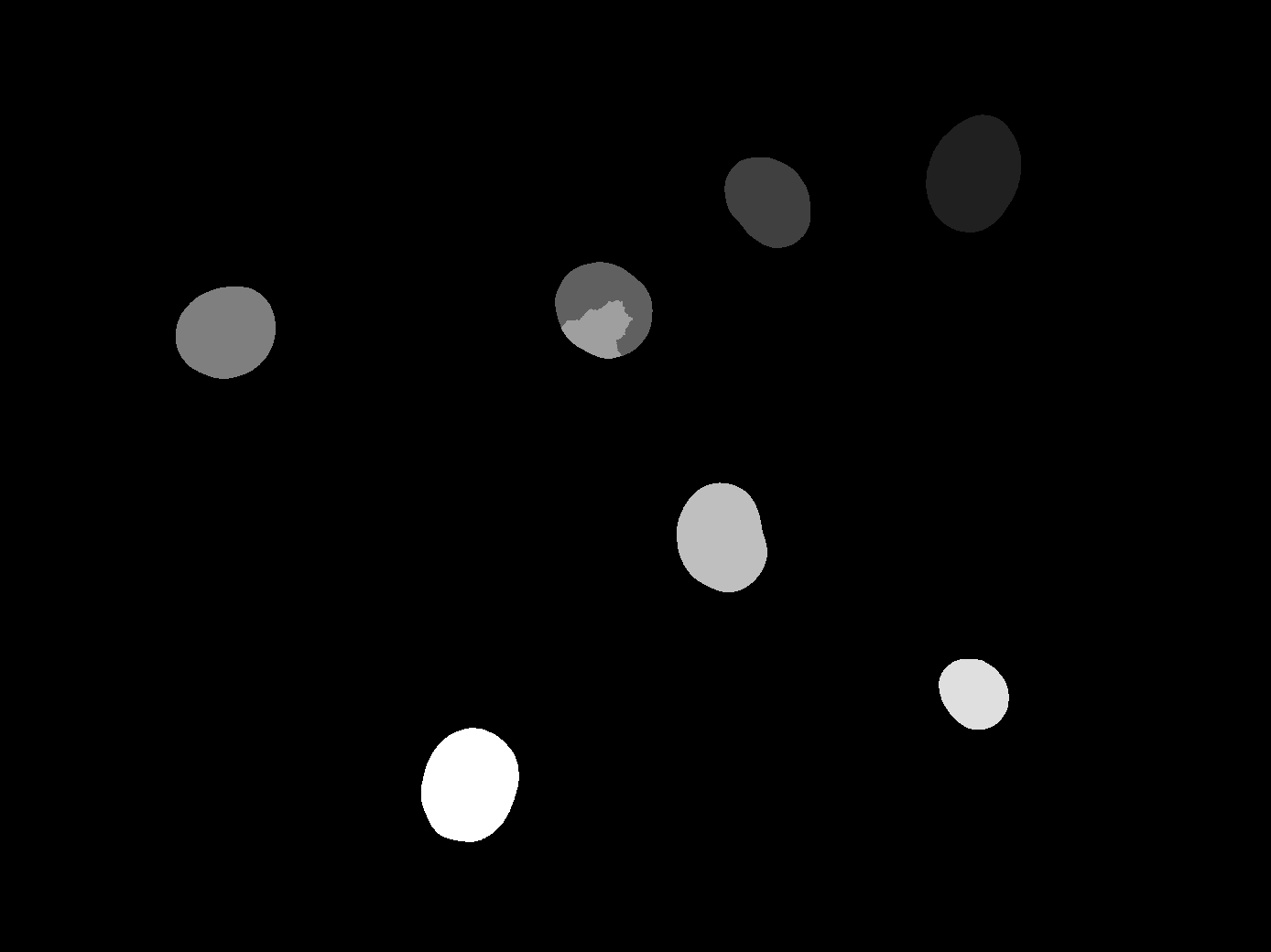

Supplement: S1 File — This file contains all scripts (CellProfiler v2.1.1 and MATLAB2016a) and data necessary to reproduce the information shown in Fig 3. (ZIP) [file pone.0180810.s001.zip › vitaminD_eColi_reproducibleResearchArchive/Results2016/B_3_c0_seg.tif]

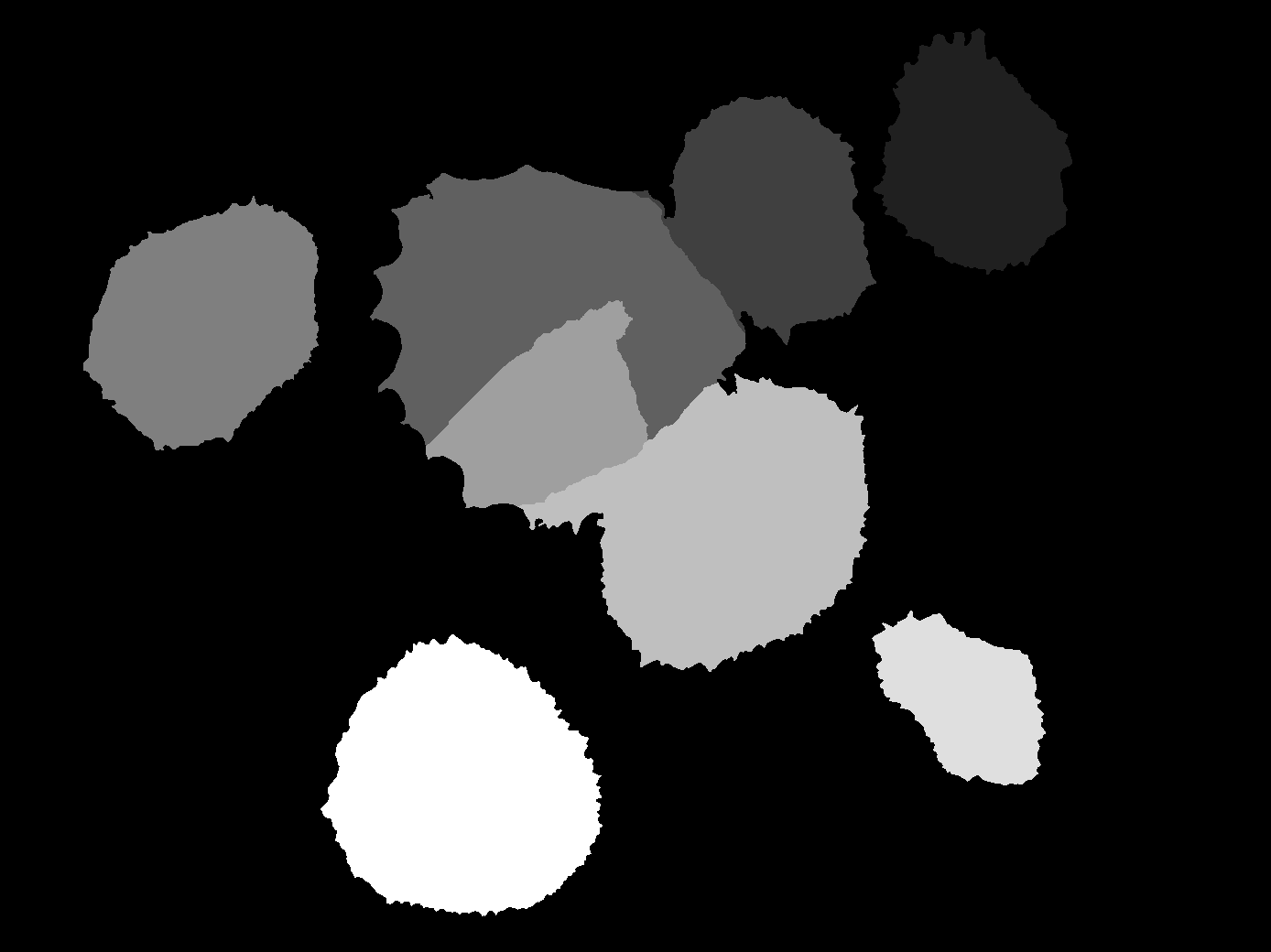

Supplement: S1 File — This file contains all scripts (CellProfiler v2.1.1 and MATLAB2016a) and data necessary to reproduce the information shown in Fig 3. (ZIP) [file pone.0180810.s001.zip › vitaminD_eColi_reproducibleResearchArchive/Results2016/B_3_c2_seg.tif]

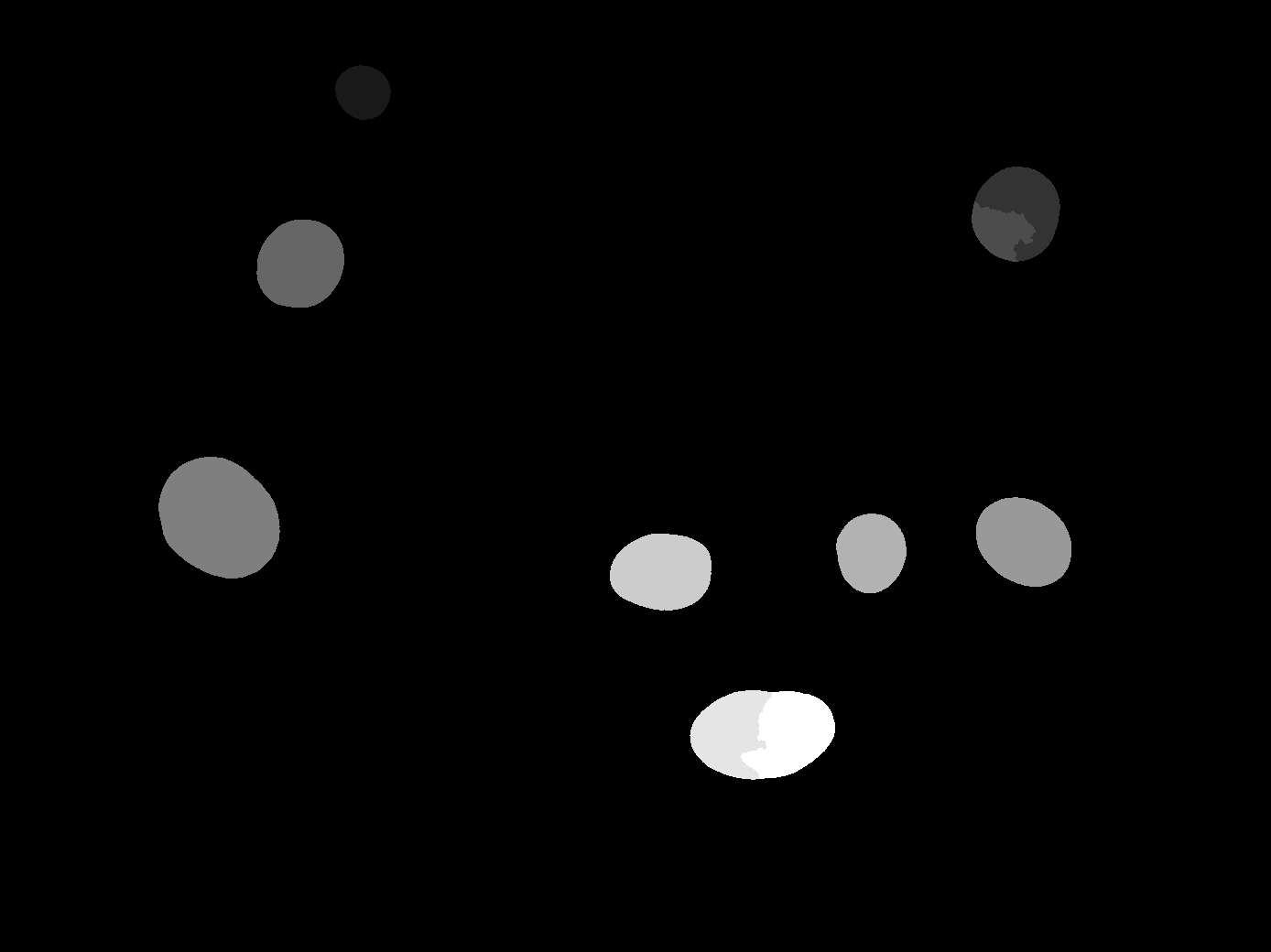

Supplement: S1 File — This file contains all scripts (CellProfiler v2.1.1 and MATLAB2016a) and data necessary to reproduce the information shown in Fig 3. (ZIP) [file pone.0180810.s001.zip › vitaminD_eColi_reproducibleResearchArchive/Results2016/B_40_c0_seg.tif]

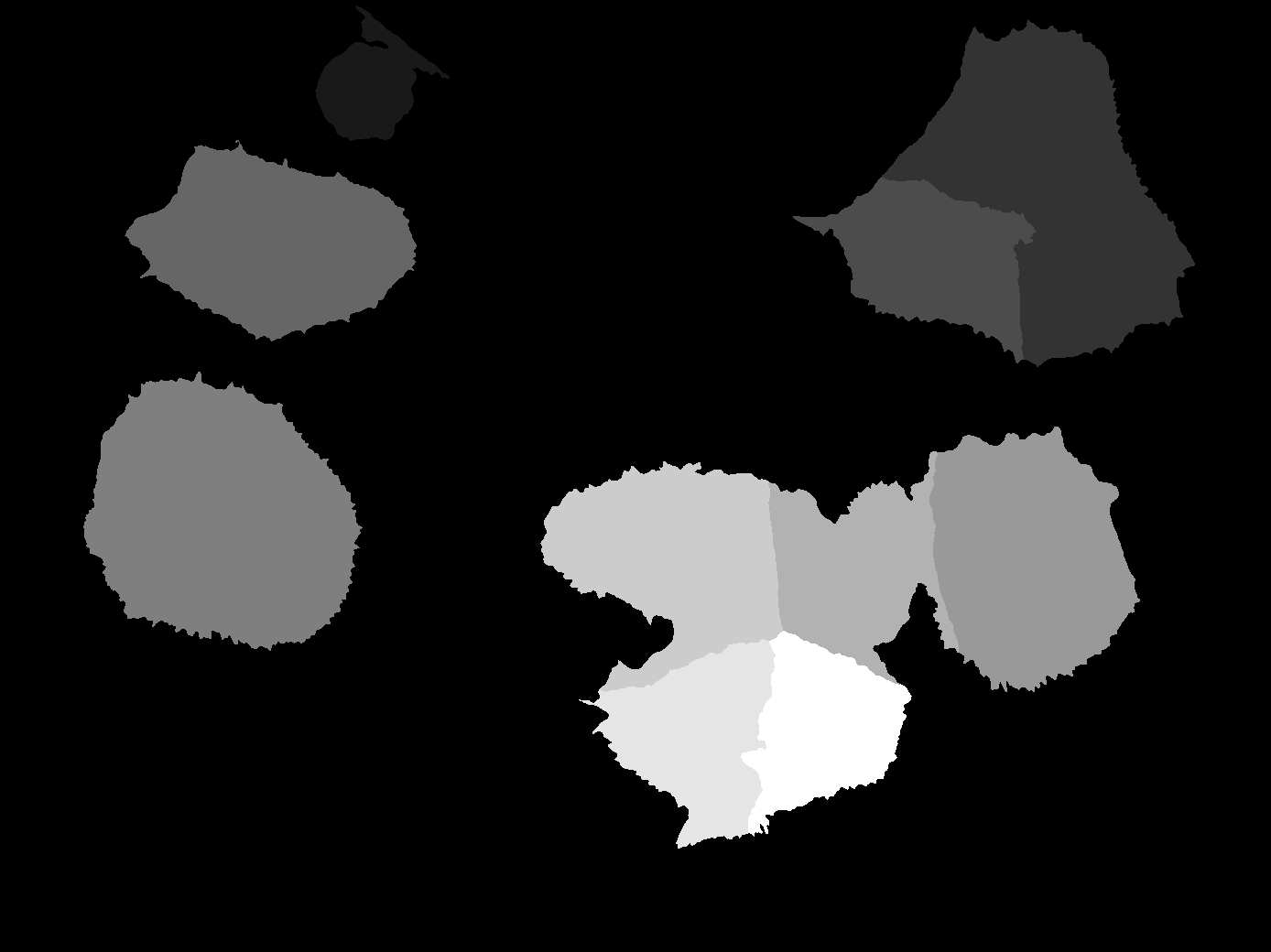

Supplement: S1 File — This file contains all scripts (CellProfiler v2.1.1 and MATLAB2016a) and data necessary to reproduce the information shown in Fig 3. (ZIP) [file pone.0180810.s001.zip › vitaminD_eColi_reproducibleResearchArchive/Results2016/B_40_c2_seg.tif]

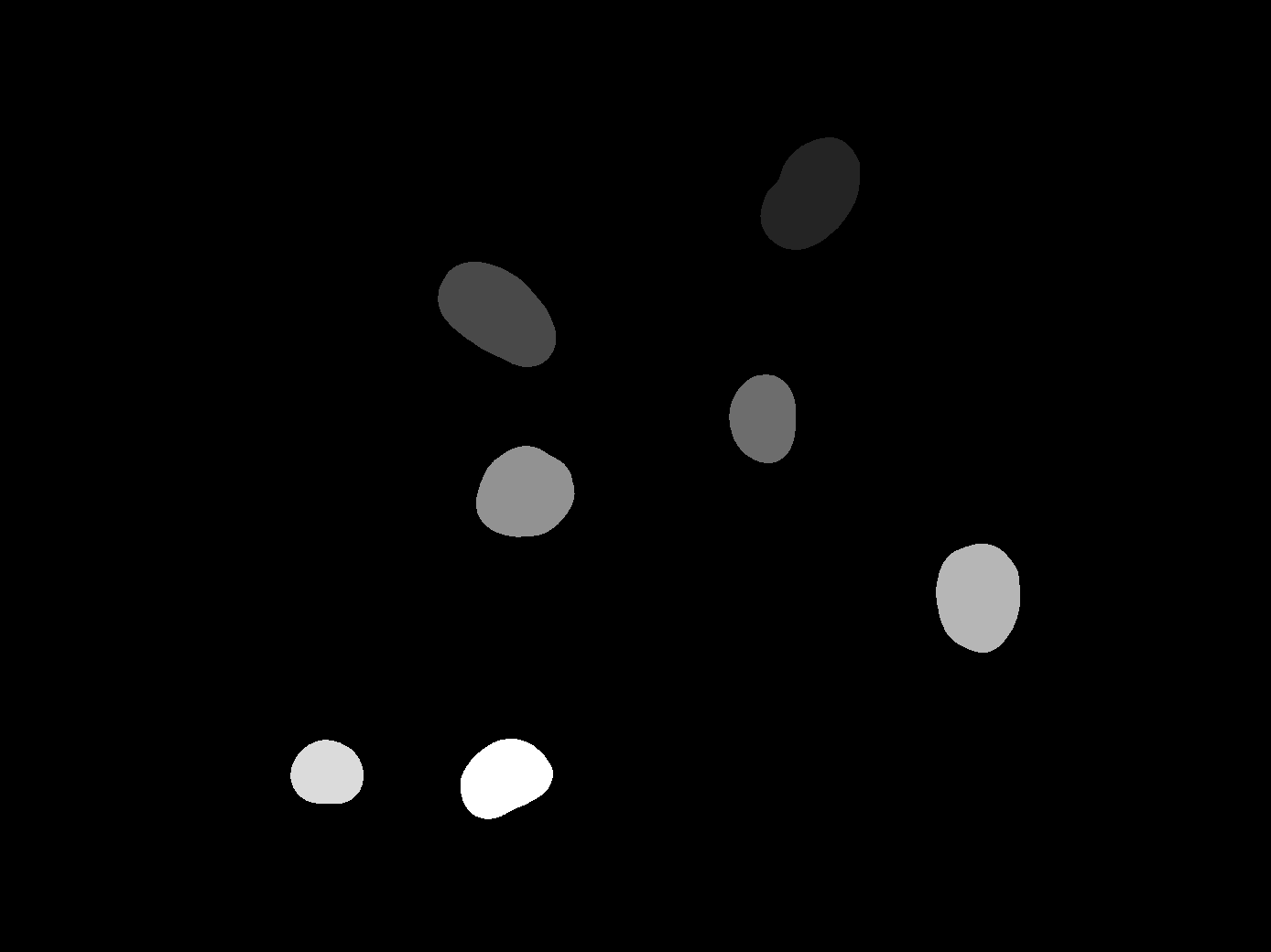

Supplement: S1 File — This file contains all scripts (CellProfiler v2.1.1 and MATLAB2016a) and data necessary to reproduce the information shown in Fig 3. (ZIP) [file pone.0180810.s001.zip › vitaminD_eColi_reproducibleResearchArchive/Results2016/B_4_c0_seg.tif]

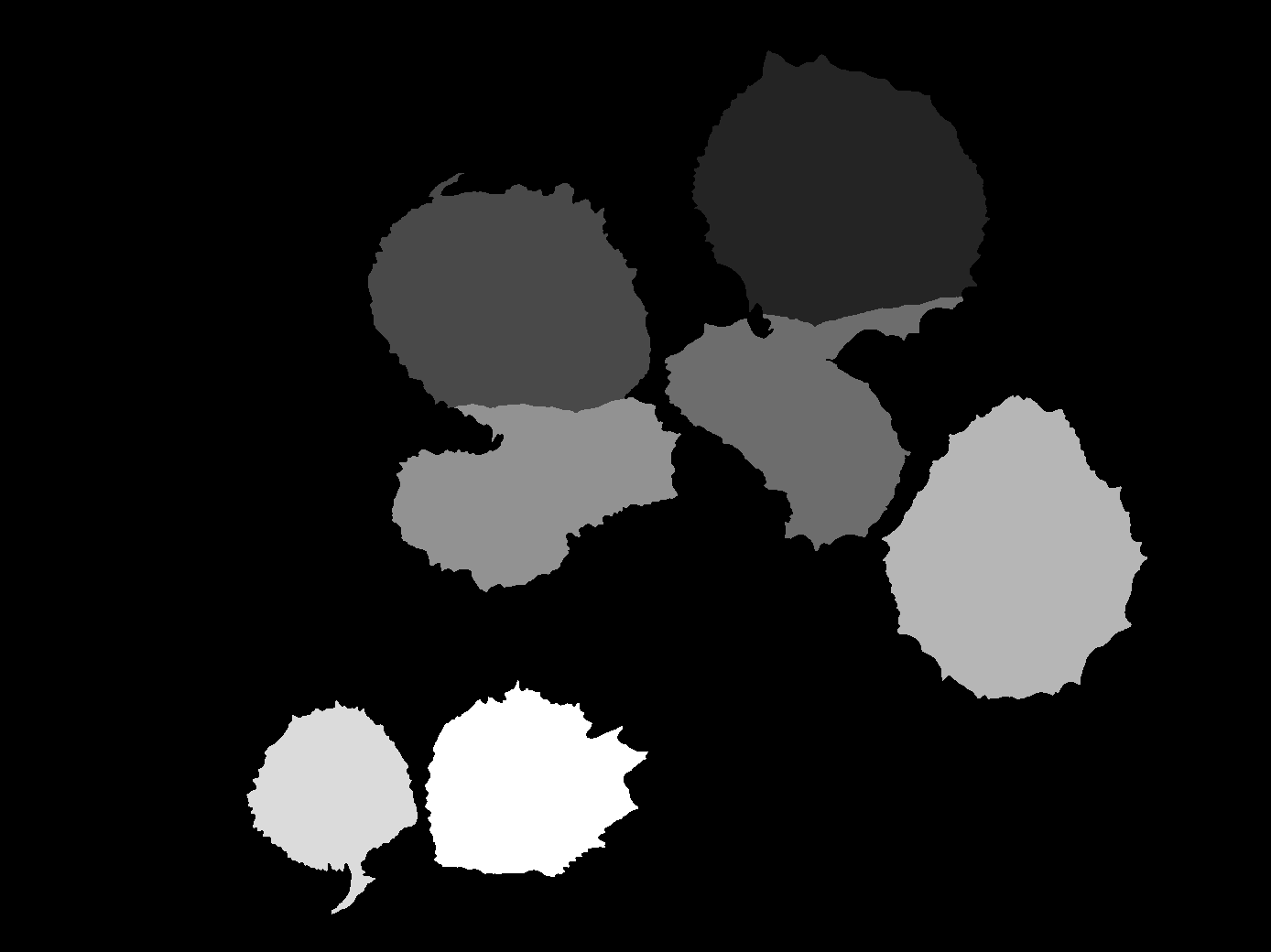

Supplement: S1 File — This file contains all scripts (CellProfiler v2.1.1 and MATLAB2016a) and data necessary to reproduce the information shown in Fig 3. (ZIP) [file pone.0180810.s001.zip › vitaminD_eColi_reproducibleResearchArchive/Results2016/B_4_c2_seg.tif]

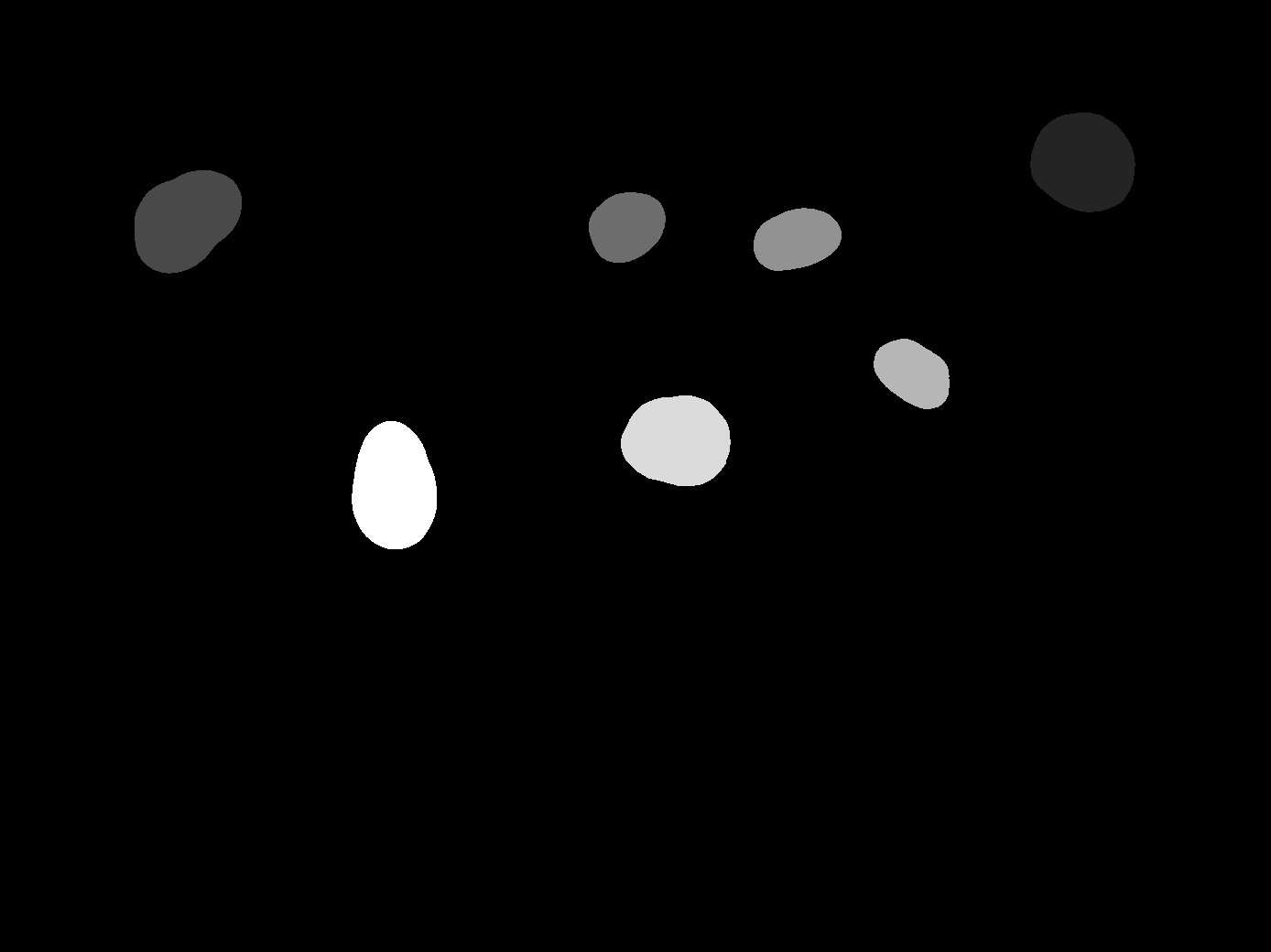

Supplement: S1 File — This file contains all scripts (CellProfiler v2.1.1 and MATLAB2016a) and data necessary to reproduce the information shown in Fig 3. (ZIP) [file pone.0180810.s001.zip › vitaminD_eColi_reproducibleResearchArchive/Results2016/B_5_c0_seg.tif]

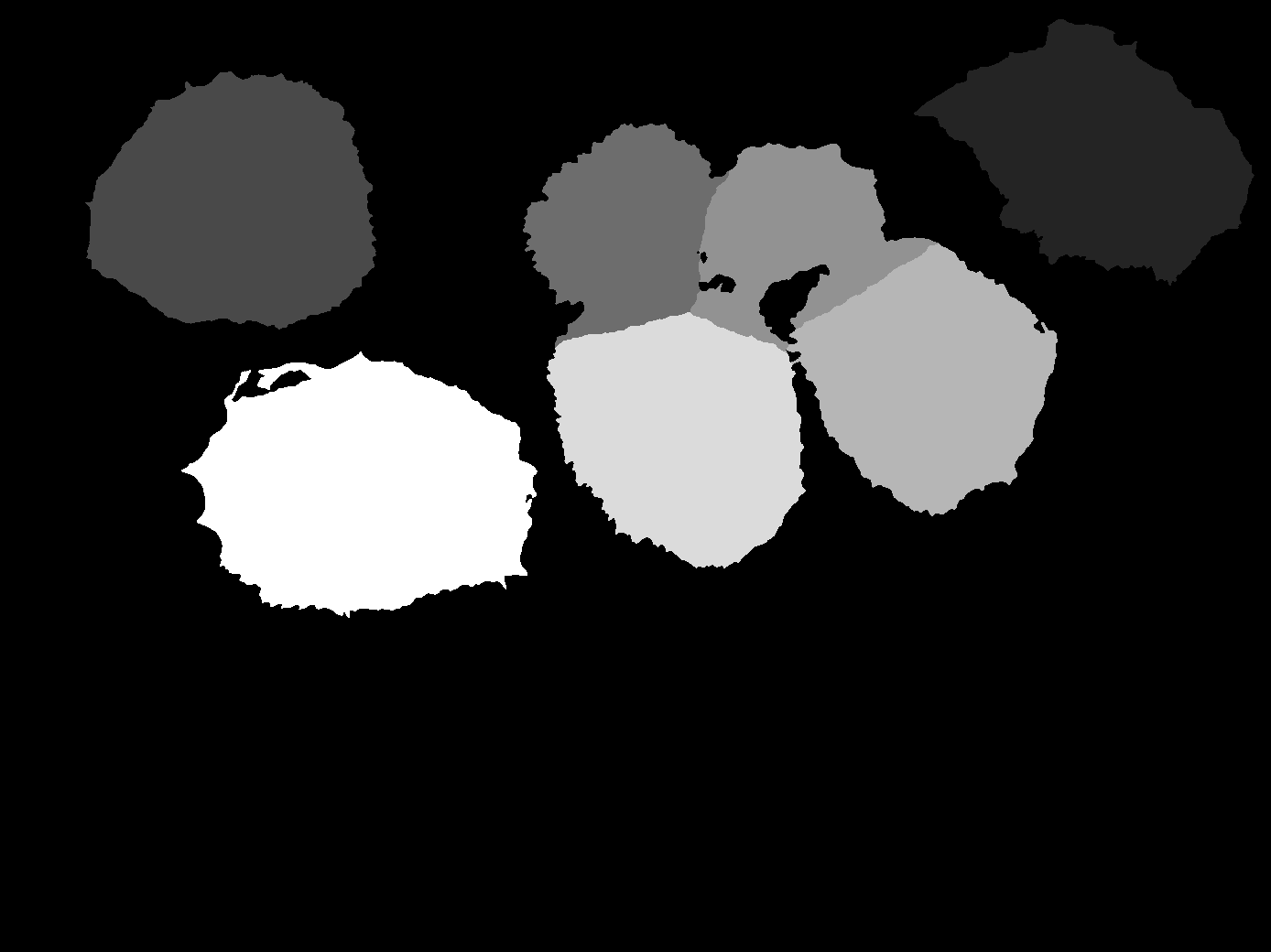

Supplement: S1 File — This file contains all scripts (CellProfiler v2.1.1 and MATLAB2016a) and data necessary to reproduce the information shown in Fig 3. (ZIP) [file pone.0180810.s001.zip › vitaminD_eColi_reproducibleResearchArchive/Results2016/B_5_c2_seg.tif]

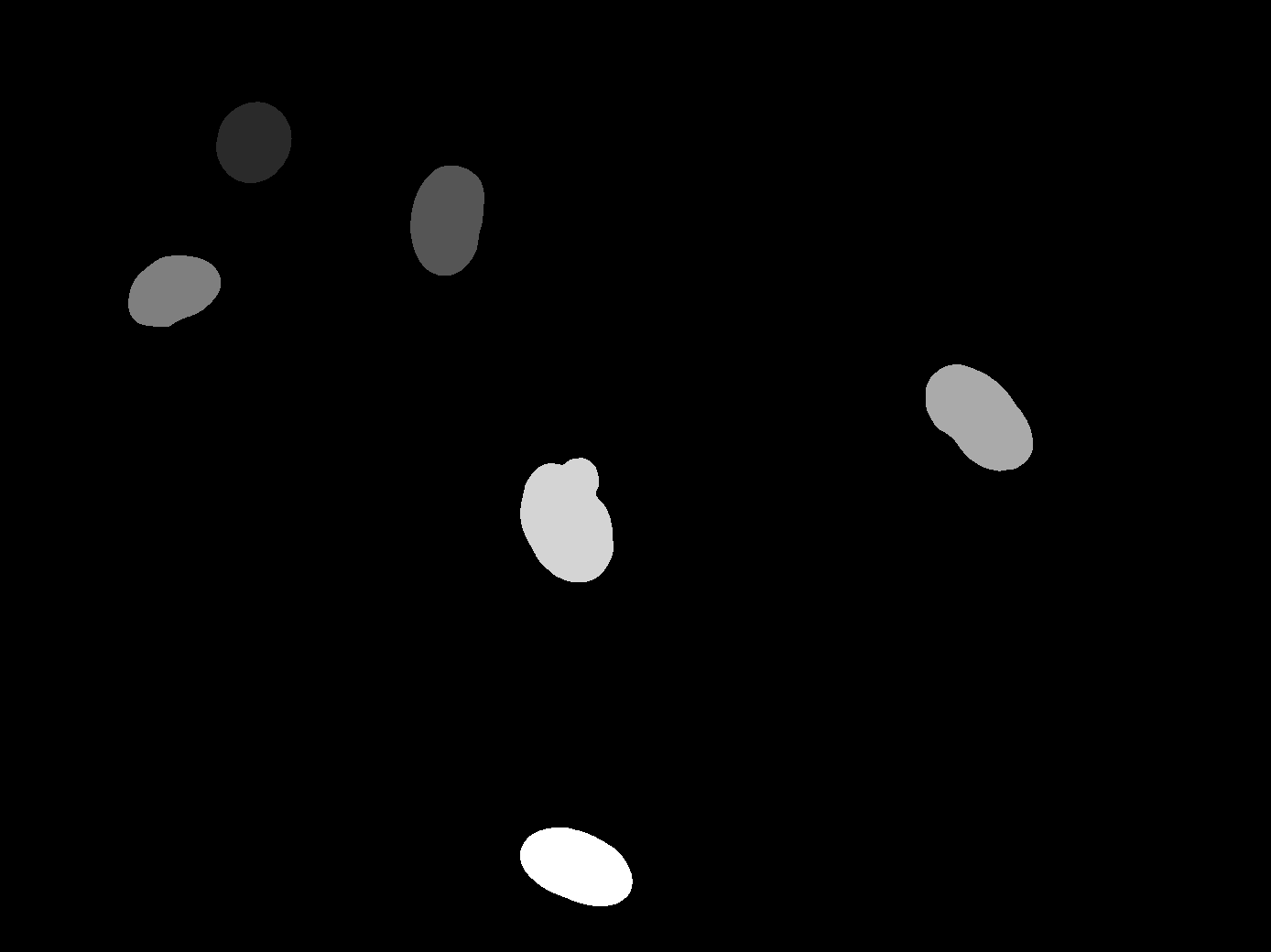

Supplement: S1 File — This file contains all scripts (CellProfiler v2.1.1 and MATLAB2016a) and data necessary to reproduce the information shown in Fig 3. (ZIP) [file pone.0180810.s001.zip › vitaminD_eColi_reproducibleResearchArchive/Results2016/B_6_c0_seg.tif]

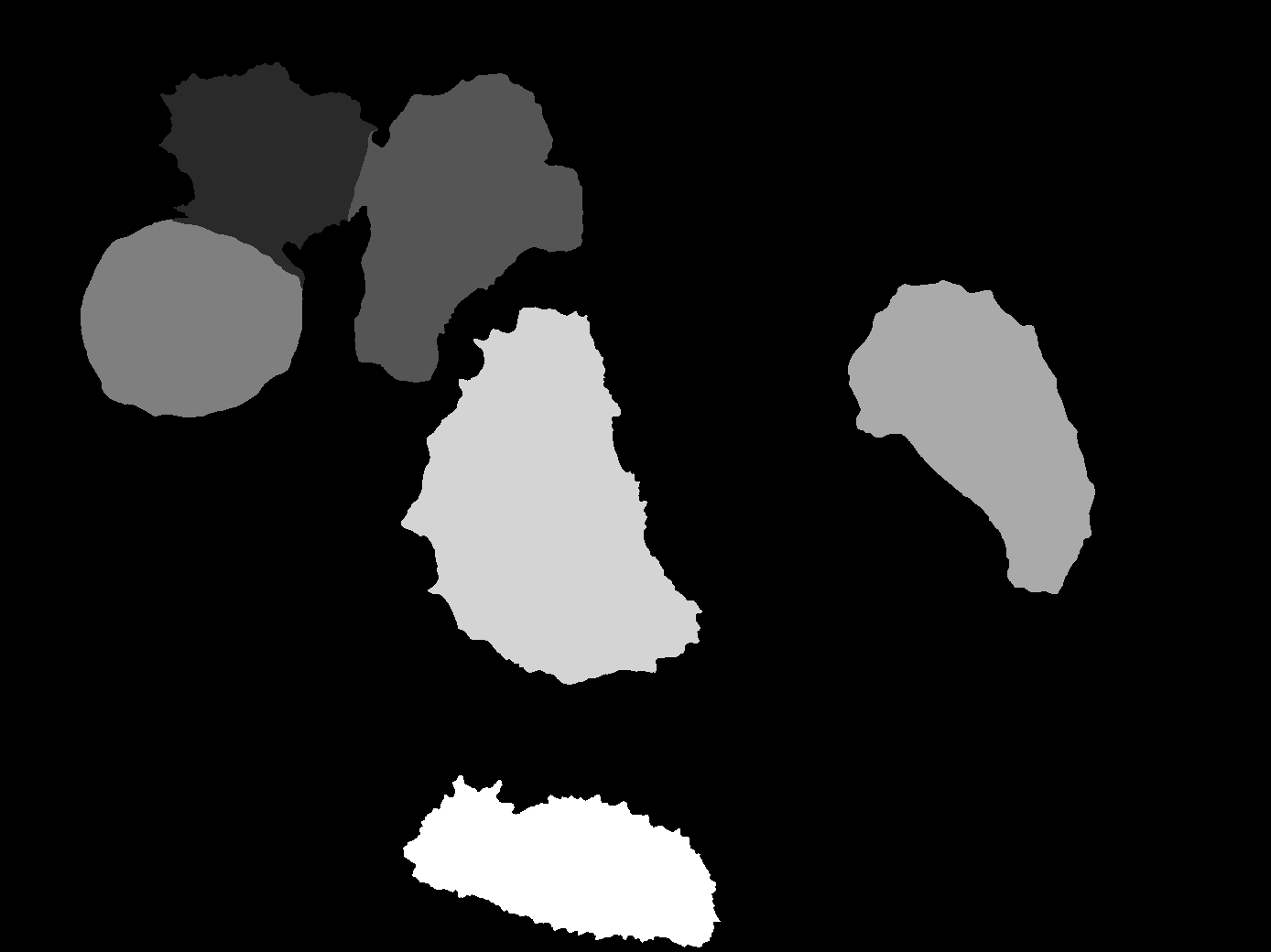

Supplement: S1 File — This file contains all scripts (CellProfiler v2.1.1 and MATLAB2016a) and data necessary to reproduce the information shown in Fig 3. (ZIP) [file pone.0180810.s001.zip › vitaminD_eColi_reproducibleResearchArchive/Results2016/B_6_c2_seg.tif]

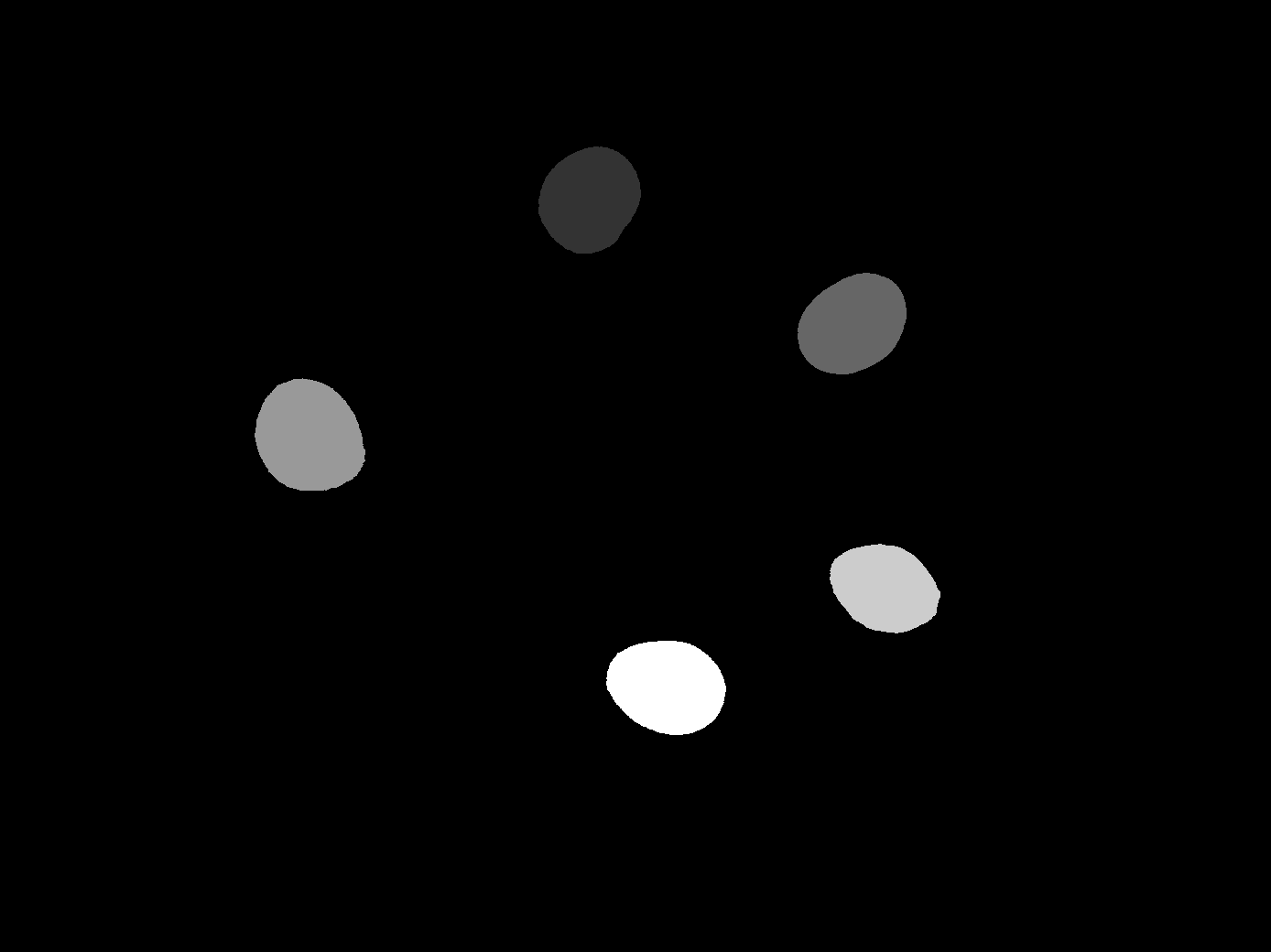

Supplement: S1 File — This file contains all scripts (CellProfiler v2.1.1 and MATLAB2016a) and data necessary to reproduce the information shown in Fig 3. (ZIP) [file pone.0180810.s001.zip › vitaminD_eColi_reproducibleResearchArchive/Results2016/B_7_c0_seg.tif]

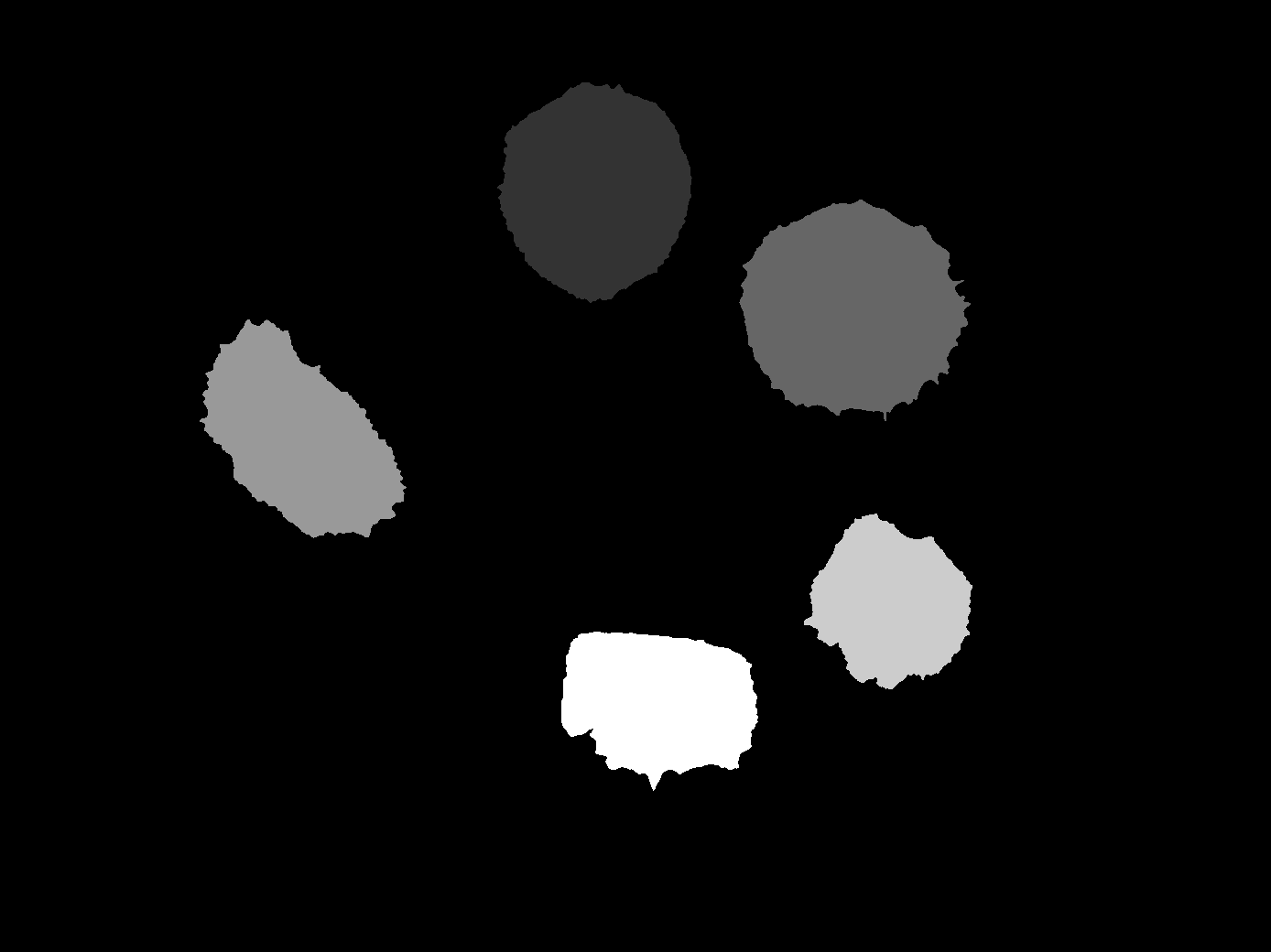

Supplement: S1 File — This file contains all scripts (CellProfiler v2.1.1 and MATLAB2016a) and data necessary to reproduce the information shown in Fig 3. (ZIP) [file pone.0180810.s001.zip › vitaminD_eColi_reproducibleResearchArchive/Results2016/B_7_c2_seg.tif]

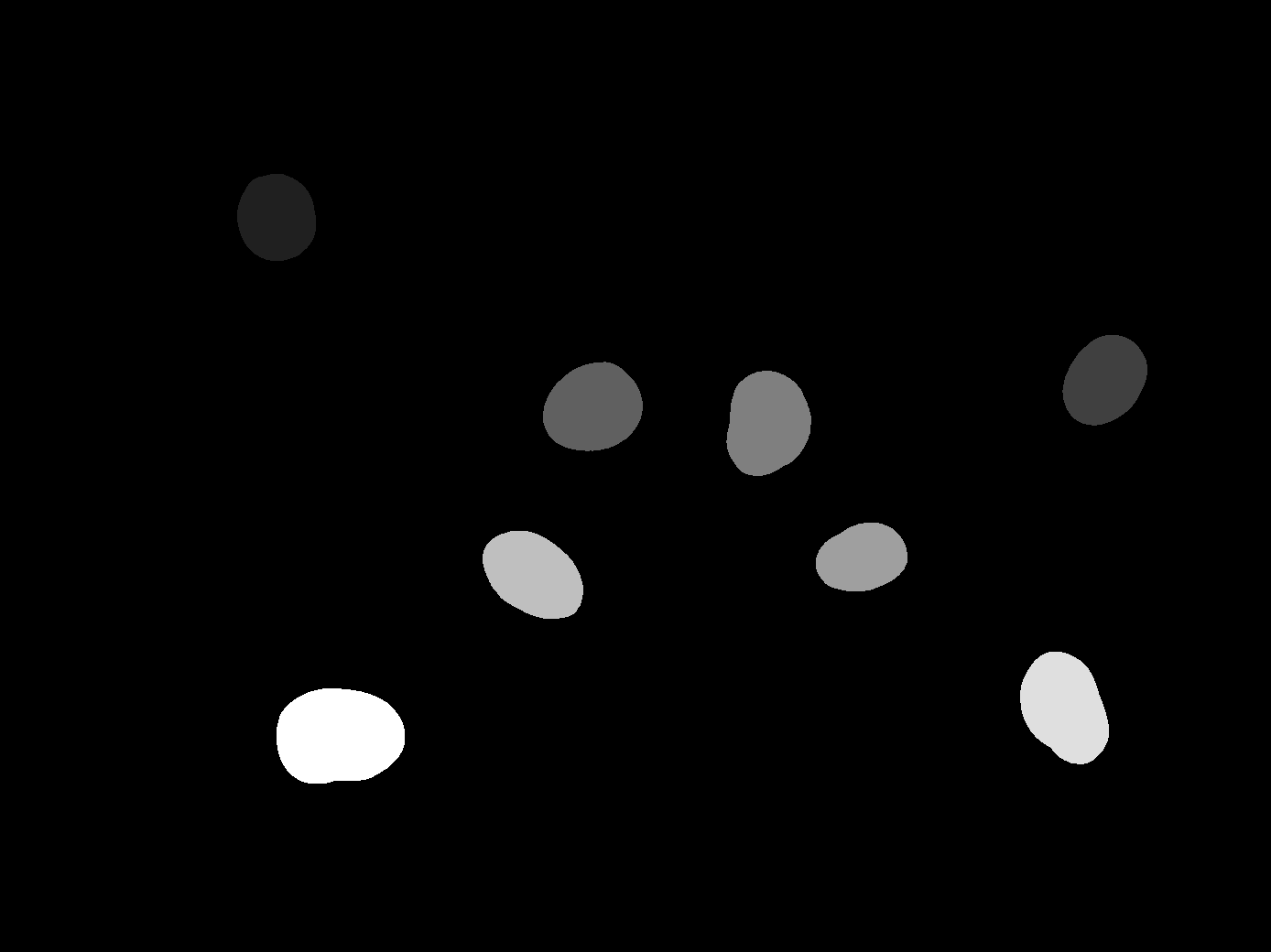

Supplement: S1 File — This file contains all scripts (CellProfiler v2.1.1 and MATLAB2016a) and data necessary to reproduce the information shown in Fig 3. (ZIP) [file pone.0180810.s001.zip › vitaminD_eColi_reproducibleResearchArchive/Results2016/B_8_c0_seg.tif]

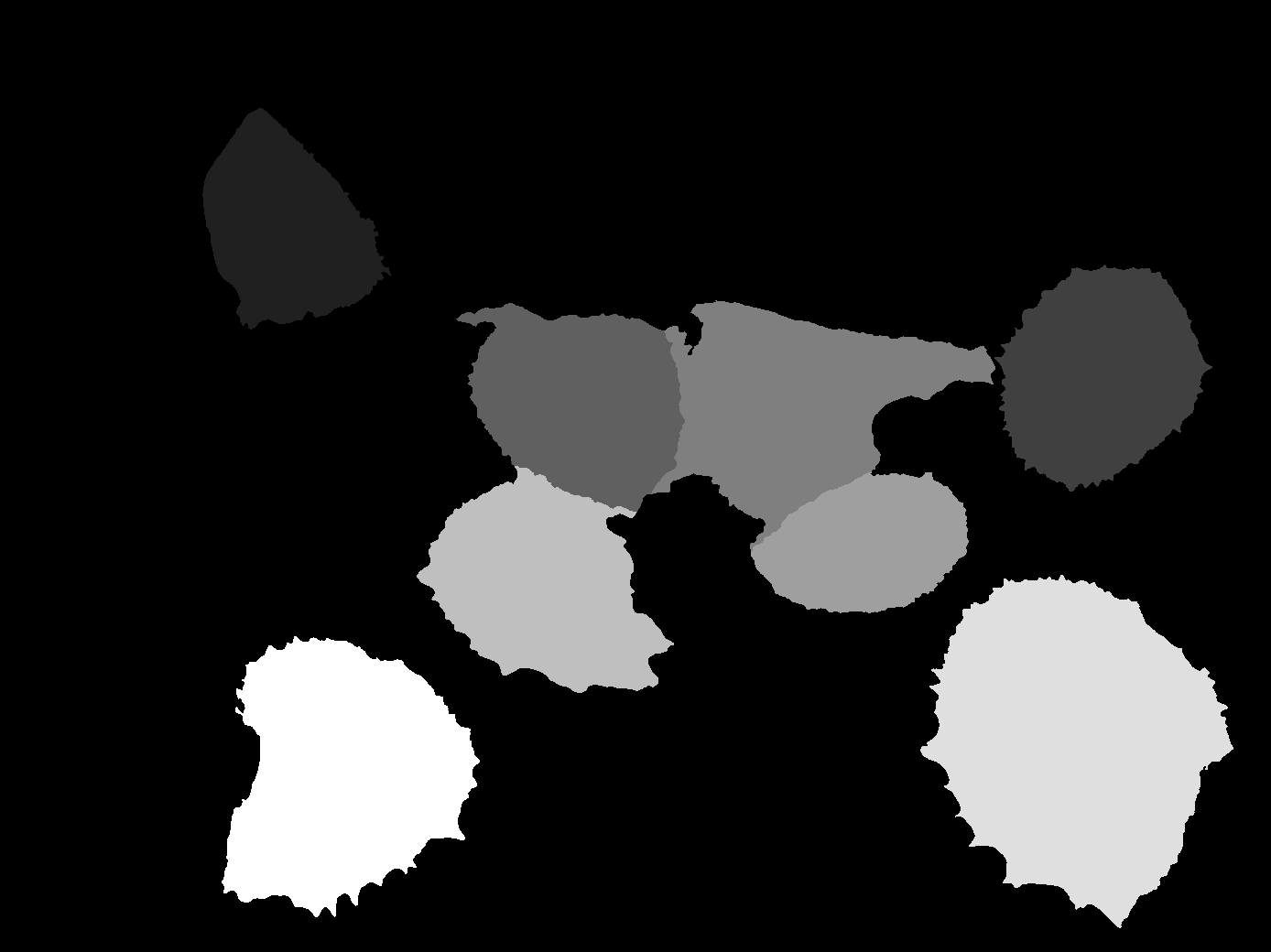

Supplement: S1 File — This file contains all scripts (CellProfiler v2.1.1 and MATLAB2016a) and data necessary to reproduce the information shown in Fig 3. (ZIP) [file pone.0180810.s001.zip › vitaminD_eColi_reproducibleResearchArchive/Results2016/B_8_c2_seg.tif]

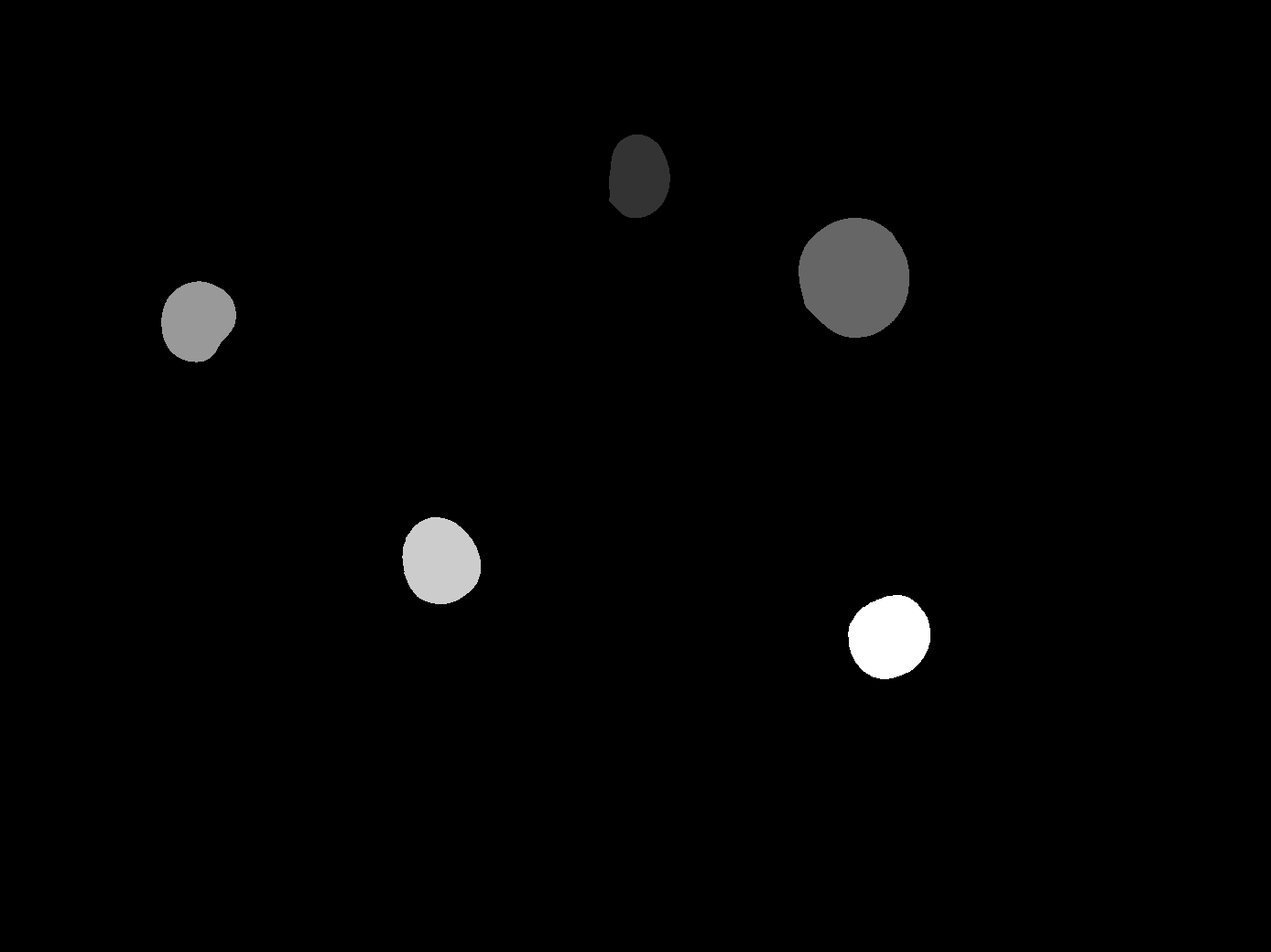

Supplement: S1 File — This file contains all scripts (CellProfiler v2.1.1 and MATLAB2016a) and data necessary to reproduce the information shown in Fig 3. (ZIP) [file pone.0180810.s001.zip › vitaminD_eColi_reproducibleResearchArchive/Results2016/B_9_c0_seg.tif]

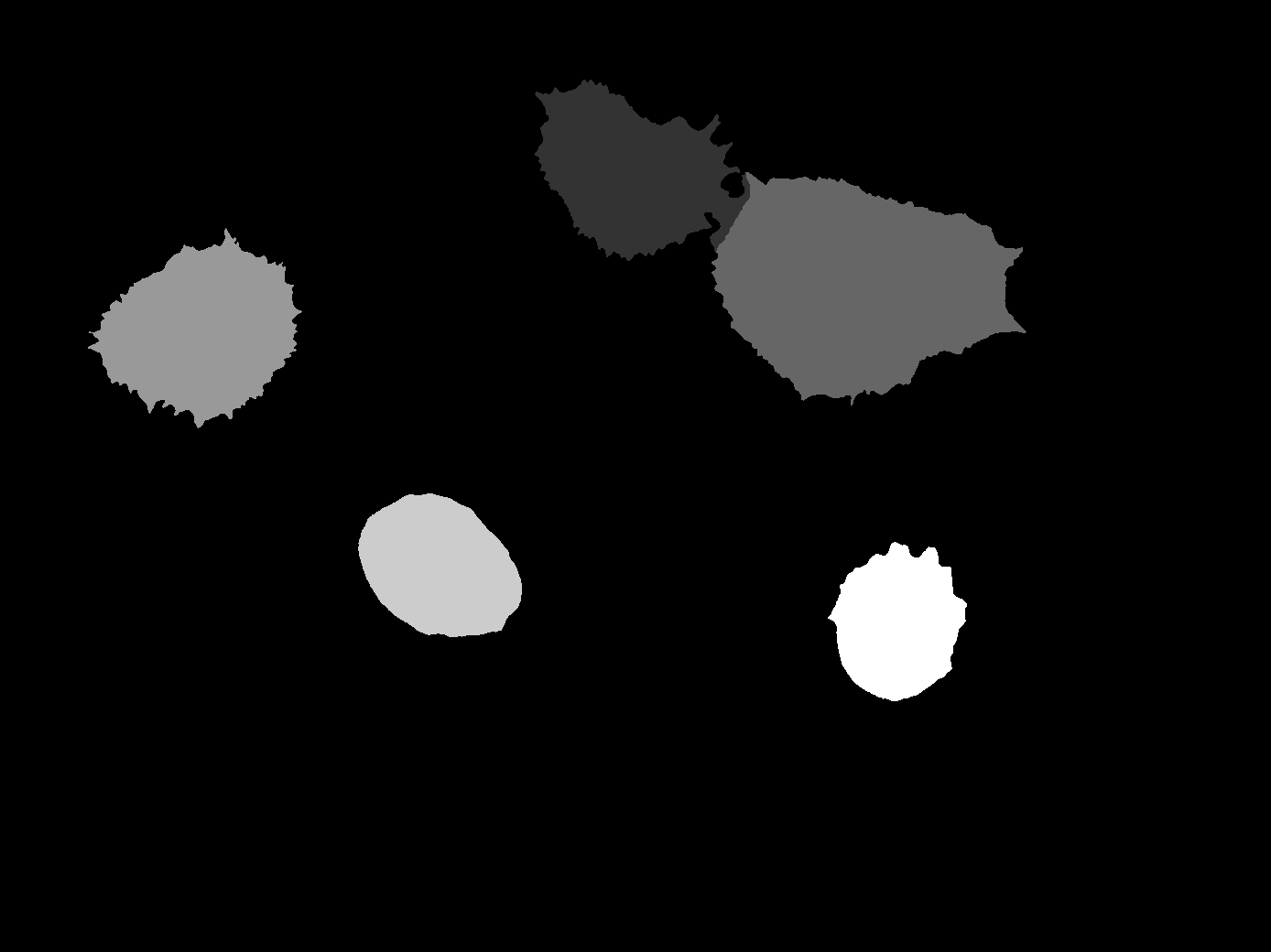

Supplement: S1 File — This file contains all scripts (CellProfiler v2.1.1 and MATLAB2016a) and data necessary to reproduce the information shown in Fig 3. (ZIP) [file pone.0180810.s001.zip › vitaminD_eColi_reproducibleResearchArchive/Results2016/B_9_c2_seg.tif]

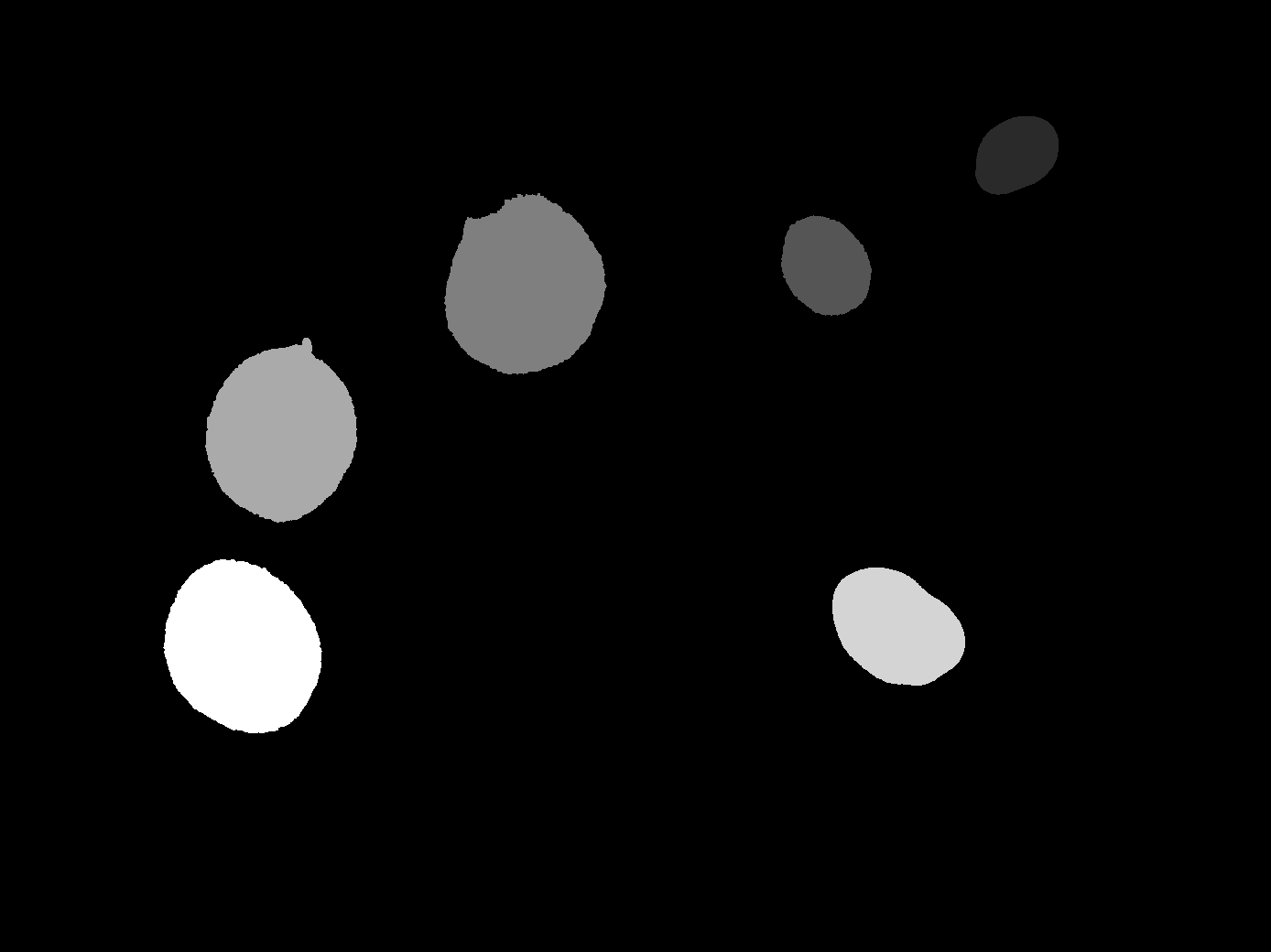

Supplement: S1 File — This file contains all scripts (CellProfiler v2.1.1 and MATLAB2016a) and data necessary to reproduce the information shown in Fig 3. (ZIP) [file pone.0180810.s001.zip › vitaminD_eColi_reproducibleResearchArchive/Results2016/C_10_c0_seg.tif]

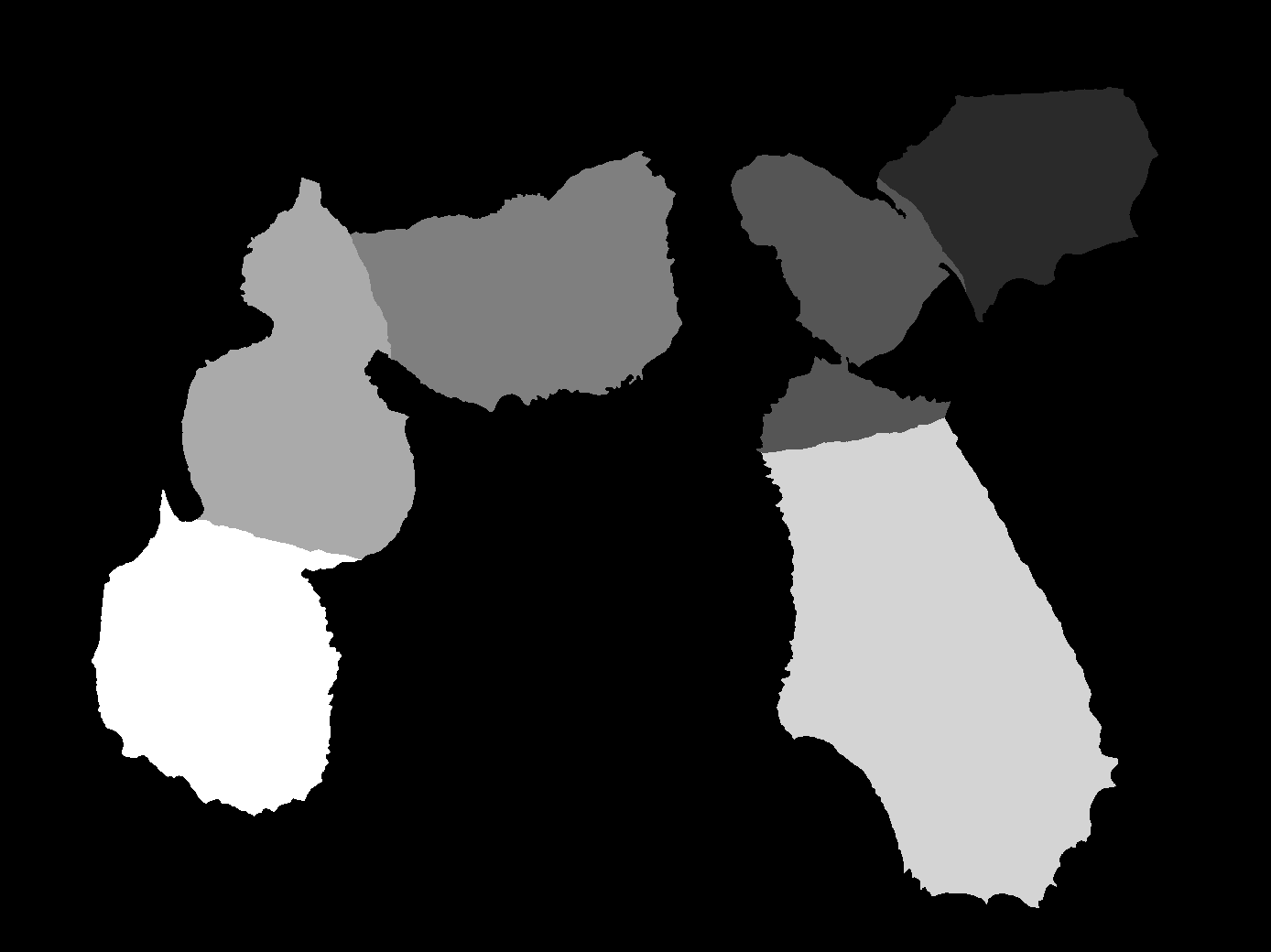

Supplement: S1 File — This file contains all scripts (CellProfiler v2.1.1 and MATLAB2016a) and data necessary to reproduce the information shown in Fig 3. (ZIP) [file pone.0180810.s001.zip › vitaminD_eColi_reproducibleResearchArchive/Results2016/C_10_c2_seg.tif]

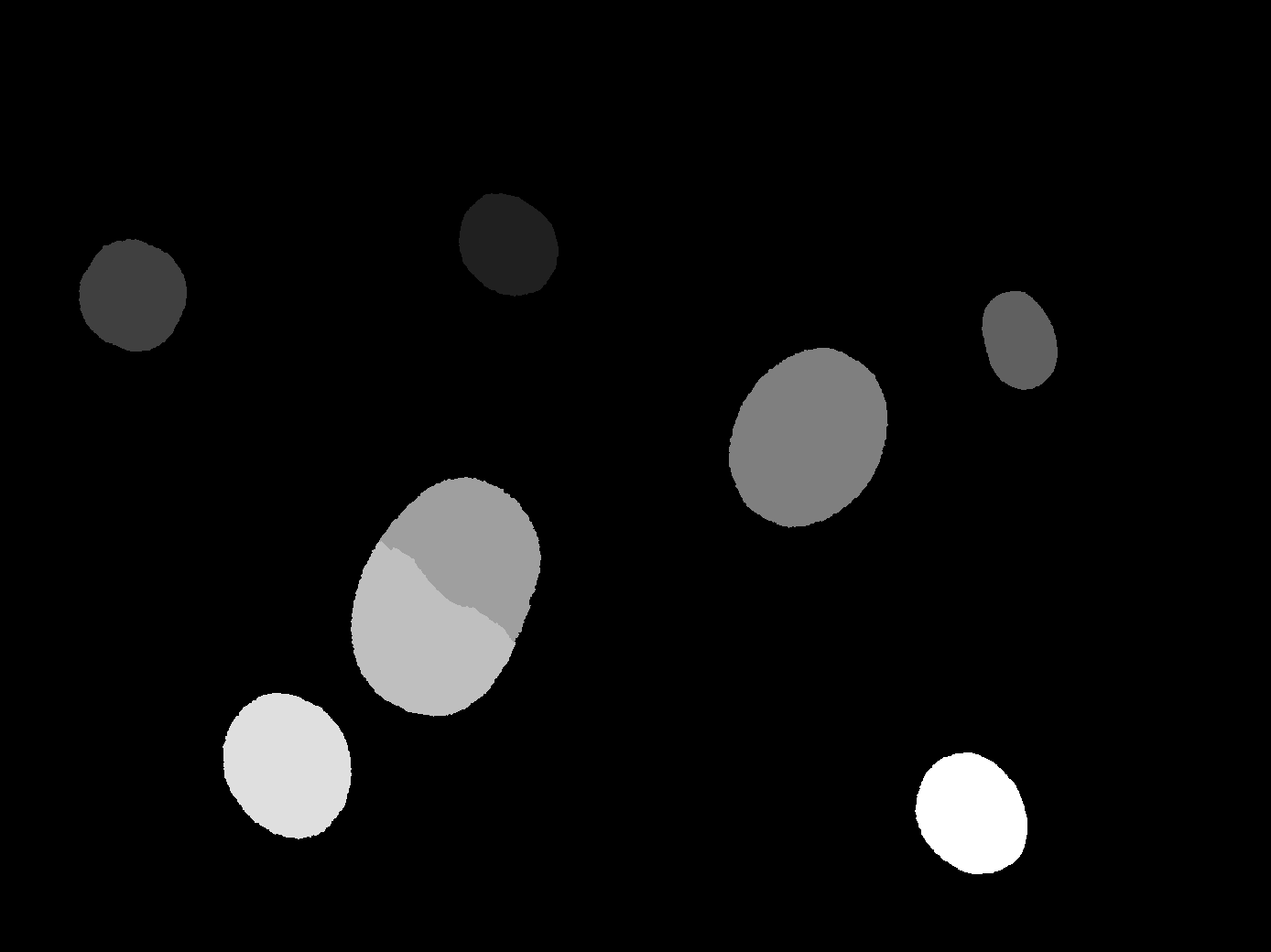

Supplement: S1 File — This file contains all scripts (CellProfiler v2.1.1 and MATLAB2016a) and data necessary to reproduce the information shown in Fig 3. (ZIP) [file pone.0180810.s001.zip › vitaminD_eColi_reproducibleResearchArchive/Results2016/C_11_c0_seg.tif]

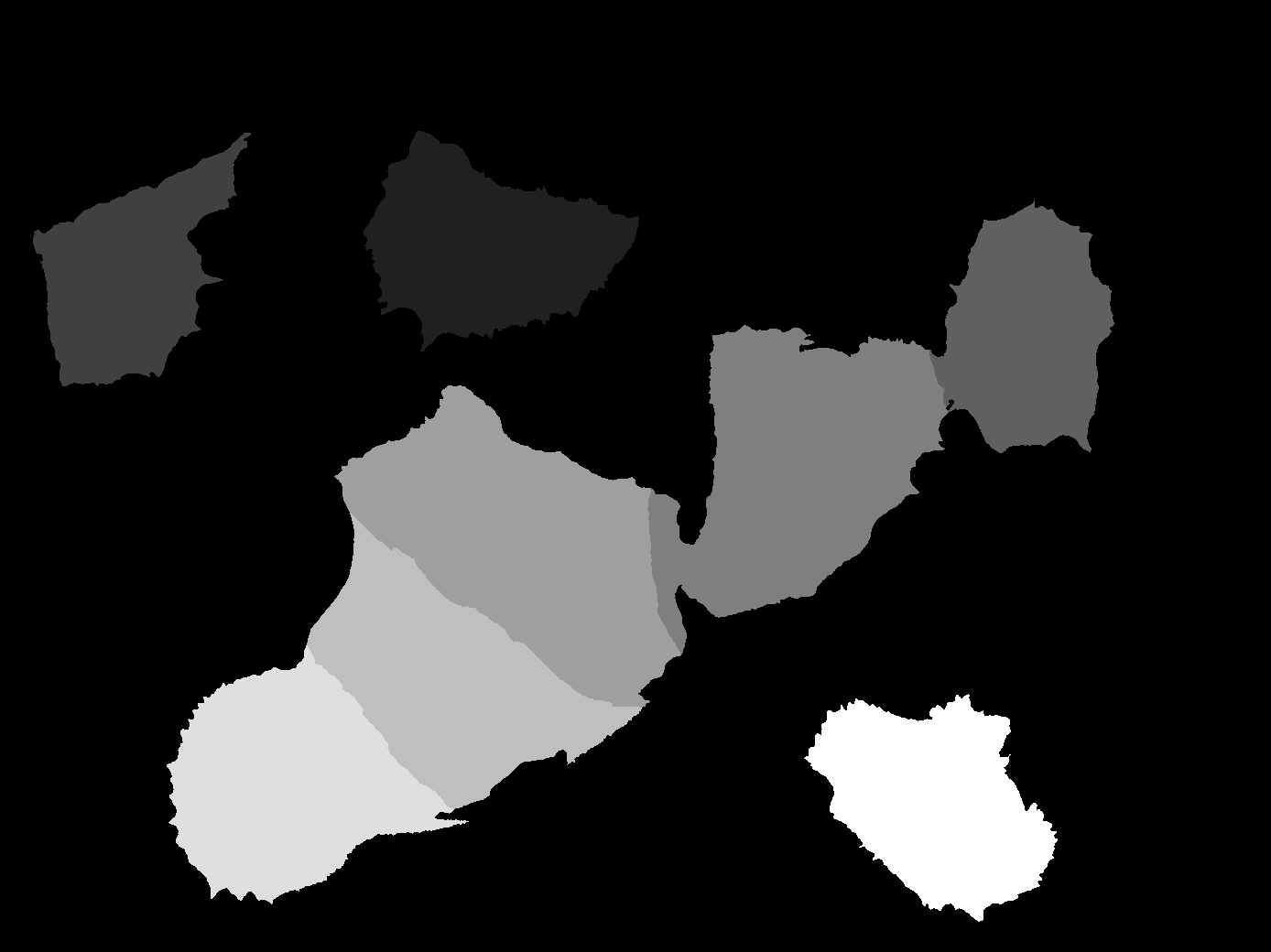

Supplement: S1 File — This file contains all scripts (CellProfiler v2.1.1 and MATLAB2016a) and data necessary to reproduce the information shown in Fig 3. (ZIP) [file pone.0180810.s001.zip › vitaminD_eColi_reproducibleResearchArchive/Results2016/C_11_c2_seg.tif]

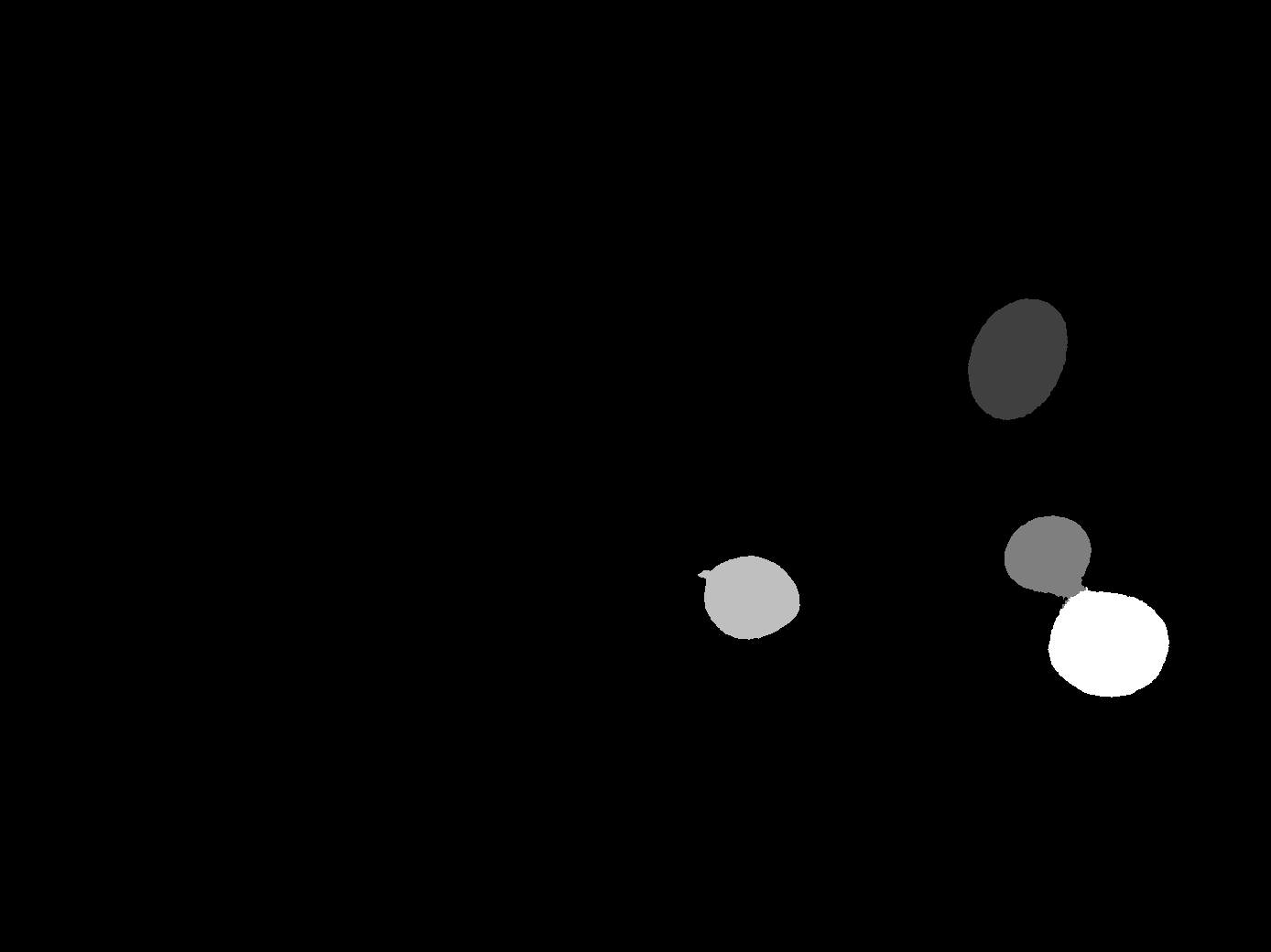

Supplement: S1 File — This file contains all scripts (CellProfiler v2.1.1 and MATLAB2016a) and data necessary to reproduce the information shown in Fig 3. (ZIP) [file pone.0180810.s001.zip › vitaminD_eColi_reproducibleResearchArchive/Results2016/C_12_c0_seg.tif]

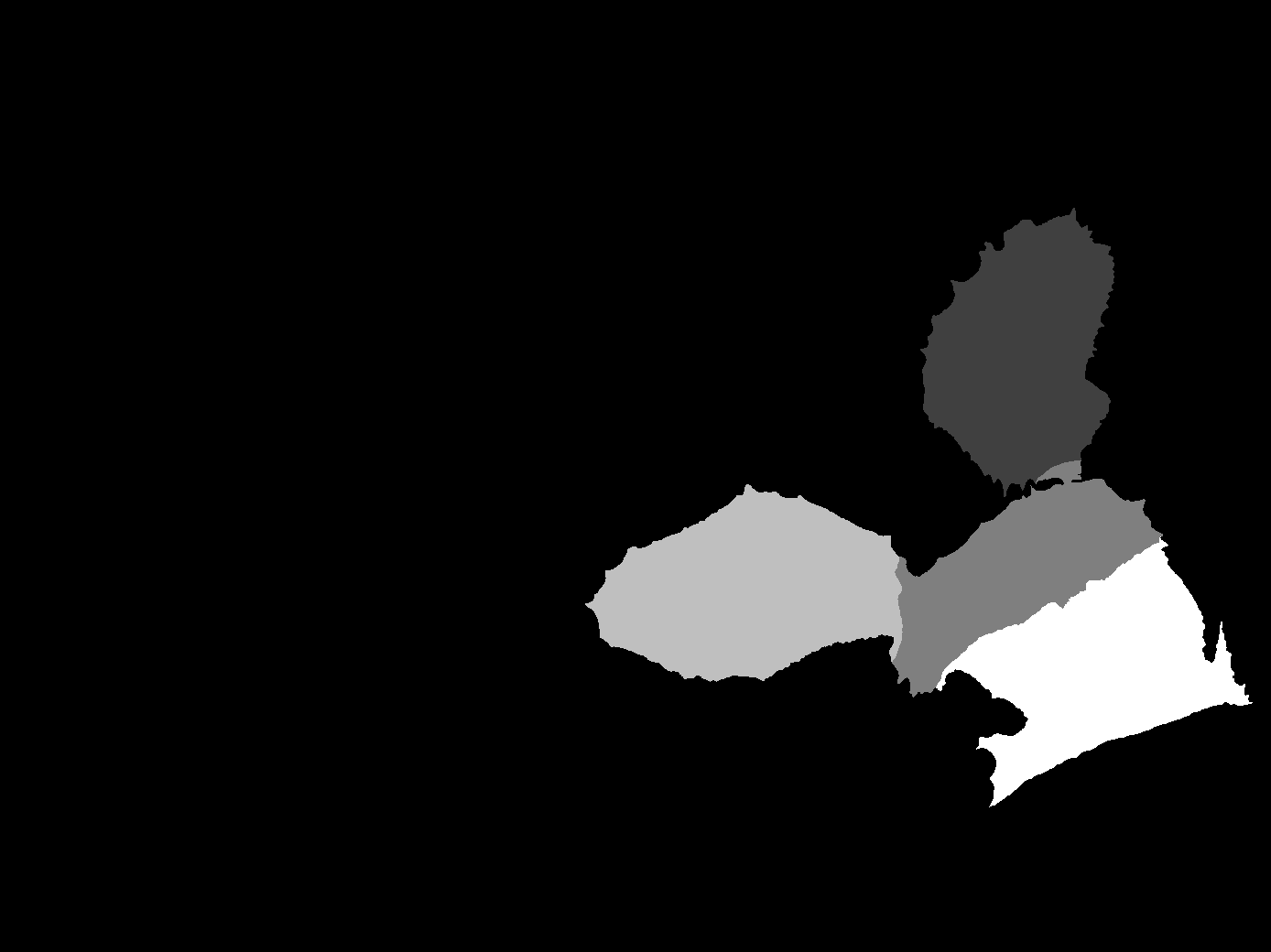

Supplement: S1 File — This file contains all scripts (CellProfiler v2.1.1 and MATLAB2016a) and data necessary to reproduce the information shown in Fig 3. (ZIP) [file pone.0180810.s001.zip › vitaminD_eColi_reproducibleResearchArchive/Results2016/C_12_c2_seg.tif]

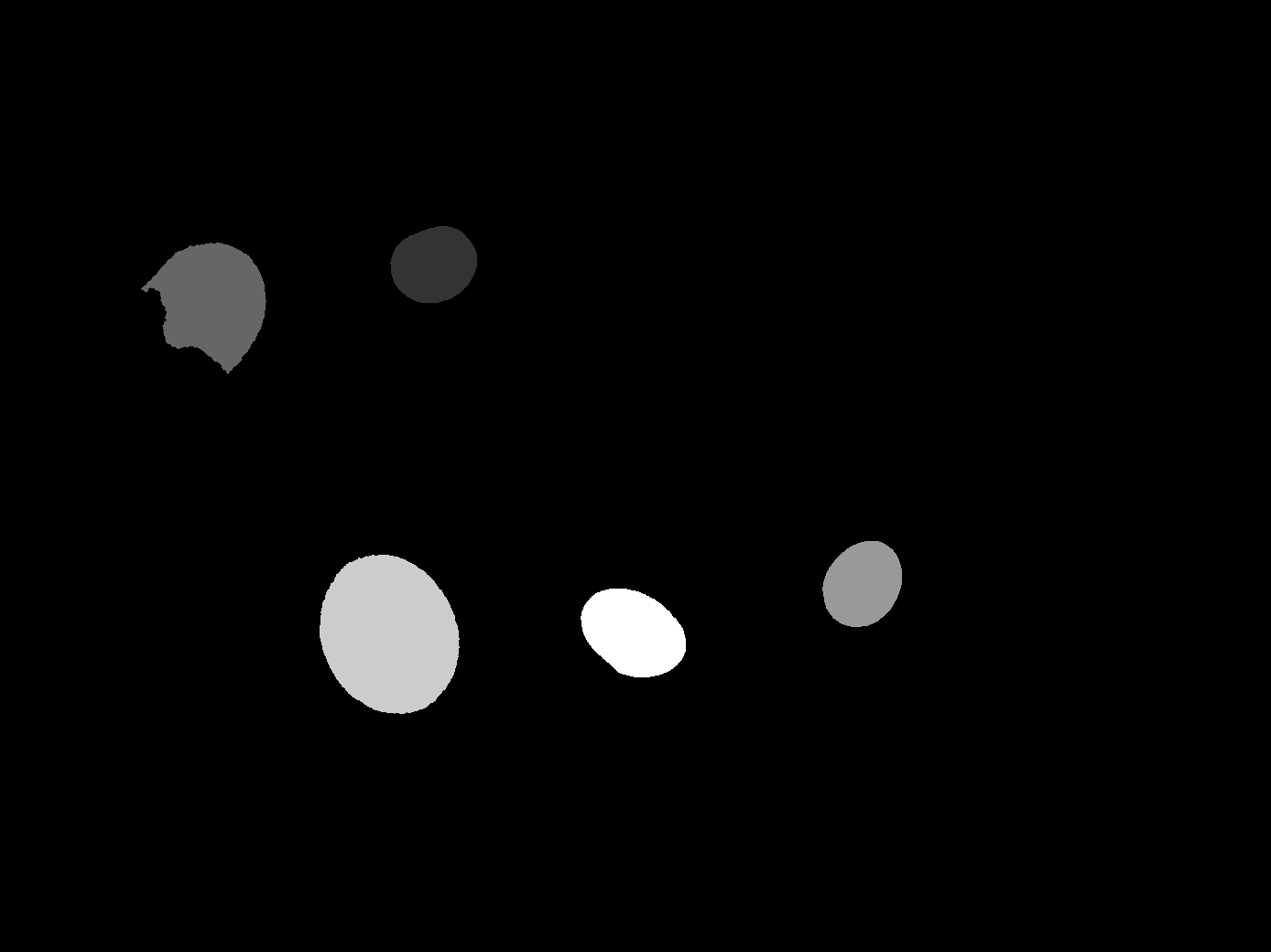

Supplement: S1 File — This file contains all scripts (CellProfiler v2.1.1 and MATLAB2016a) and data necessary to reproduce the information shown in Fig 3. (ZIP) [file pone.0180810.s001.zip › vitaminD_eColi_reproducibleResearchArchive/Results2016/C_13_c0_seg.tif]

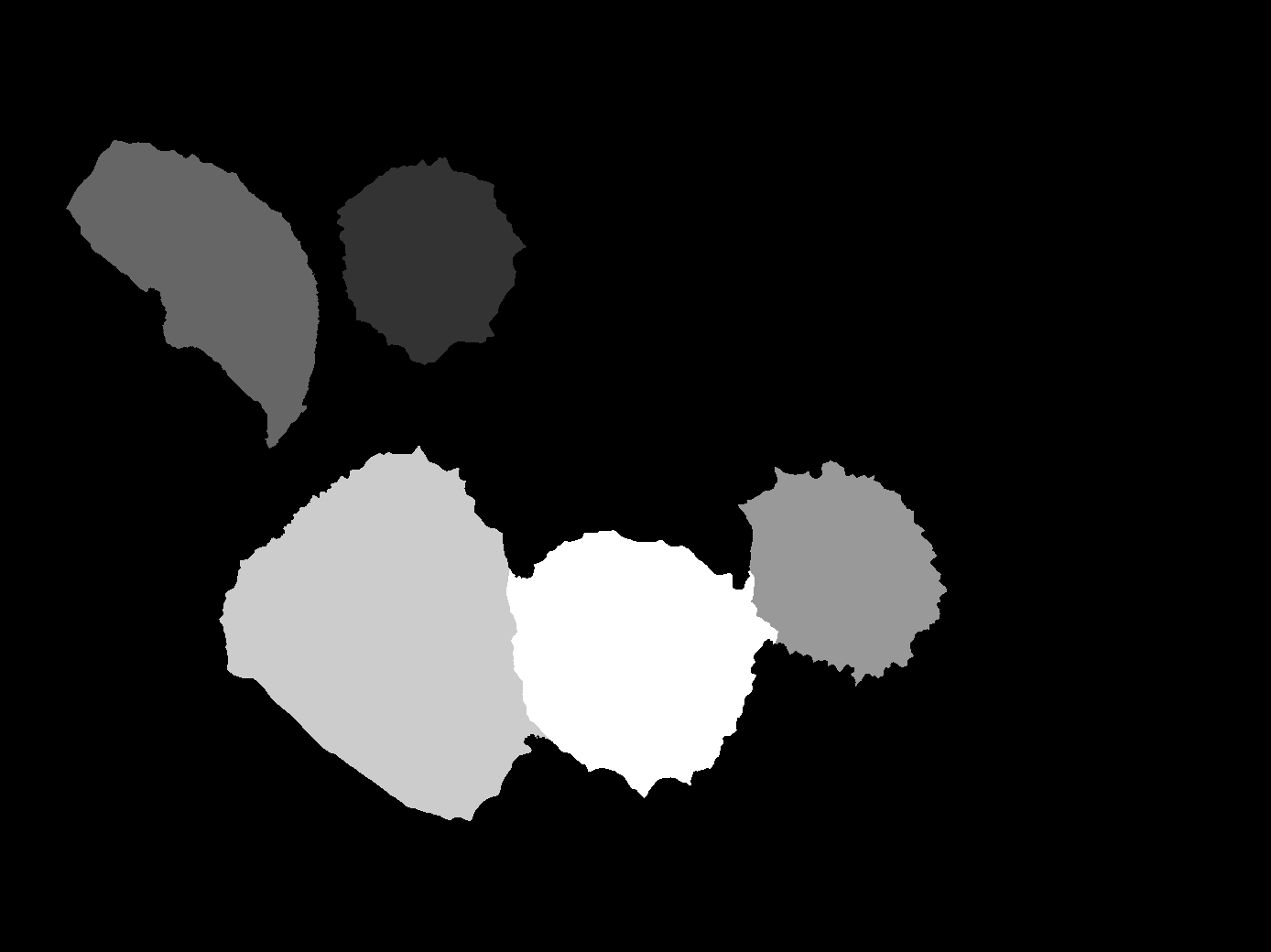

Supplement: S1 File — This file contains all scripts (CellProfiler v2.1.1 and MATLAB2016a) and data necessary to reproduce the information shown in Fig 3. (ZIP) [file pone.0180810.s001.zip › vitaminD_eColi_reproducibleResearchArchive/Results2016/C_13_c2_seg.tif]

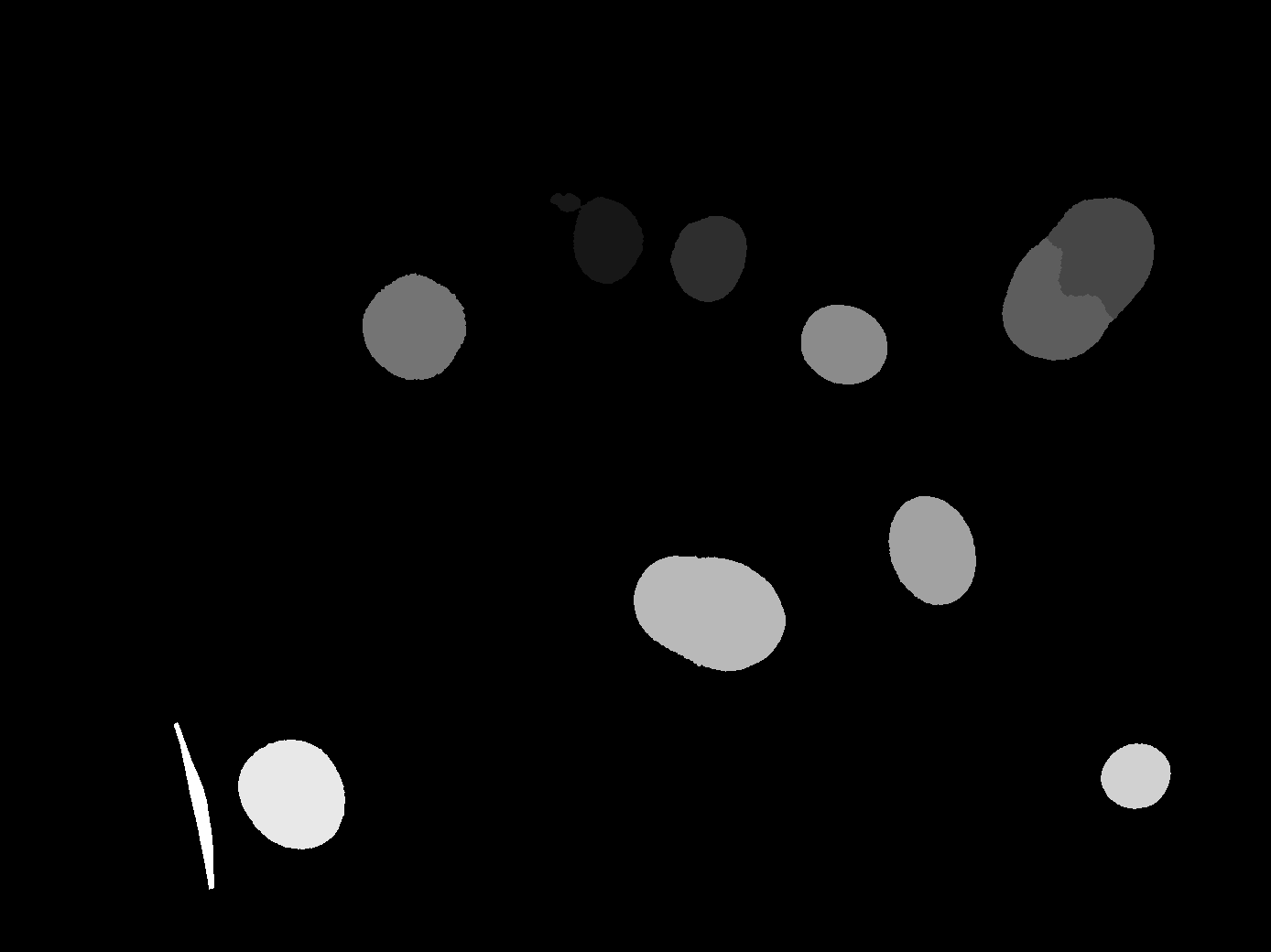

Supplement: S1 File — This file contains all scripts (CellProfiler v2.1.1 and MATLAB2016a) and data necessary to reproduce the information shown in Fig 3. (ZIP) [file pone.0180810.s001.zip › vitaminD_eColi_reproducibleResearchArchive/Results2016/C_14_c0_seg.tif]

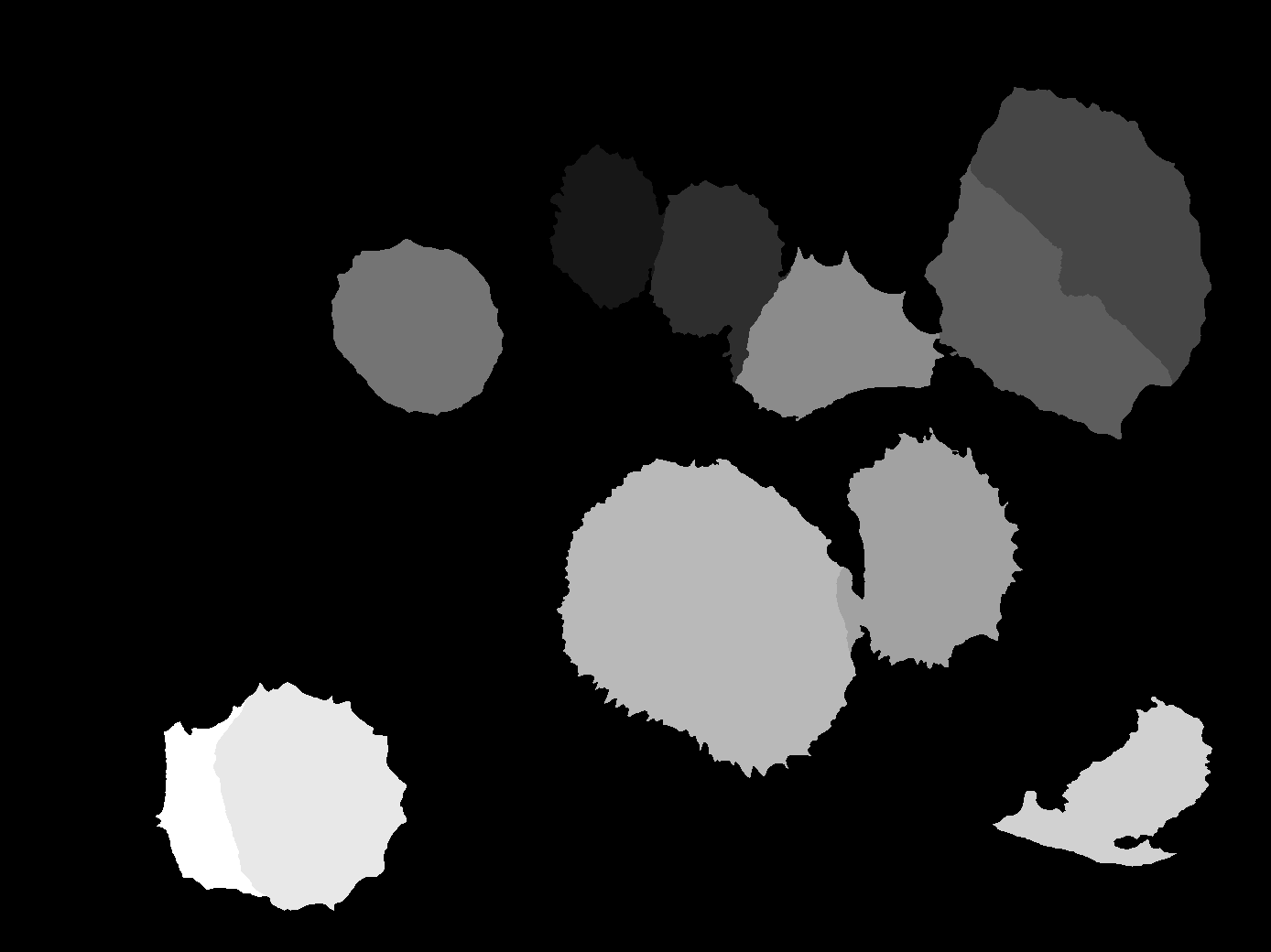

Supplement: S1 File — This file contains all scripts (CellProfiler v2.1.1 and MATLAB2016a) and data necessary to reproduce the information shown in Fig 3. (ZIP) [file pone.0180810.s001.zip › vitaminD_eColi_reproducibleResearchArchive/Results2016/C_14_c2_seg.tif]

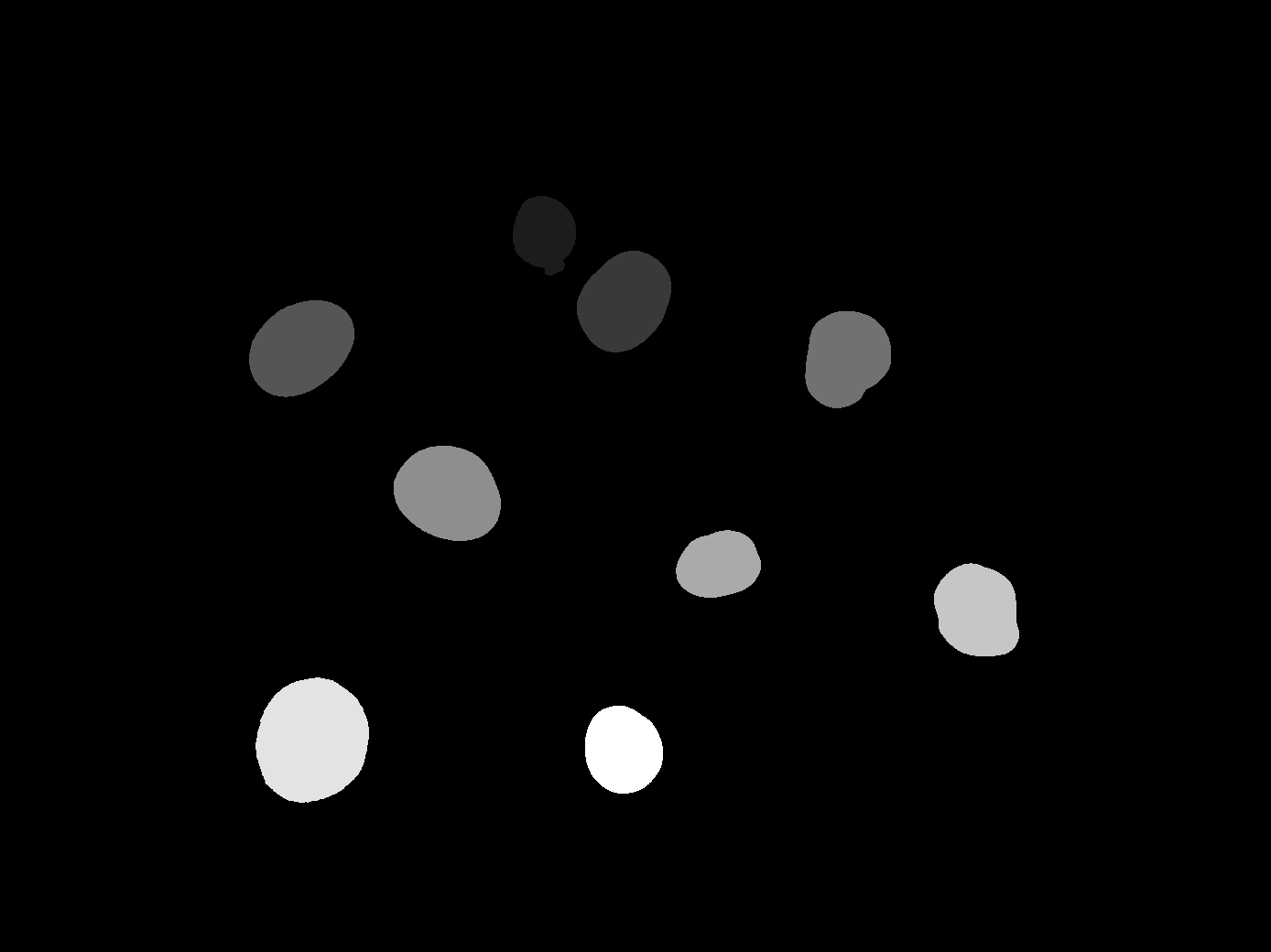

Supplement: S1 File — This file contains all scripts (CellProfiler v2.1.1 and MATLAB2016a) and data necessary to reproduce the information shown in Fig 3. (ZIP) [file pone.0180810.s001.zip › vitaminD_eColi_reproducibleResearchArchive/Results2016/C_15_c0_seg.tif]

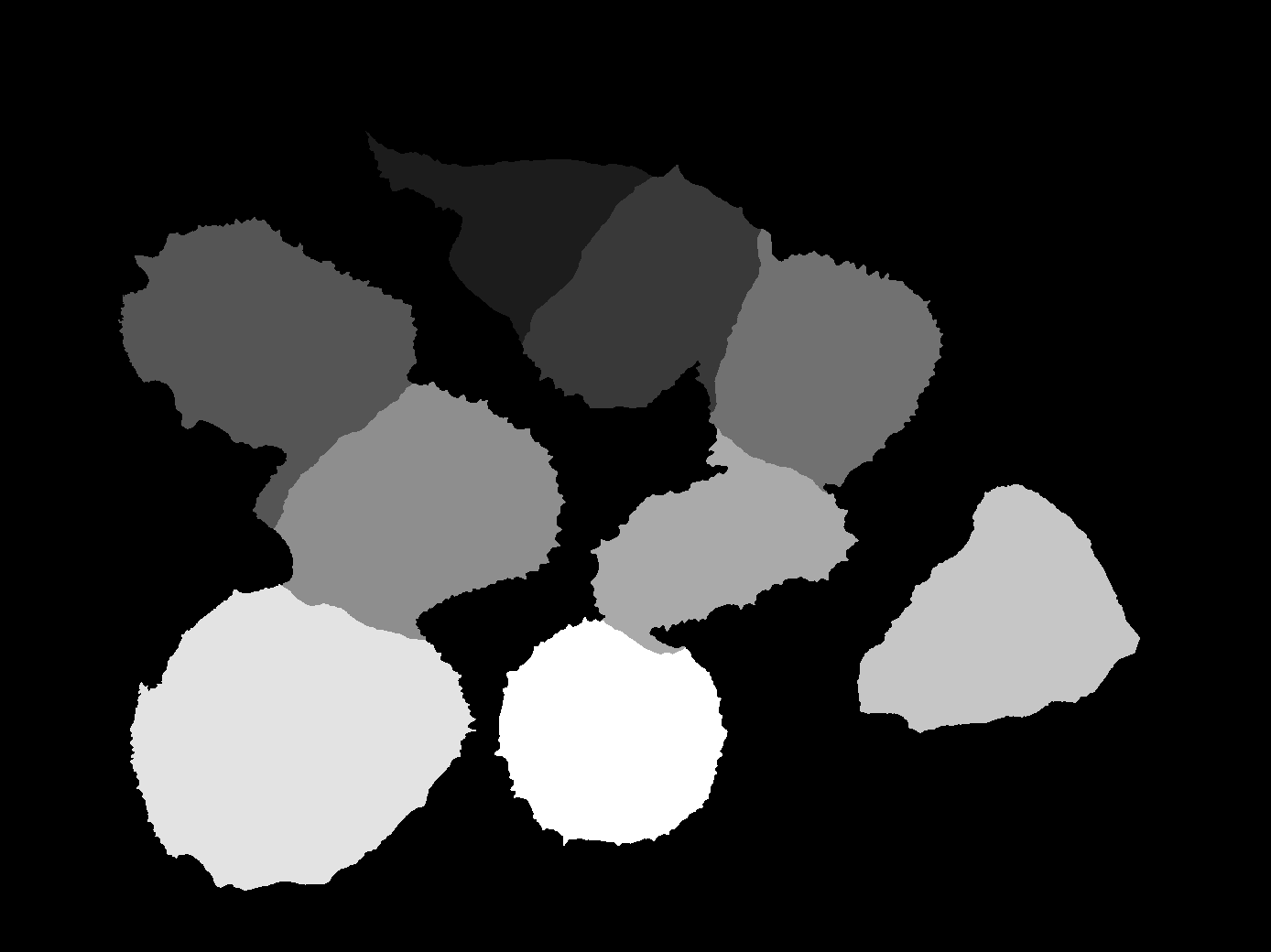

Supplement: S1 File — This file contains all scripts (CellProfiler v2.1.1 and MATLAB2016a) and data necessary to reproduce the information shown in Fig 3. (ZIP) [file pone.0180810.s001.zip › vitaminD_eColi_reproducibleResearchArchive/Results2016/C_15_c2_seg.tif]

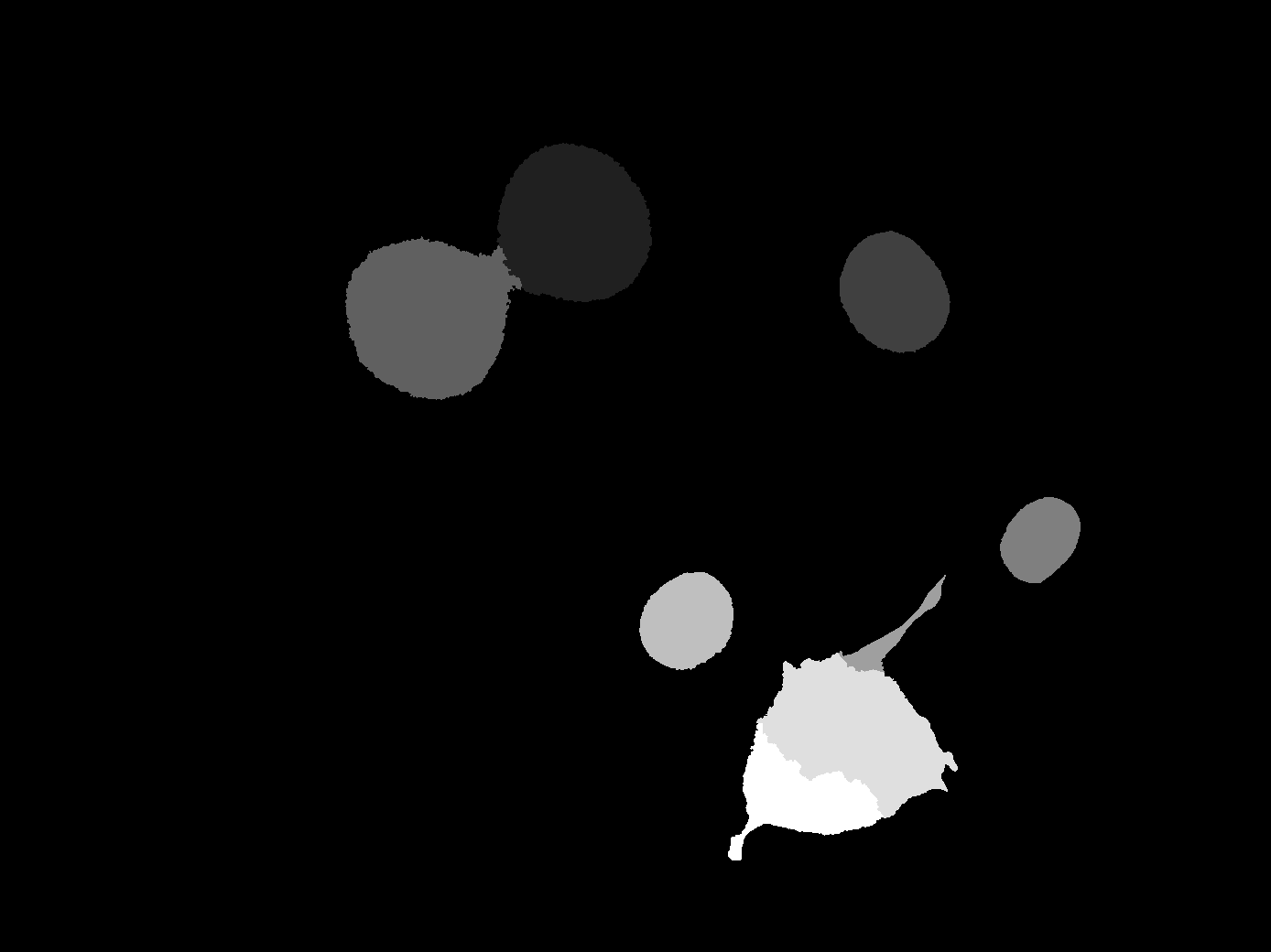

Supplement: S1 File — This file contains all scripts (CellProfiler v2.1.1 and MATLAB2016a) and data necessary to reproduce the information shown in Fig 3. (ZIP) [file pone.0180810.s001.zip › vitaminD_eColi_reproducibleResearchArchive/Results2016/C_16_c0_seg.tif]

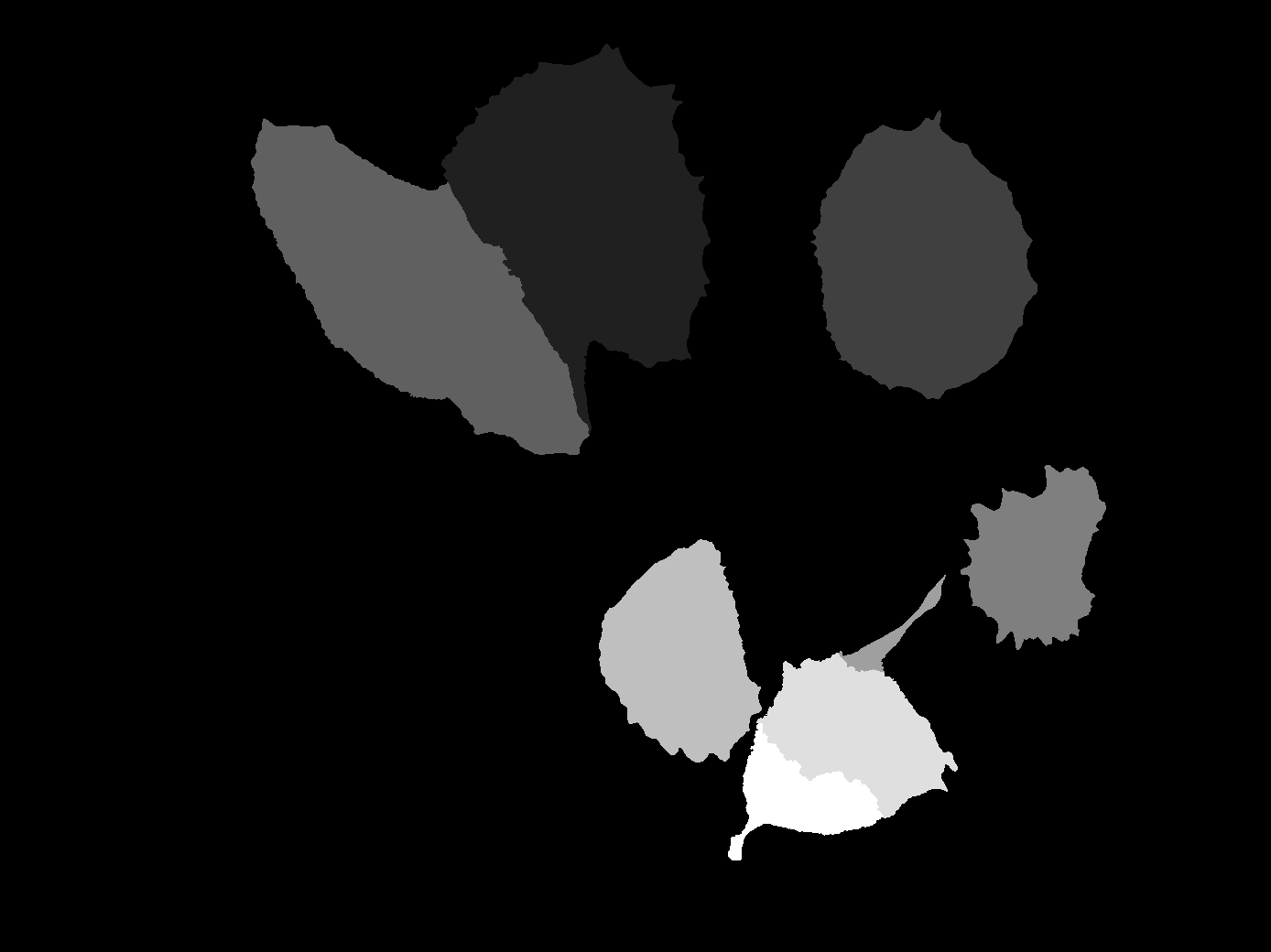

Supplement: S1 File — This file contains all scripts (CellProfiler v2.1.1 and MATLAB2016a) and data necessary to reproduce the information shown in Fig 3. (ZIP) [file pone.0180810.s001.zip › vitaminD_eColi_reproducibleResearchArchive/Results2016/C_16_c2_seg.tif]

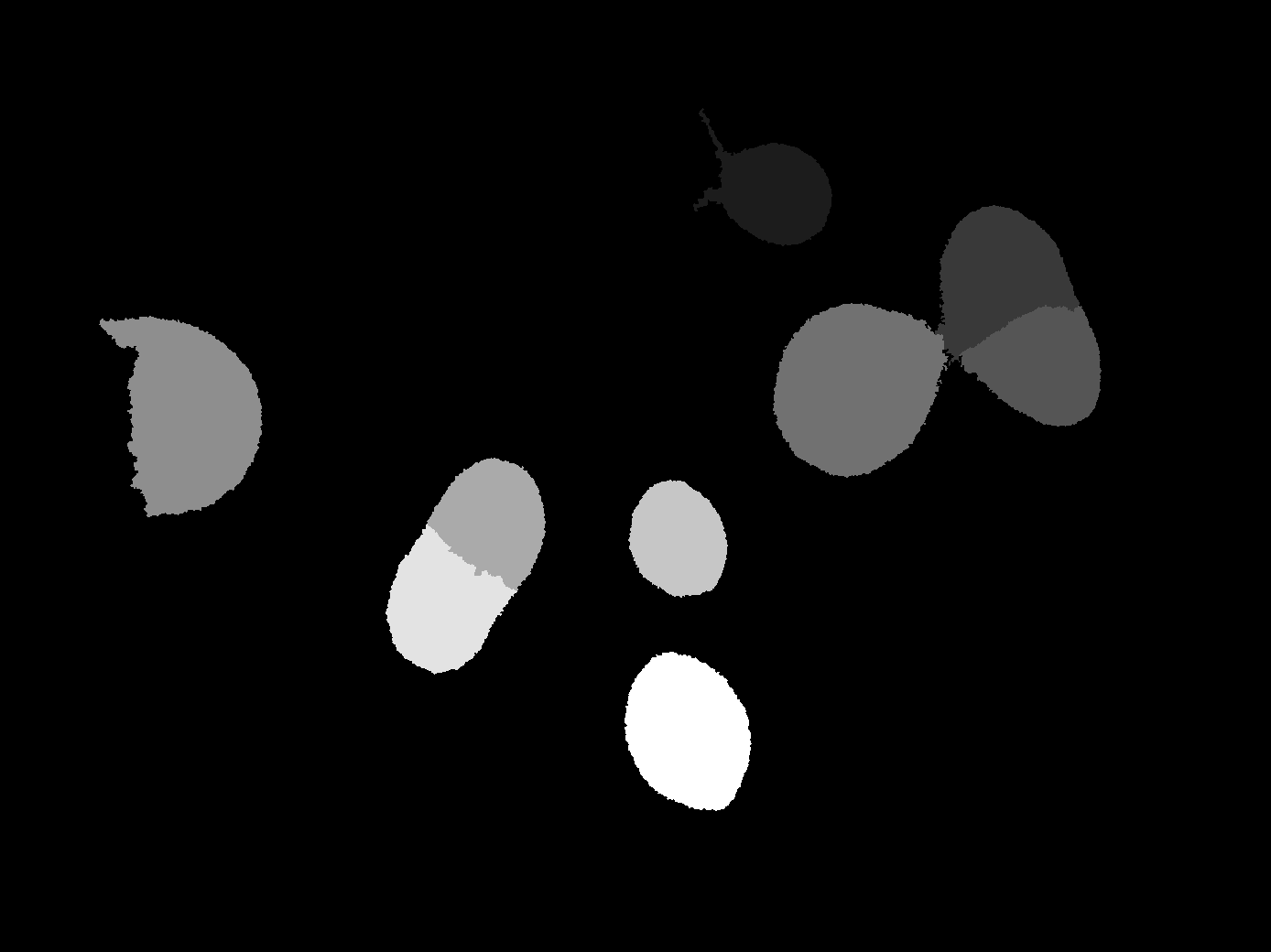

Supplement: S1 File — This file contains all scripts (CellProfiler v2.1.1 and MATLAB2016a) and data necessary to reproduce the information shown in Fig 3. (ZIP) [file pone.0180810.s001.zip › vitaminD_eColi_reproducibleResearchArchive/Results2016/C_17_c0_seg.tif]

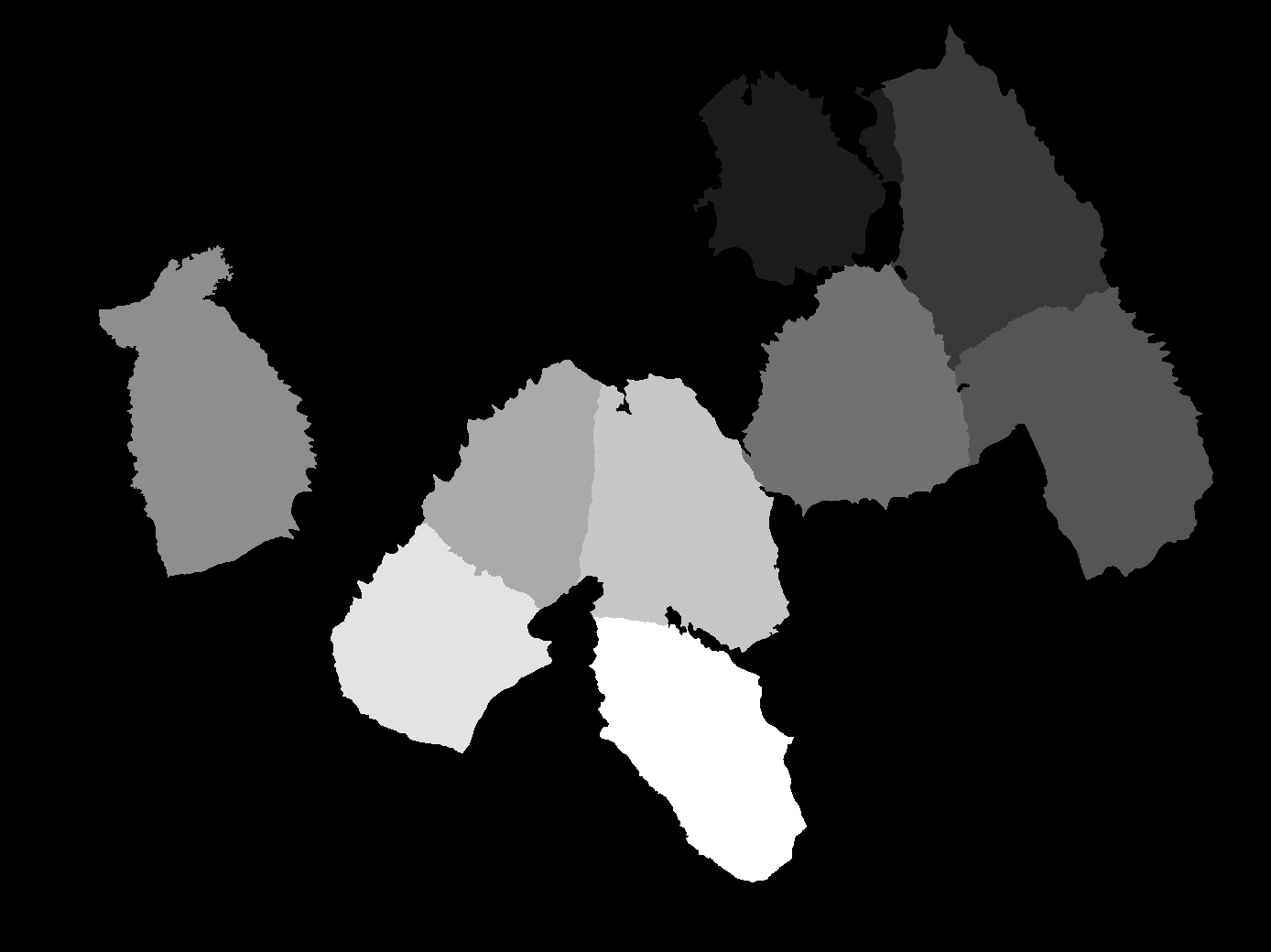

Supplement: S1 File — This file contains all scripts (CellProfiler v2.1.1 and MATLAB2016a) and data necessary to reproduce the information shown in Fig 3. (ZIP) [file pone.0180810.s001.zip › vitaminD_eColi_reproducibleResearchArchive/Results2016/C_17_c2_seg.tif]

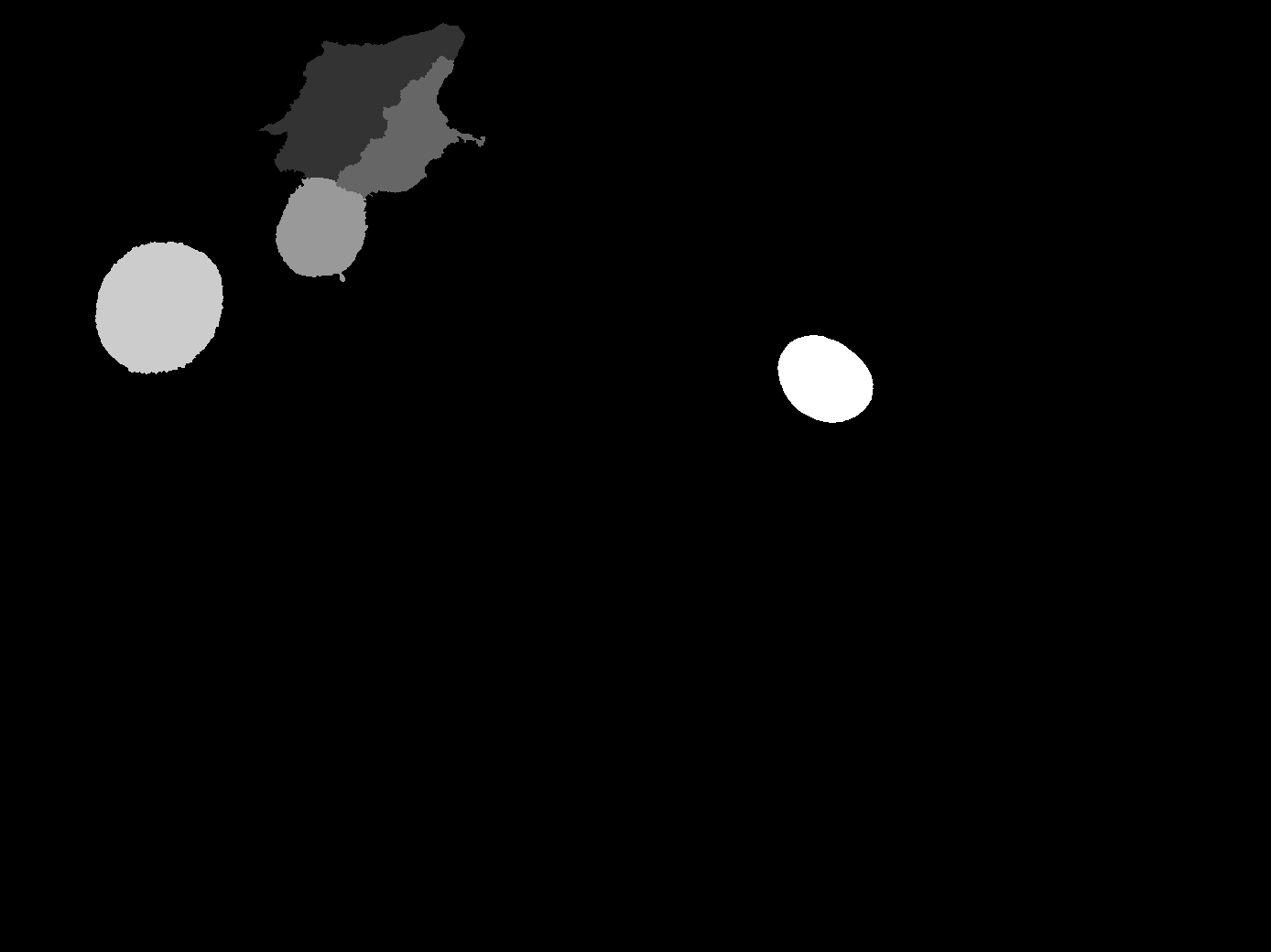

Supplement: S1 File — This file contains all scripts (CellProfiler v2.1.1 and MATLAB2016a) and data necessary to reproduce the information shown in Fig 3. (ZIP) [file pone.0180810.s001.zip › vitaminD_eColi_reproducibleResearchArchive/Results2016/C_18_c0_seg.tif]

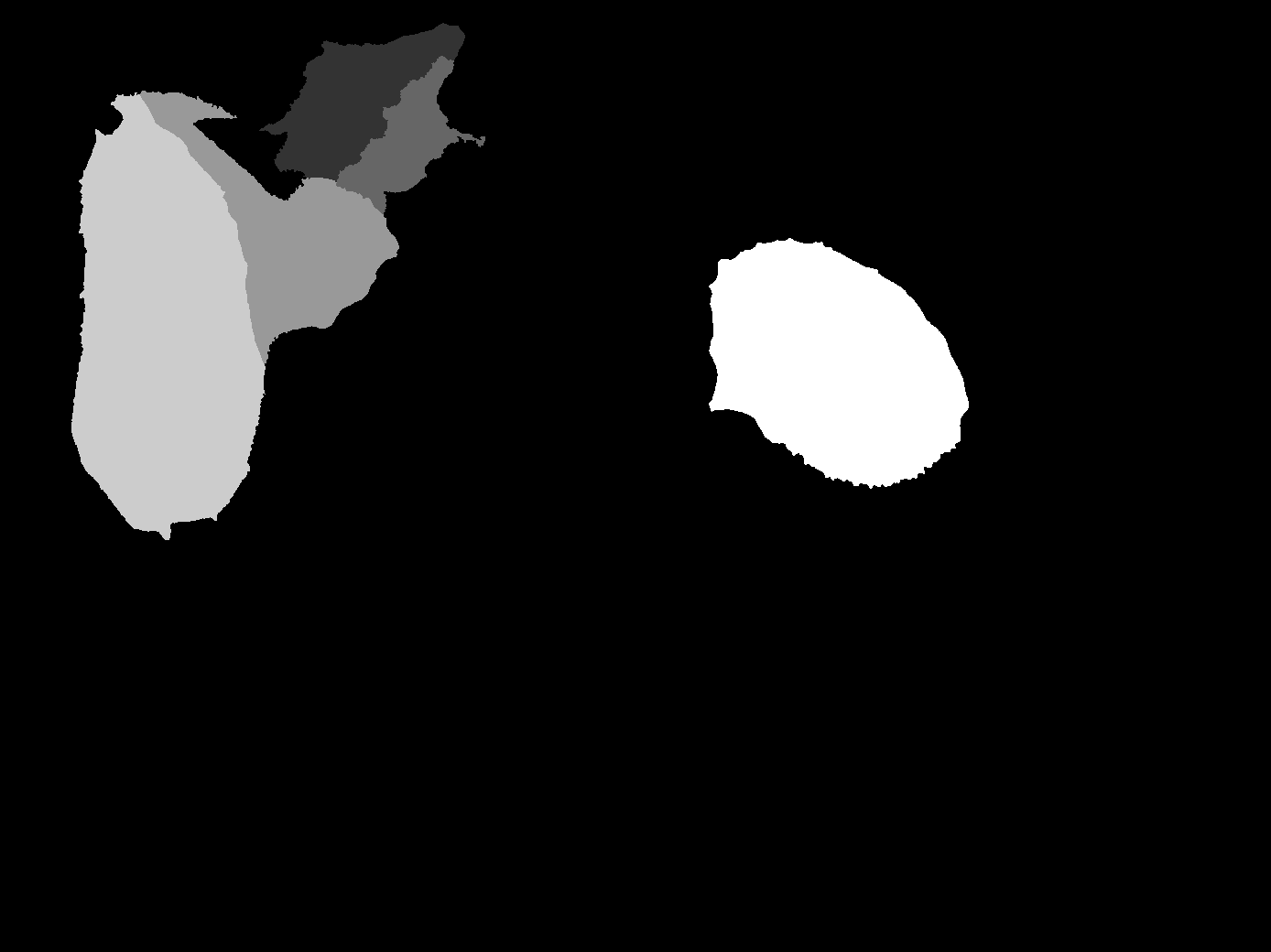

Supplement: S1 File — This file contains all scripts (CellProfiler v2.1.1 and MATLAB2016a) and data necessary to reproduce the information shown in Fig 3. (ZIP) [file pone.0180810.s001.zip › vitaminD_eColi_reproducibleResearchArchive/Results2016/C_18_c2_seg.tif]

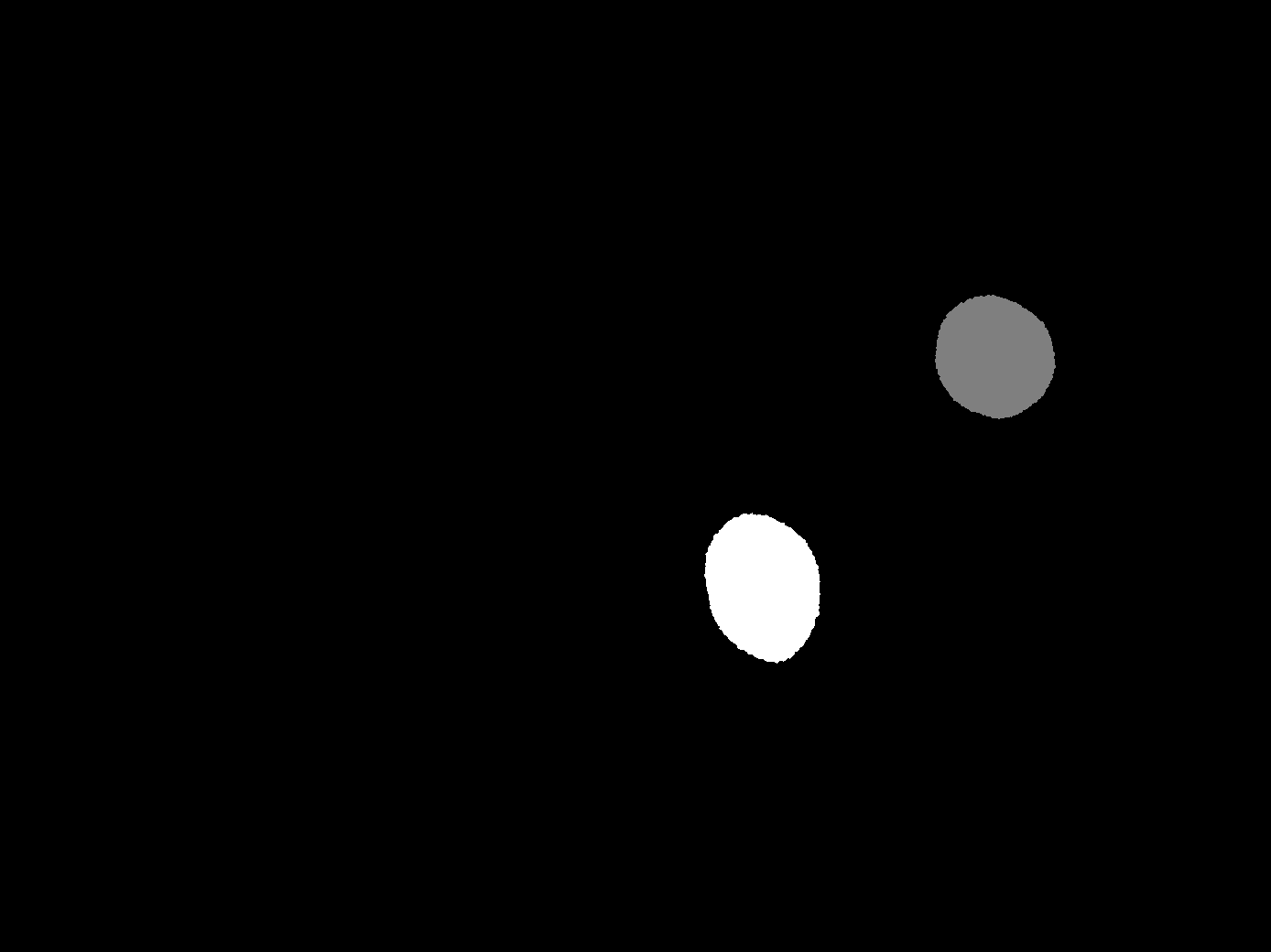

Supplement: S1 File — This file contains all scripts (CellProfiler v2.1.1 and MATLAB2016a) and data necessary to reproduce the information shown in Fig 3. (ZIP) [file pone.0180810.s001.zip › vitaminD_eColi_reproducibleResearchArchive/Results2016/C_19_c0_seg.tif]

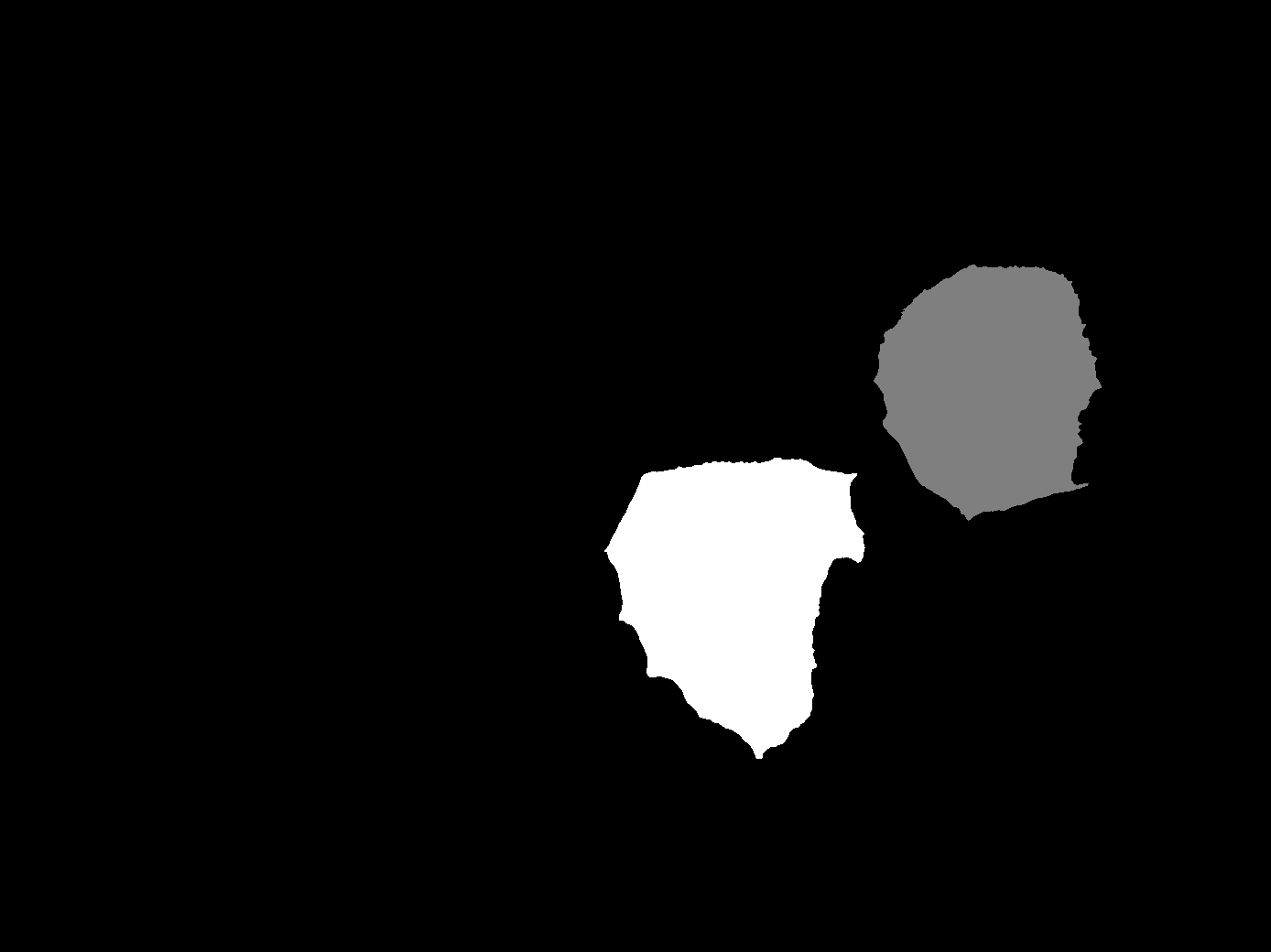

Supplement: S1 File — This file contains all scripts (CellProfiler v2.1.1 and MATLAB2016a) and data necessary to reproduce the information shown in Fig 3. (ZIP) [file pone.0180810.s001.zip › vitaminD_eColi_reproducibleResearchArchive/Results2016/C_19_c2_seg.tif]

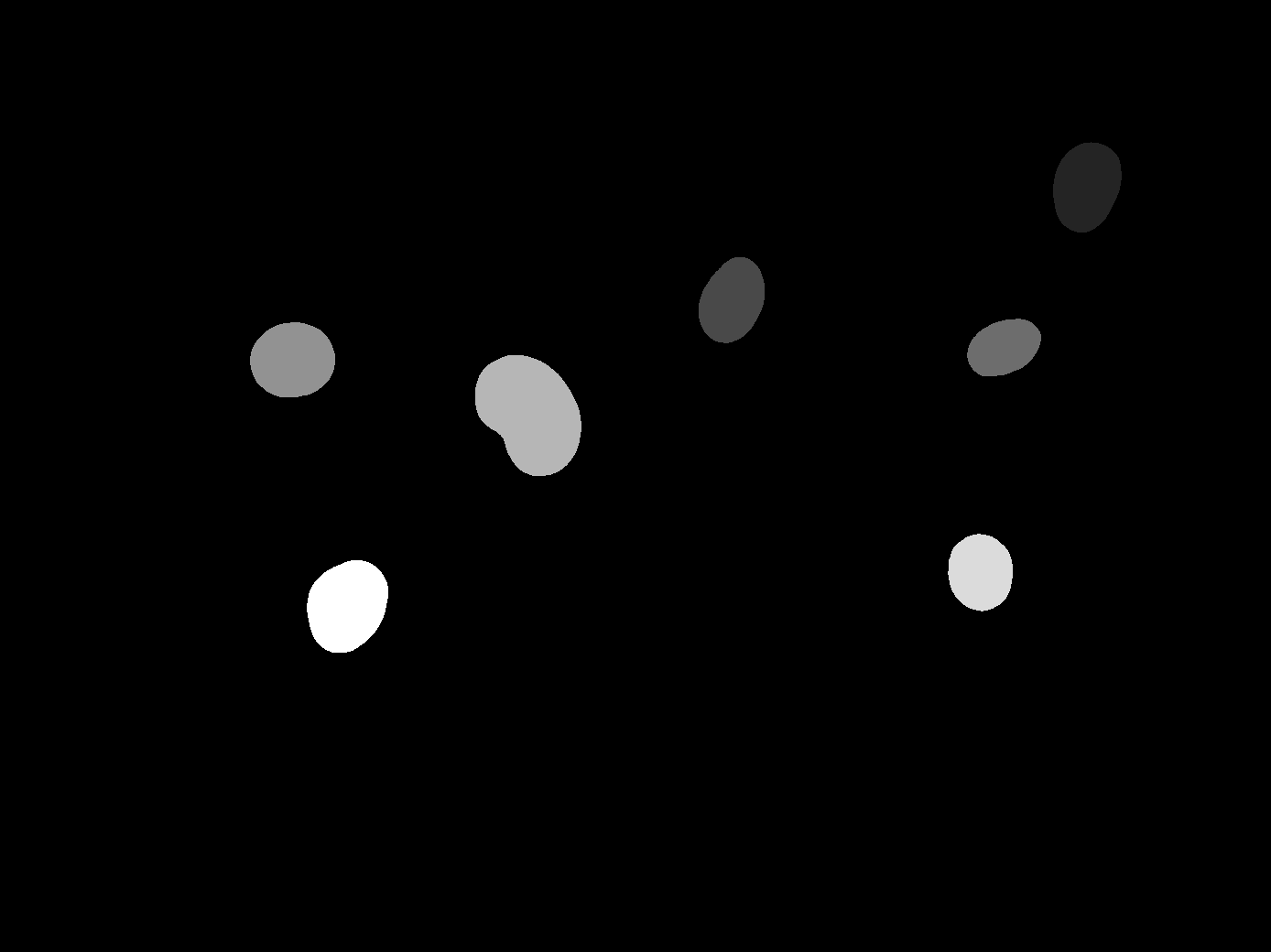

Supplement: S1 File — This file contains all scripts (CellProfiler v2.1.1 and MATLAB2016a) and data necessary to reproduce the information shown in Fig 3. (ZIP) [file pone.0180810.s001.zip › vitaminD_eColi_reproducibleResearchArchive/Results2016/C_1_c0_seg.tif]

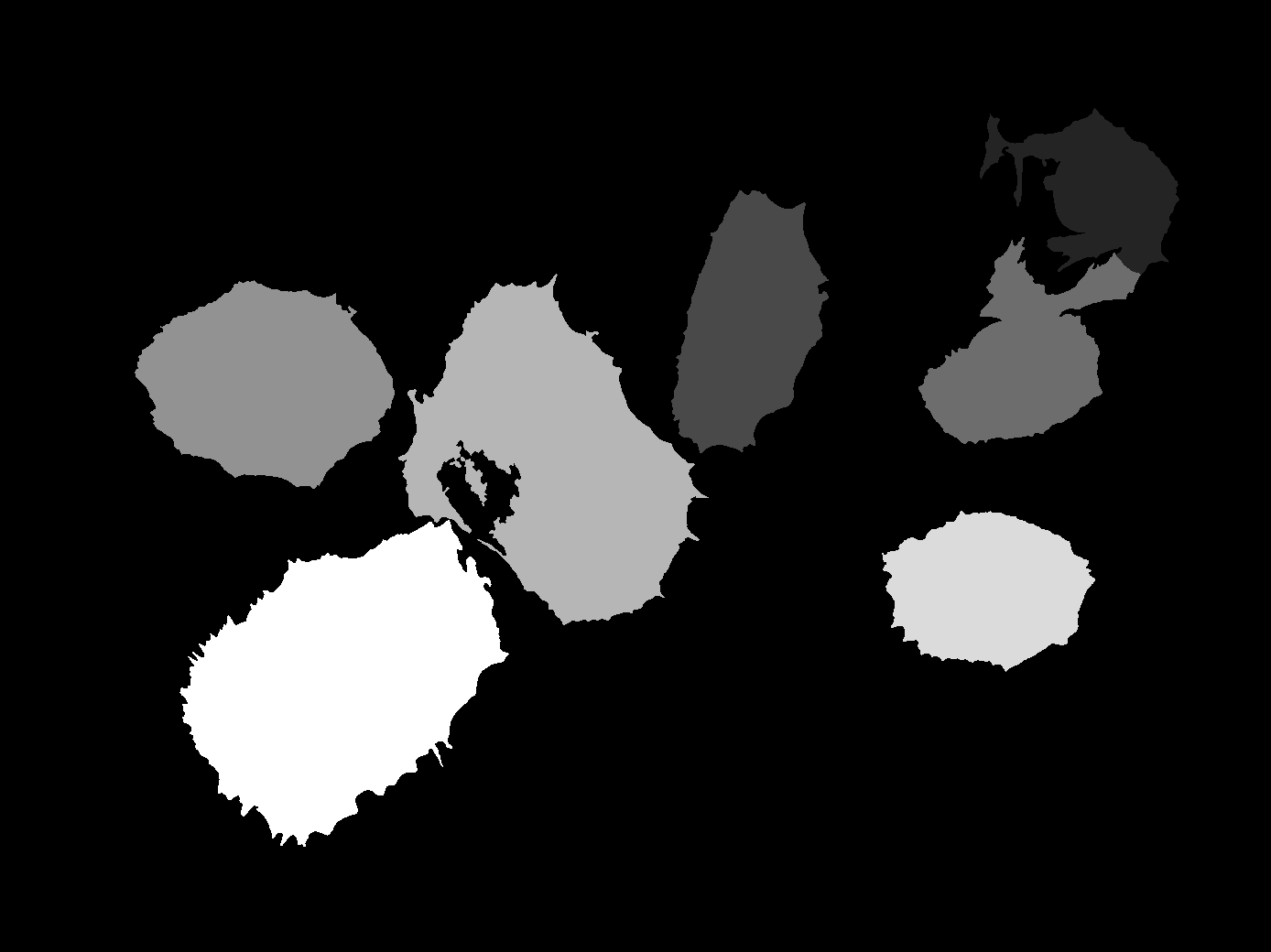

Supplement: S1 File — This file contains all scripts (CellProfiler v2.1.1 and MATLAB2016a) and data necessary to reproduce the information shown in Fig 3. (ZIP) [file pone.0180810.s001.zip › vitaminD_eColi_reproducibleResearchArchive/Results2016/C_1_c2_seg.tif]

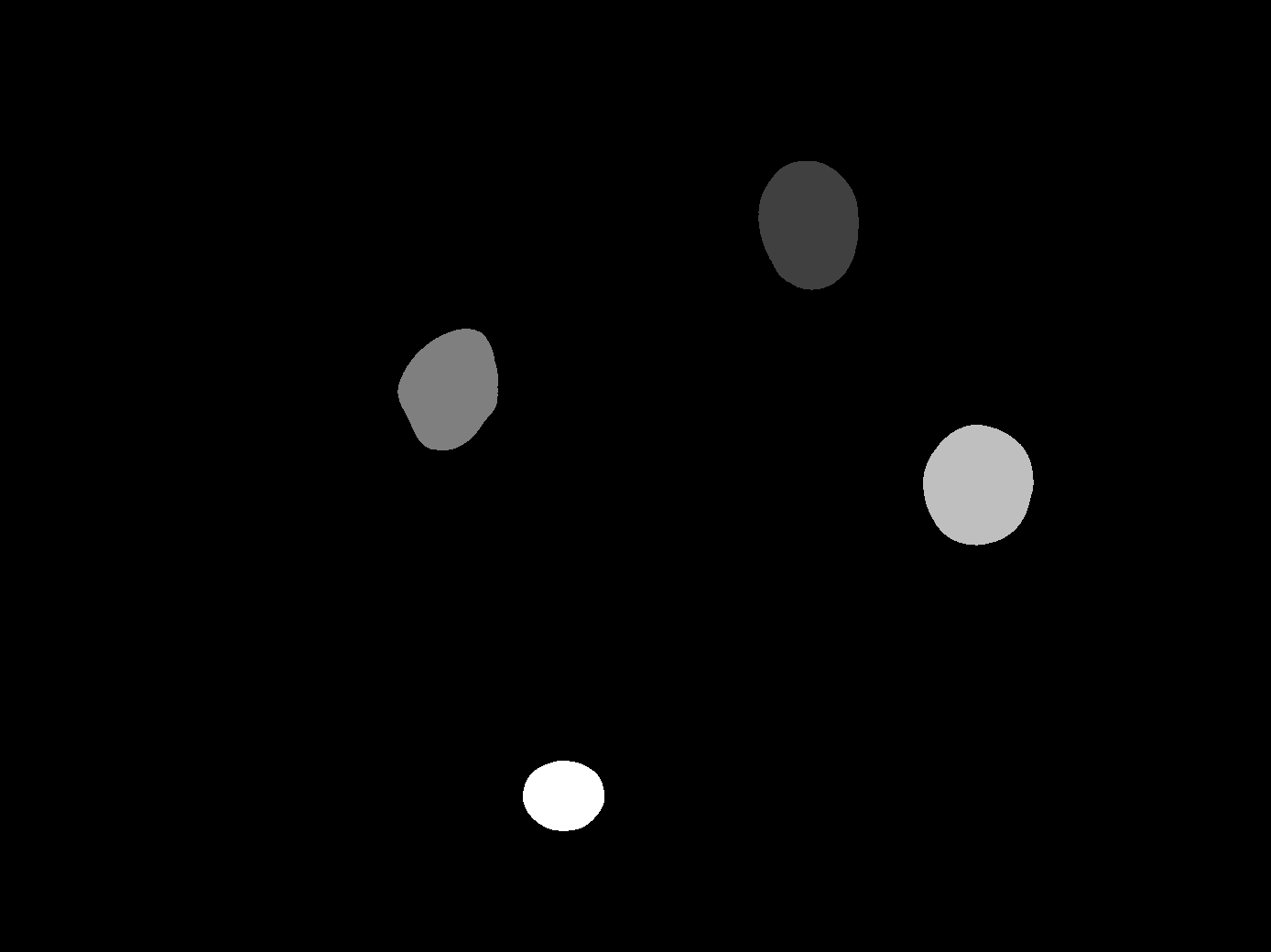

Supplement: S1 File — This file contains all scripts (CellProfiler v2.1.1 and MATLAB2016a) and data necessary to reproduce the information shown in Fig 3. (ZIP) [file pone.0180810.s001.zip › vitaminD_eColi_reproducibleResearchArchive/Results2016/C_20_c0_seg.tif]

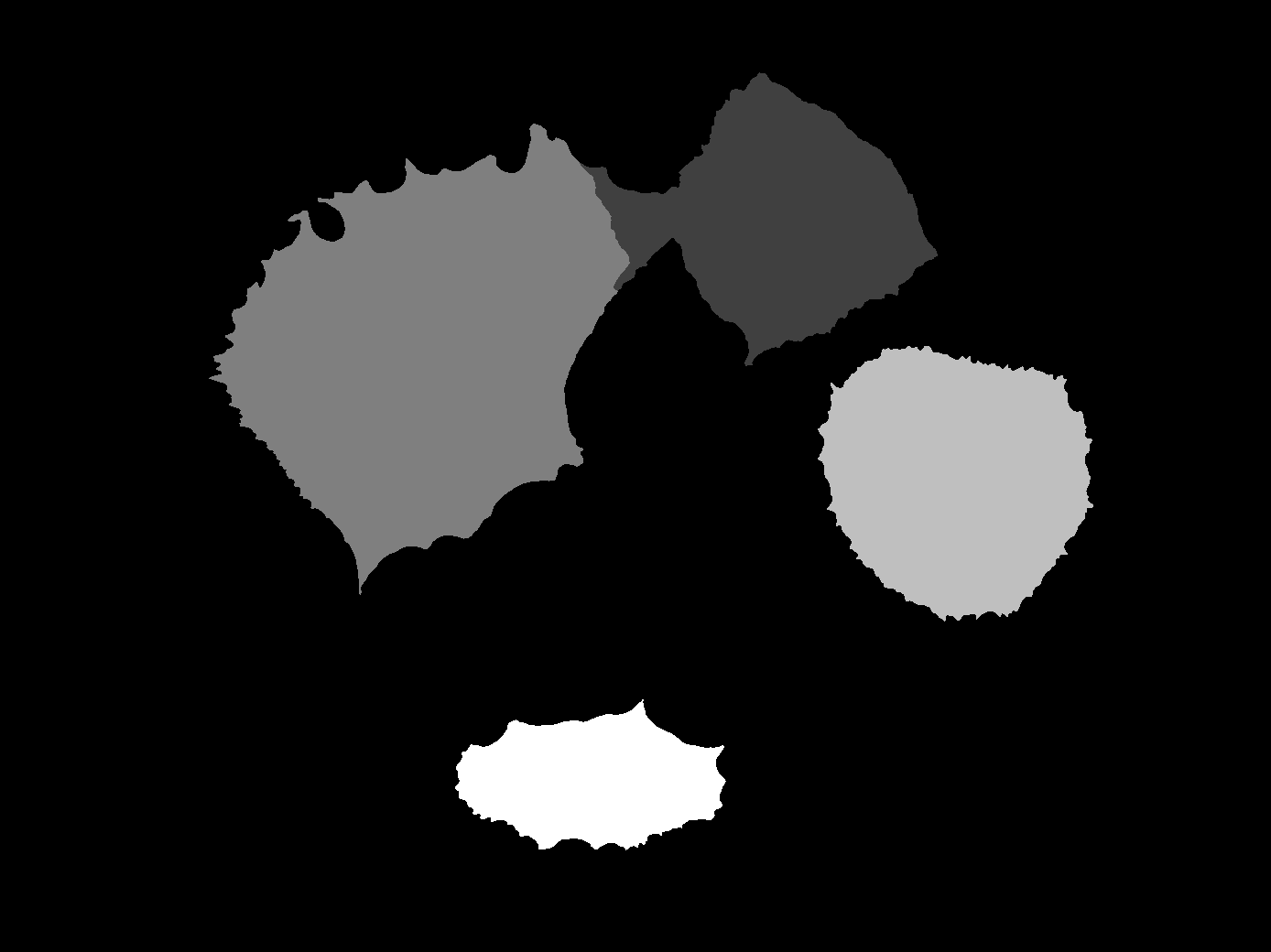

Supplement: S1 File — This file contains all scripts (CellProfiler v2.1.1 and MATLAB2016a) and data necessary to reproduce the information shown in Fig 3. (ZIP) [file pone.0180810.s001.zip › vitaminD_eColi_reproducibleResearchArchive/Results2016/C_20_c2_seg.tif]

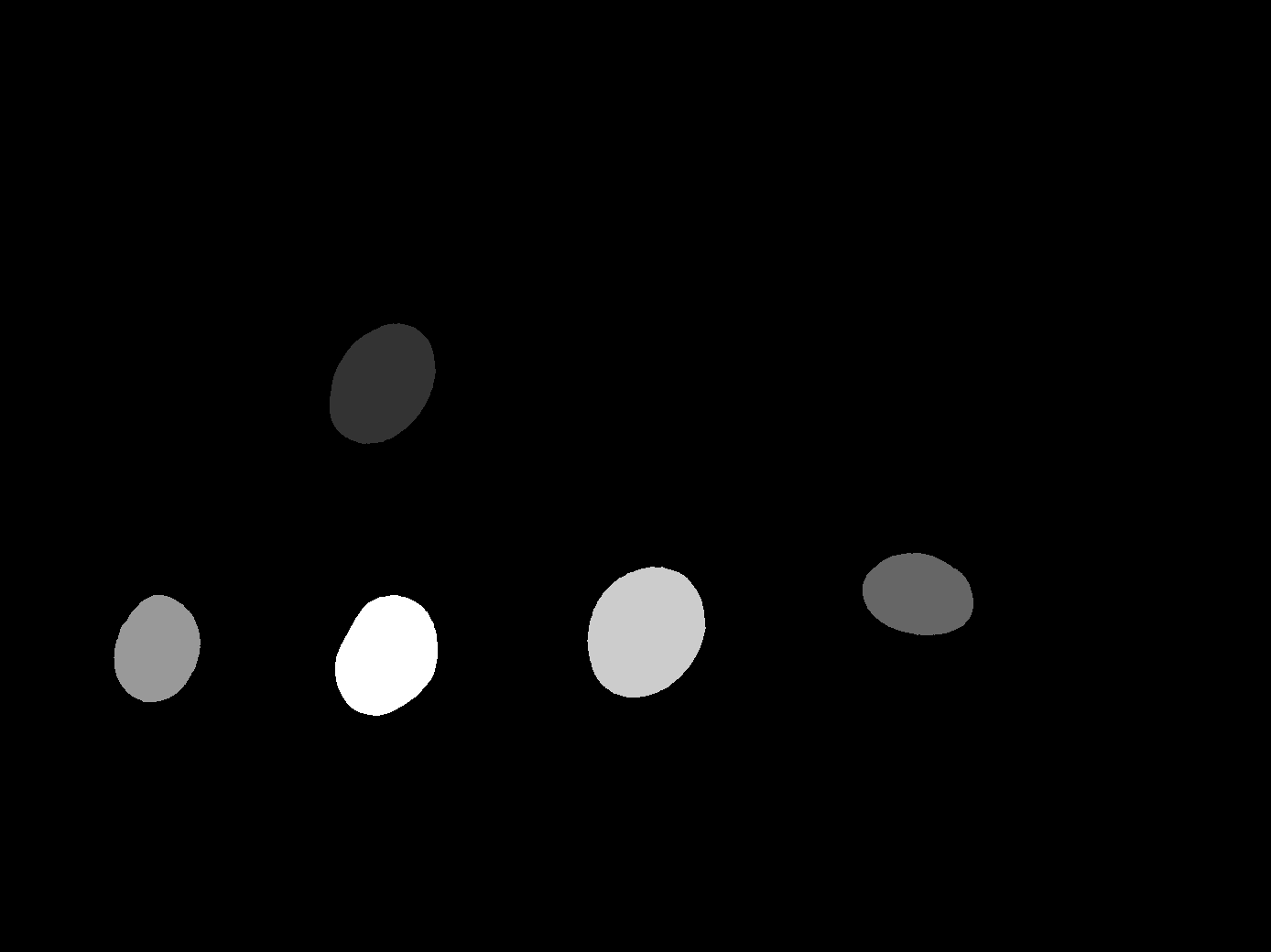

Supplement: S1 File — This file contains all scripts (CellProfiler v2.1.1 and MATLAB2016a) and data necessary to reproduce the information shown in Fig 3. (ZIP) [file pone.0180810.s001.zip › vitaminD_eColi_reproducibleResearchArchive/Results2016/C_21_c0_seg.tif]

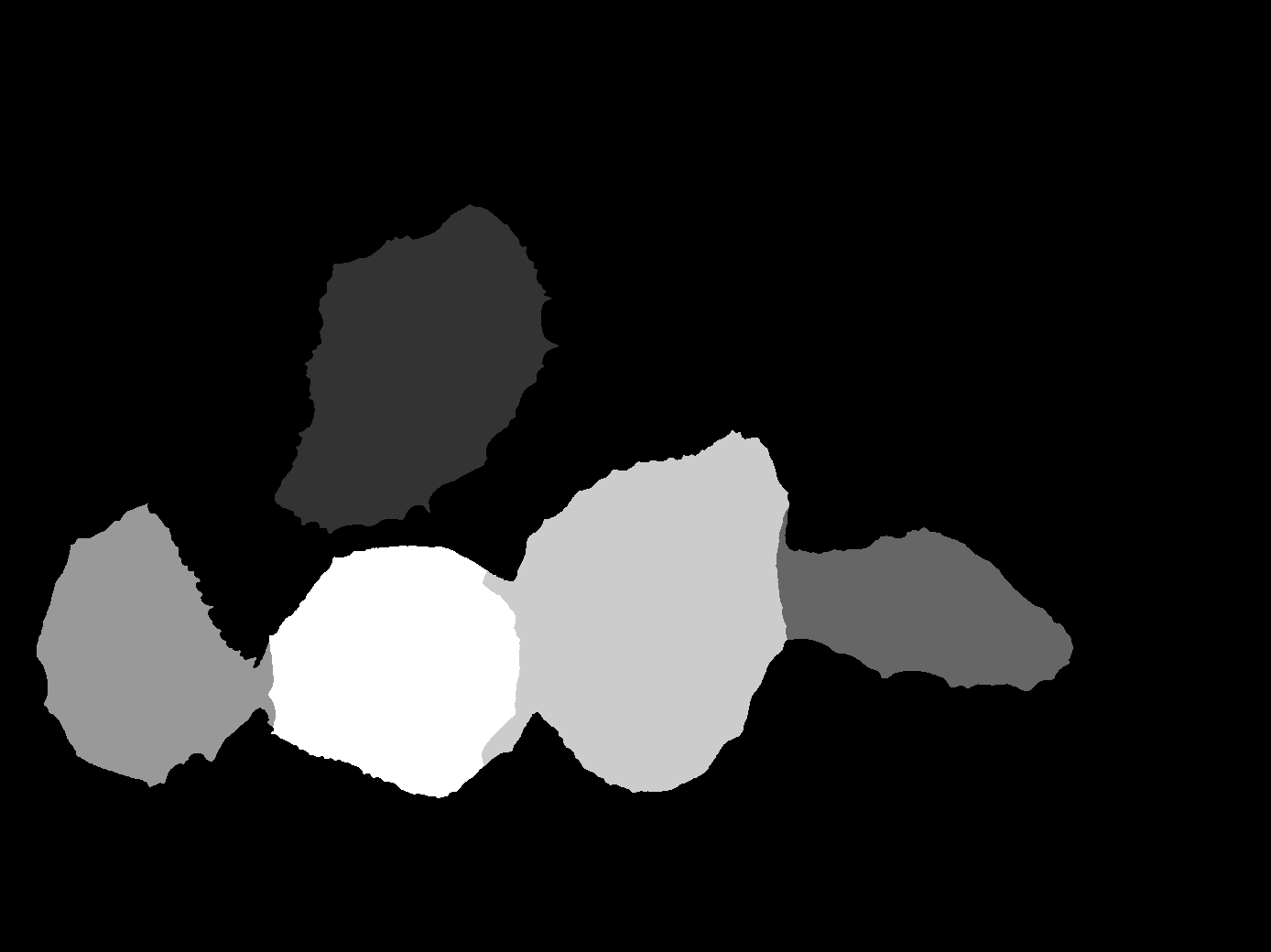

Supplement: S1 File — This file contains all scripts (CellProfiler v2.1.1 and MATLAB2016a) and data necessary to reproduce the information shown in Fig 3. (ZIP) [file pone.0180810.s001.zip › vitaminD_eColi_reproducibleResearchArchive/Results2016/C_21_c2_seg.tif]

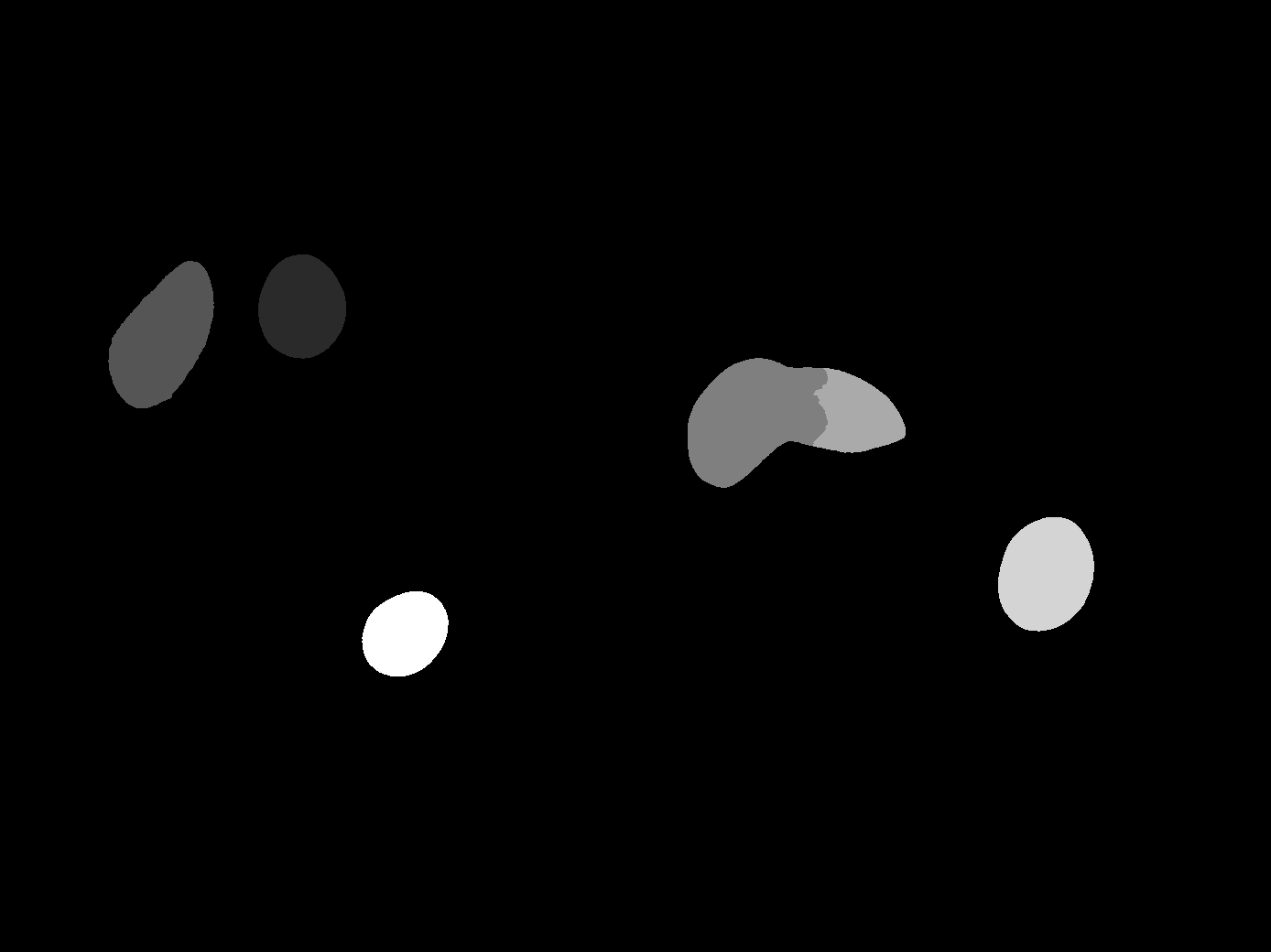

Supplement: S1 File — This file contains all scripts (CellProfiler v2.1.1 and MATLAB2016a) and data necessary to reproduce the information shown in Fig 3. (ZIP) [file pone.0180810.s001.zip › vitaminD_eColi_reproducibleResearchArchive/Results2016/C_22_c0_seg.tif]

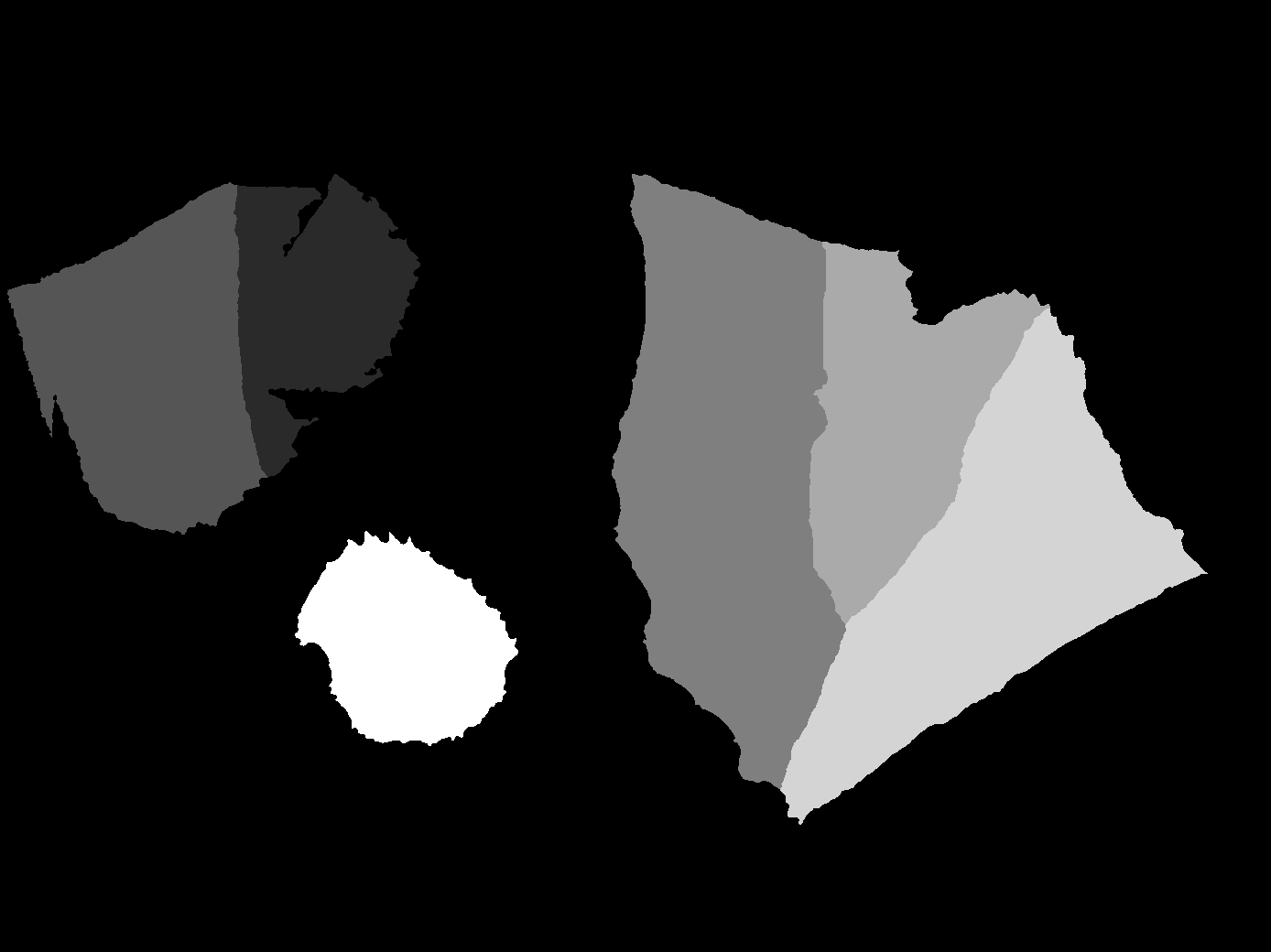

Supplement: S1 File — This file contains all scripts (CellProfiler v2.1.1 and MATLAB2016a) and data necessary to reproduce the information shown in Fig 3. (ZIP) [file pone.0180810.s001.zip › vitaminD_eColi_reproducibleResearchArchive/Results2016/C_22_c2_seg.tif]

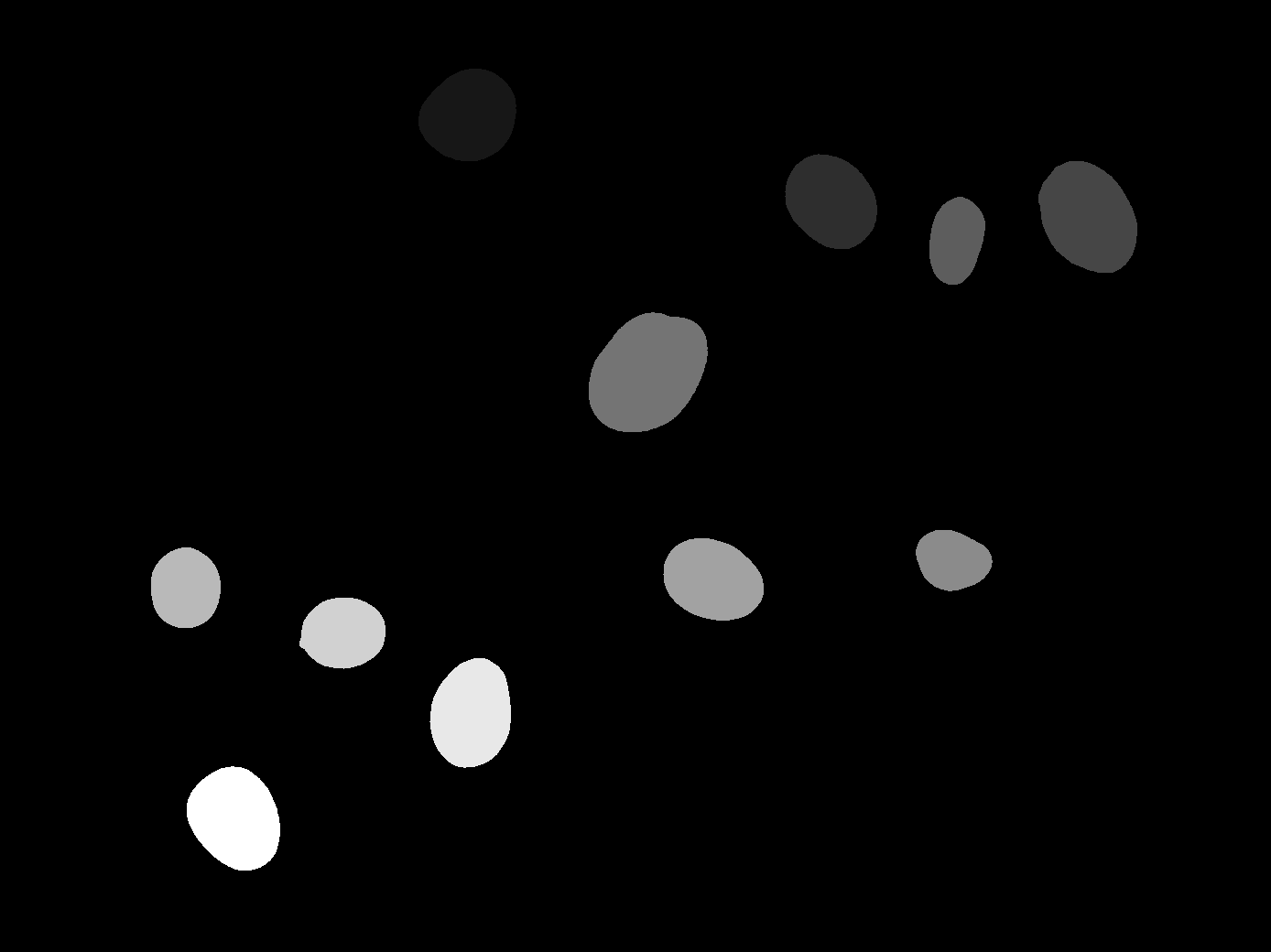

Supplement: S1 File — This file contains all scripts (CellProfiler v2.1.1 and MATLAB2016a) and data necessary to reproduce the information shown in Fig 3. (ZIP) [file pone.0180810.s001.zip › vitaminD_eColi_reproducibleResearchArchive/Results2016/C_23_c0_seg.tif]

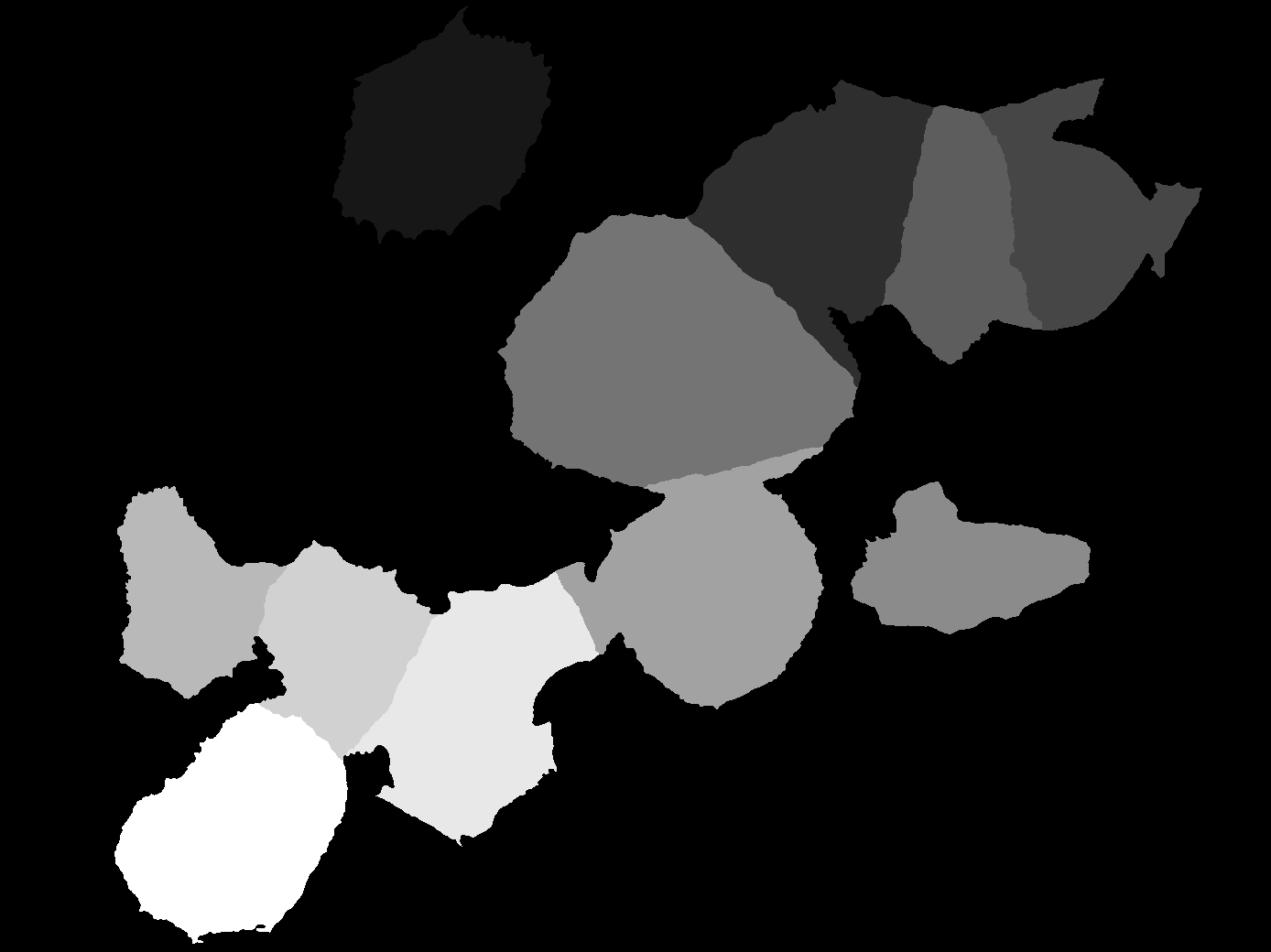

Supplement: S1 File — This file contains all scripts (CellProfiler v2.1.1 and MATLAB2016a) and data necessary to reproduce the information shown in Fig 3. (ZIP) [file pone.0180810.s001.zip › vitaminD_eColi_reproducibleResearchArchive/Results2016/C_23_c2_seg.tif]

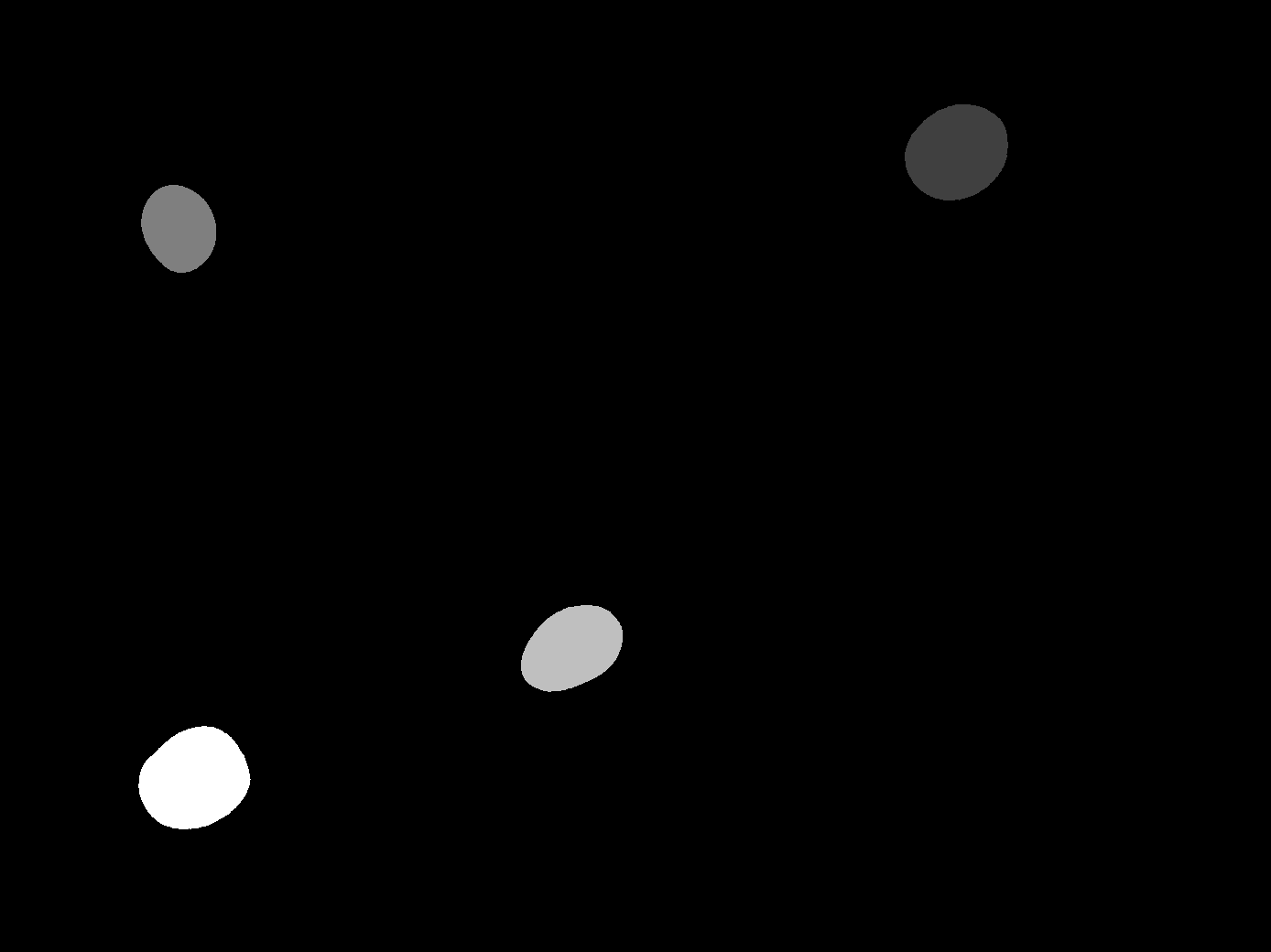

Supplement: S1 File — This file contains all scripts (CellProfiler v2.1.1 and MATLAB2016a) and data necessary to reproduce the information shown in Fig 3. (ZIP) [file pone.0180810.s001.zip › vitaminD_eColi_reproducibleResearchArchive/Results2016/C_24_c0_seg.tif]

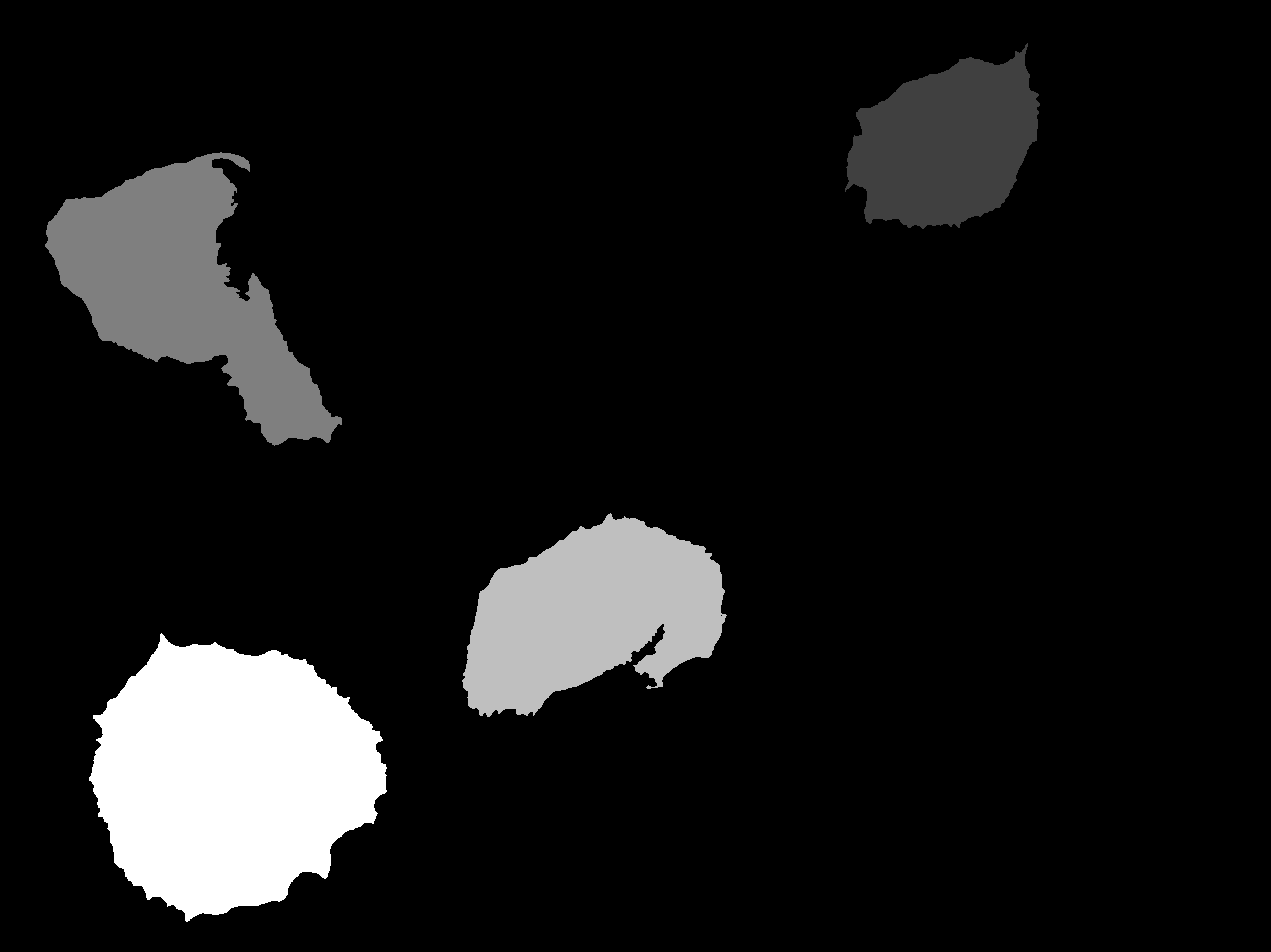

Supplement: S1 File — This file contains all scripts (CellProfiler v2.1.1 and MATLAB2016a) and data necessary to reproduce the information shown in Fig 3. (ZIP) [file pone.0180810.s001.zip › vitaminD_eColi_reproducibleResearchArchive/Results2016/C_24_c2_seg.tif]

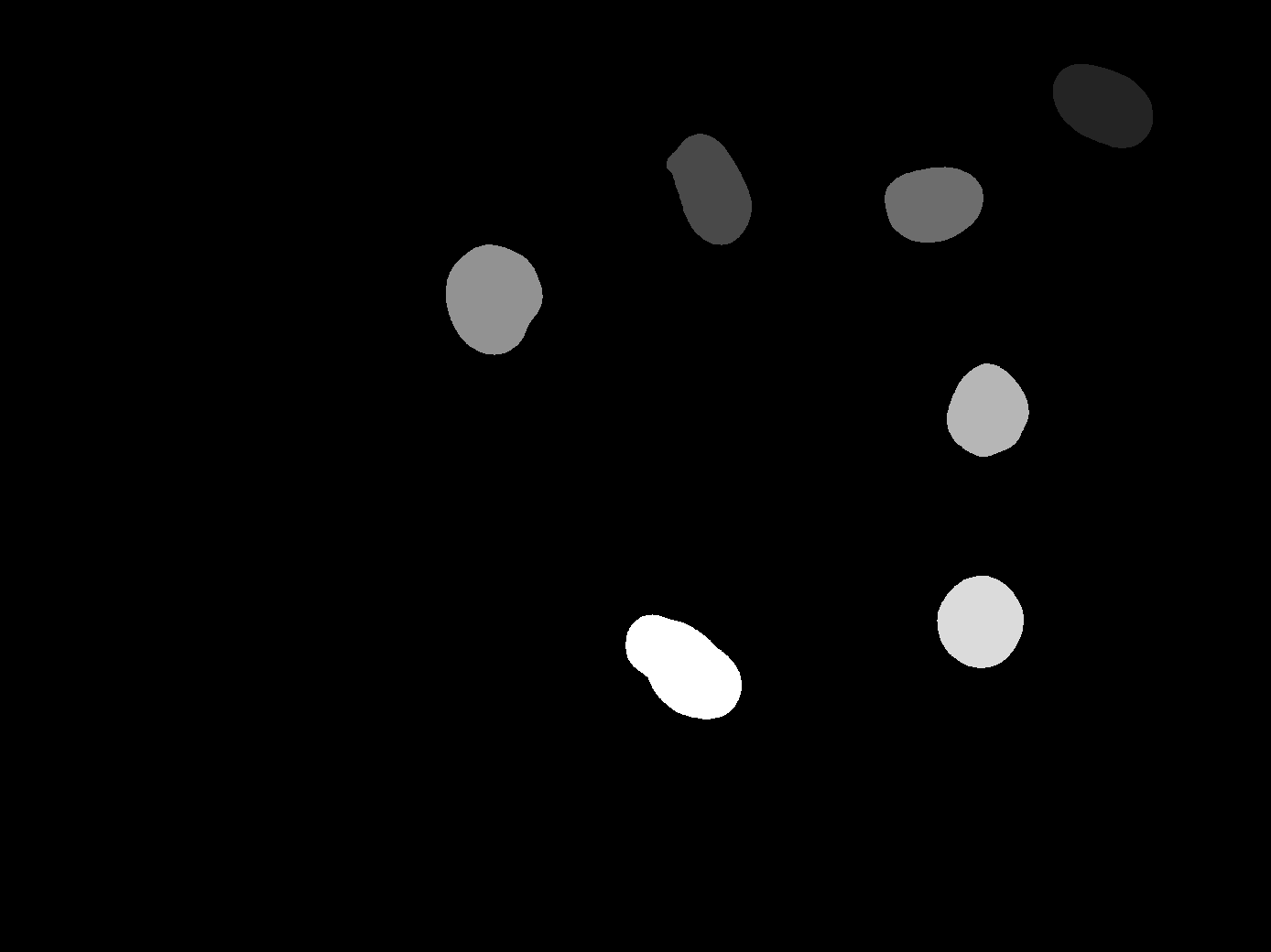

Supplement: S1 File — This file contains all scripts (CellProfiler v2.1.1 and MATLAB2016a) and data necessary to reproduce the information shown in Fig 3. (ZIP) [file pone.0180810.s001.zip › vitaminD_eColi_reproducibleResearchArchive/Results2016/C_25_c0_seg.tif]

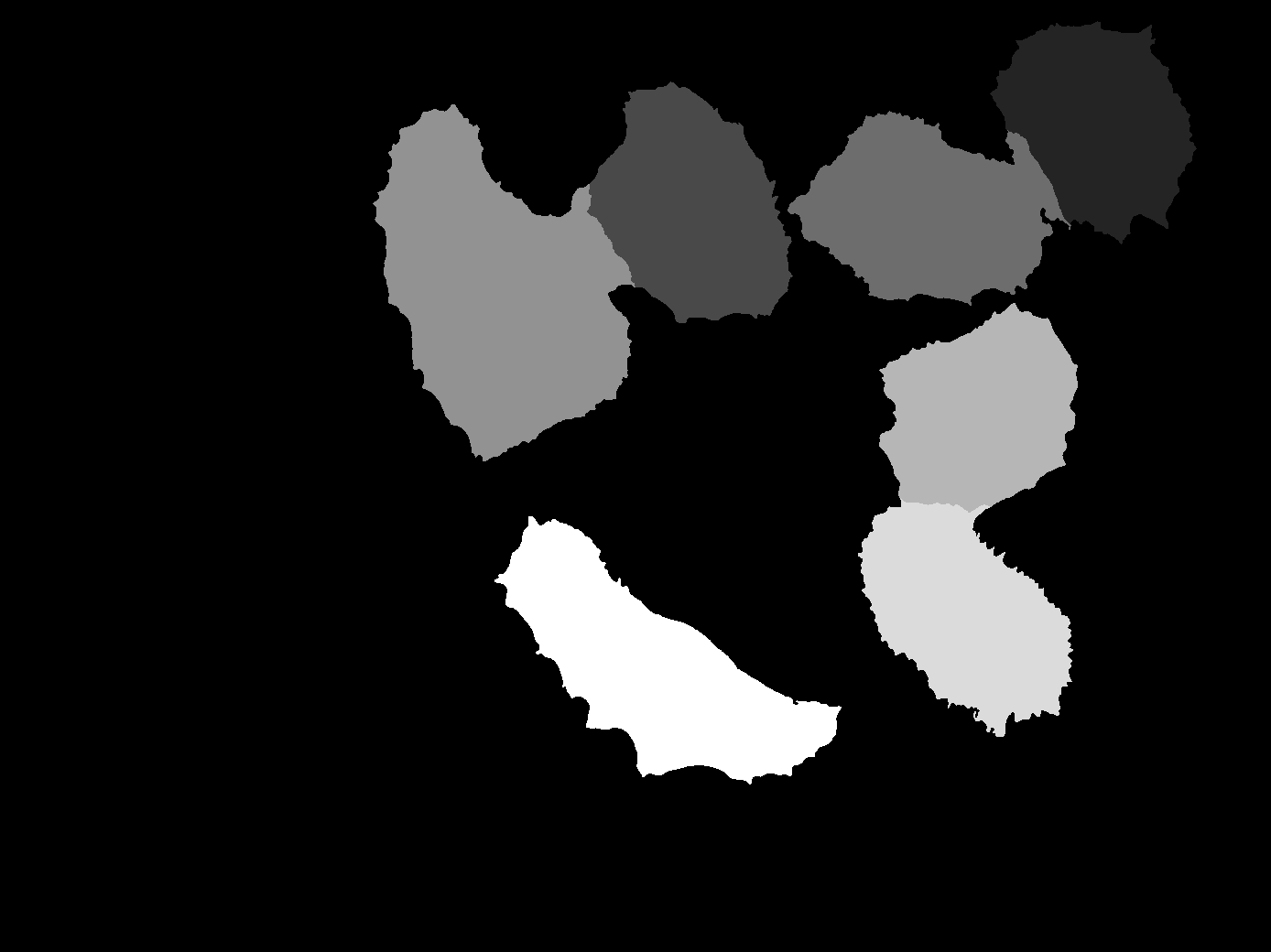

Supplement: S1 File — This file contains all scripts (CellProfiler v2.1.1 and MATLAB2016a) and data necessary to reproduce the information shown in Fig 3. (ZIP) [file pone.0180810.s001.zip › vitaminD_eColi_reproducibleResearchArchive/Results2016/C_25_c2_seg.tif]

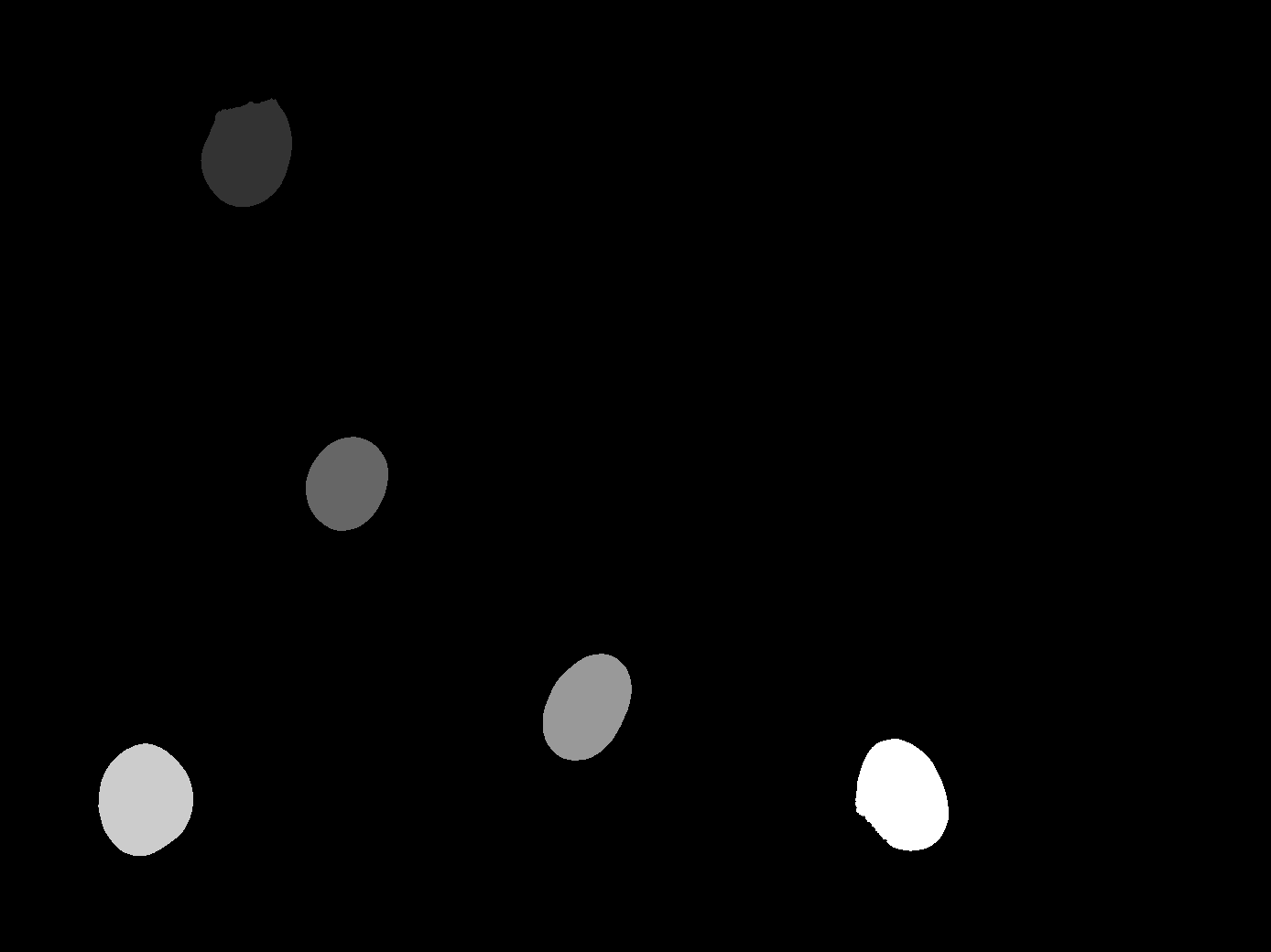

Supplement: S1 File — This file contains all scripts (CellProfiler v2.1.1 and MATLAB2016a) and data necessary to reproduce the information shown in Fig 3. (ZIP) [file pone.0180810.s001.zip › vitaminD_eColi_reproducibleResearchArchive/Results2016/C_26_c0_seg.tif]

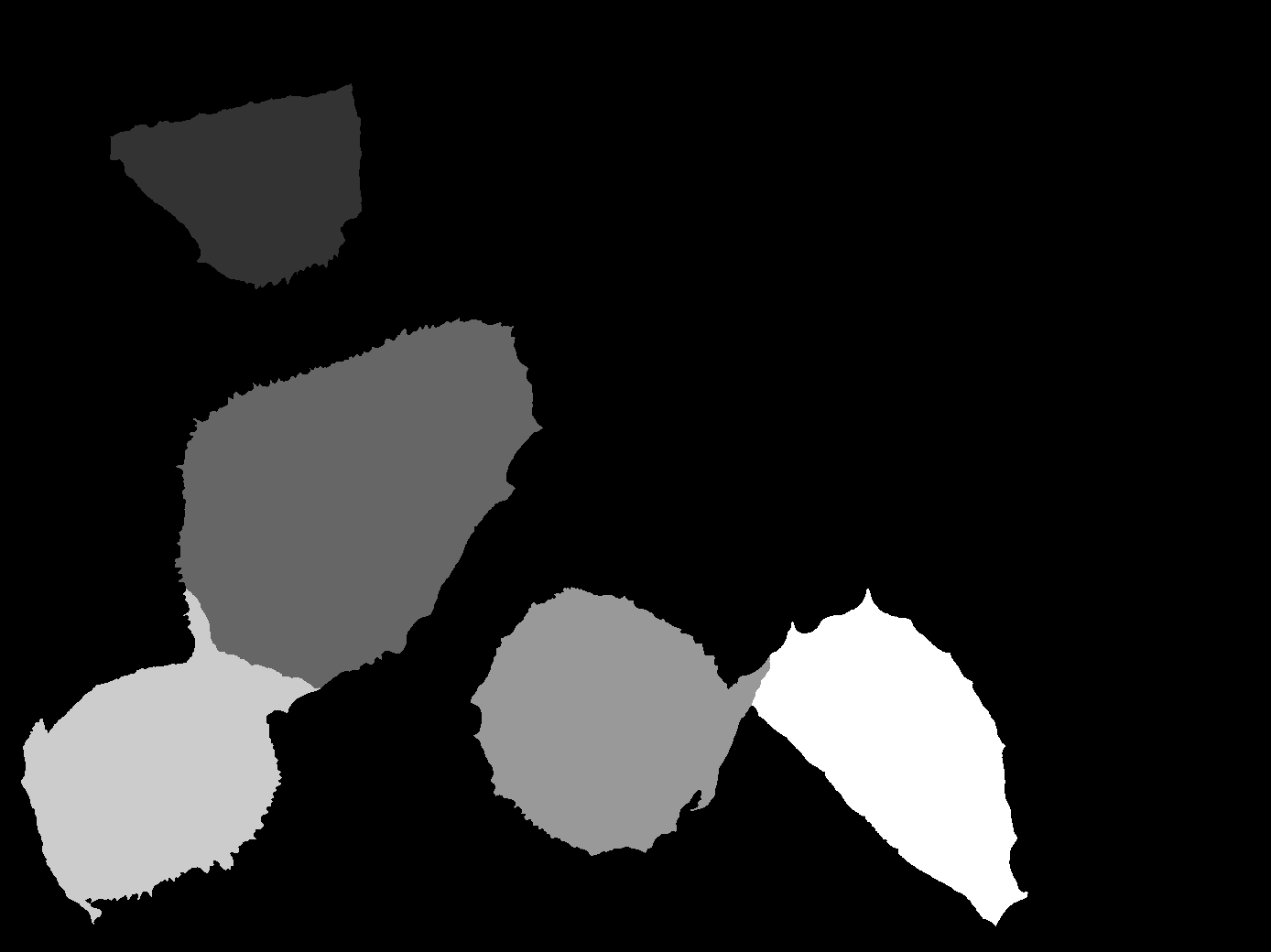

Supplement: S1 File — This file contains all scripts (CellProfiler v2.1.1 and MATLAB2016a) and data necessary to reproduce the information shown in Fig 3. (ZIP) [file pone.0180810.s001.zip › vitaminD_eColi_reproducibleResearchArchive/Results2016/C_26_c2_seg.tif]
