# Supplementary figures and images for: Vitamin D-deficient mice have more invasive urinary tract infection (part 1 of 2)
Source: PLoS One. 2017 Jul 27;12(7):e0180810. doi: 10.1371/journal.pone.0180810 (PMC5531565; doi:10.1371/journal.pone.0180810)

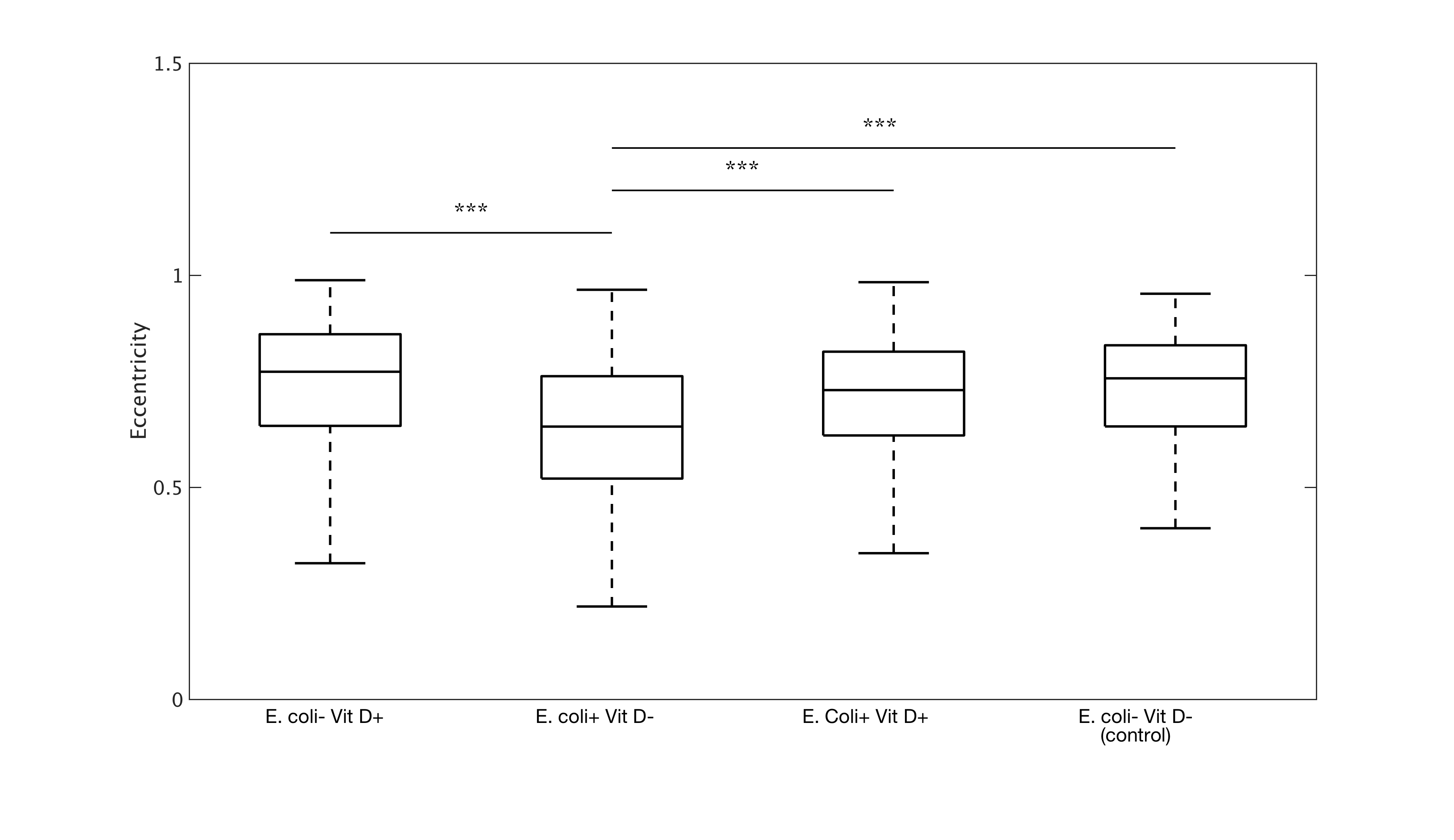

Supplement: S1 File — This file contains all scripts (CellProfiler v2.1.1 and MATLAB2016a) and data necessary to reproduce the information shown in Fig 3. (ZIP) [file pone.0180810.s001.zip › vitaminD_eColi_reproducibleResearchArchive/EccentricityPlot_v5_2016_wStars.png]

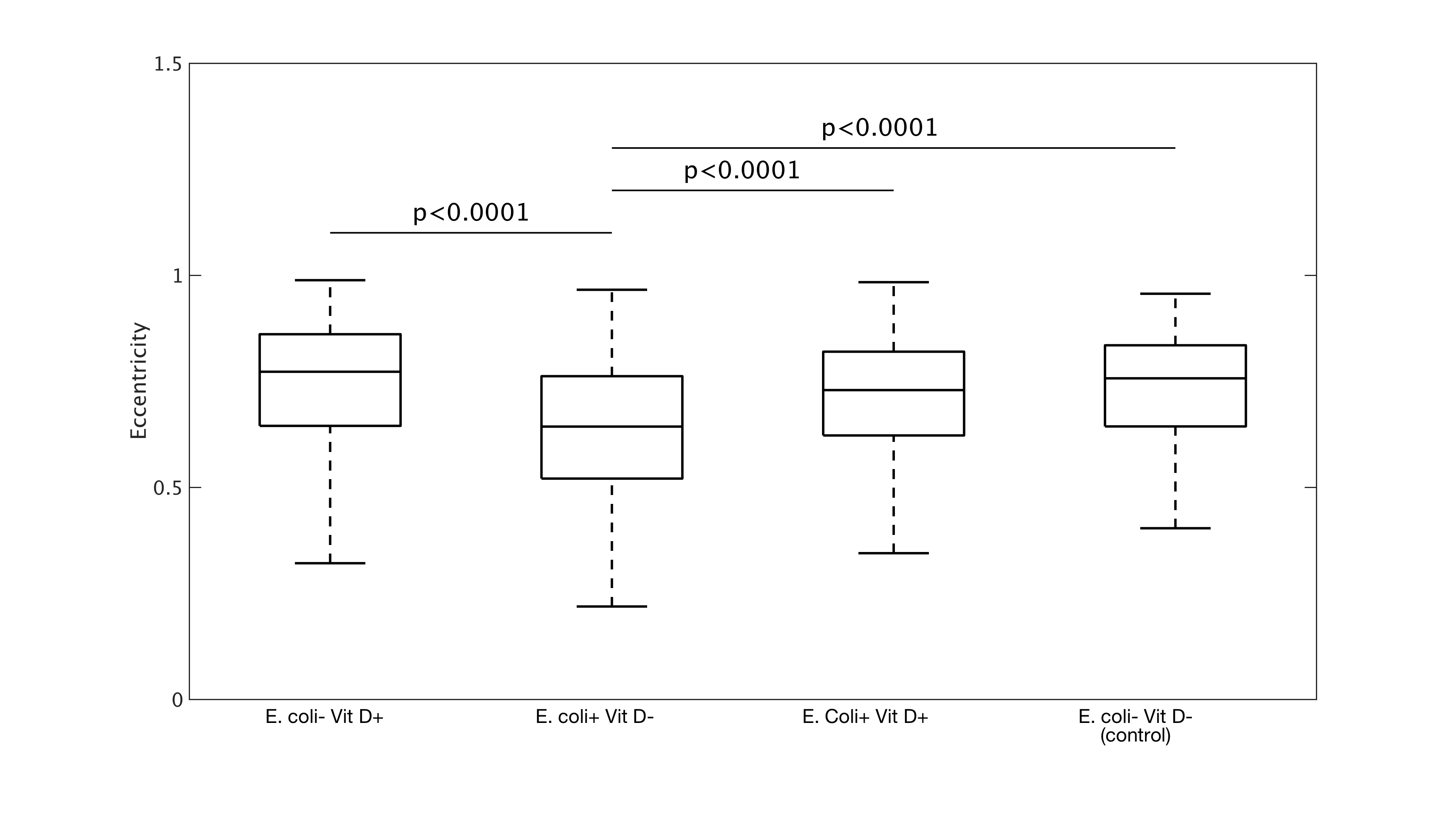

Supplement: S1 File — This file contains all scripts (CellProfiler v2.1.1 and MATLAB2016a) and data necessary to reproduce the information shown in Fig 3. (ZIP) [file pone.0180810.s001.zip › vitaminD_eColi_reproducibleResearchArchive/EccentricityPlot_v6_2016_wPvals.png]

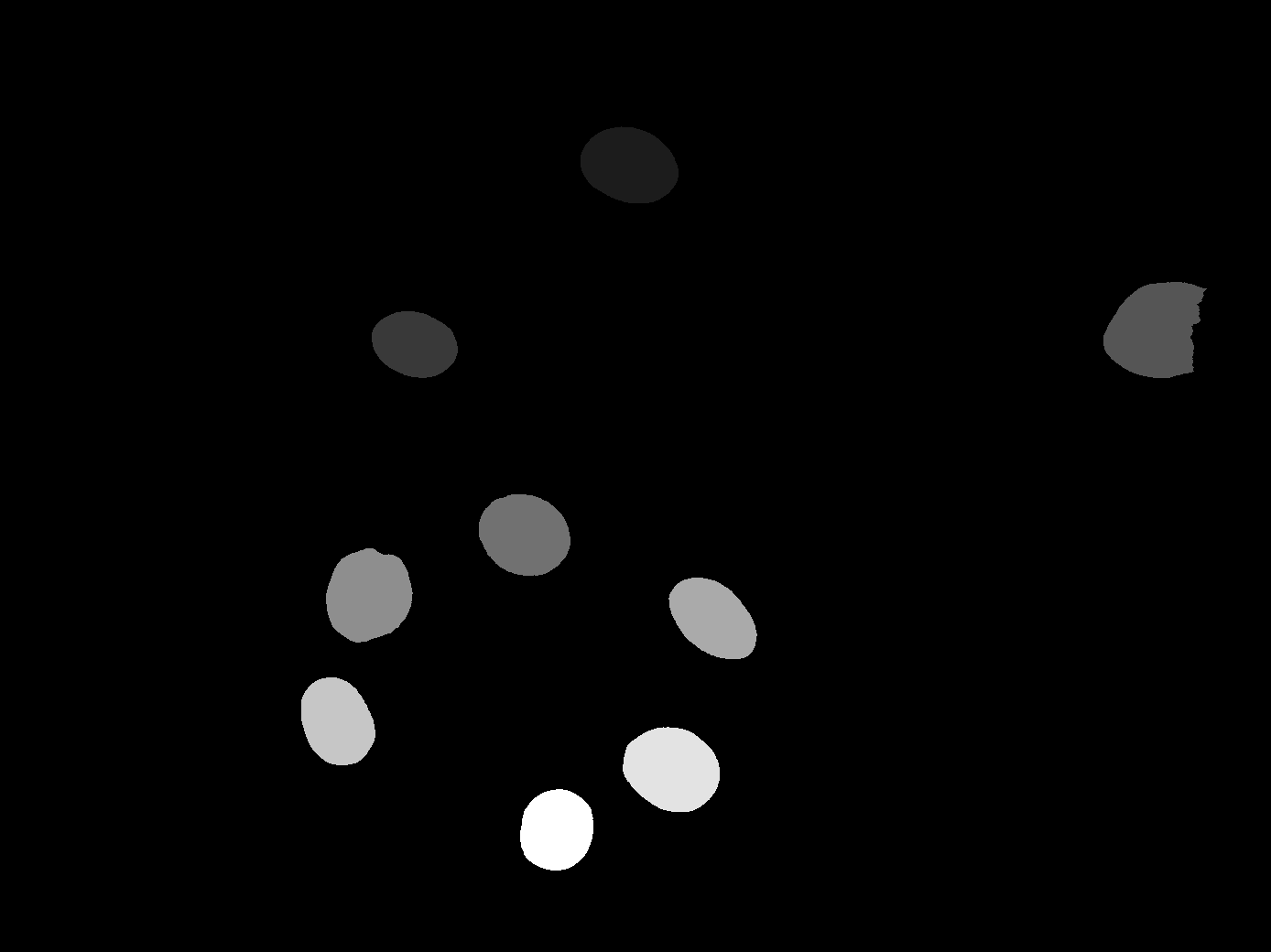

Supplement: S1 File — This file contains all scripts (CellProfiler v2.1.1 and MATLAB2016a) and data necessary to reproduce the information shown in Fig 3. (ZIP) [file pone.0180810.s001.zip › vitaminD_eColi_reproducibleResearchArchive/Results2016/A_10_c0_seg.tif]

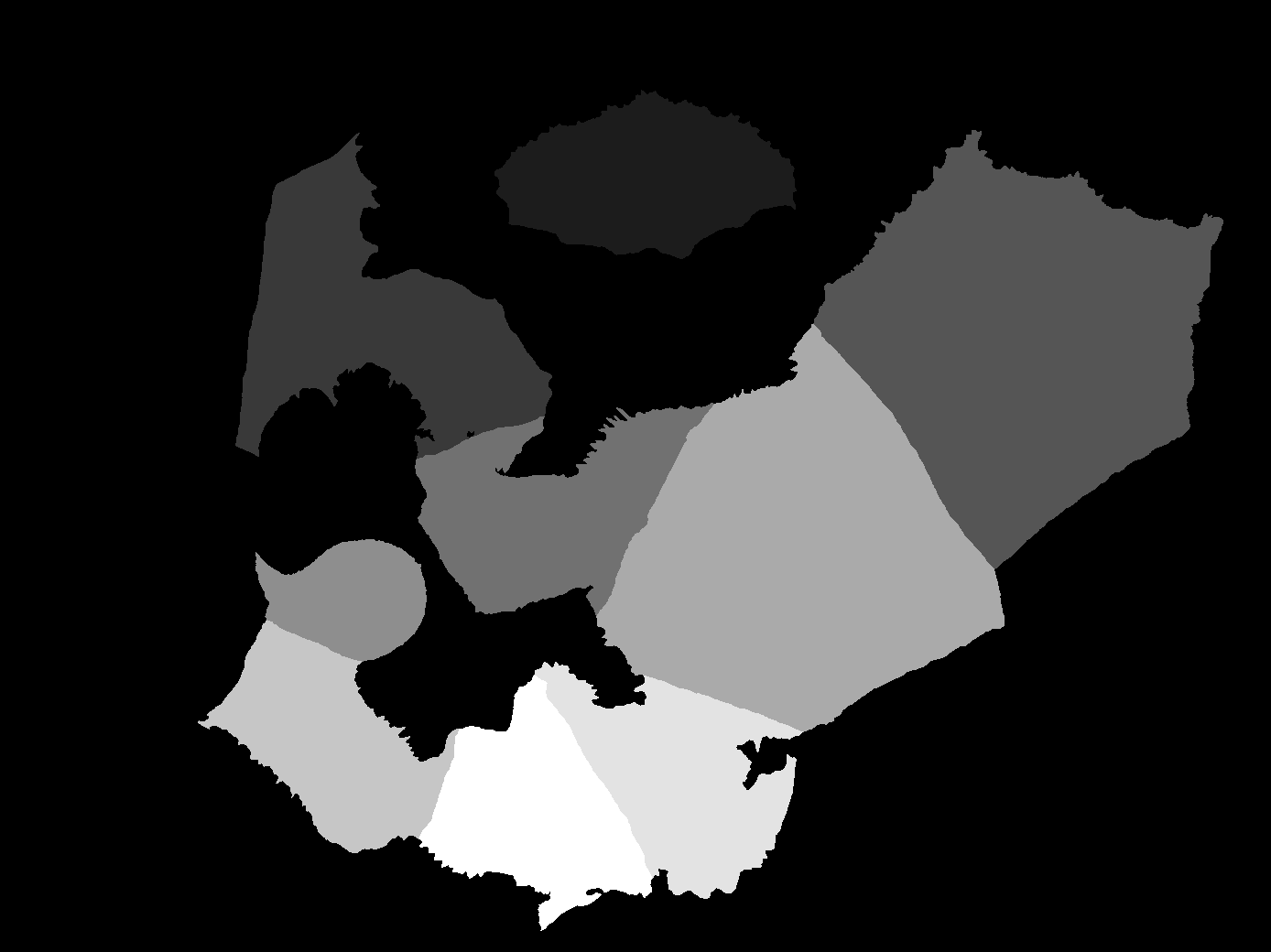

Supplement: S1 File — This file contains all scripts (CellProfiler v2.1.1 and MATLAB2016a) and data necessary to reproduce the information shown in Fig 3. (ZIP) [file pone.0180810.s001.zip › vitaminD_eColi_reproducibleResearchArchive/Results2016/A_10_c2_seg.tif]

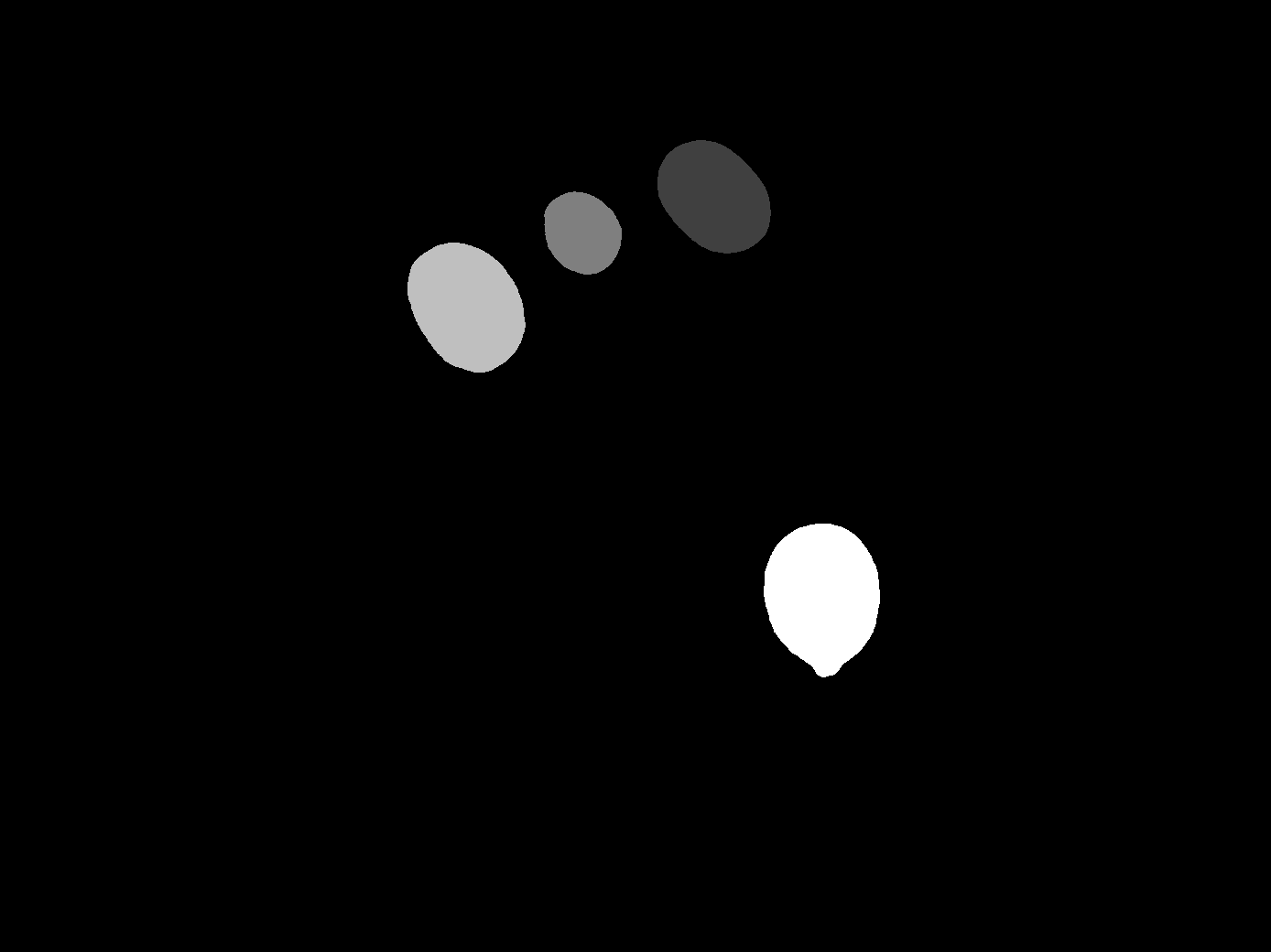

Supplement: S1 File — This file contains all scripts (CellProfiler v2.1.1 and MATLAB2016a) and data necessary to reproduce the information shown in Fig 3. (ZIP) [file pone.0180810.s001.zip › vitaminD_eColi_reproducibleResearchArchive/Results2016/A_11_c0_seg.tif]

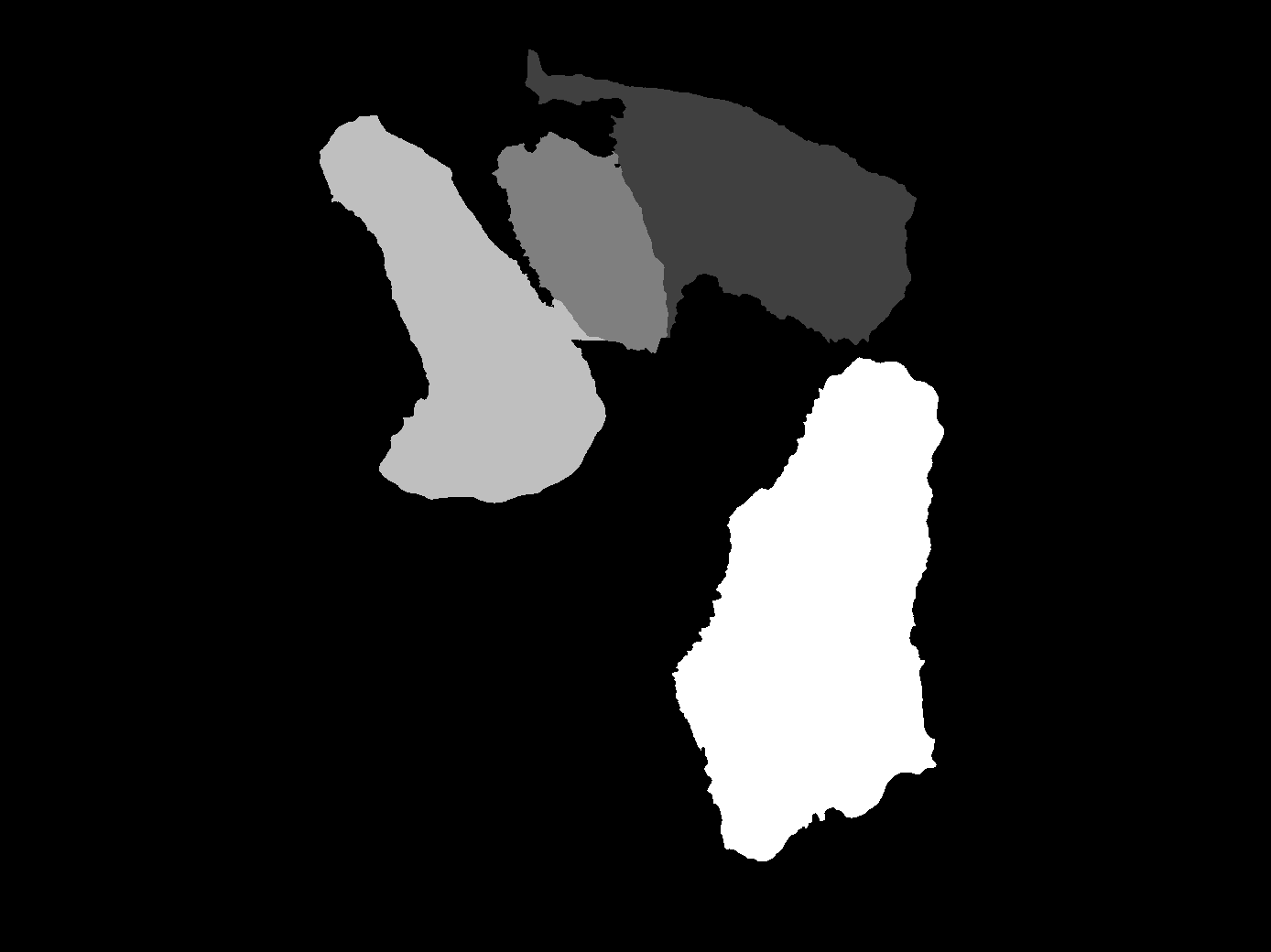

Supplement: S1 File — This file contains all scripts (CellProfiler v2.1.1 and MATLAB2016a) and data necessary to reproduce the information shown in Fig 3. (ZIP) [file pone.0180810.s001.zip › vitaminD_eColi_reproducibleResearchArchive/Results2016/A_11_c2_seg.tif]

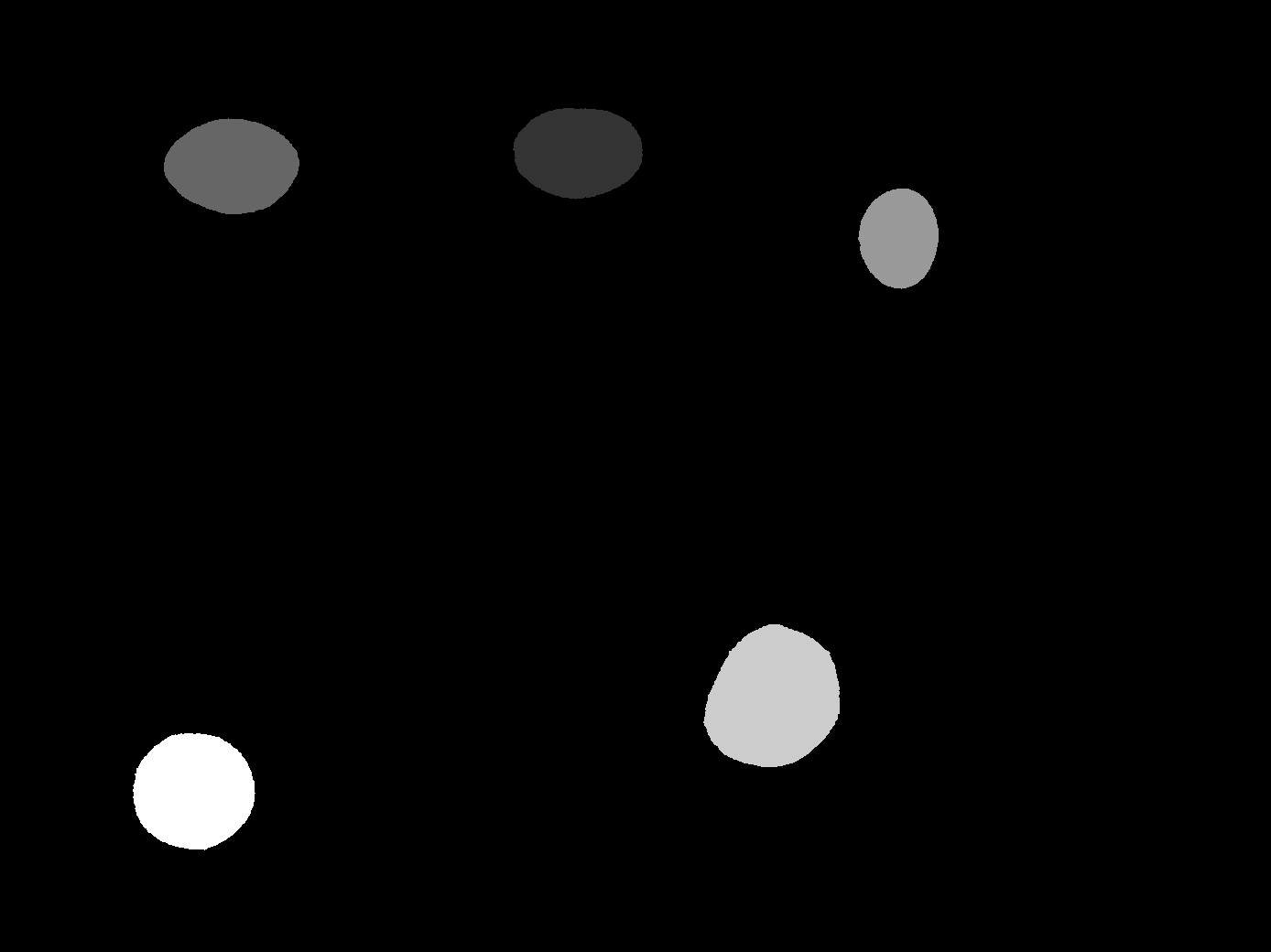

Supplement: S1 File — This file contains all scripts (CellProfiler v2.1.1 and MATLAB2016a) and data necessary to reproduce the information shown in Fig 3. (ZIP) [file pone.0180810.s001.zip › vitaminD_eColi_reproducibleResearchArchive/Results2016/A_12_c0_seg.tif]

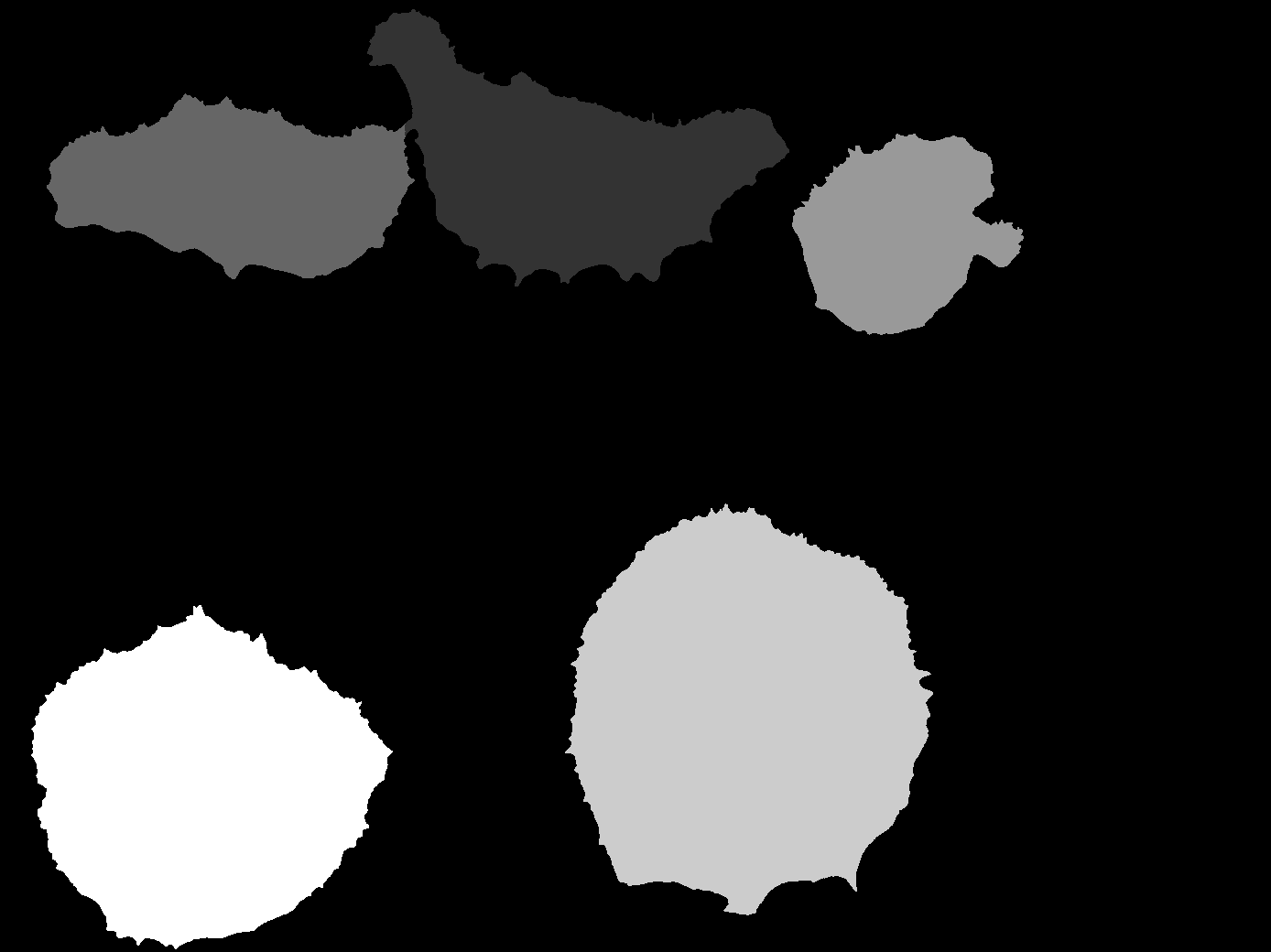

Supplement: S1 File — This file contains all scripts (CellProfiler v2.1.1 and MATLAB2016a) and data necessary to reproduce the information shown in Fig 3. (ZIP) [file pone.0180810.s001.zip › vitaminD_eColi_reproducibleResearchArchive/Results2016/A_12_c2_seg.tif]

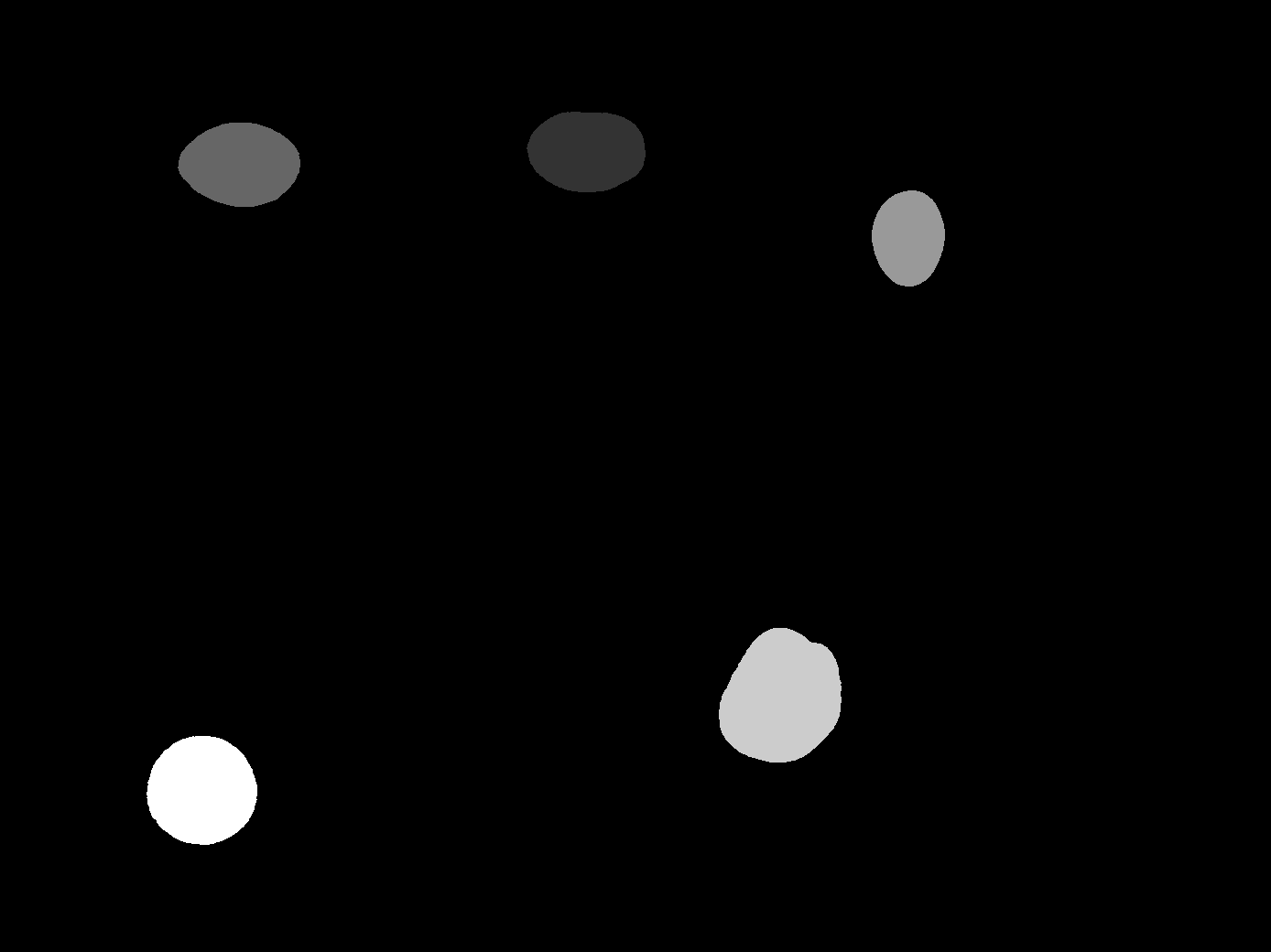

Supplement: S1 File — This file contains all scripts (CellProfiler v2.1.1 and MATLAB2016a) and data necessary to reproduce the information shown in Fig 3. (ZIP) [file pone.0180810.s001.zip › vitaminD_eColi_reproducibleResearchArchive/Results2016/A_13_c0_seg.tif]

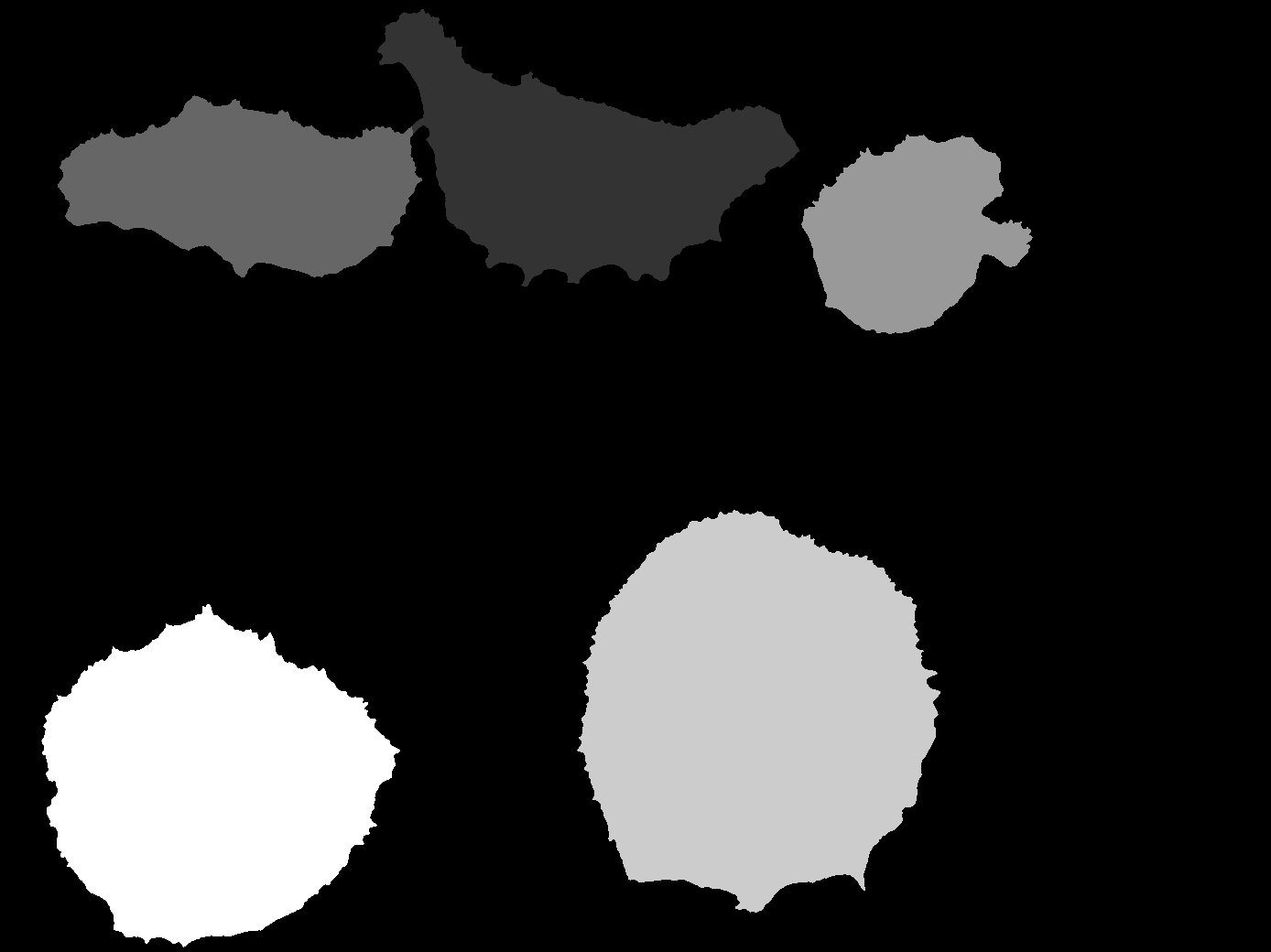

Supplement: S1 File — This file contains all scripts (CellProfiler v2.1.1 and MATLAB2016a) and data necessary to reproduce the information shown in Fig 3. (ZIP) [file pone.0180810.s001.zip › vitaminD_eColi_reproducibleResearchArchive/Results2016/A_13_c2_seg.tif]

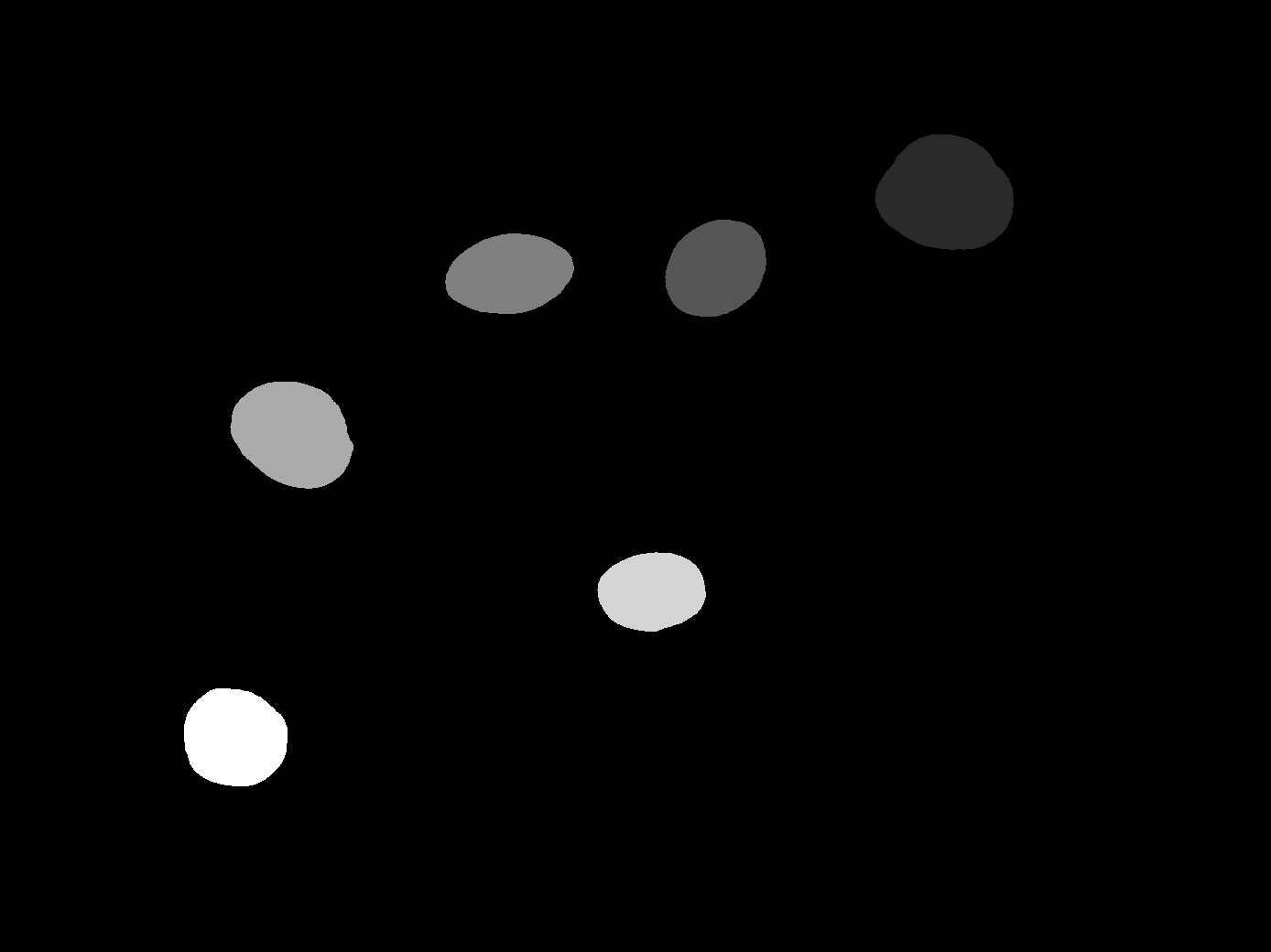

Supplement: S1 File — This file contains all scripts (CellProfiler v2.1.1 and MATLAB2016a) and data necessary to reproduce the information shown in Fig 3. (ZIP) [file pone.0180810.s001.zip › vitaminD_eColi_reproducibleResearchArchive/Results2016/A_14_c0_seg.tif]

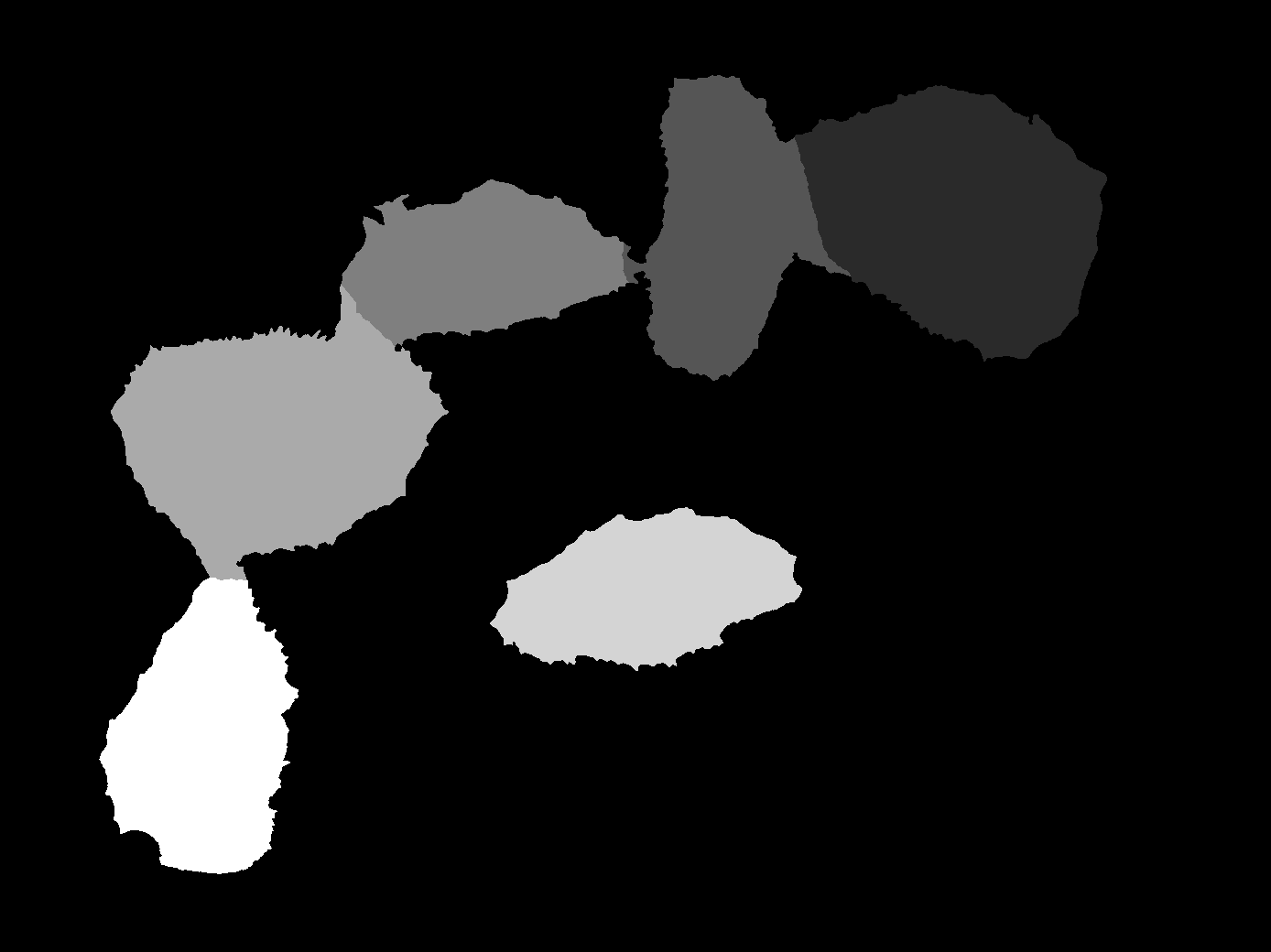

Supplement: S1 File — This file contains all scripts (CellProfiler v2.1.1 and MATLAB2016a) and data necessary to reproduce the information shown in Fig 3. (ZIP) [file pone.0180810.s001.zip › vitaminD_eColi_reproducibleResearchArchive/Results2016/A_14_c2_seg.tif]

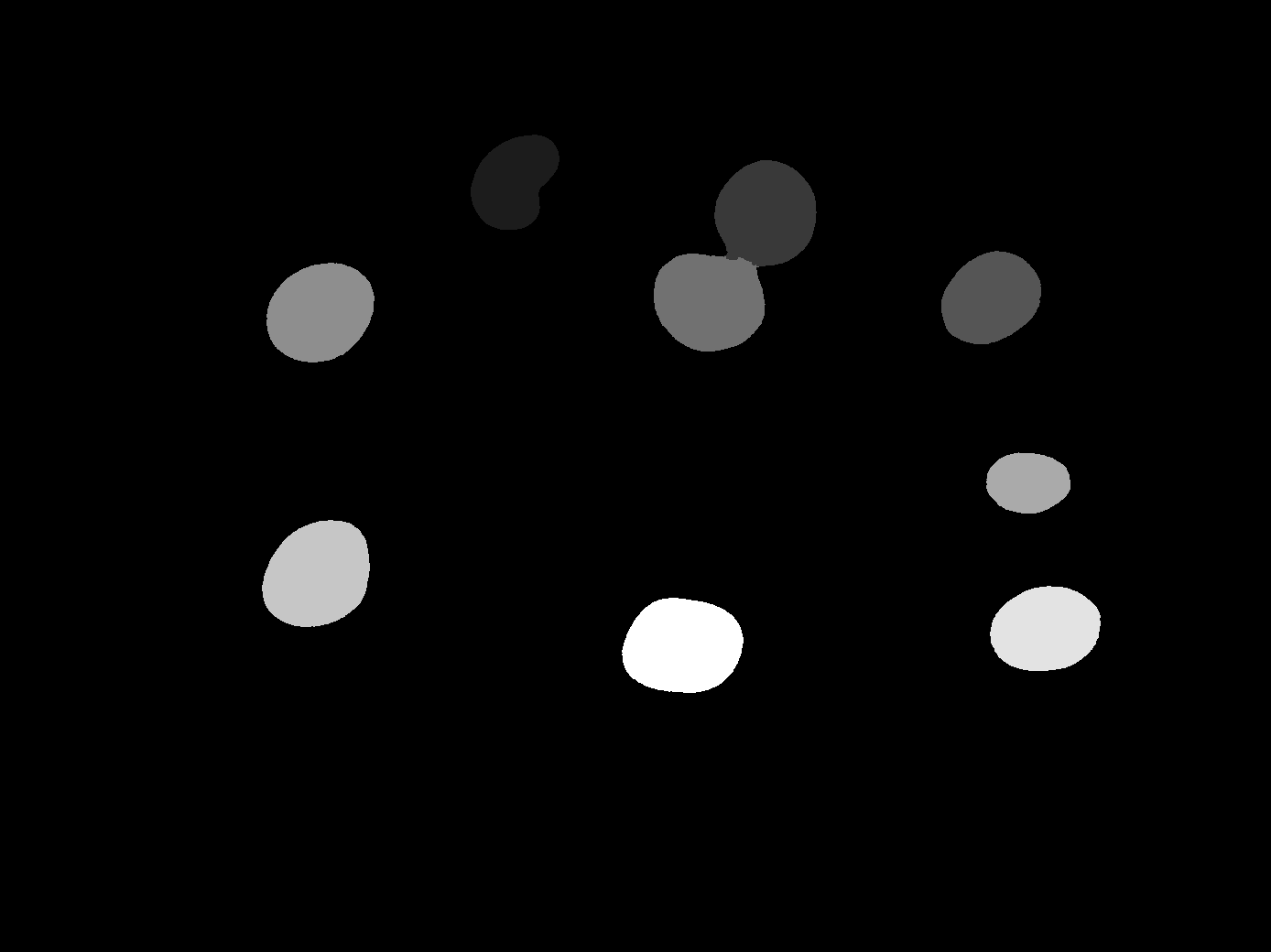

Supplement: S1 File — This file contains all scripts (CellProfiler v2.1.1 and MATLAB2016a) and data necessary to reproduce the information shown in Fig 3. (ZIP) [file pone.0180810.s001.zip › vitaminD_eColi_reproducibleResearchArchive/Results2016/A_15_c0_seg.tif]

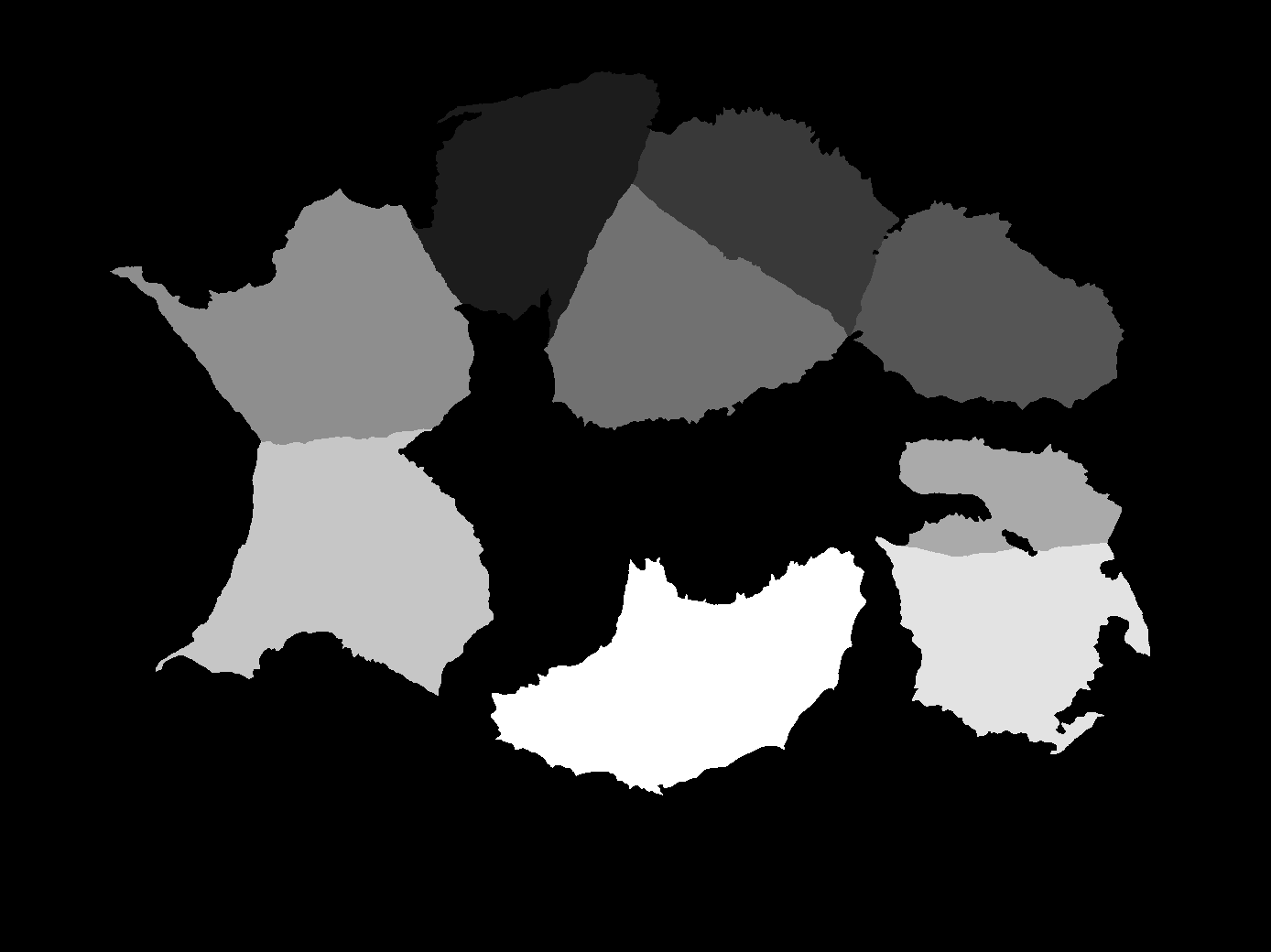

Supplement: S1 File — This file contains all scripts (CellProfiler v2.1.1 and MATLAB2016a) and data necessary to reproduce the information shown in Fig 3. (ZIP) [file pone.0180810.s001.zip › vitaminD_eColi_reproducibleResearchArchive/Results2016/A_15_c2_seg.tif]

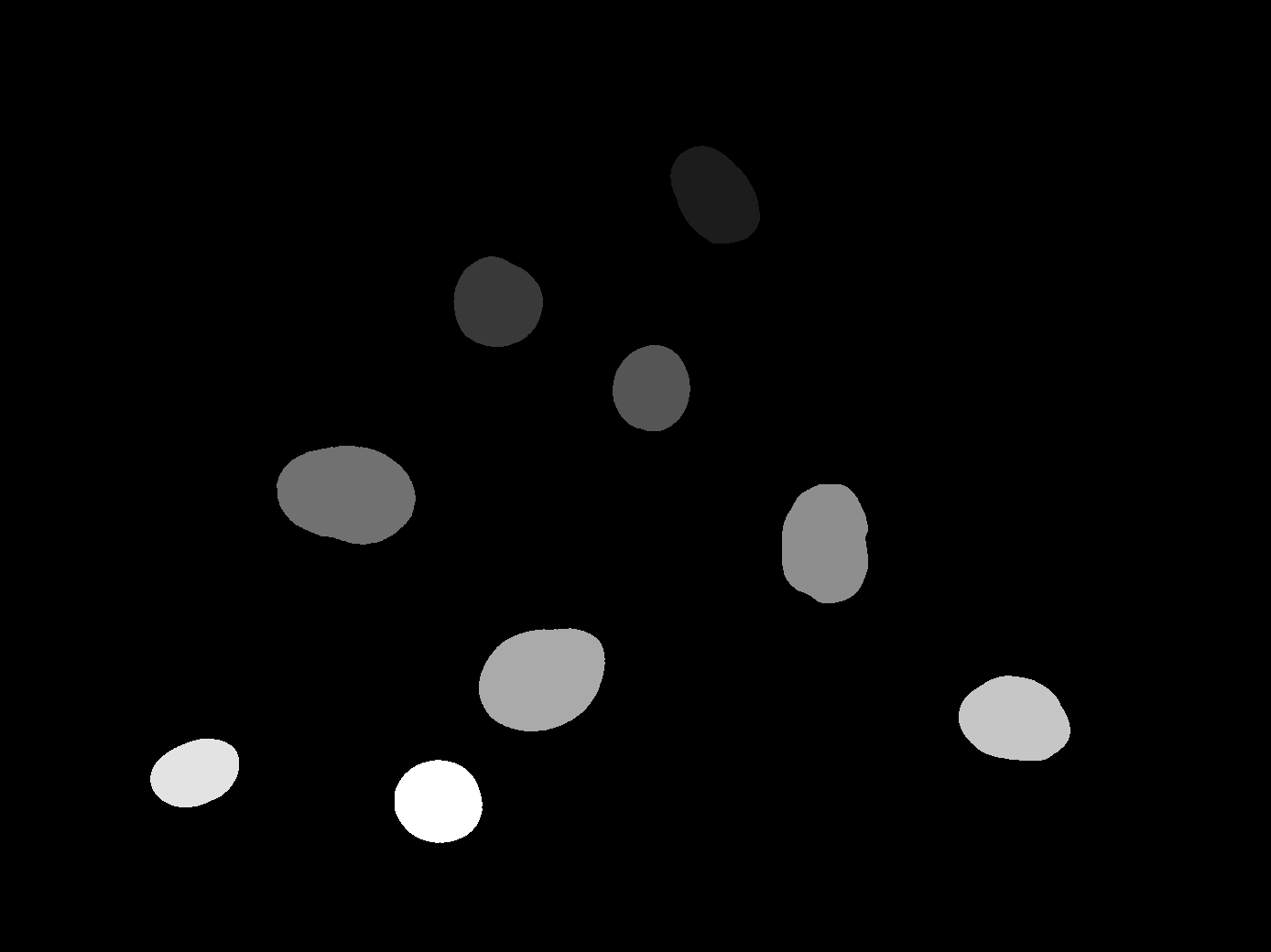

Supplement: S1 File — This file contains all scripts (CellProfiler v2.1.1 and MATLAB2016a) and data necessary to reproduce the information shown in Fig 3. (ZIP) [file pone.0180810.s001.zip › vitaminD_eColi_reproducibleResearchArchive/Results2016/A_16_c0_seg.tif]

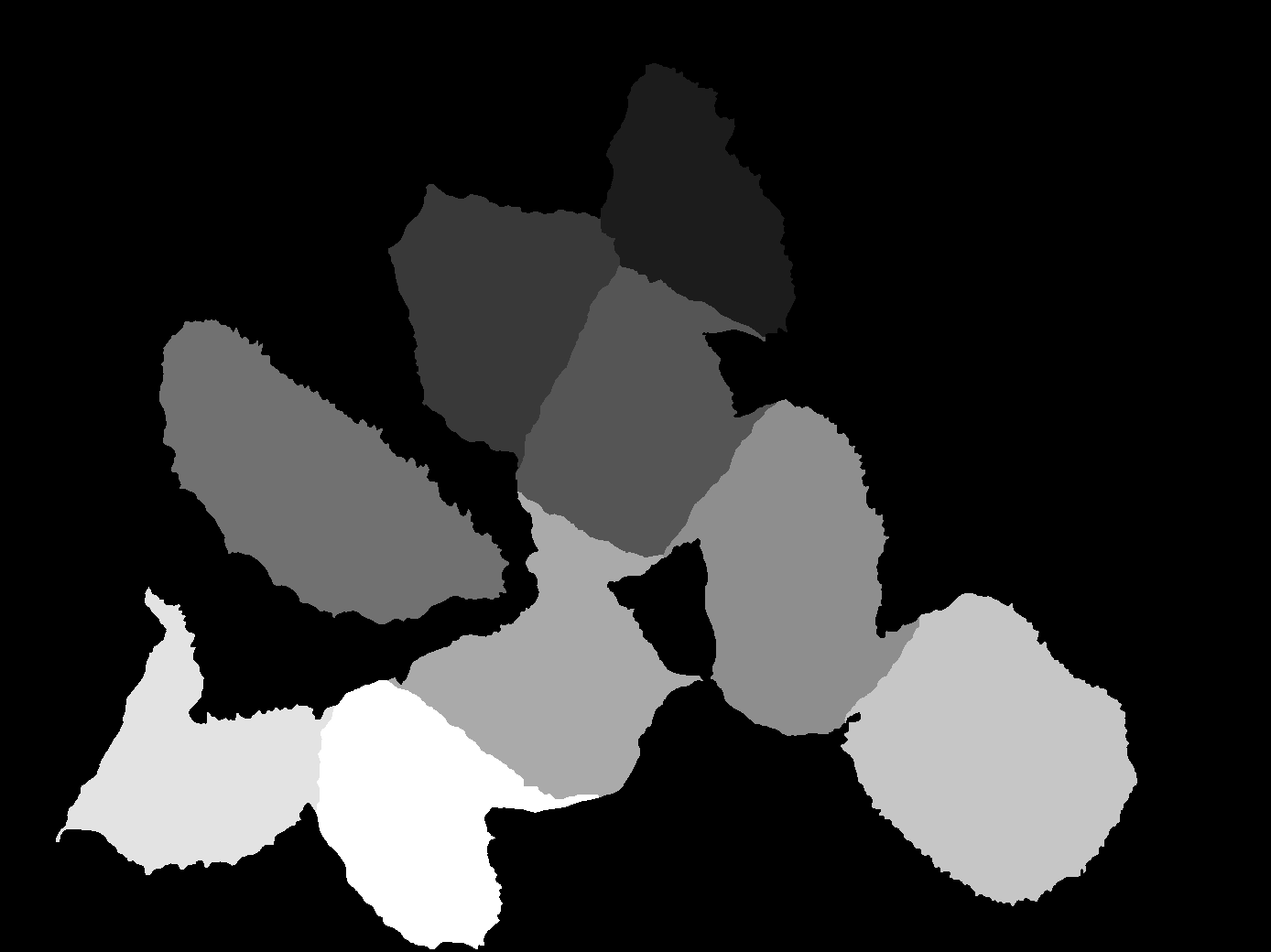

Supplement: S1 File — This file contains all scripts (CellProfiler v2.1.1 and MATLAB2016a) and data necessary to reproduce the information shown in Fig 3. (ZIP) [file pone.0180810.s001.zip › vitaminD_eColi_reproducibleResearchArchive/Results2016/A_16_c2_seg.tif]

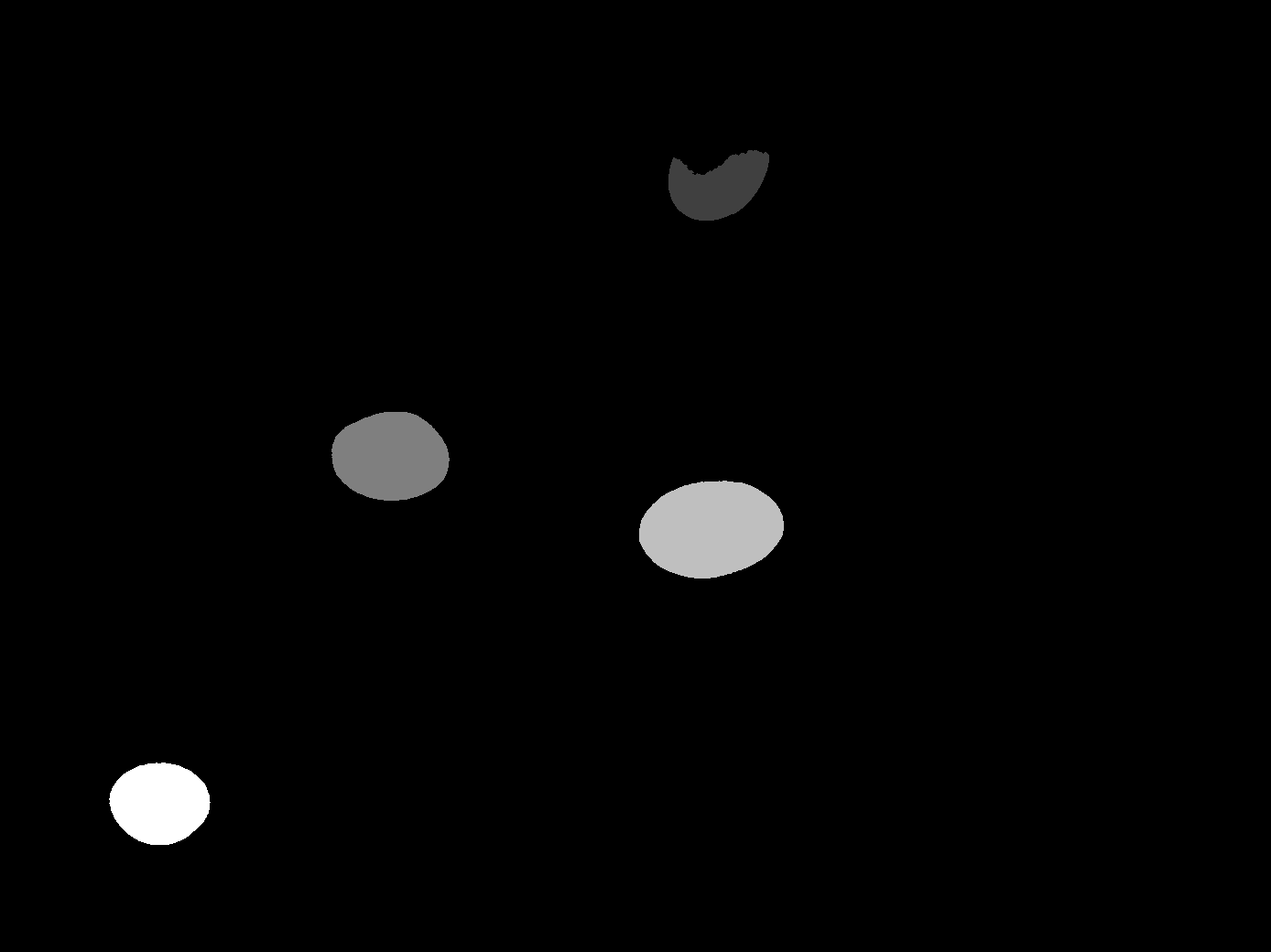

Supplement: S1 File — This file contains all scripts (CellProfiler v2.1.1 and MATLAB2016a) and data necessary to reproduce the information shown in Fig 3. (ZIP) [file pone.0180810.s001.zip › vitaminD_eColi_reproducibleResearchArchive/Results2016/A_17_c0_seg.tif]

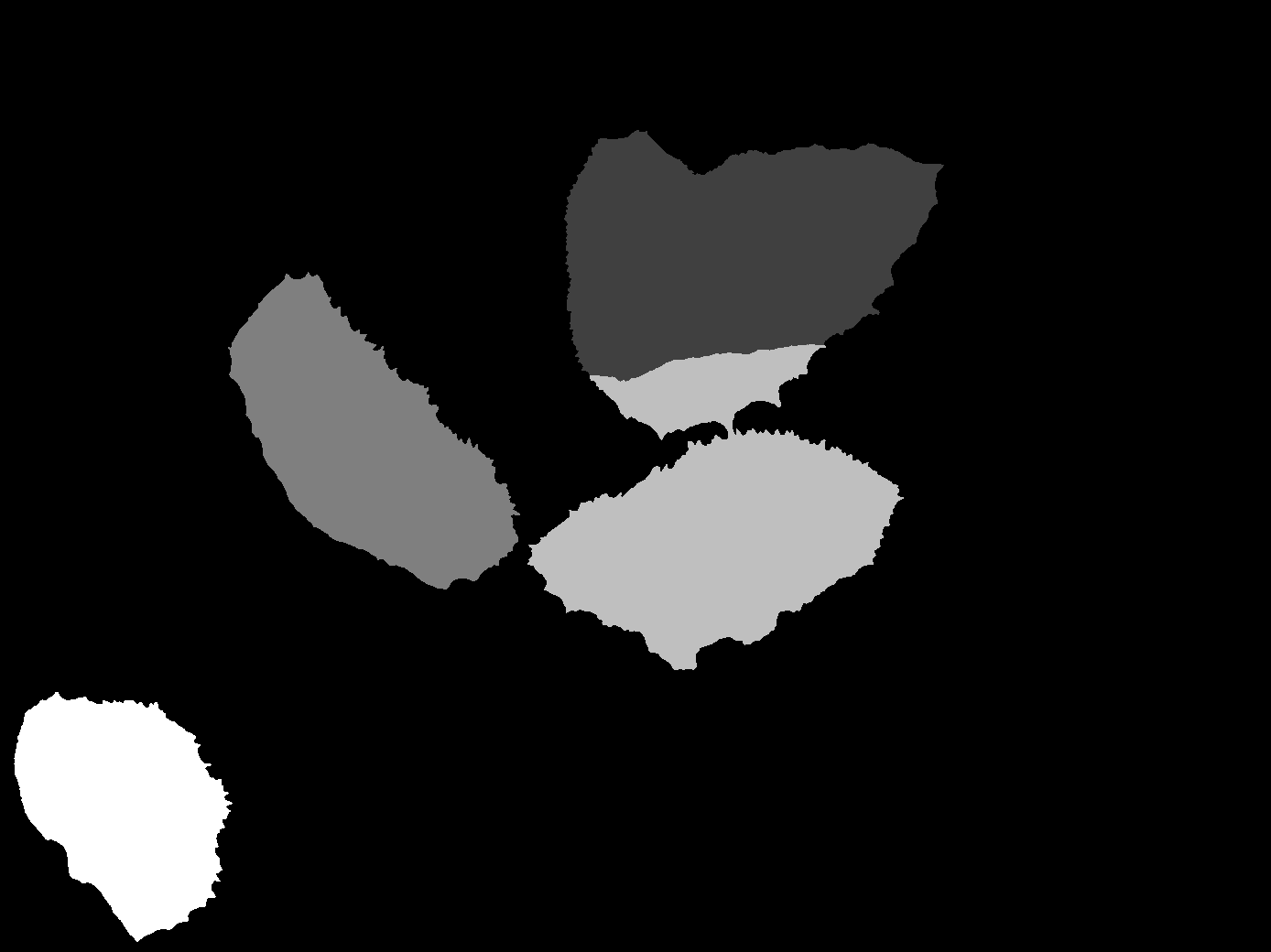

Supplement: S1 File — This file contains all scripts (CellProfiler v2.1.1 and MATLAB2016a) and data necessary to reproduce the information shown in Fig 3. (ZIP) [file pone.0180810.s001.zip › vitaminD_eColi_reproducibleResearchArchive/Results2016/A_17_c2_seg.tif]

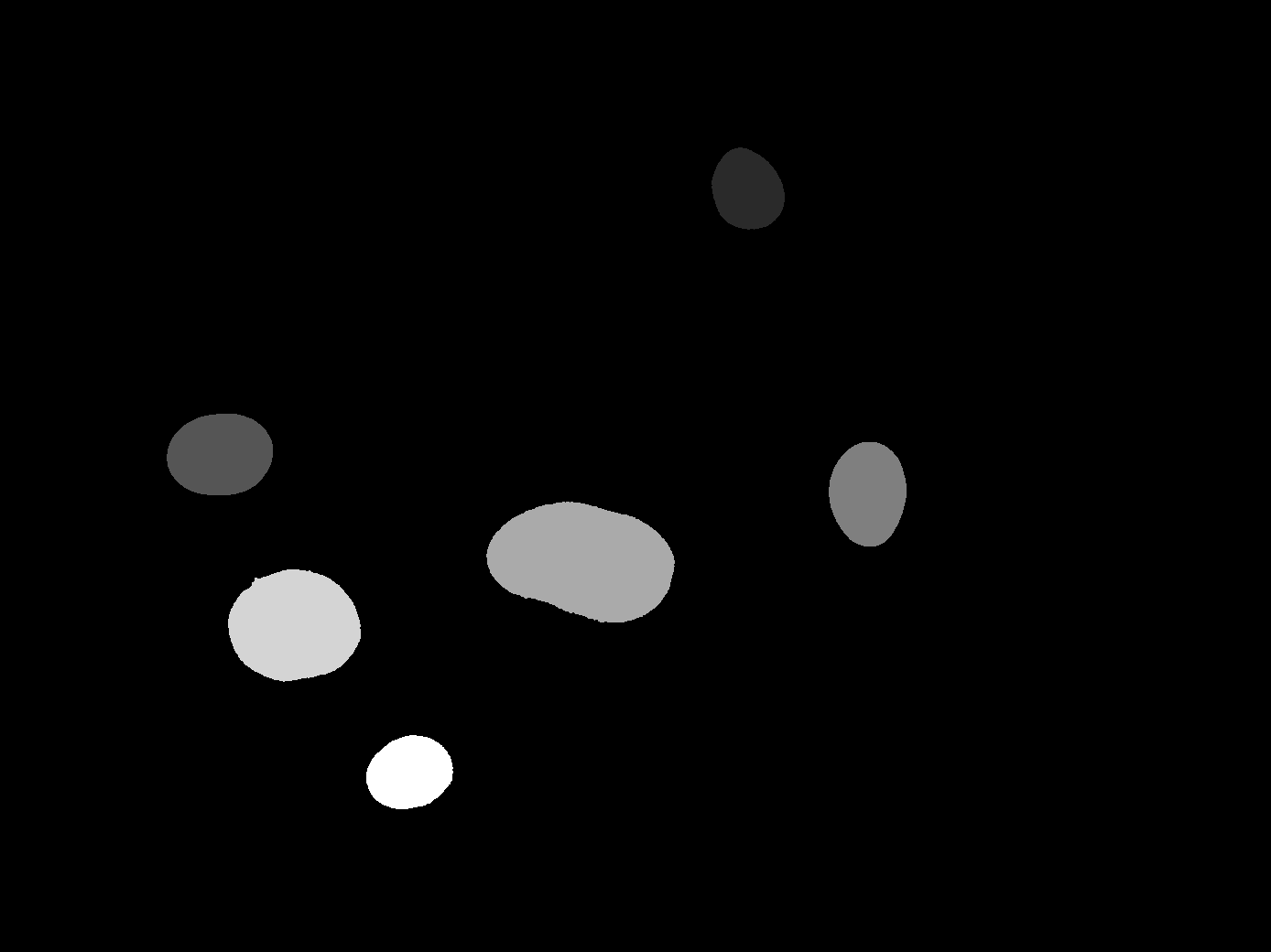

Supplement: S1 File — This file contains all scripts (CellProfiler v2.1.1 and MATLAB2016a) and data necessary to reproduce the information shown in Fig 3. (ZIP) [file pone.0180810.s001.zip › vitaminD_eColi_reproducibleResearchArchive/Results2016/A_18_c0_seg.tif]

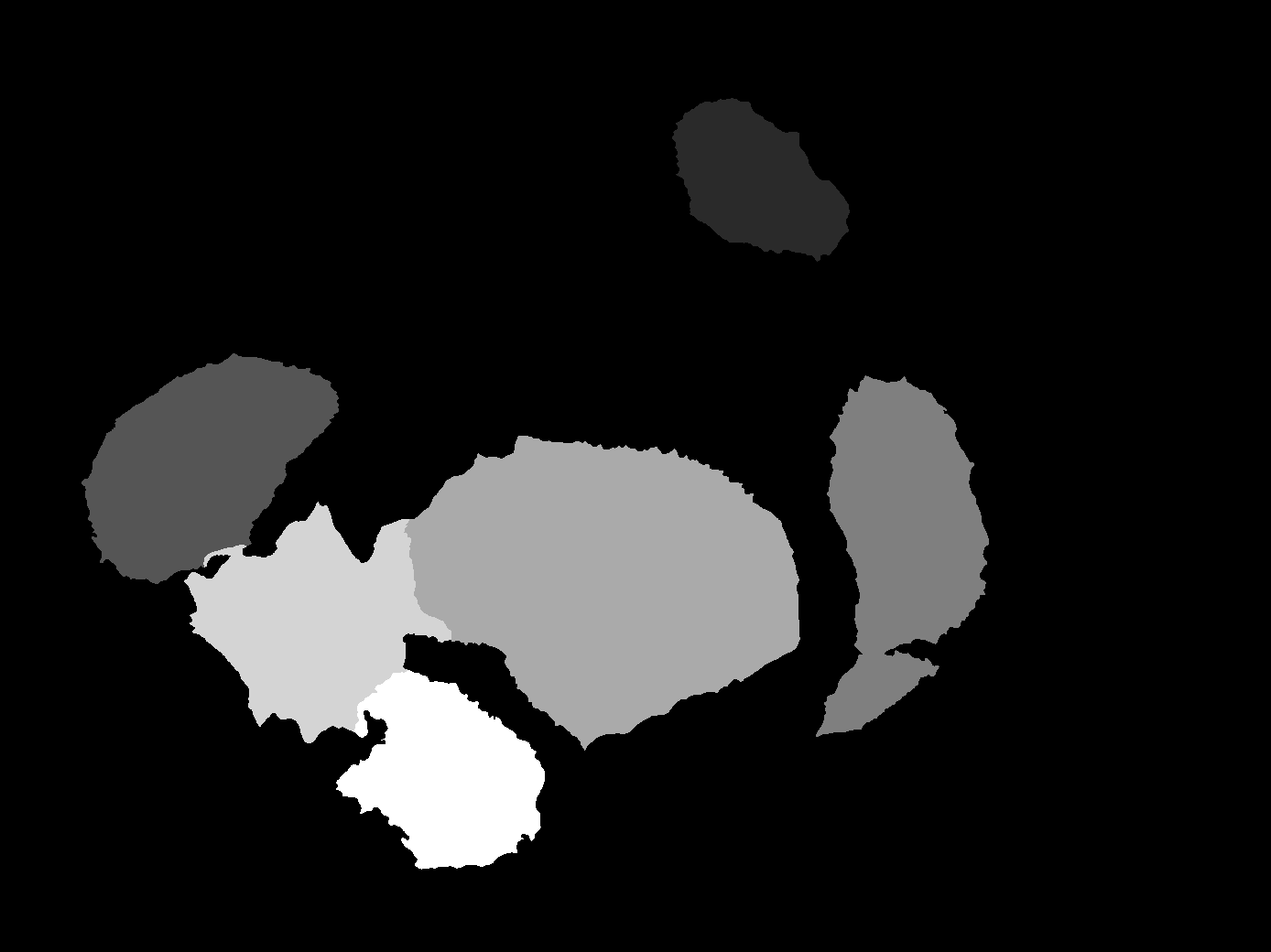

Supplement: S1 File — This file contains all scripts (CellProfiler v2.1.1 and MATLAB2016a) and data necessary to reproduce the information shown in Fig 3. (ZIP) [file pone.0180810.s001.zip › vitaminD_eColi_reproducibleResearchArchive/Results2016/A_18_c2_seg.tif]

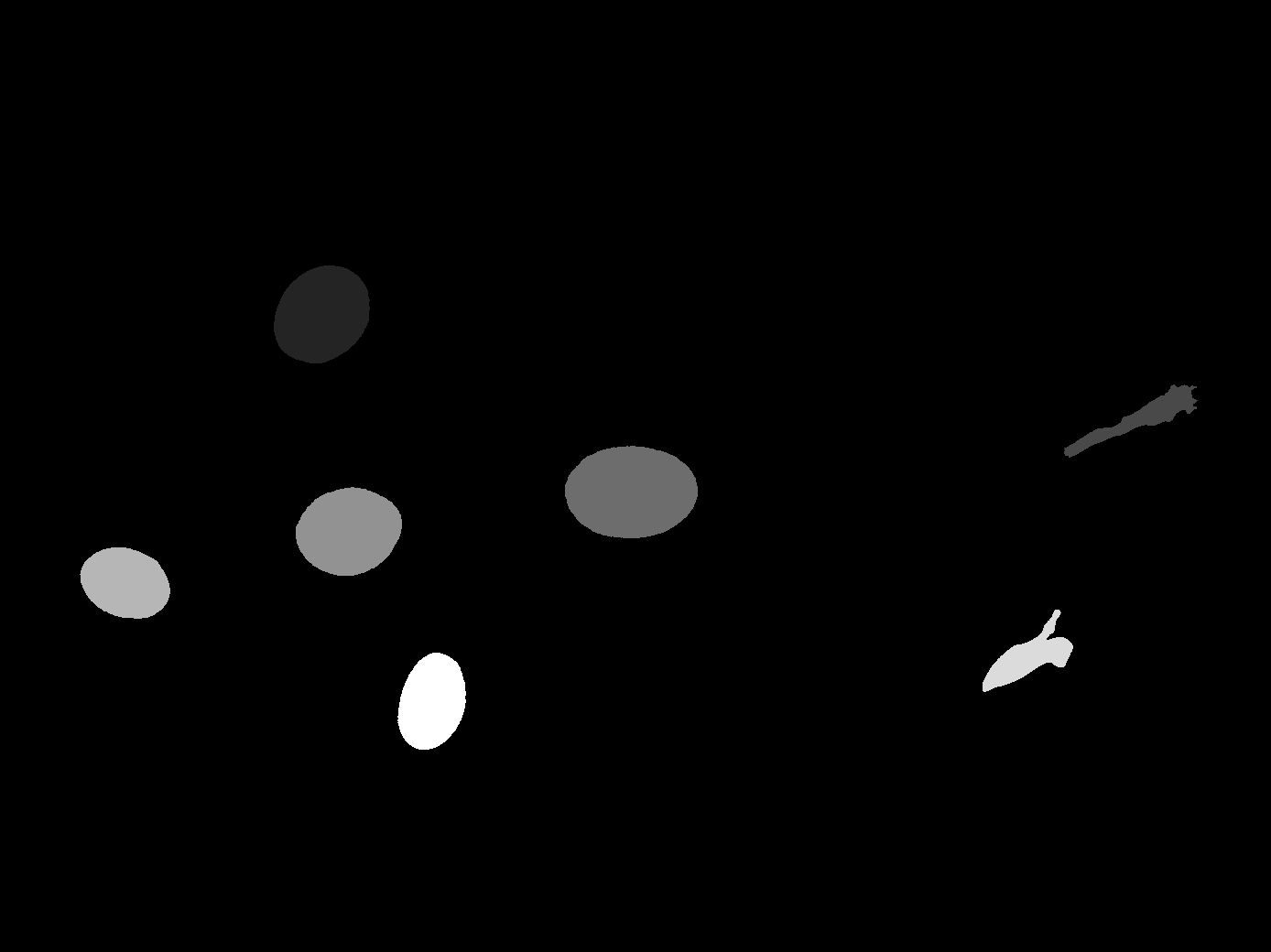

Supplement: S1 File — This file contains all scripts (CellProfiler v2.1.1 and MATLAB2016a) and data necessary to reproduce the information shown in Fig 3. (ZIP) [file pone.0180810.s001.zip › vitaminD_eColi_reproducibleResearchArchive/Results2016/A_19_c0_seg.tif]

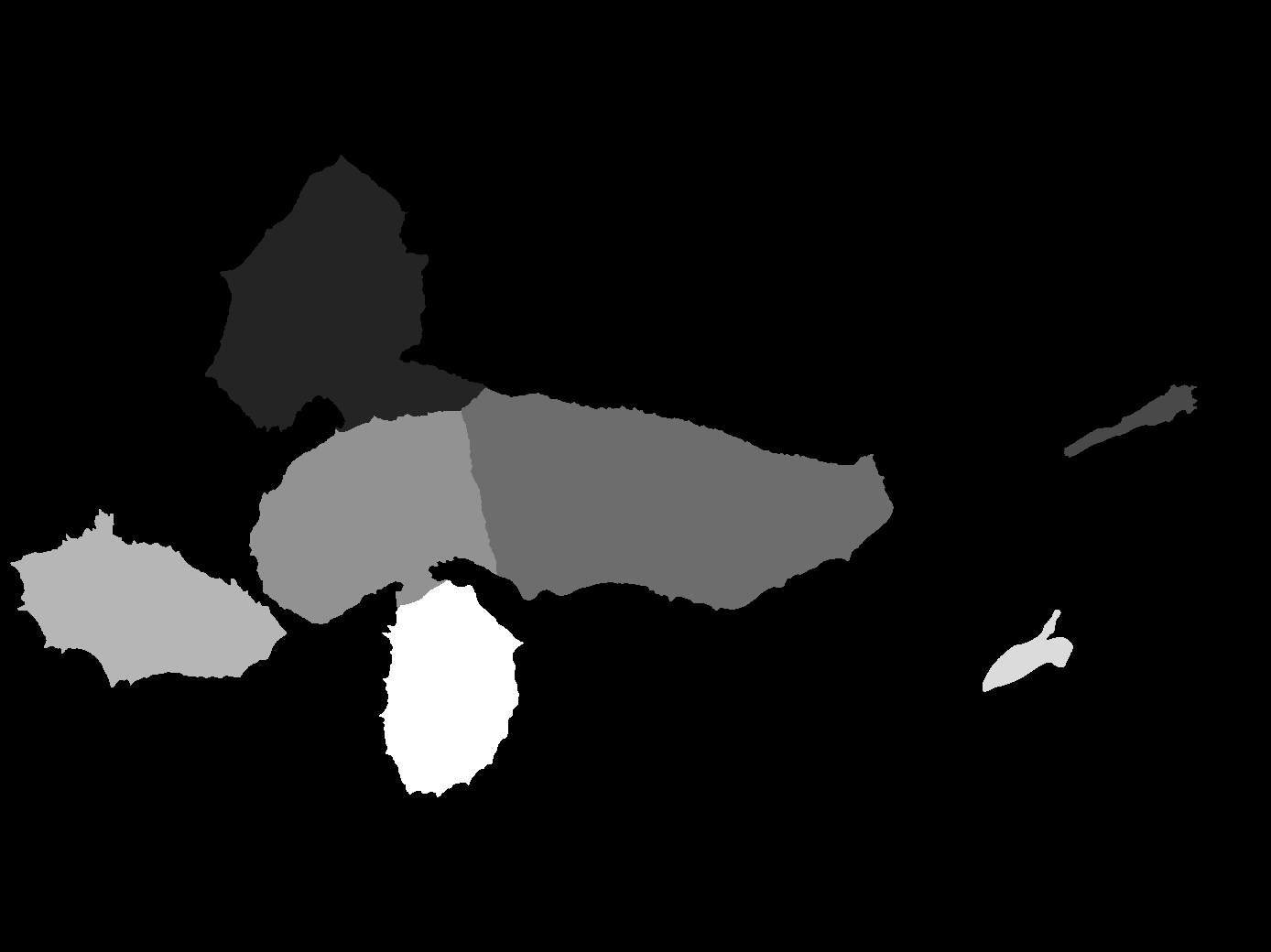

Supplement: S1 File — This file contains all scripts (CellProfiler v2.1.1 and MATLAB2016a) and data necessary to reproduce the information shown in Fig 3. (ZIP) [file pone.0180810.s001.zip › vitaminD_eColi_reproducibleResearchArchive/Results2016/A_19_c2_seg.tif]

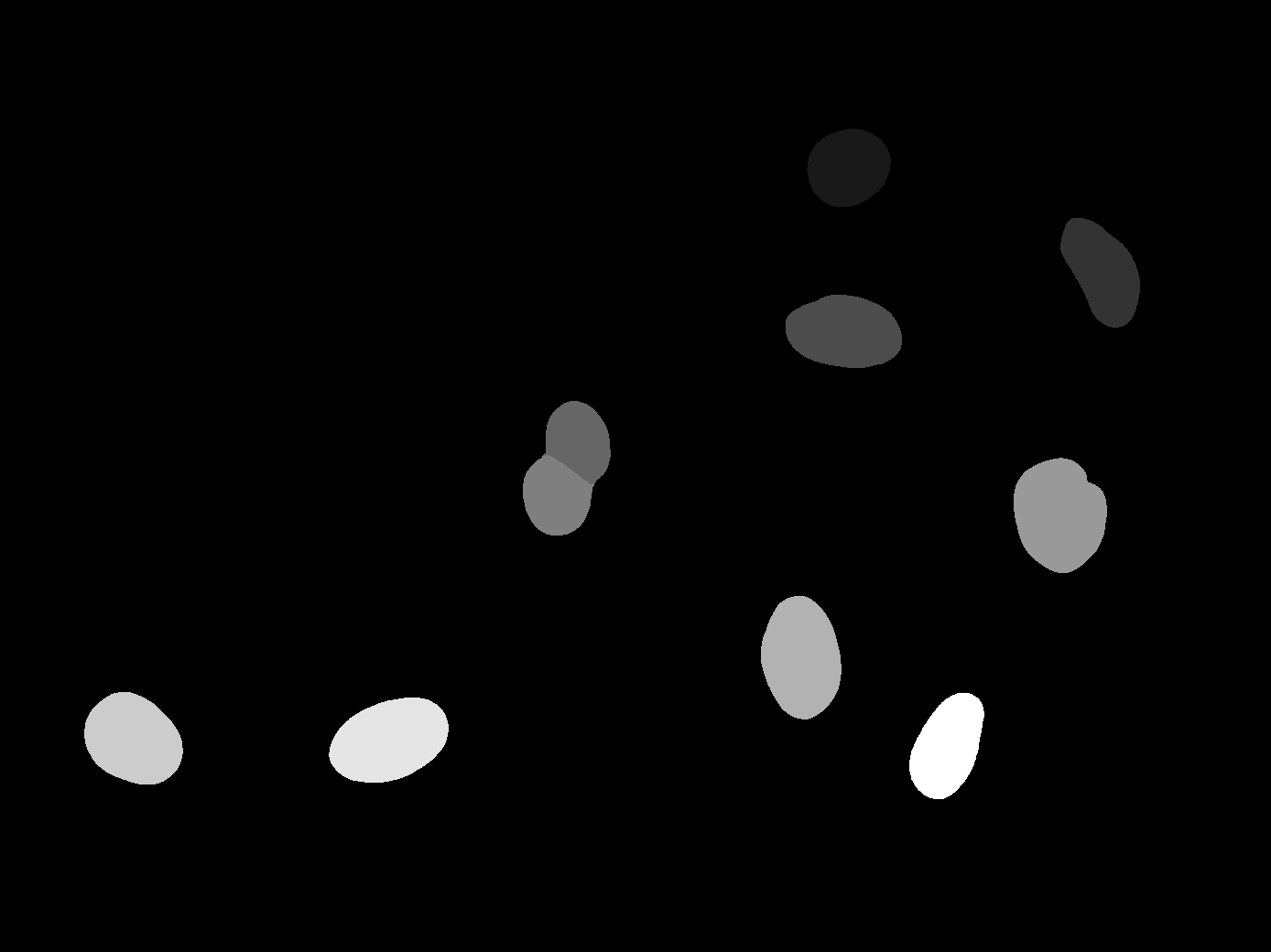

Supplement: S1 File — This file contains all scripts (CellProfiler v2.1.1 and MATLAB2016a) and data necessary to reproduce the information shown in Fig 3. (ZIP) [file pone.0180810.s001.zip › vitaminD_eColi_reproducibleResearchArchive/Results2016/A_1_c0_seg.tif]

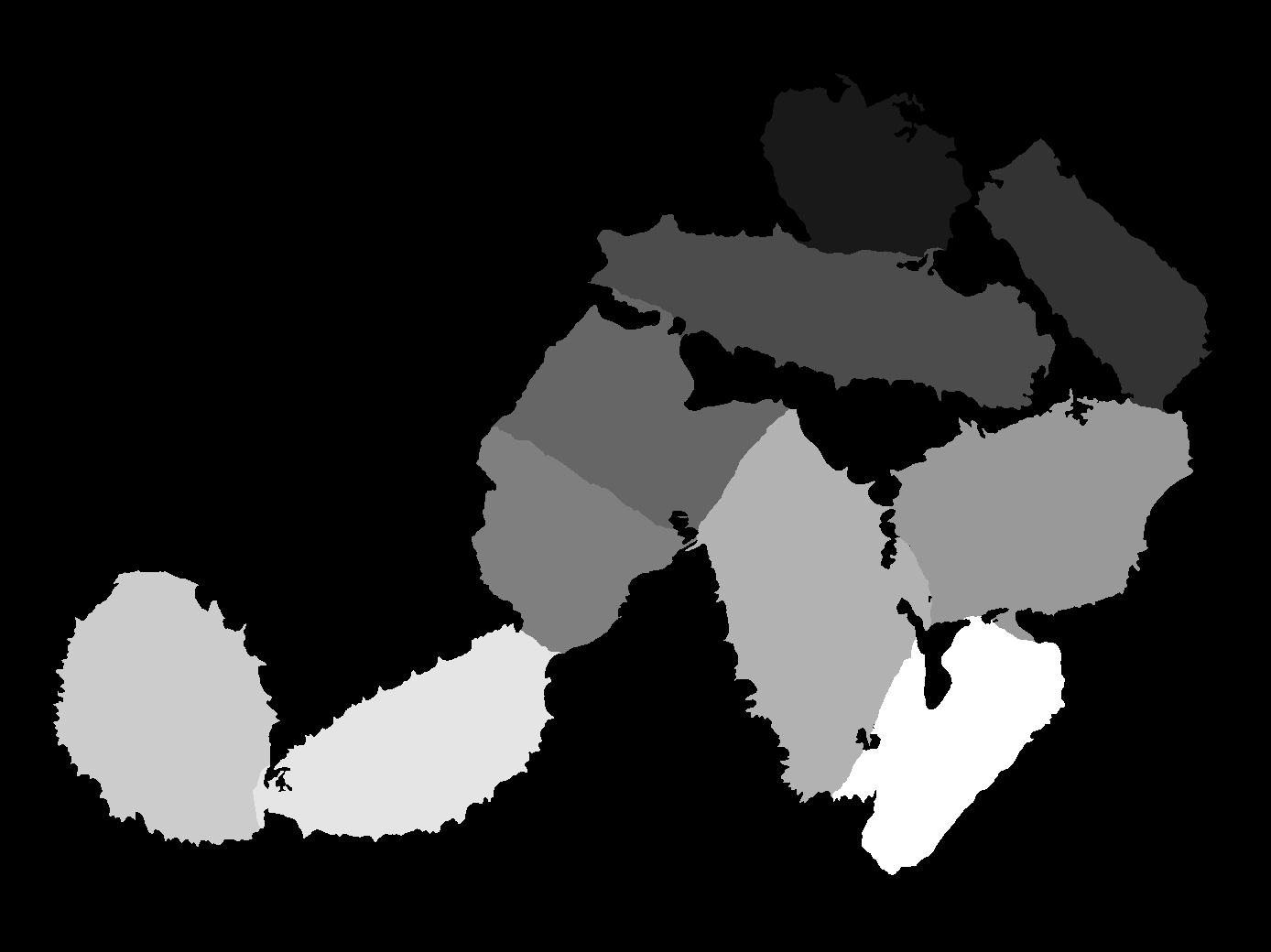

Supplement: S1 File — This file contains all scripts (CellProfiler v2.1.1 and MATLAB2016a) and data necessary to reproduce the information shown in Fig 3. (ZIP) [file pone.0180810.s001.zip › vitaminD_eColi_reproducibleResearchArchive/Results2016/A_1_c2_seg.tif]

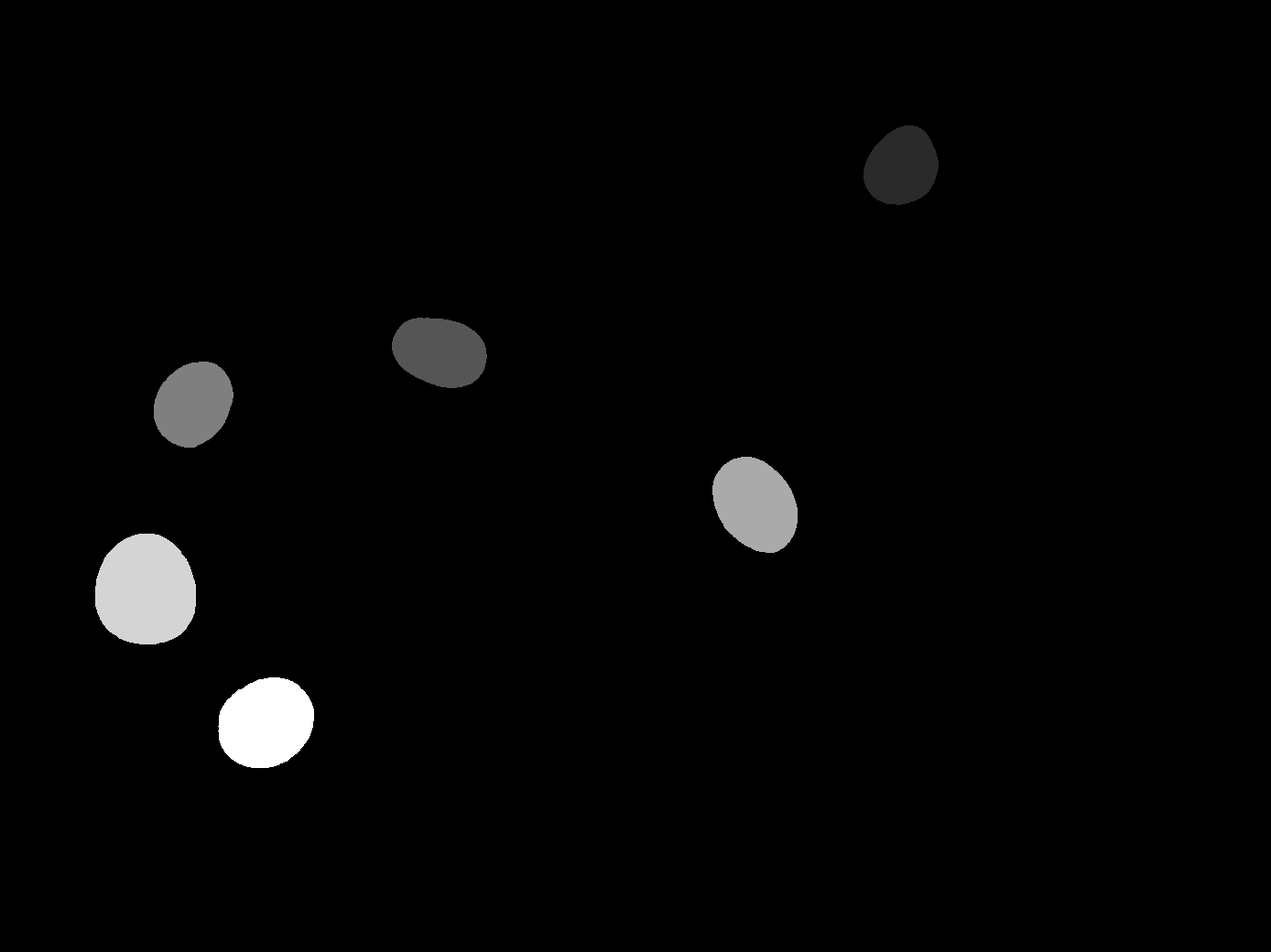

Supplement: S1 File — This file contains all scripts (CellProfiler v2.1.1 and MATLAB2016a) and data necessary to reproduce the information shown in Fig 3. (ZIP) [file pone.0180810.s001.zip › vitaminD_eColi_reproducibleResearchArchive/Results2016/A_20_c0_seg.tif]

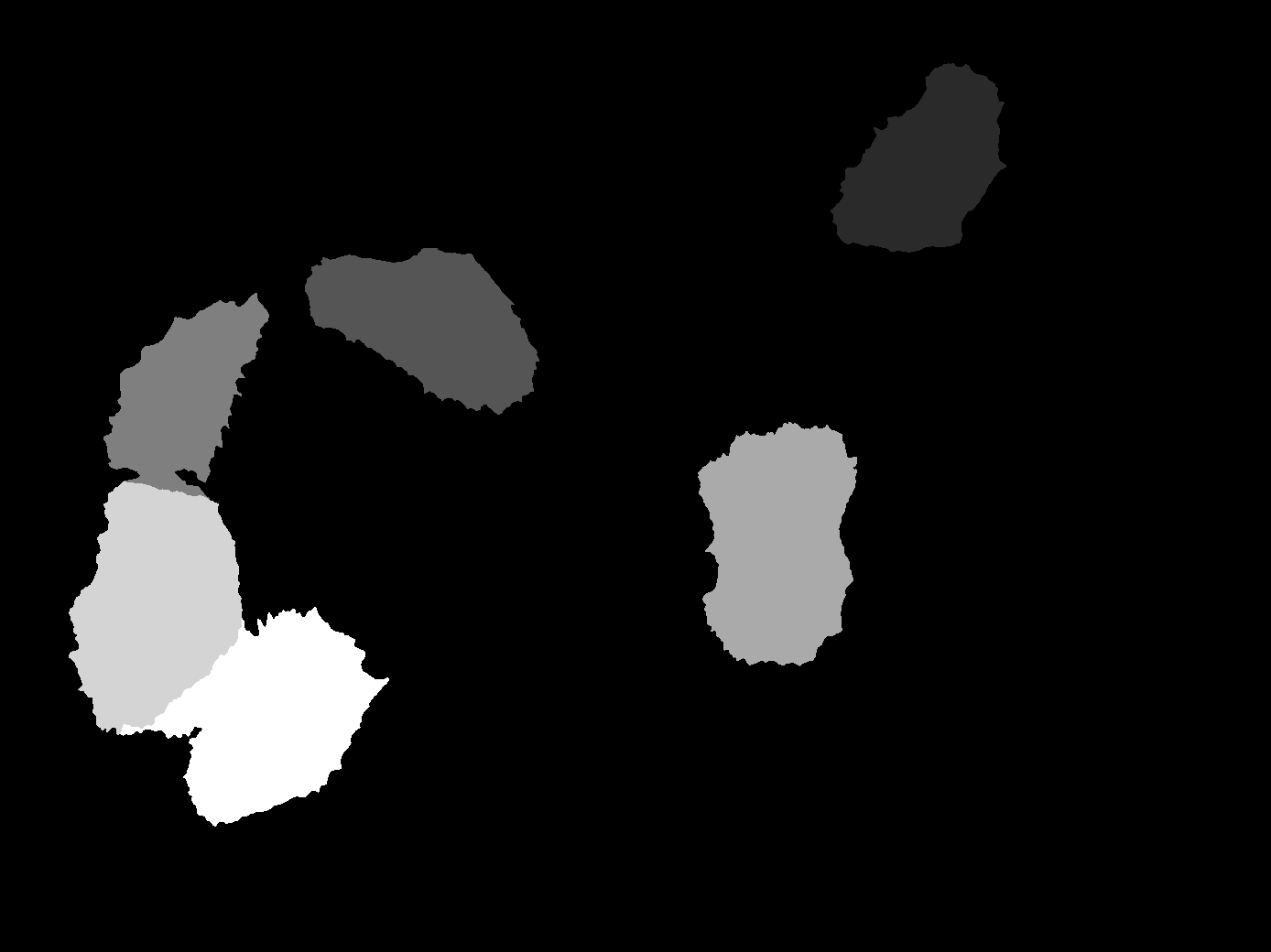

Supplement: S1 File — This file contains all scripts (CellProfiler v2.1.1 and MATLAB2016a) and data necessary to reproduce the information shown in Fig 3. (ZIP) [file pone.0180810.s001.zip › vitaminD_eColi_reproducibleResearchArchive/Results2016/A_20_c2_seg.tif]

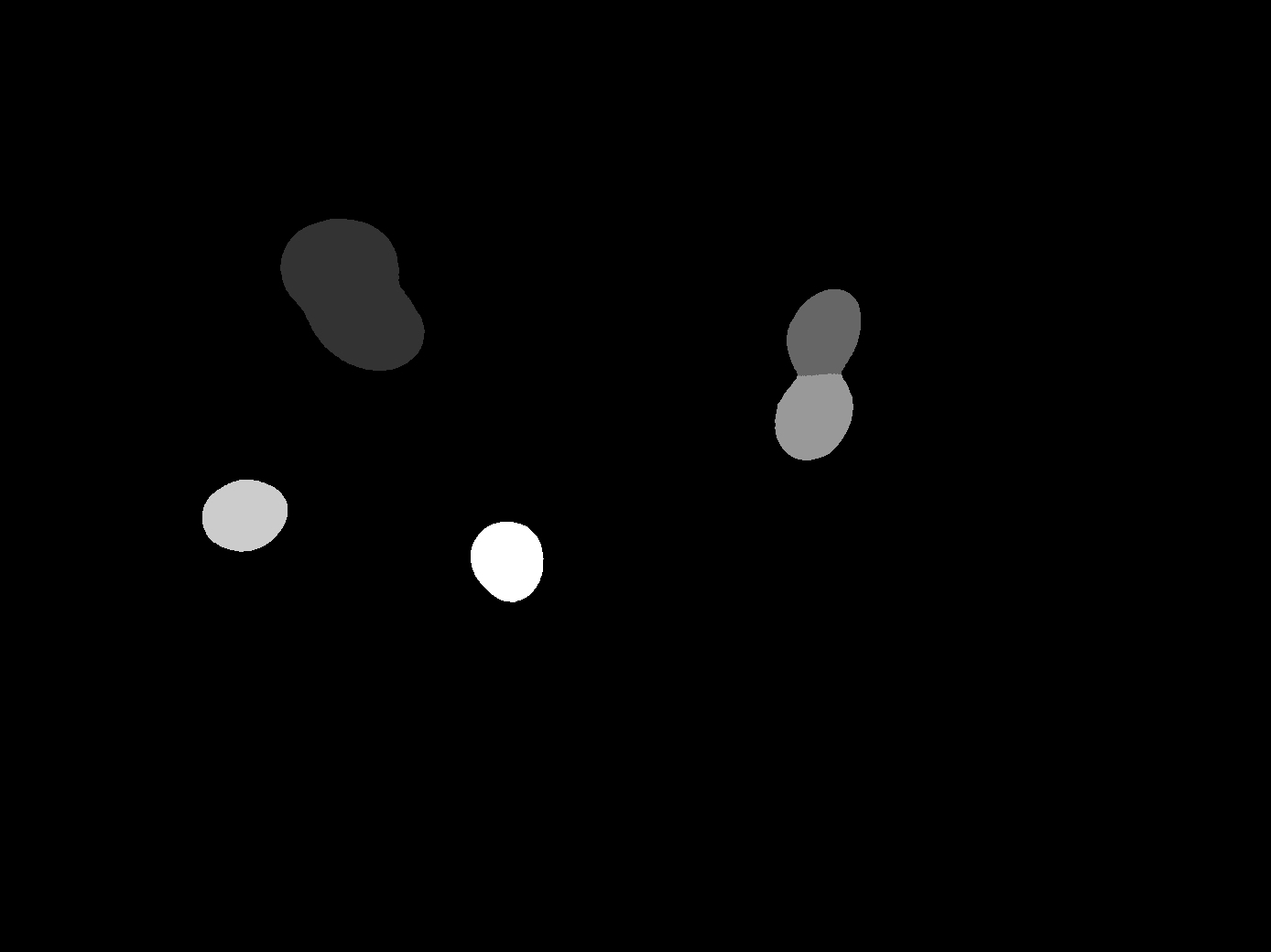

Supplement: S1 File — This file contains all scripts (CellProfiler v2.1.1 and MATLAB2016a) and data necessary to reproduce the information shown in Fig 3. (ZIP) [file pone.0180810.s001.zip › vitaminD_eColi_reproducibleResearchArchive/Results2016/A_21_c0_seg.tif]

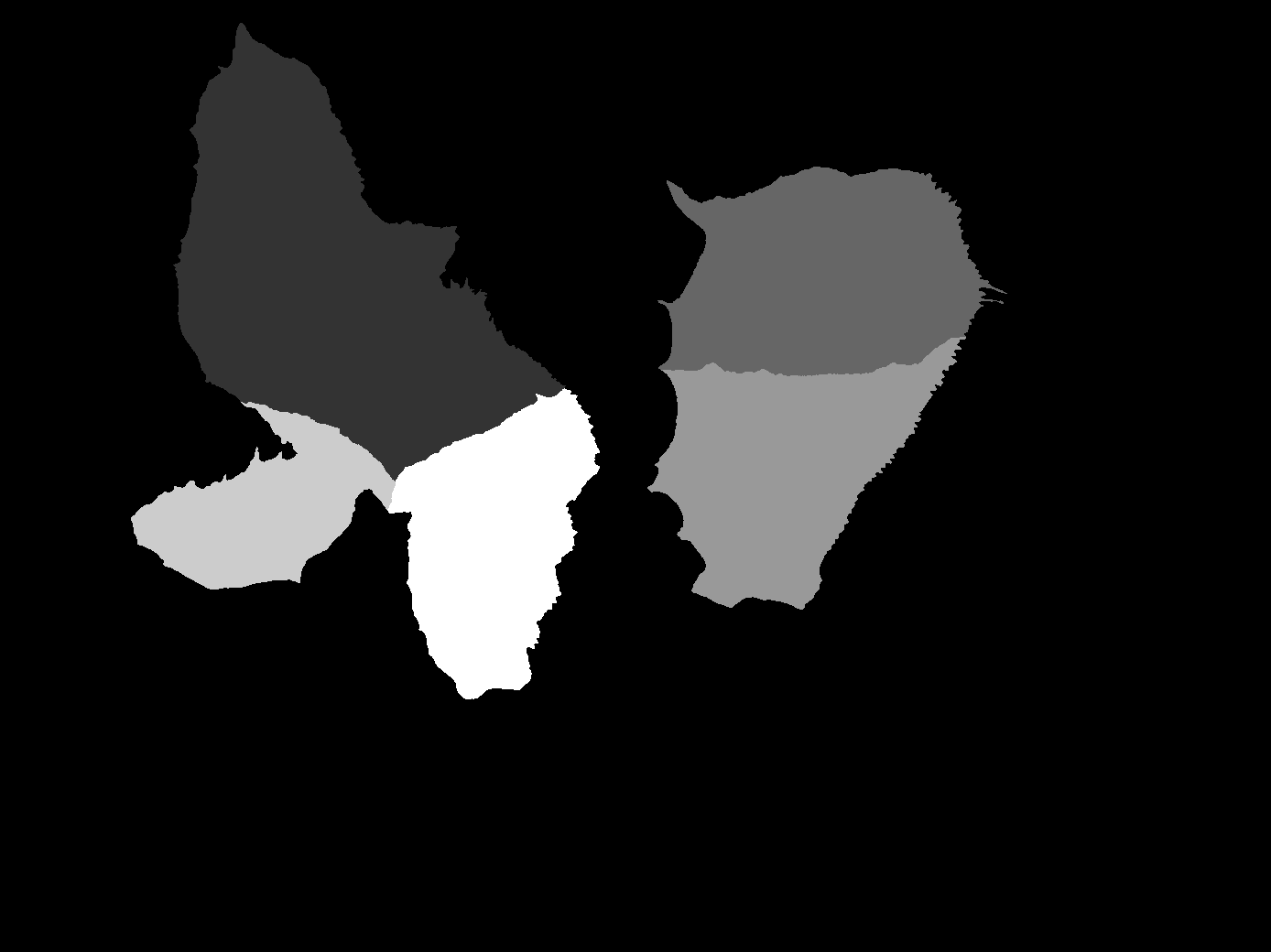

Supplement: S1 File — This file contains all scripts (CellProfiler v2.1.1 and MATLAB2016a) and data necessary to reproduce the information shown in Fig 3. (ZIP) [file pone.0180810.s001.zip › vitaminD_eColi_reproducibleResearchArchive/Results2016/A_21_c2_seg.tif]

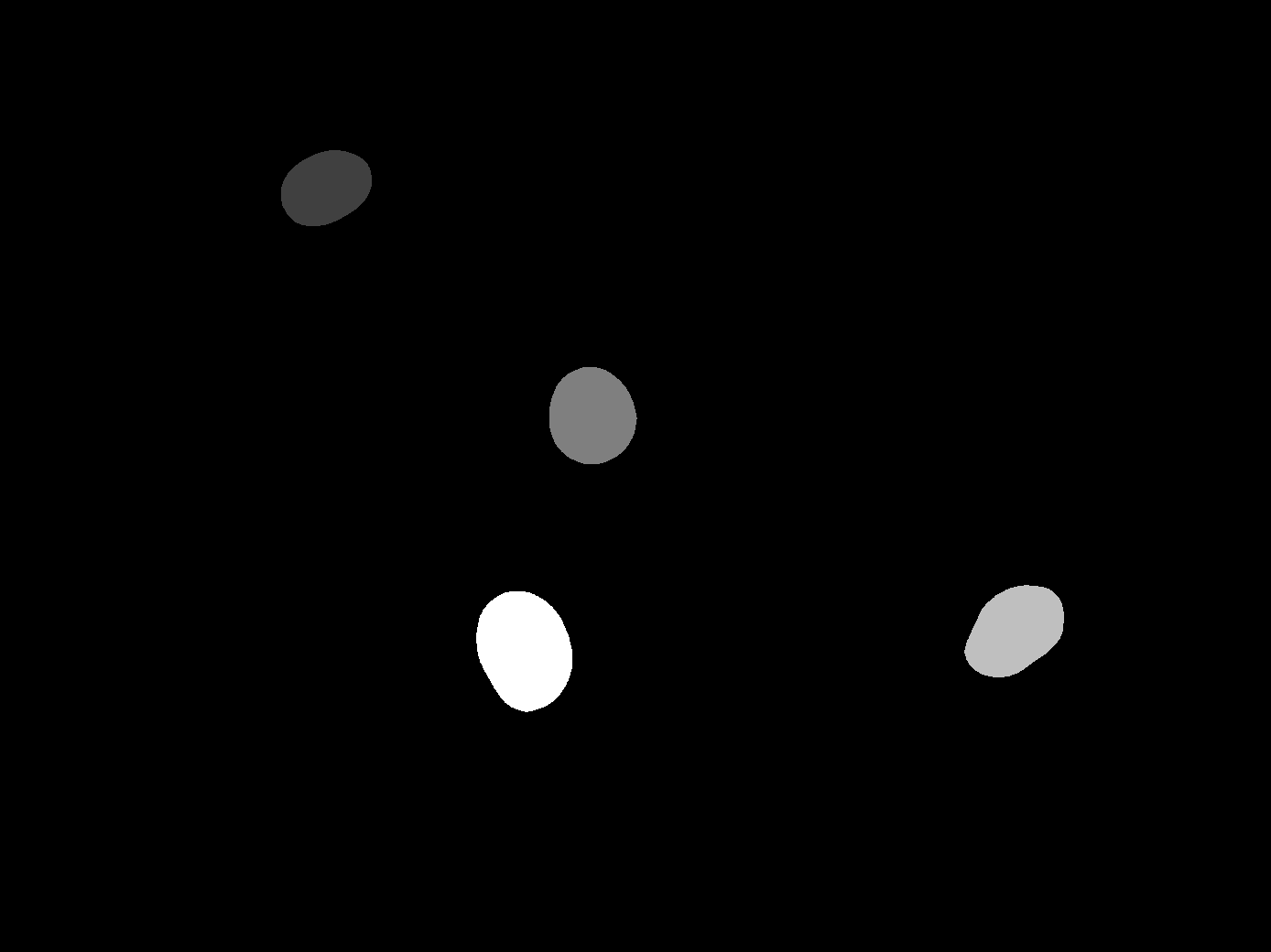

Supplement: S1 File — This file contains all scripts (CellProfiler v2.1.1 and MATLAB2016a) and data necessary to reproduce the information shown in Fig 3. (ZIP) [file pone.0180810.s001.zip › vitaminD_eColi_reproducibleResearchArchive/Results2016/A_22_c0_seg.tif]

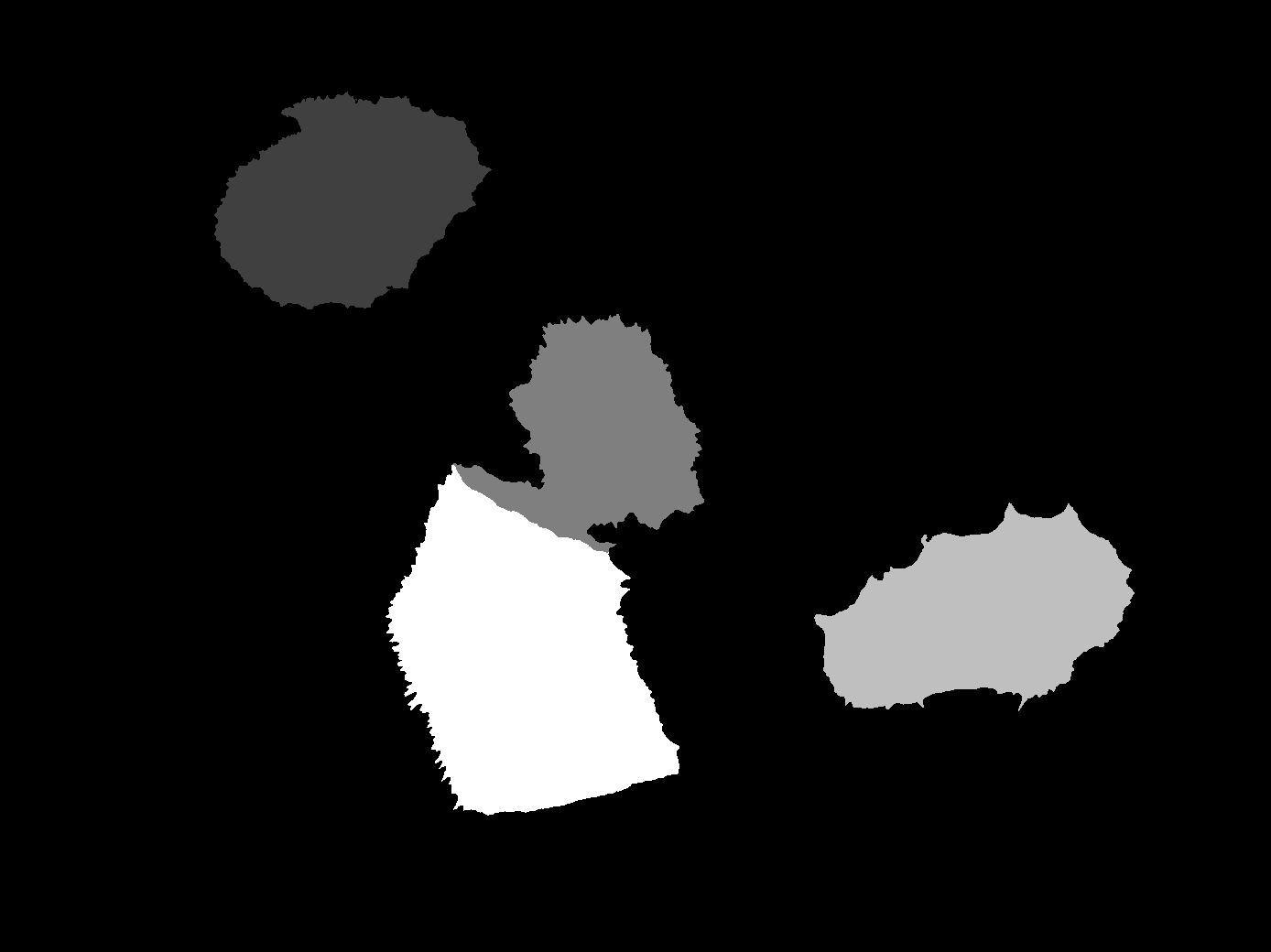

Supplement: S1 File — This file contains all scripts (CellProfiler v2.1.1 and MATLAB2016a) and data necessary to reproduce the information shown in Fig 3. (ZIP) [file pone.0180810.s001.zip › vitaminD_eColi_reproducibleResearchArchive/Results2016/A_22_c2_seg.tif]

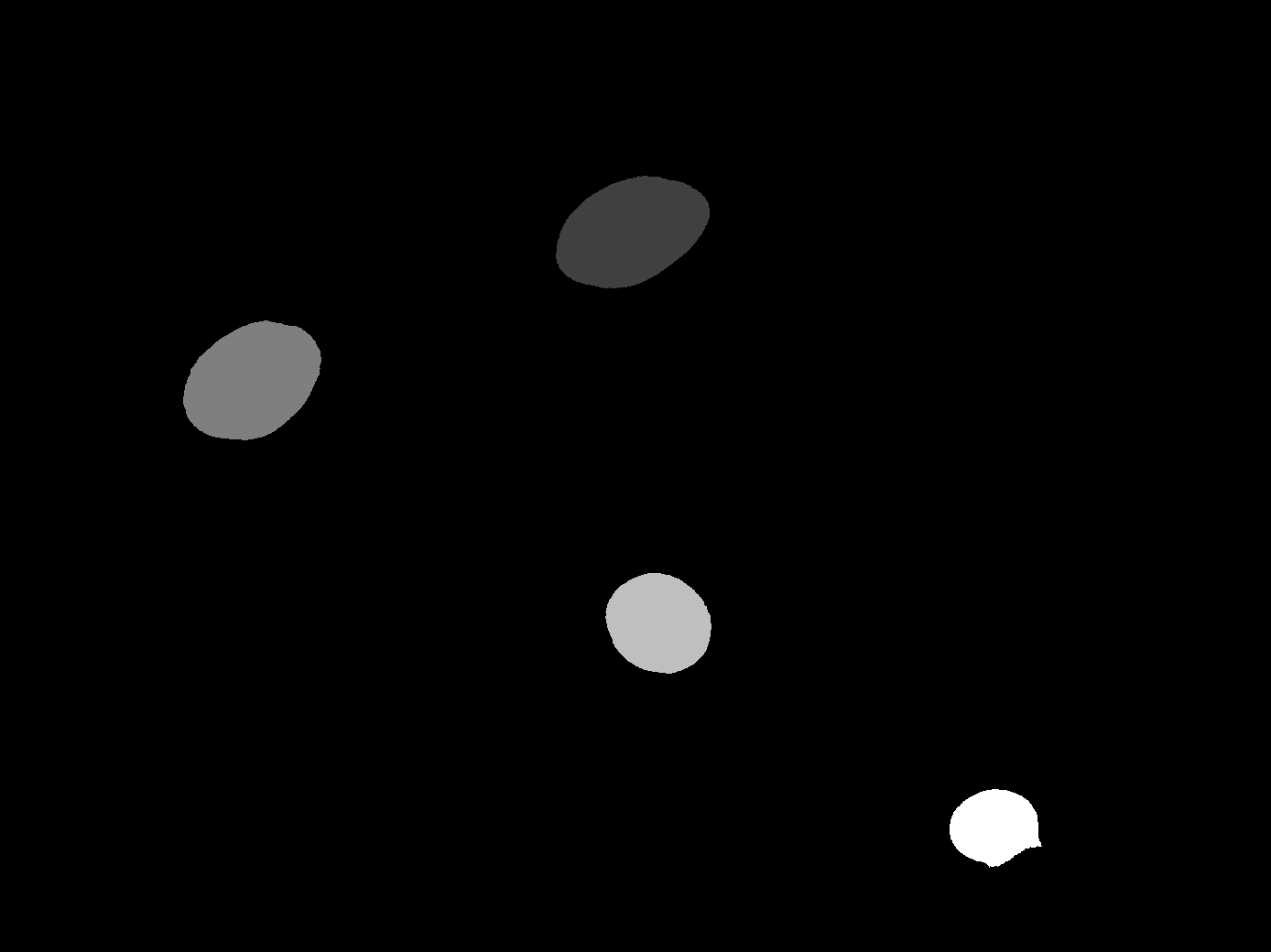

Supplement: S1 File — This file contains all scripts (CellProfiler v2.1.1 and MATLAB2016a) and data necessary to reproduce the information shown in Fig 3. (ZIP) [file pone.0180810.s001.zip › vitaminD_eColi_reproducibleResearchArchive/Results2016/A_23_c0_seg.tif]

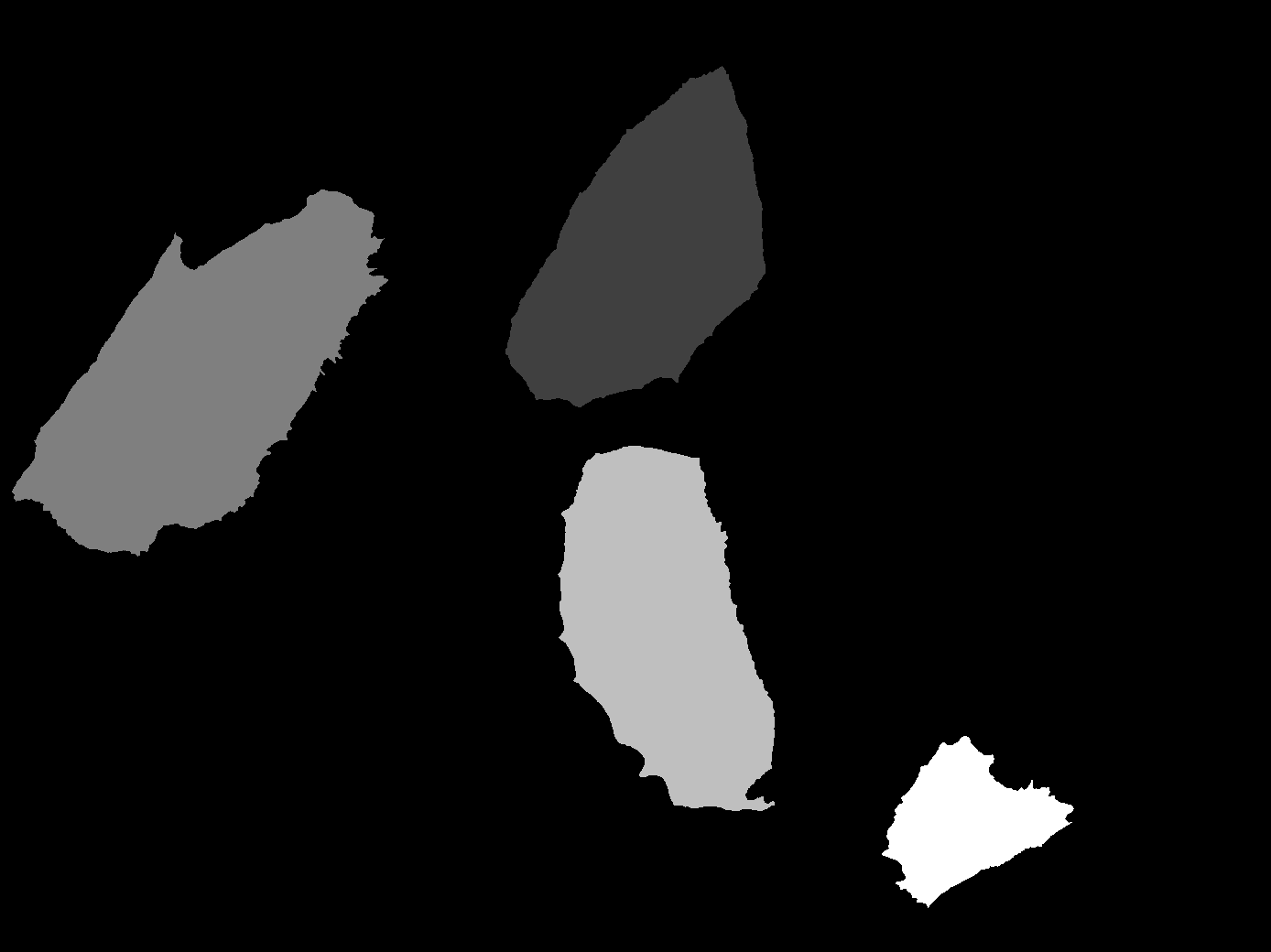

Supplement: S1 File — This file contains all scripts (CellProfiler v2.1.1 and MATLAB2016a) and data necessary to reproduce the information shown in Fig 3. (ZIP) [file pone.0180810.s001.zip › vitaminD_eColi_reproducibleResearchArchive/Results2016/A_23_c2_seg.tif]

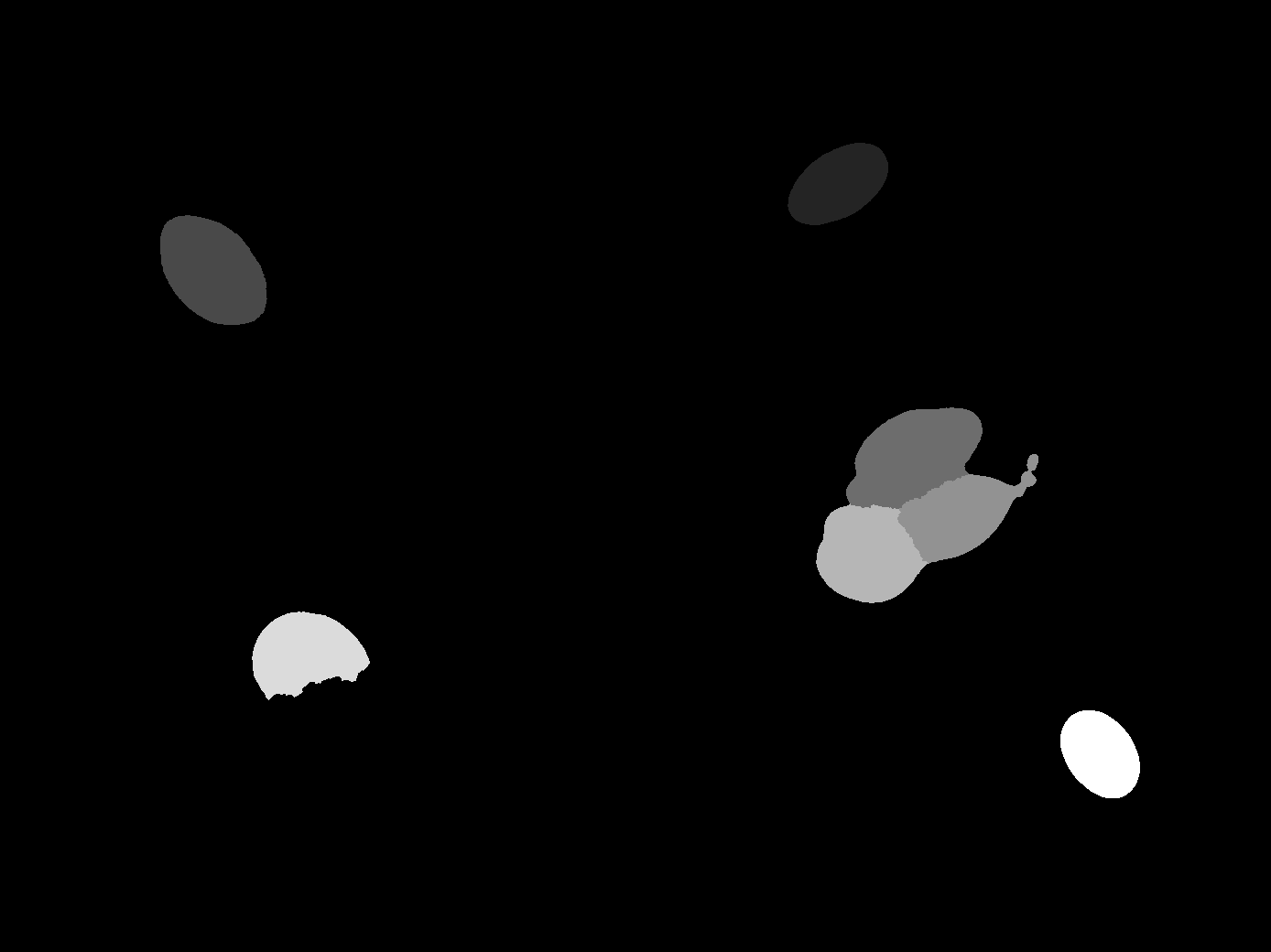

Supplement: S1 File — This file contains all scripts (CellProfiler v2.1.1 and MATLAB2016a) and data necessary to reproduce the information shown in Fig 3. (ZIP) [file pone.0180810.s001.zip › vitaminD_eColi_reproducibleResearchArchive/Results2016/A_24_c0_seg.tif]

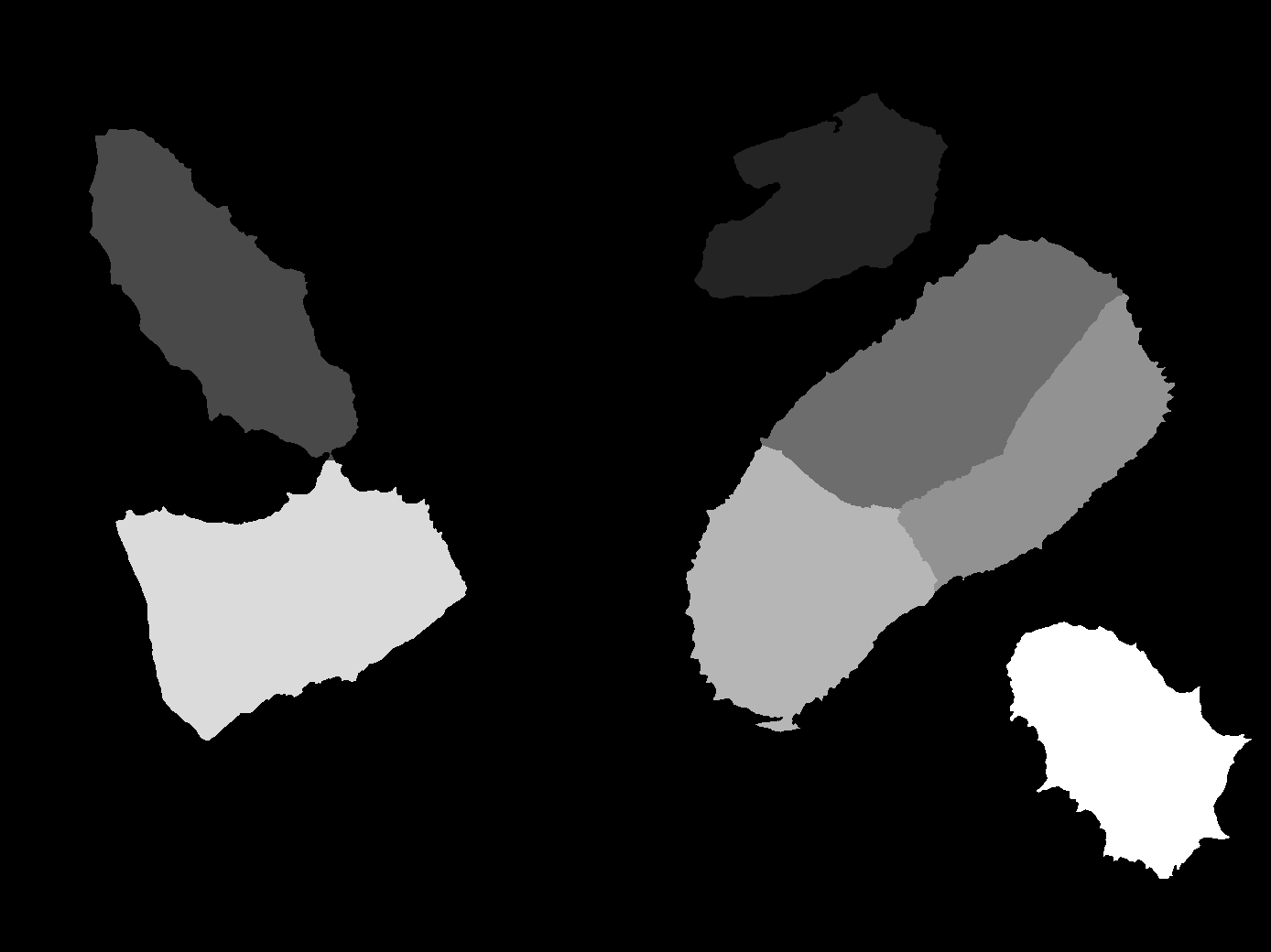

Supplement: S1 File — This file contains all scripts (CellProfiler v2.1.1 and MATLAB2016a) and data necessary to reproduce the information shown in Fig 3. (ZIP) [file pone.0180810.s001.zip › vitaminD_eColi_reproducibleResearchArchive/Results2016/A_24_c2_seg.tif]

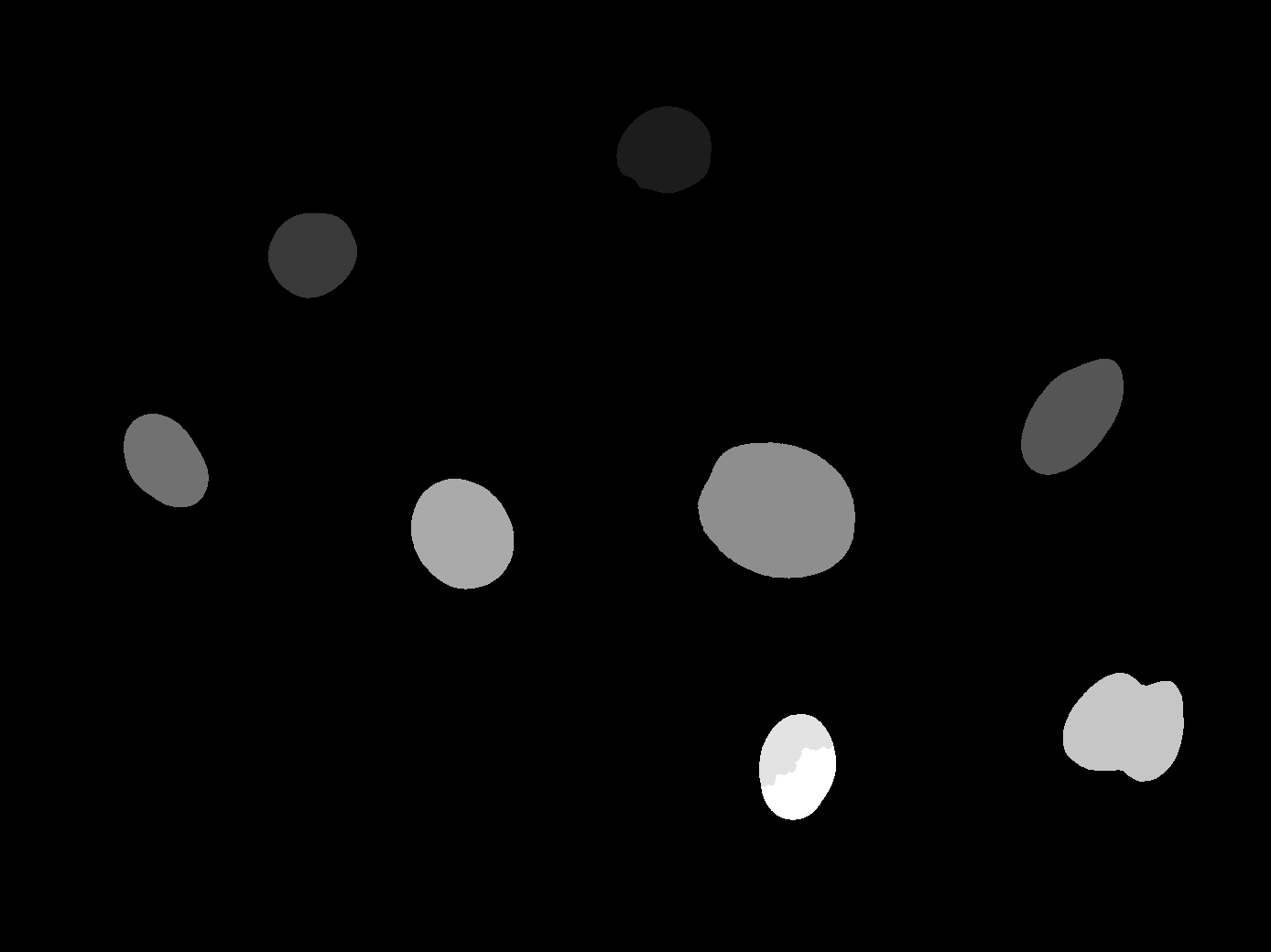

Supplement: S1 File — This file contains all scripts (CellProfiler v2.1.1 and MATLAB2016a) and data necessary to reproduce the information shown in Fig 3. (ZIP) [file pone.0180810.s001.zip › vitaminD_eColi_reproducibleResearchArchive/Results2016/A_25_c0_seg.tif]

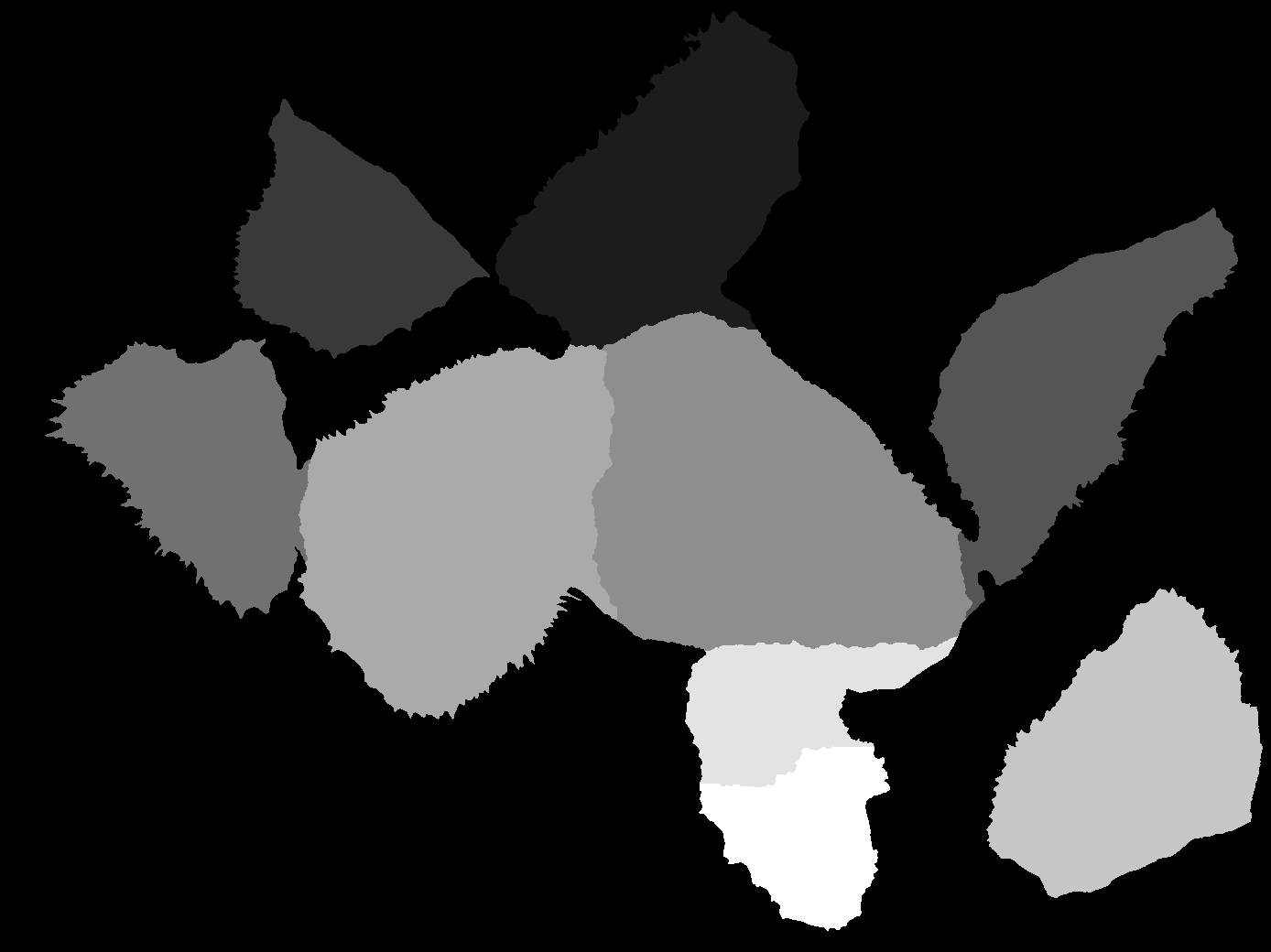

Supplement: S1 File — This file contains all scripts (CellProfiler v2.1.1 and MATLAB2016a) and data necessary to reproduce the information shown in Fig 3. (ZIP) [file pone.0180810.s001.zip › vitaminD_eColi_reproducibleResearchArchive/Results2016/A_25_c2_seg.tif]

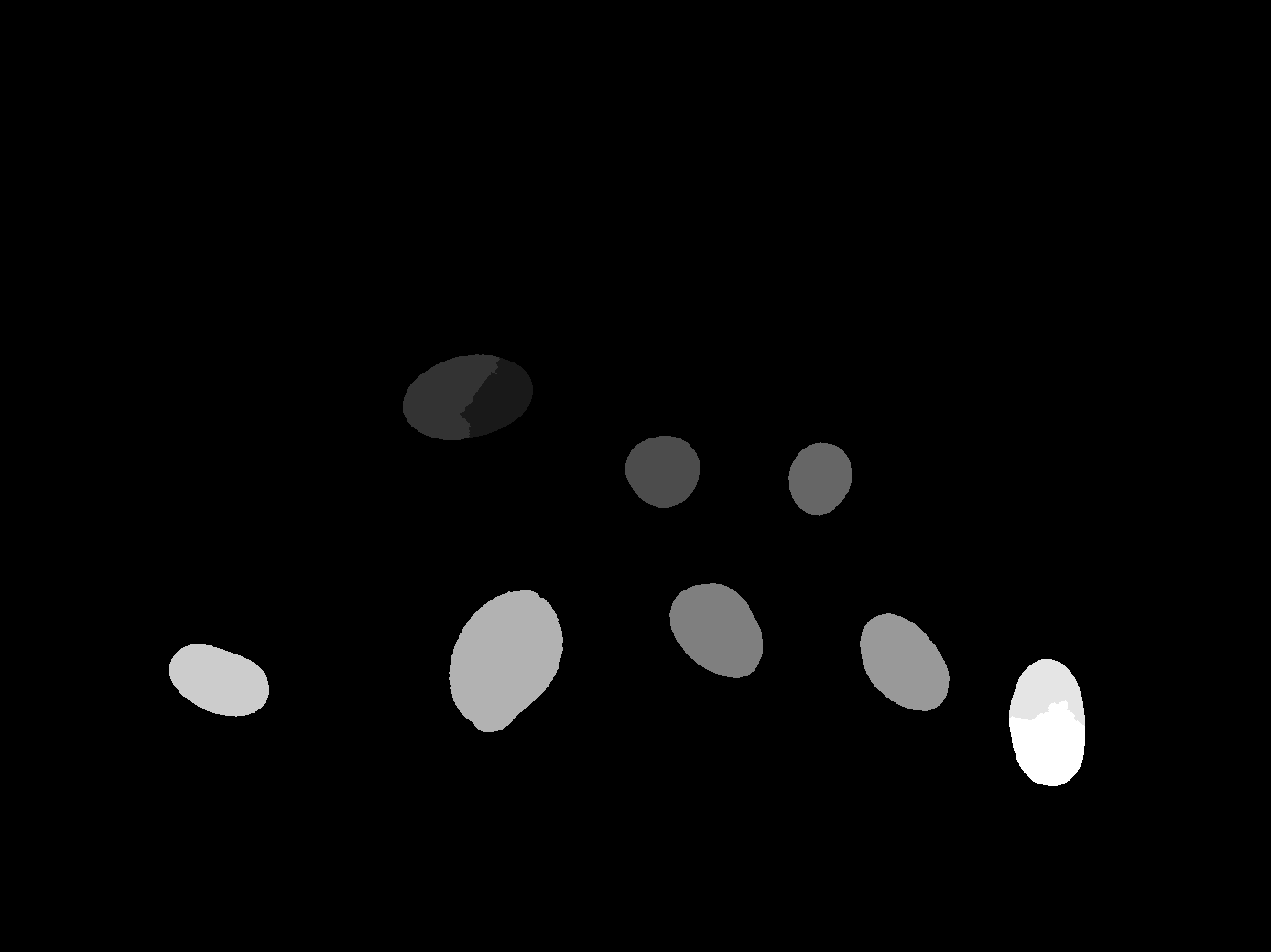

Supplement: S1 File — This file contains all scripts (CellProfiler v2.1.1 and MATLAB2016a) and data necessary to reproduce the information shown in Fig 3. (ZIP) [file pone.0180810.s001.zip › vitaminD_eColi_reproducibleResearchArchive/Results2016/A_26_c0_seg.tif]

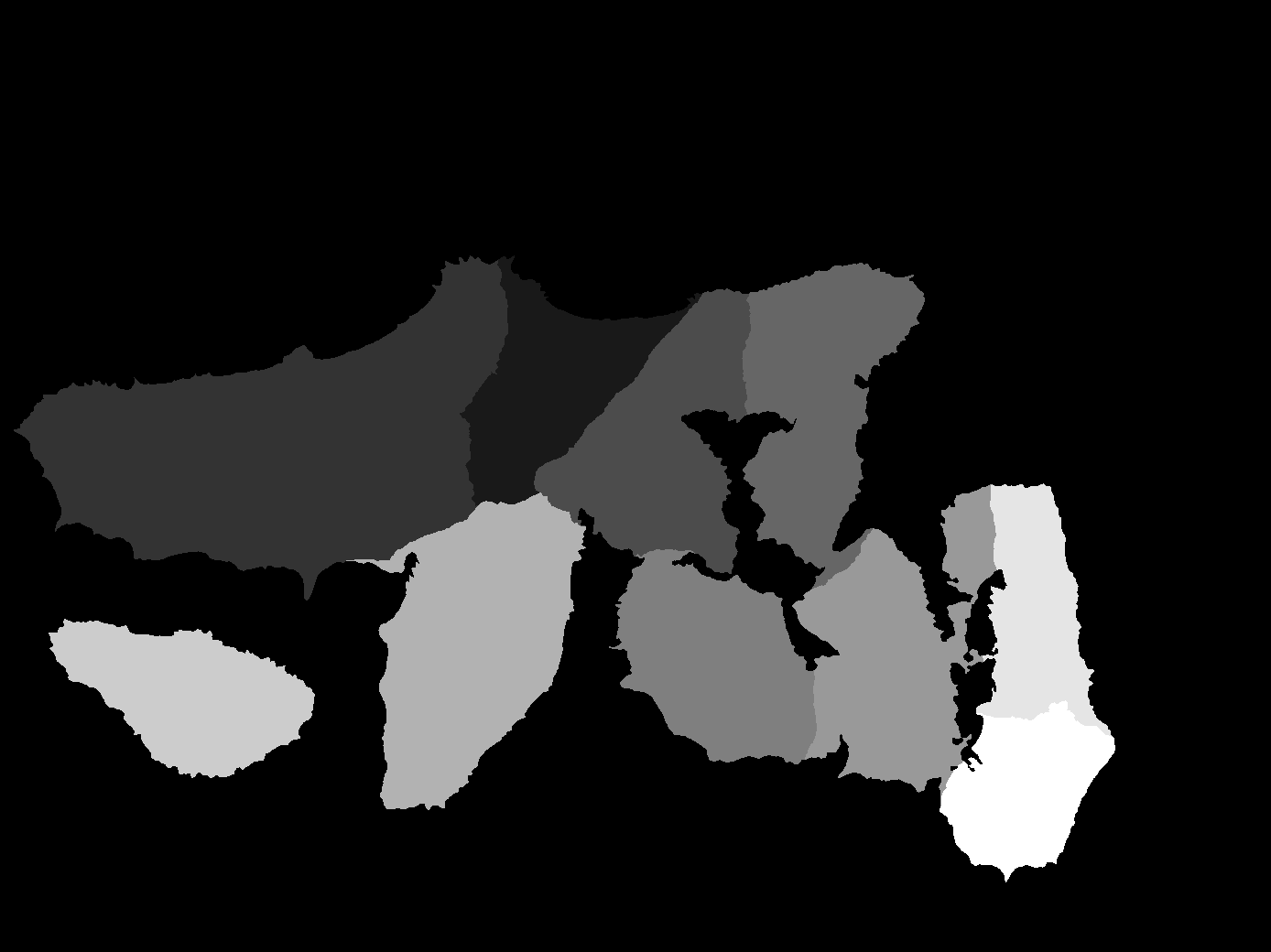

Supplement: S1 File — This file contains all scripts (CellProfiler v2.1.1 and MATLAB2016a) and data necessary to reproduce the information shown in Fig 3. (ZIP) [file pone.0180810.s001.zip › vitaminD_eColi_reproducibleResearchArchive/Results2016/A_26_c2_seg.tif]

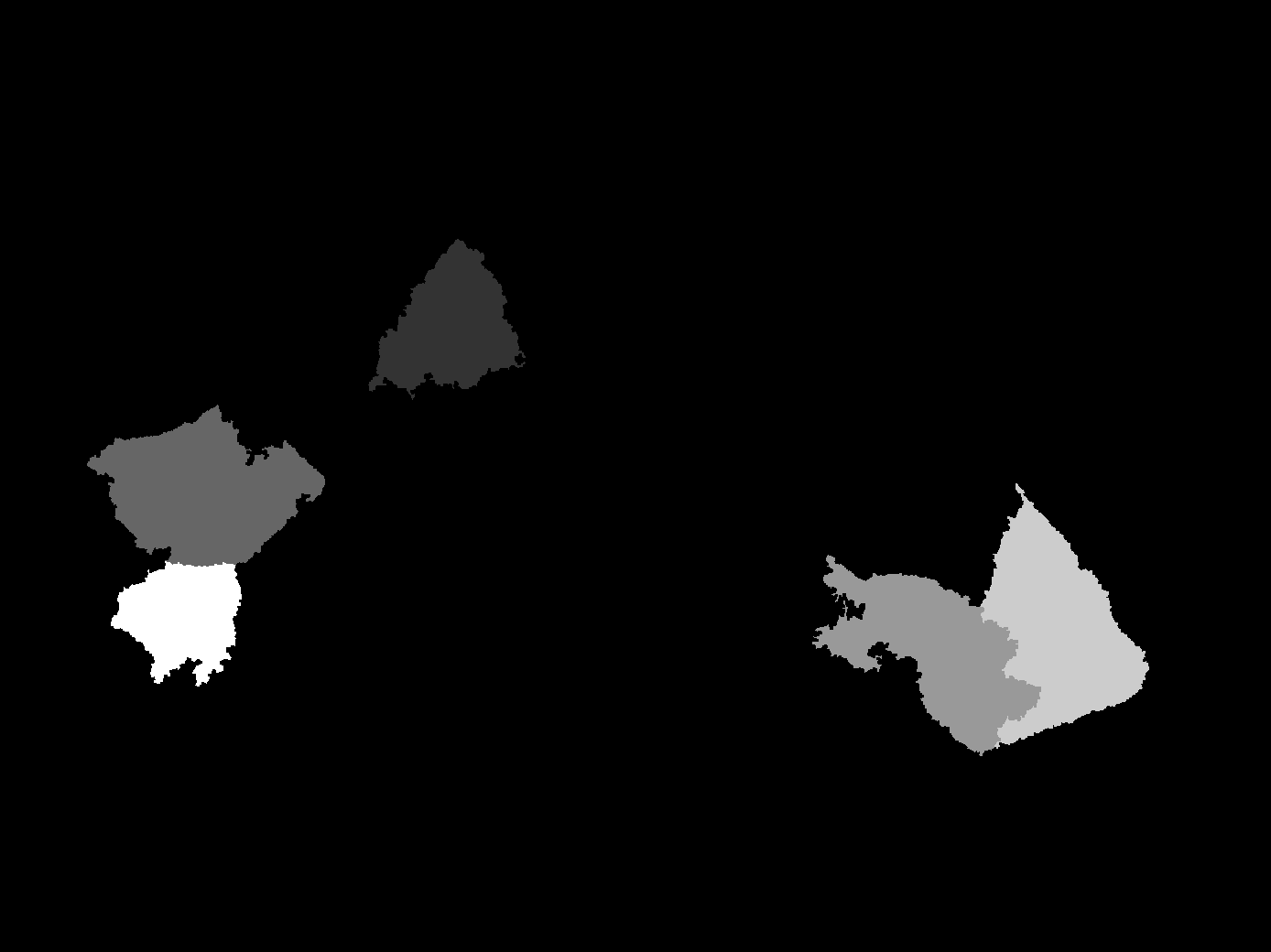

Supplement: S1 File — This file contains all scripts (CellProfiler v2.1.1 and MATLAB2016a) and data necessary to reproduce the information shown in Fig 3. (ZIP) [file pone.0180810.s001.zip › vitaminD_eColi_reproducibleResearchArchive/Results2016/A_27_c0_seg.tif]

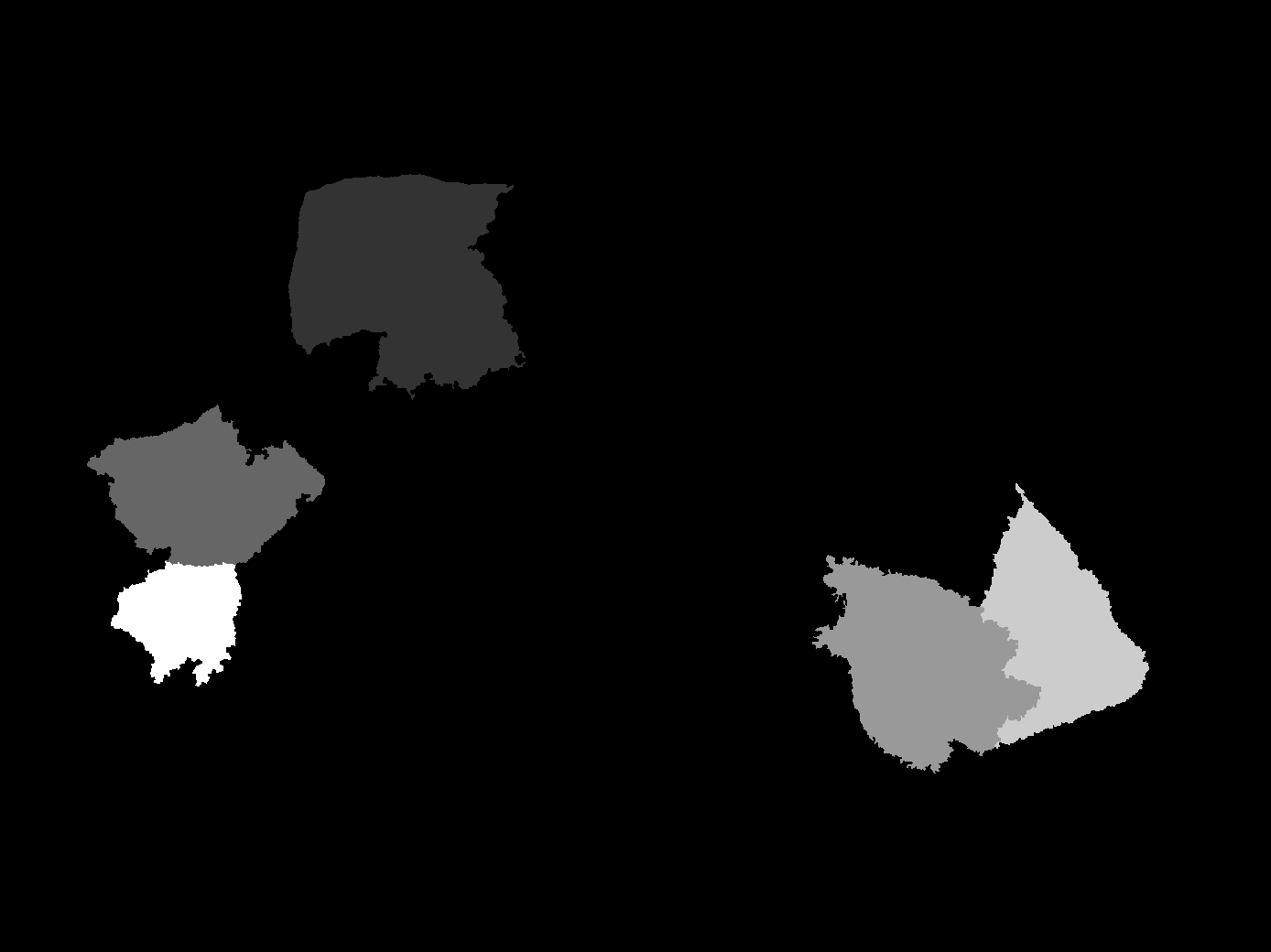

Supplement: S1 File — This file contains all scripts (CellProfiler v2.1.1 and MATLAB2016a) and data necessary to reproduce the information shown in Fig 3. (ZIP) [file pone.0180810.s001.zip › vitaminD_eColi_reproducibleResearchArchive/Results2016/A_27_c2_seg.tif]

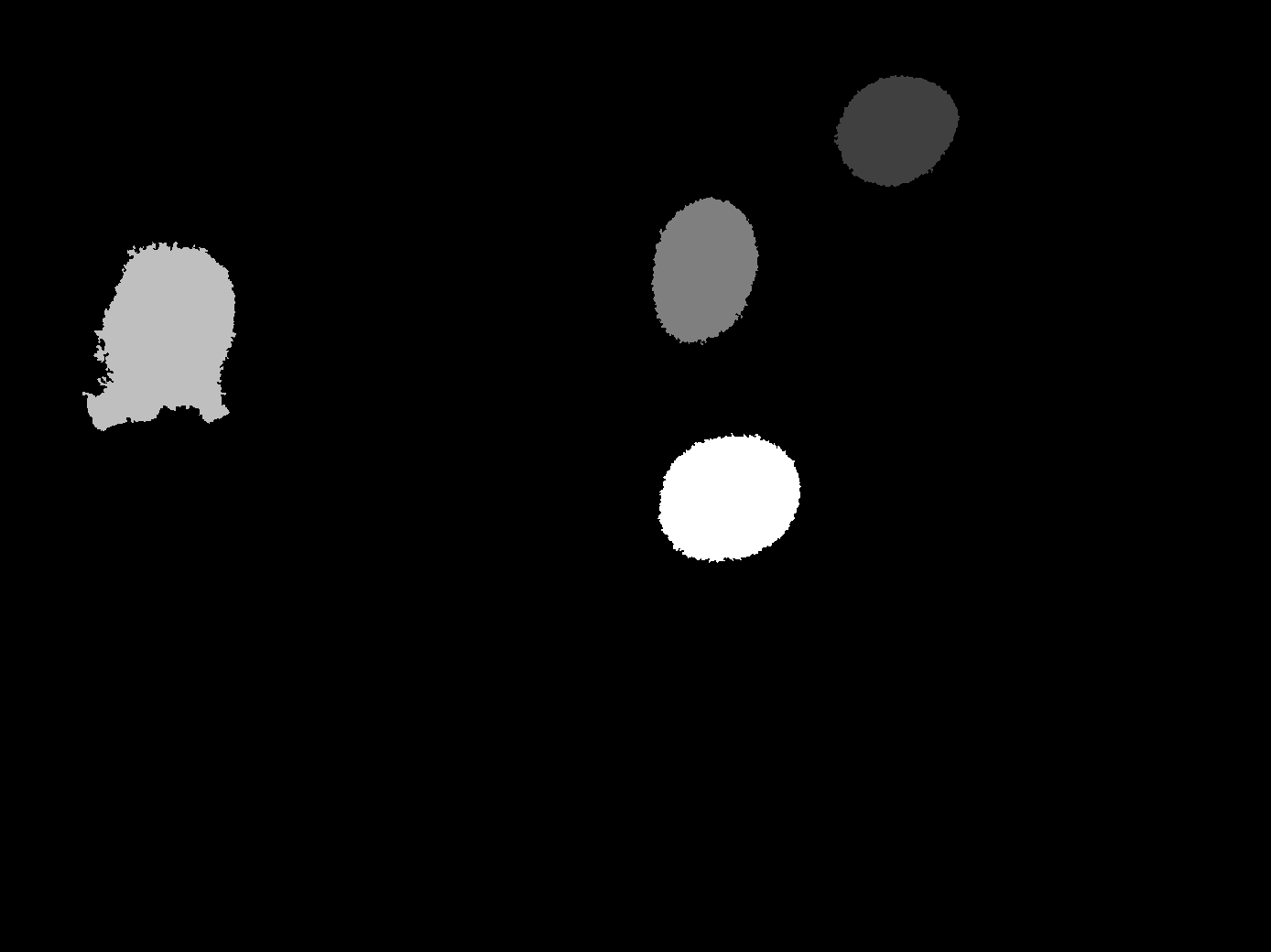

Supplement: S1 File — This file contains all scripts (CellProfiler v2.1.1 and MATLAB2016a) and data necessary to reproduce the information shown in Fig 3. (ZIP) [file pone.0180810.s001.zip › vitaminD_eColi_reproducibleResearchArchive/Results2016/A_28_c0_seg.tif]

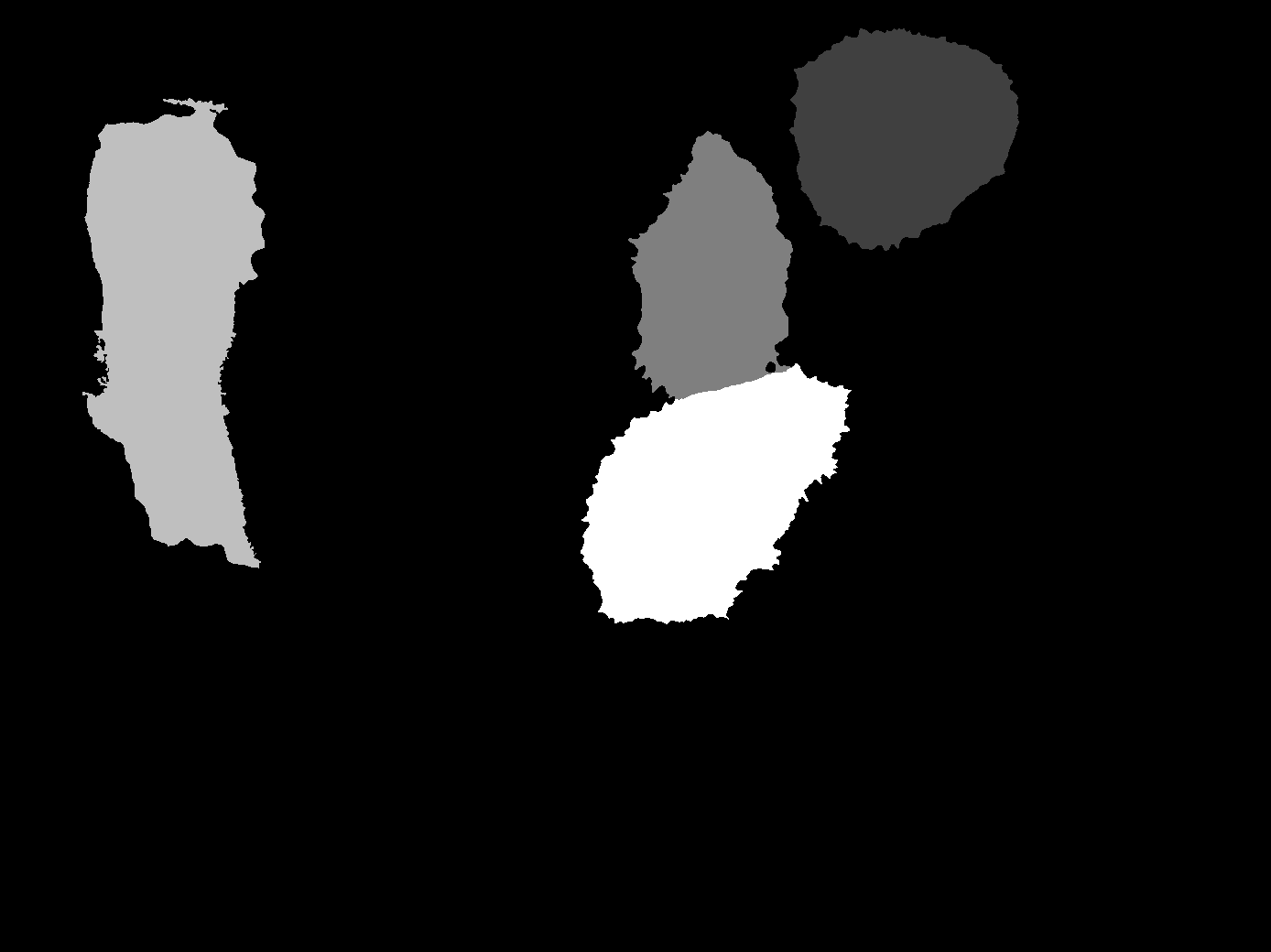

Supplement: S1 File — This file contains all scripts (CellProfiler v2.1.1 and MATLAB2016a) and data necessary to reproduce the information shown in Fig 3. (ZIP) [file pone.0180810.s001.zip › vitaminD_eColi_reproducibleResearchArchive/Results2016/A_28_c2_seg.tif]

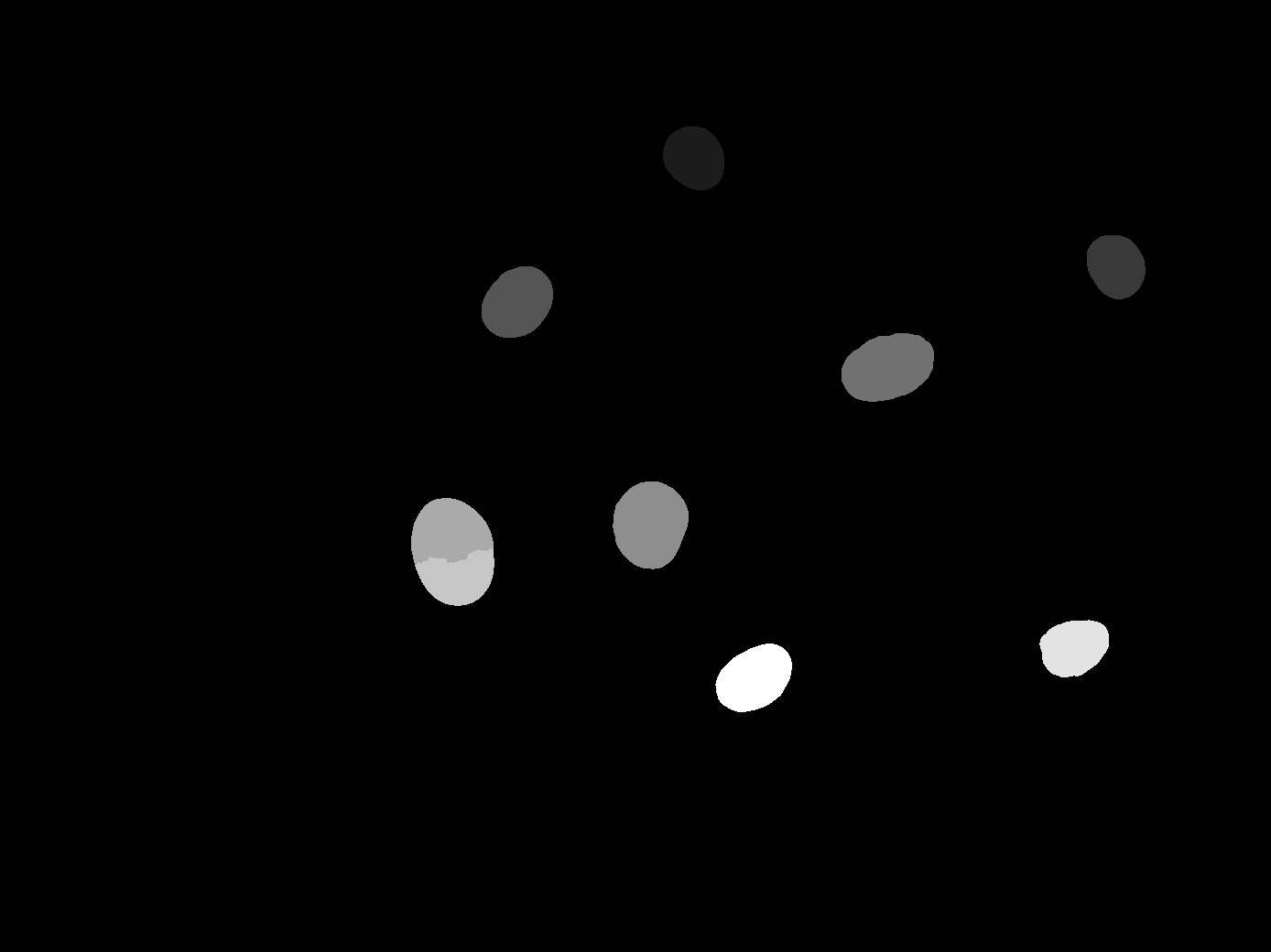

Supplement: S1 File — This file contains all scripts (CellProfiler v2.1.1 and MATLAB2016a) and data necessary to reproduce the information shown in Fig 3. (ZIP) [file pone.0180810.s001.zip › vitaminD_eColi_reproducibleResearchArchive/Results2016/A_29_c0_seg.tif]

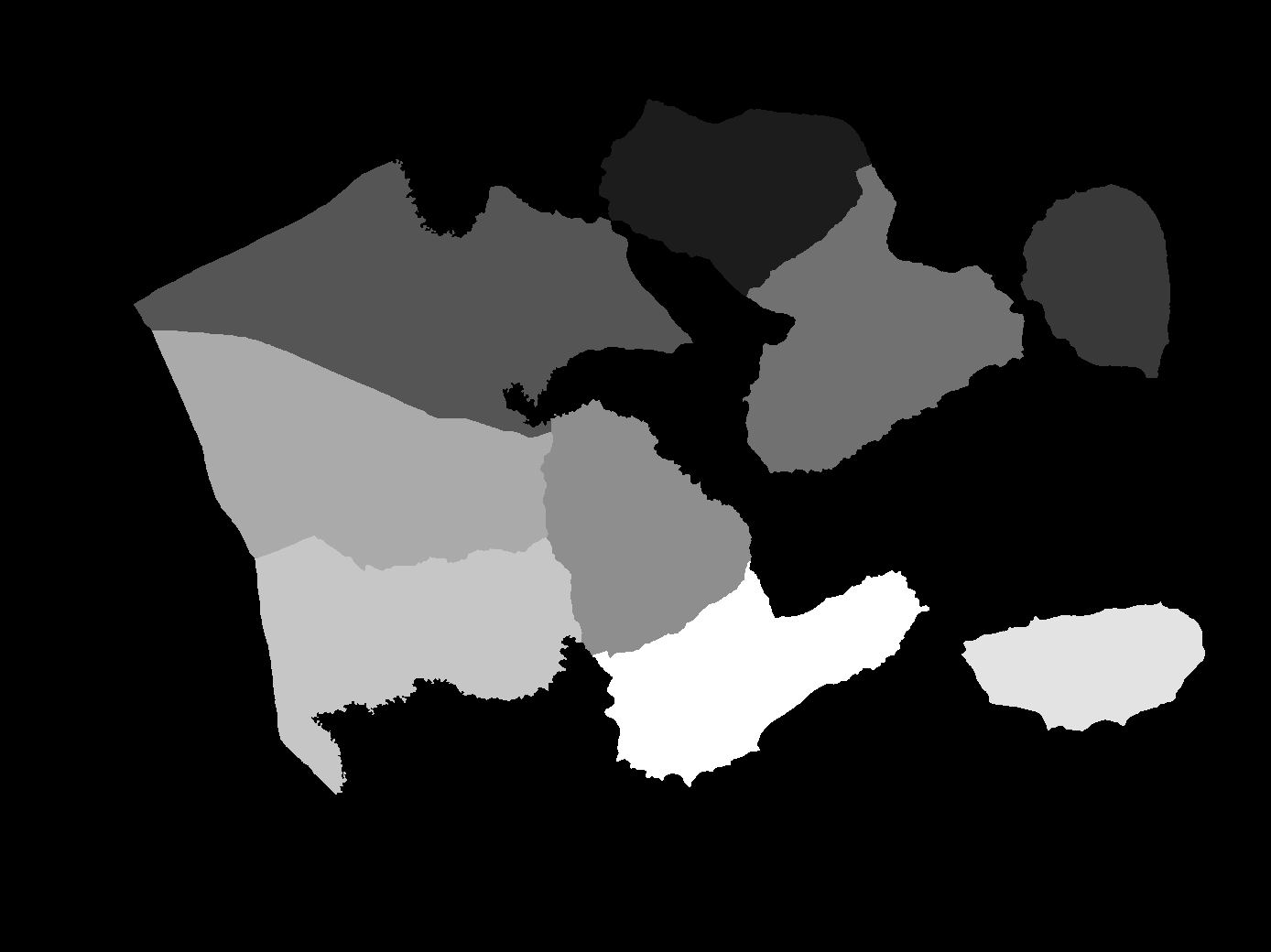

Supplement: S1 File — This file contains all scripts (CellProfiler v2.1.1 and MATLAB2016a) and data necessary to reproduce the information shown in Fig 3. (ZIP) [file pone.0180810.s001.zip › vitaminD_eColi_reproducibleResearchArchive/Results2016/A_29_c2_seg.tif]

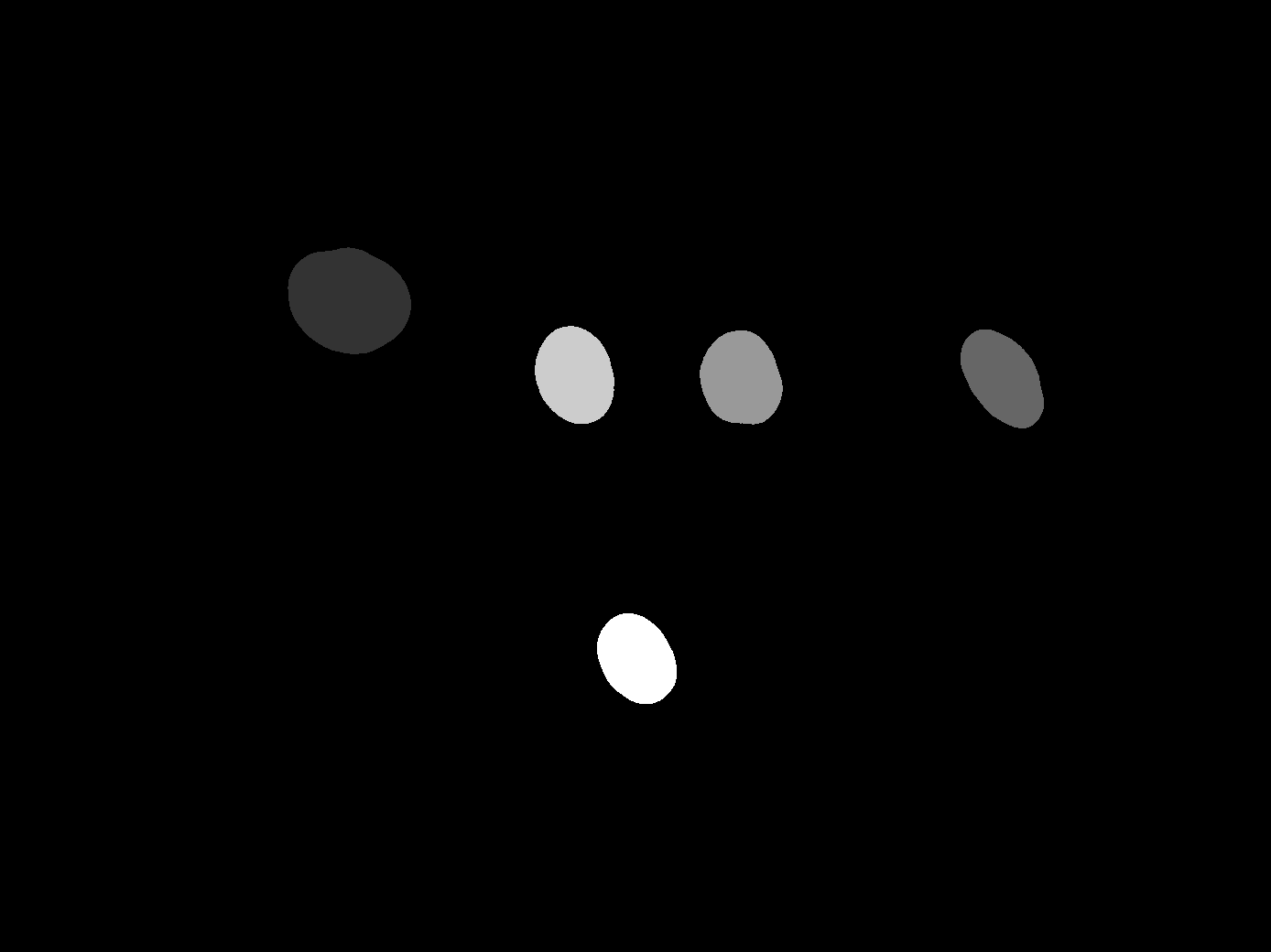

Supplement: S1 File — This file contains all scripts (CellProfiler v2.1.1 and MATLAB2016a) and data necessary to reproduce the information shown in Fig 3. (ZIP) [file pone.0180810.s001.zip › vitaminD_eColi_reproducibleResearchArchive/Results2016/A_2_c0_seg.tif]

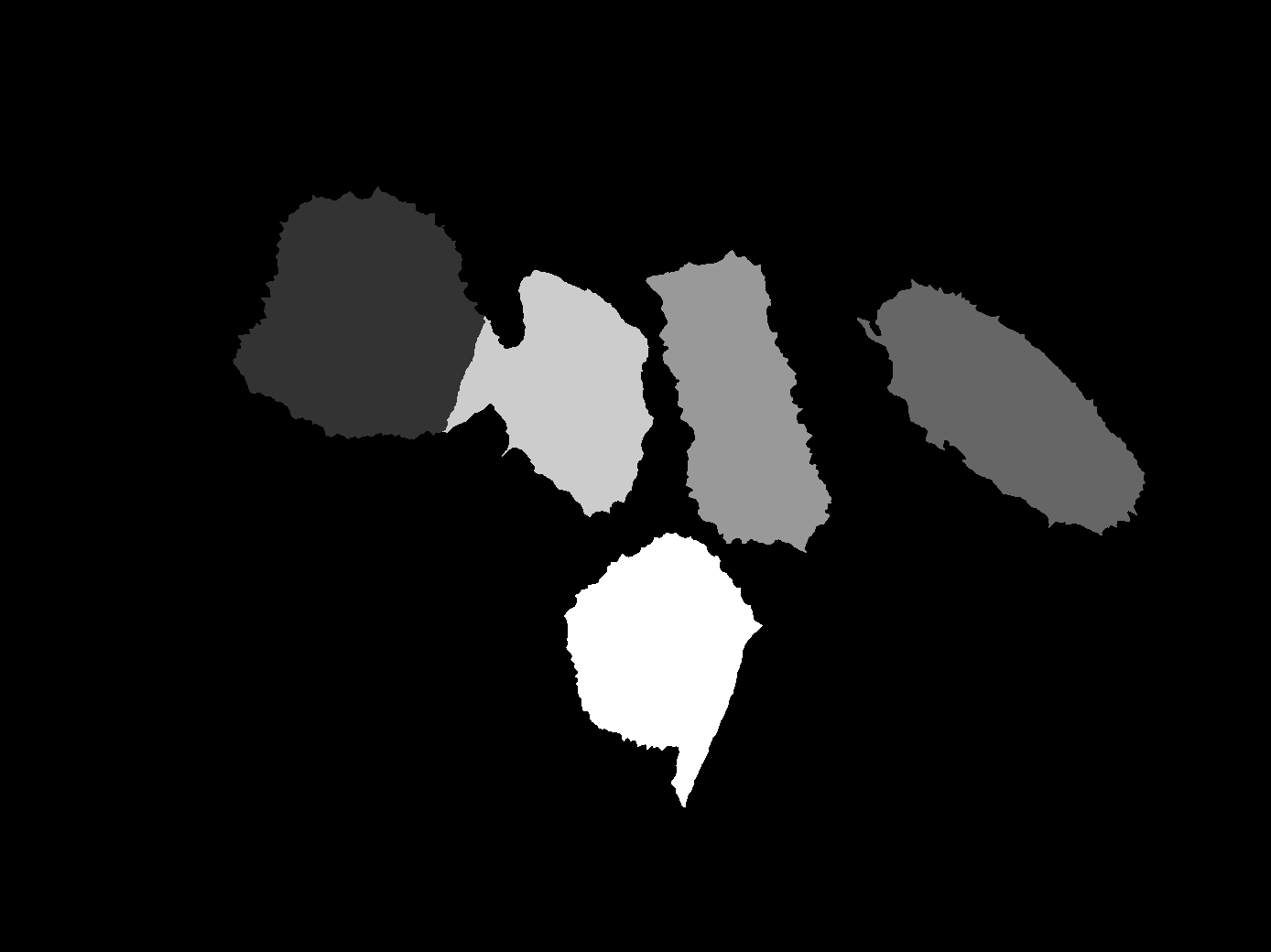

Supplement: S1 File — This file contains all scripts (CellProfiler v2.1.1 and MATLAB2016a) and data necessary to reproduce the information shown in Fig 3. (ZIP) [file pone.0180810.s001.zip › vitaminD_eColi_reproducibleResearchArchive/Results2016/A_2_c2_seg.tif]

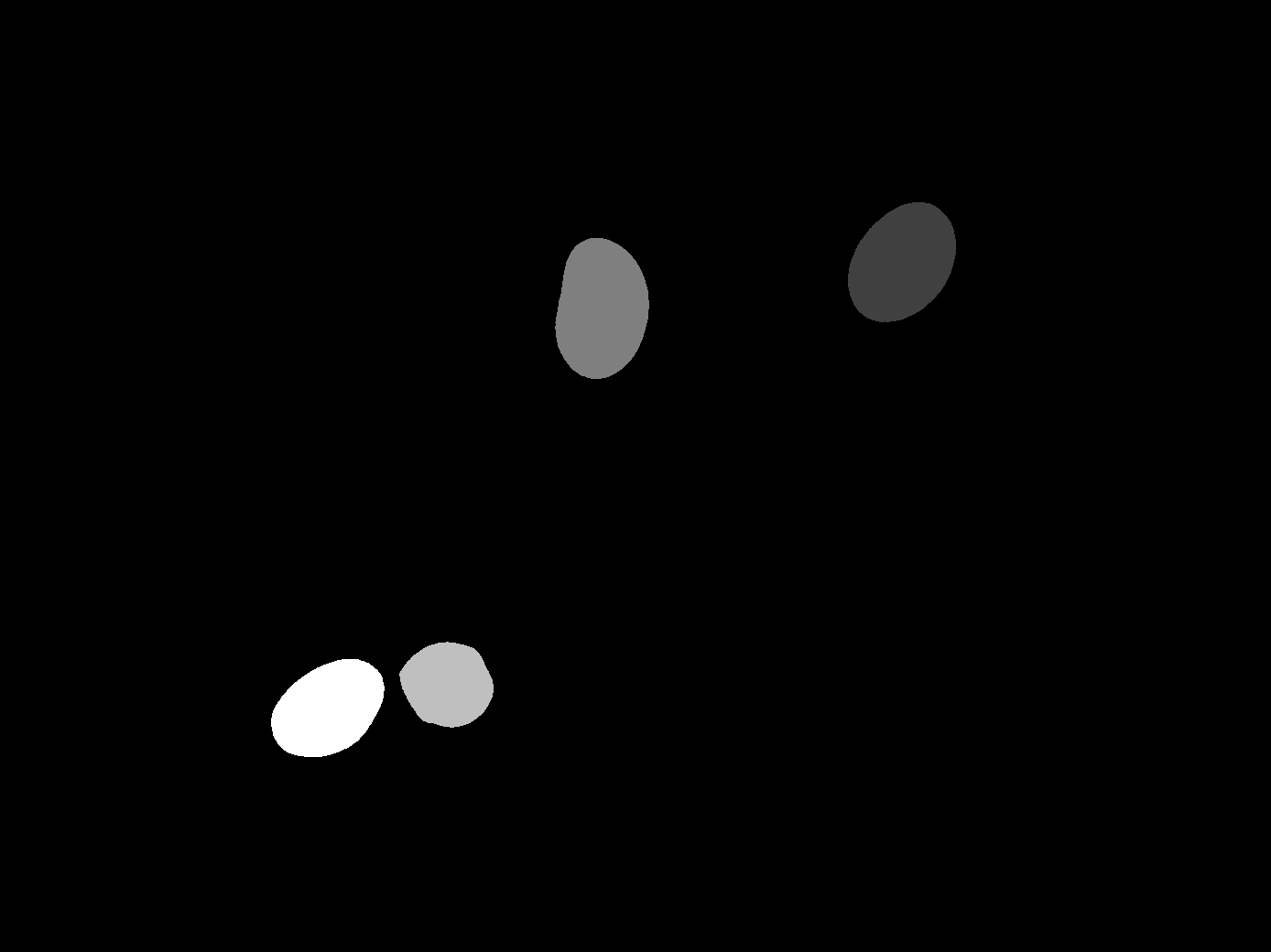

Supplement: S1 File — This file contains all scripts (CellProfiler v2.1.1 and MATLAB2016a) and data necessary to reproduce the information shown in Fig 3. (ZIP) [file pone.0180810.s001.zip › vitaminD_eColi_reproducibleResearchArchive/Results2016/A_30_c0_seg.tif]

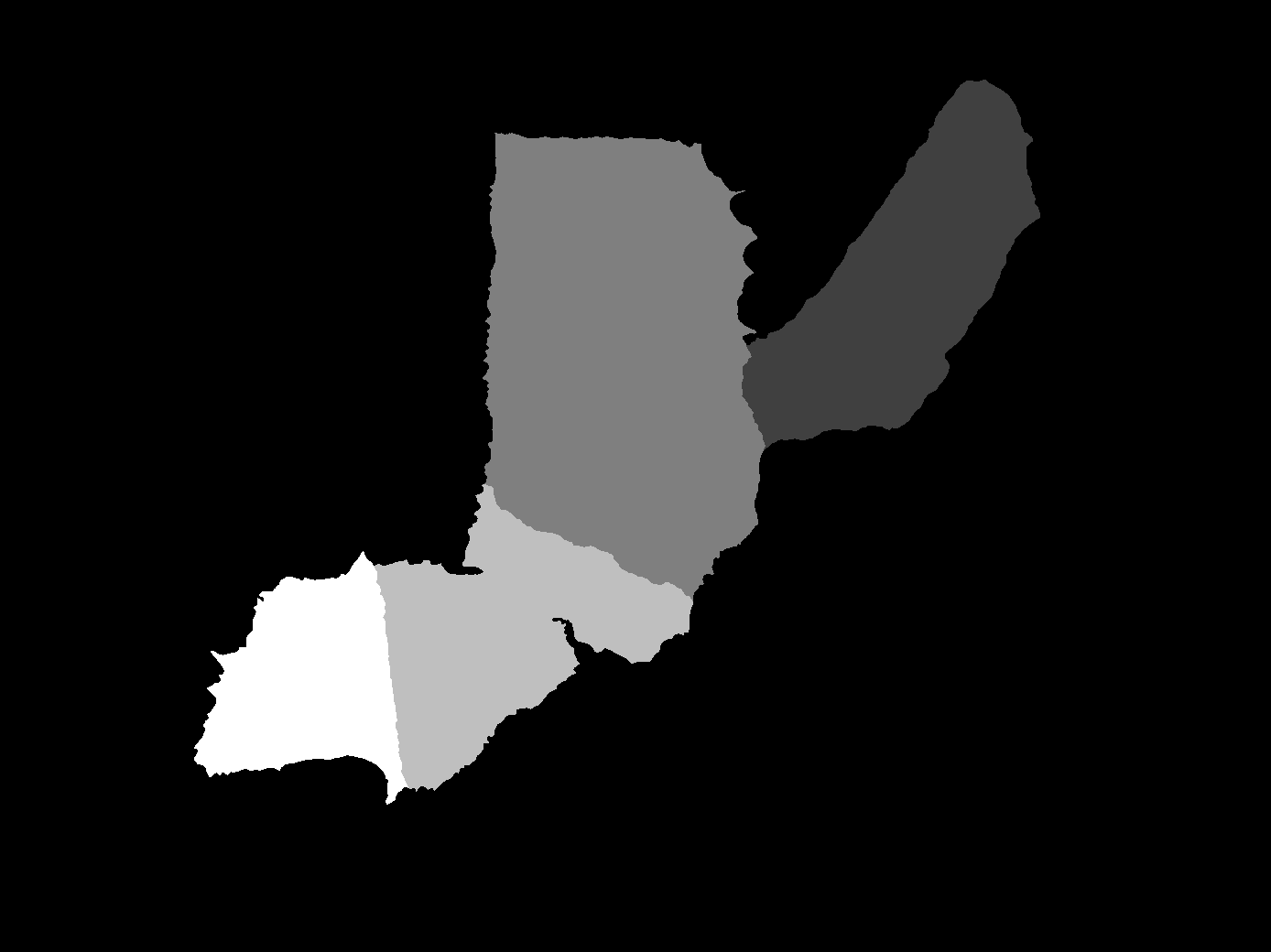

Supplement: S1 File — This file contains all scripts (CellProfiler v2.1.1 and MATLAB2016a) and data necessary to reproduce the information shown in Fig 3. (ZIP) [file pone.0180810.s001.zip › vitaminD_eColi_reproducibleResearchArchive/Results2016/A_30_c2_seg.tif]

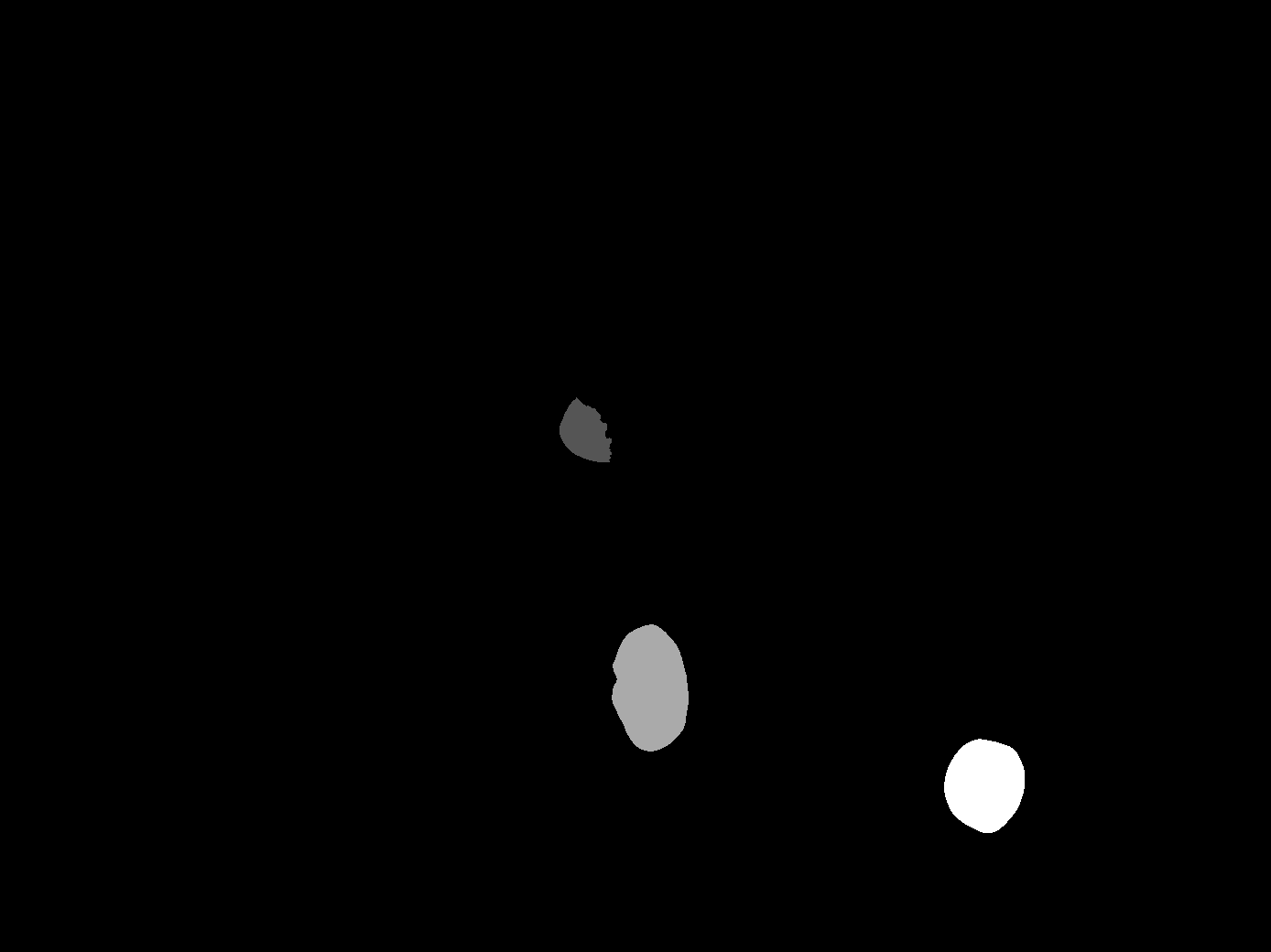

Supplement: S1 File — This file contains all scripts (CellProfiler v2.1.1 and MATLAB2016a) and data necessary to reproduce the information shown in Fig 3. (ZIP) [file pone.0180810.s001.zip › vitaminD_eColi_reproducibleResearchArchive/Results2016/A_31_c0_seg.tif]

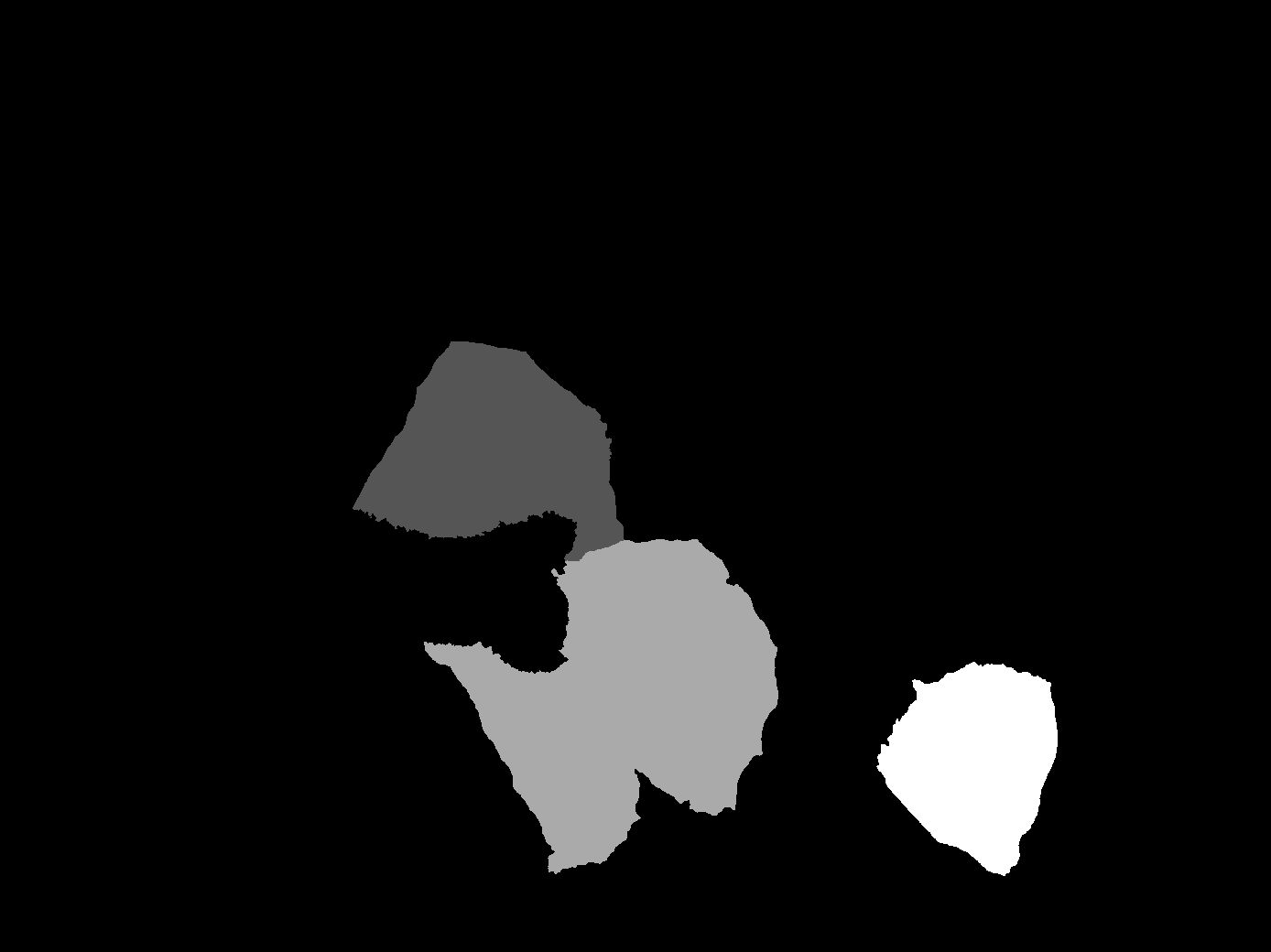

Supplement: S1 File — This file contains all scripts (CellProfiler v2.1.1 and MATLAB2016a) and data necessary to reproduce the information shown in Fig 3. (ZIP) [file pone.0180810.s001.zip › vitaminD_eColi_reproducibleResearchArchive/Results2016/A_31_c2_seg.tif]

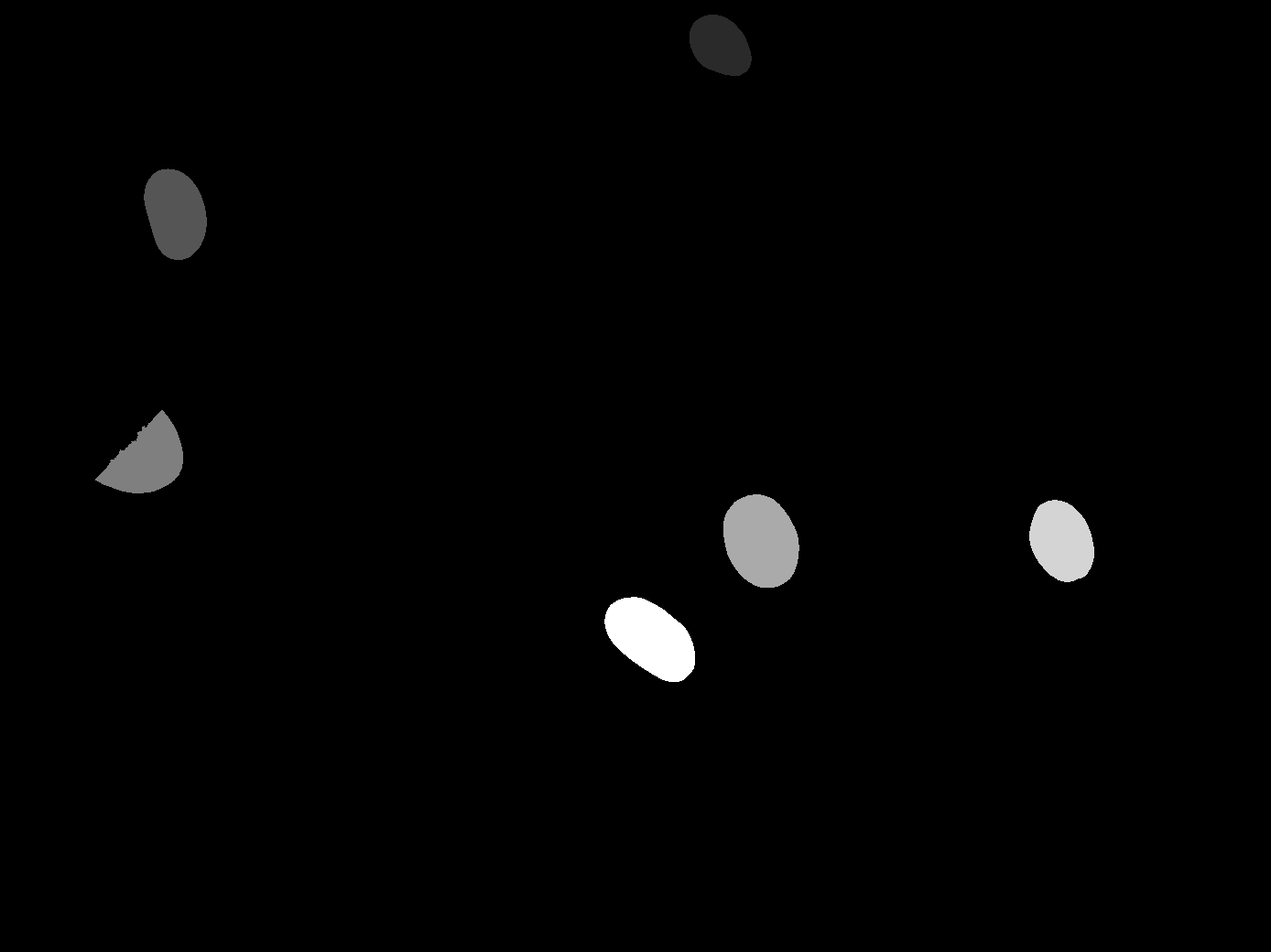

Supplement: S1 File — This file contains all scripts (CellProfiler v2.1.1 and MATLAB2016a) and data necessary to reproduce the information shown in Fig 3. (ZIP) [file pone.0180810.s001.zip › vitaminD_eColi_reproducibleResearchArchive/Results2016/A_32_c0_seg.tif]

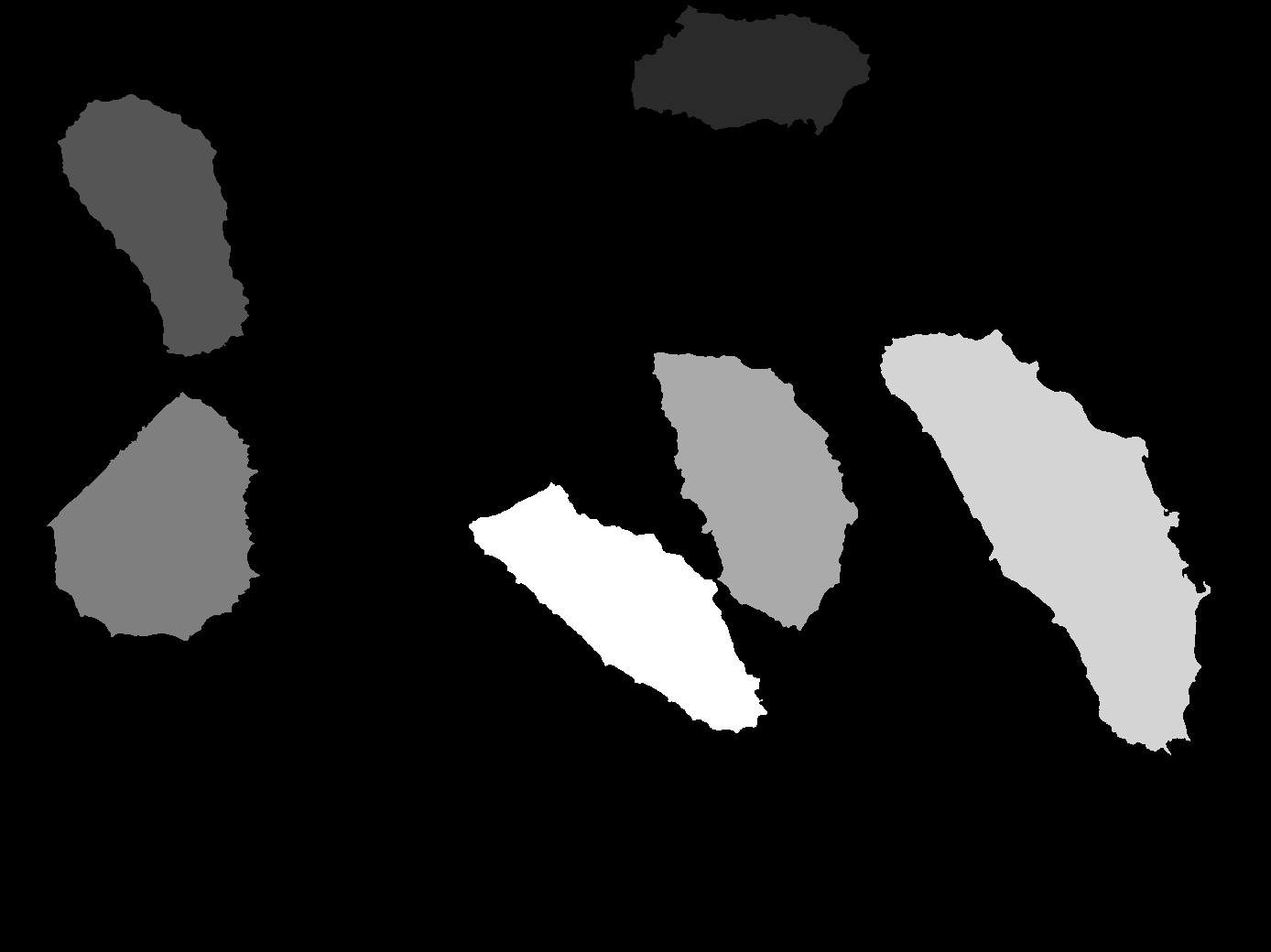

Supplement: S1 File — This file contains all scripts (CellProfiler v2.1.1 and MATLAB2016a) and data necessary to reproduce the information shown in Fig 3. (ZIP) [file pone.0180810.s001.zip › vitaminD_eColi_reproducibleResearchArchive/Results2016/A_32_c2_seg.tif]

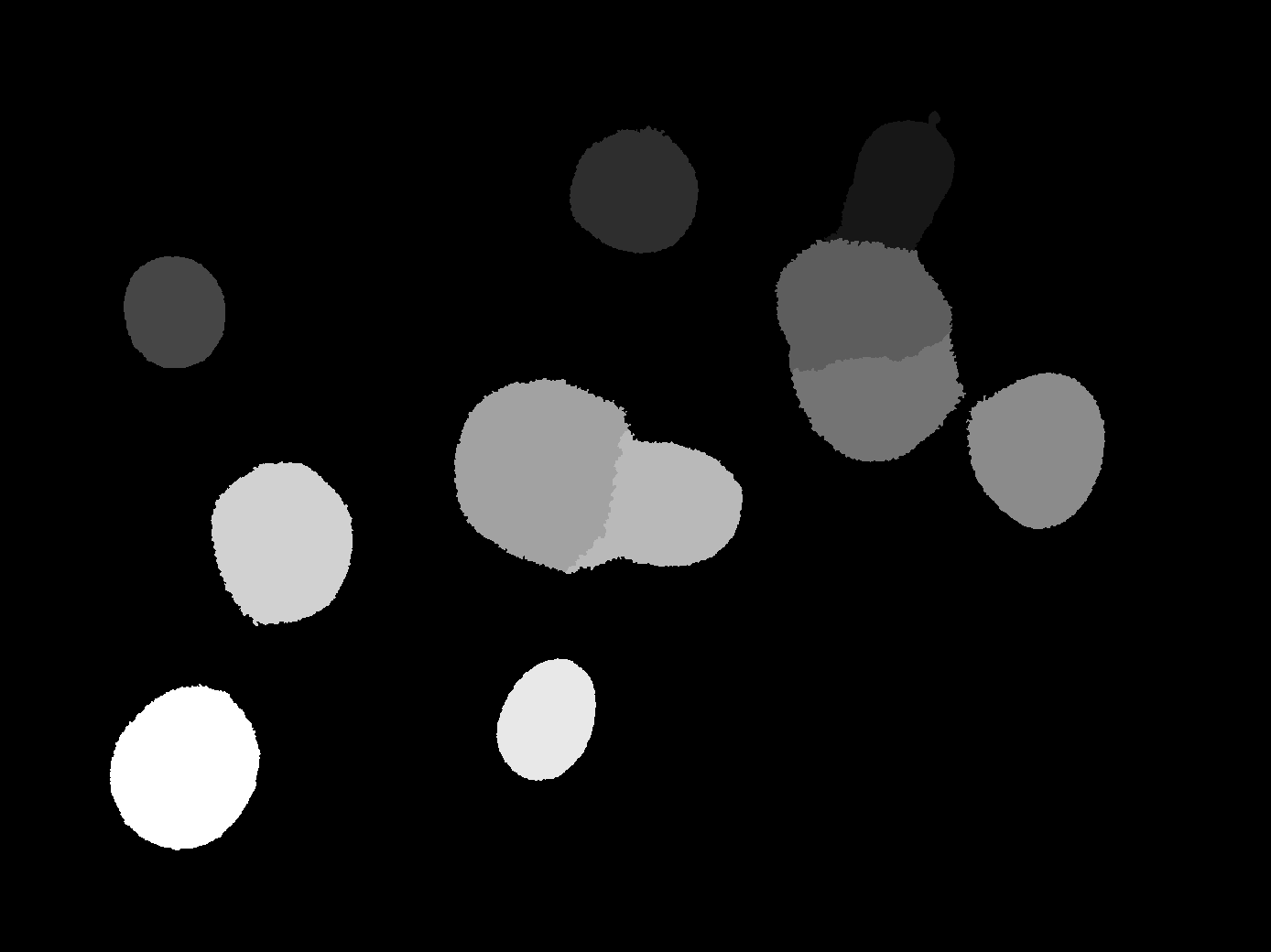

Supplement: S1 File — This file contains all scripts (CellProfiler v2.1.1 and MATLAB2016a) and data necessary to reproduce the information shown in Fig 3. (ZIP) [file pone.0180810.s001.zip › vitaminD_eColi_reproducibleResearchArchive/Results2016/A_33_c0_seg.tif]

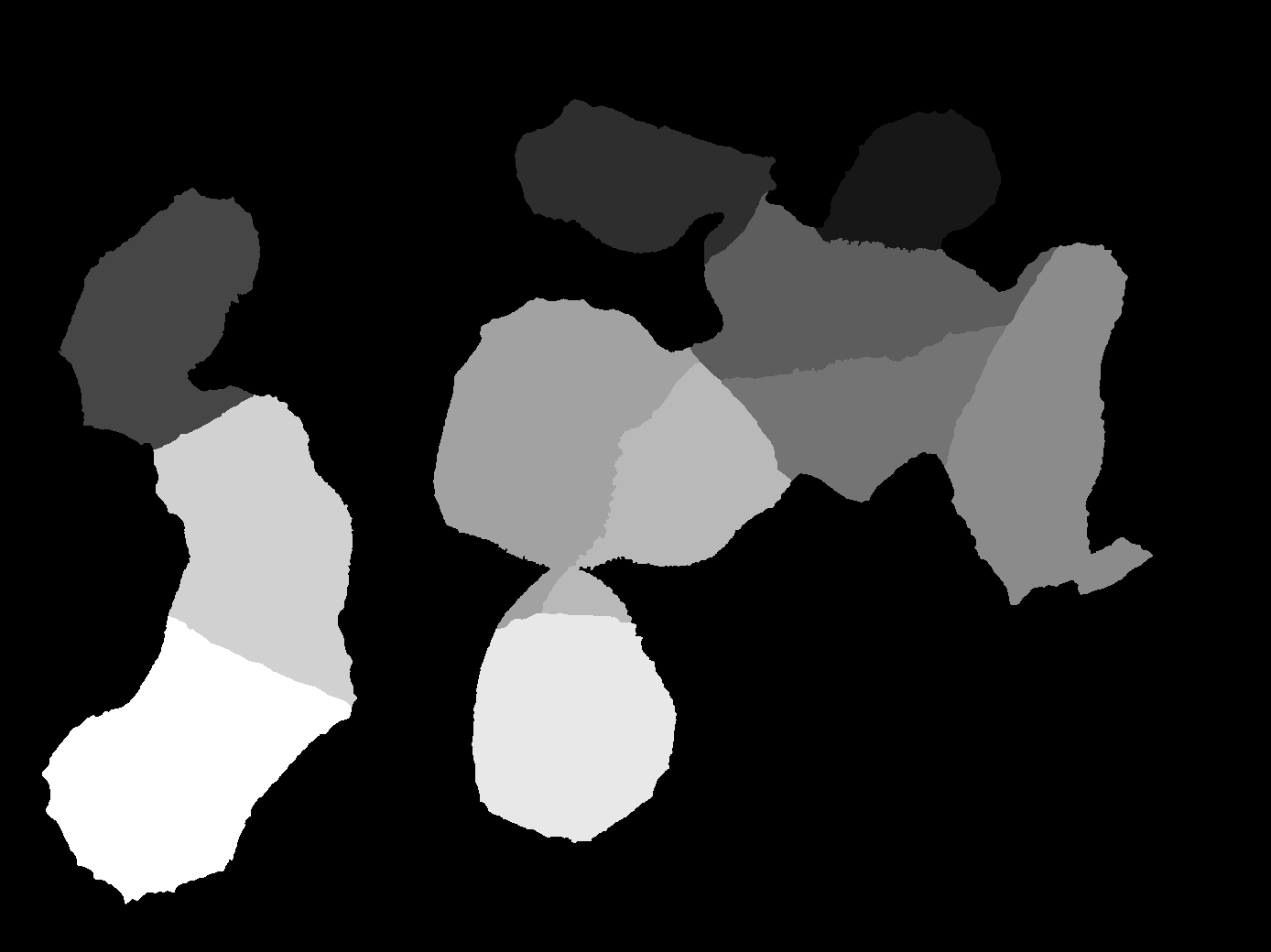

Supplement: S1 File — This file contains all scripts (CellProfiler v2.1.1 and MATLAB2016a) and data necessary to reproduce the information shown in Fig 3. (ZIP) [file pone.0180810.s001.zip › vitaminD_eColi_reproducibleResearchArchive/Results2016/A_33_c2_seg.tif]

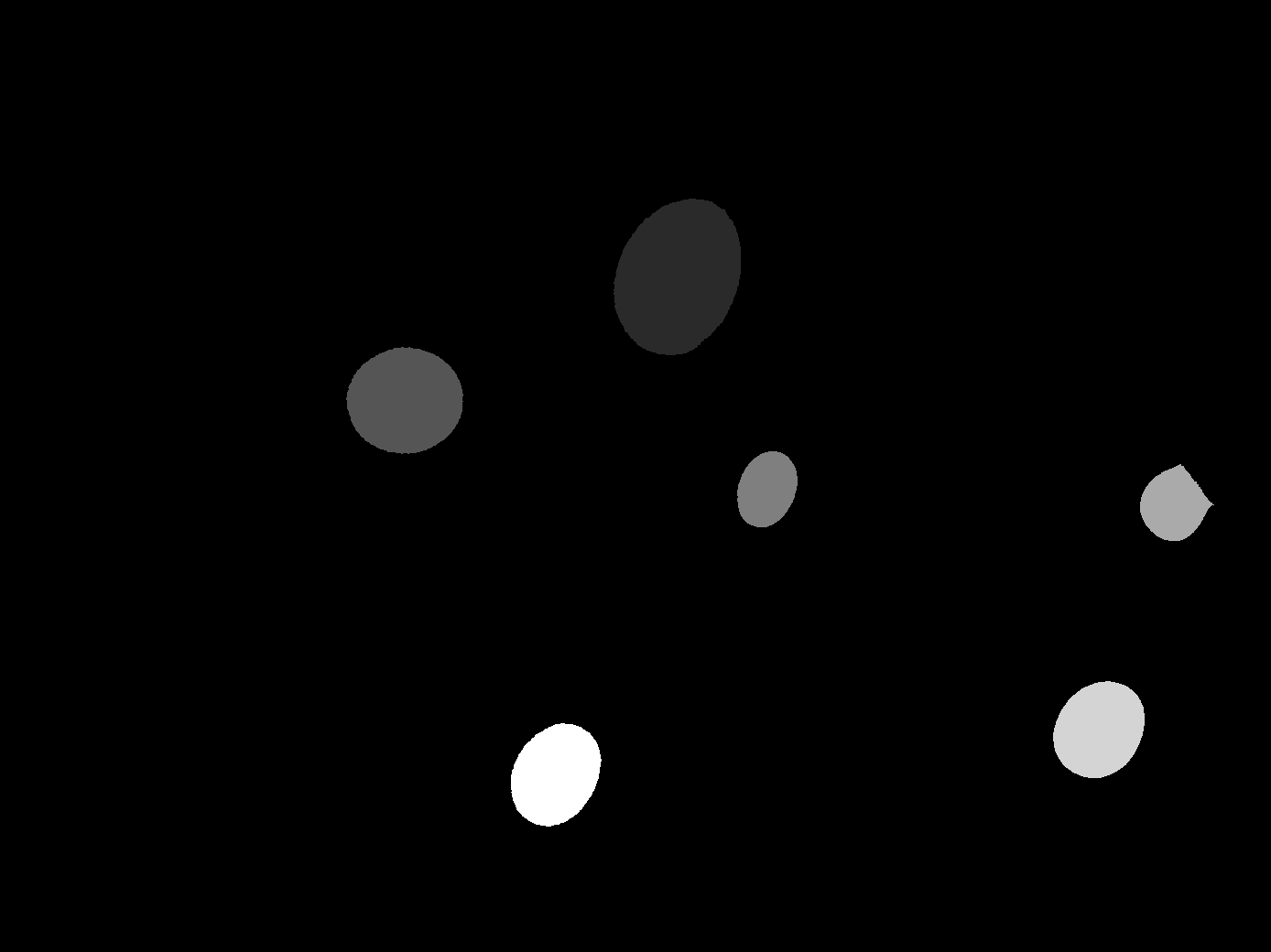

Supplement: S1 File — This file contains all scripts (CellProfiler v2.1.1 and MATLAB2016a) and data necessary to reproduce the information shown in Fig 3. (ZIP) [file pone.0180810.s001.zip › vitaminD_eColi_reproducibleResearchArchive/Results2016/A_34_c0_seg.tif]

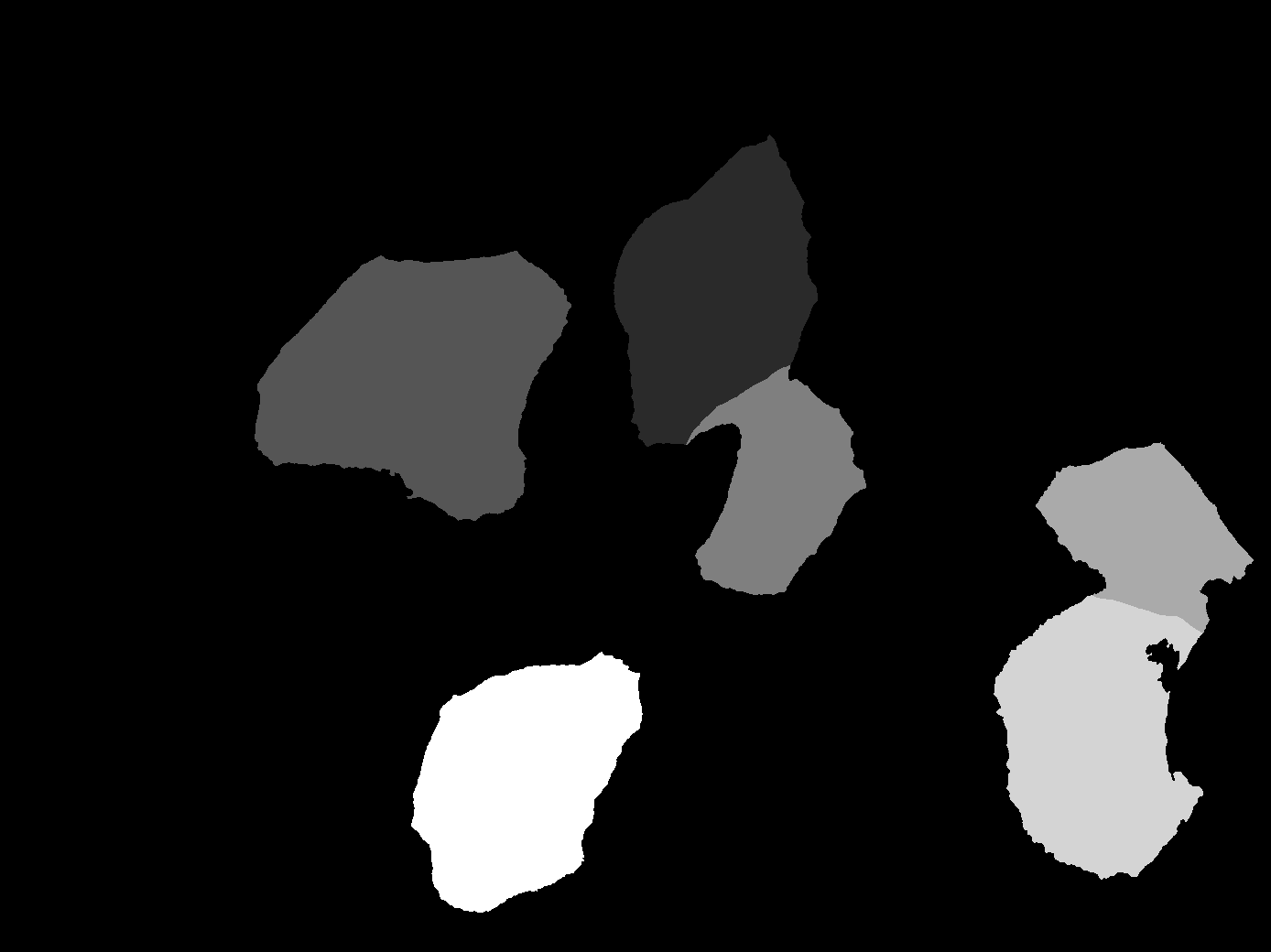

Supplement: S1 File — This file contains all scripts (CellProfiler v2.1.1 and MATLAB2016a) and data necessary to reproduce the information shown in Fig 3. (ZIP) [file pone.0180810.s001.zip › vitaminD_eColi_reproducibleResearchArchive/Results2016/A_34_c2_seg.tif]

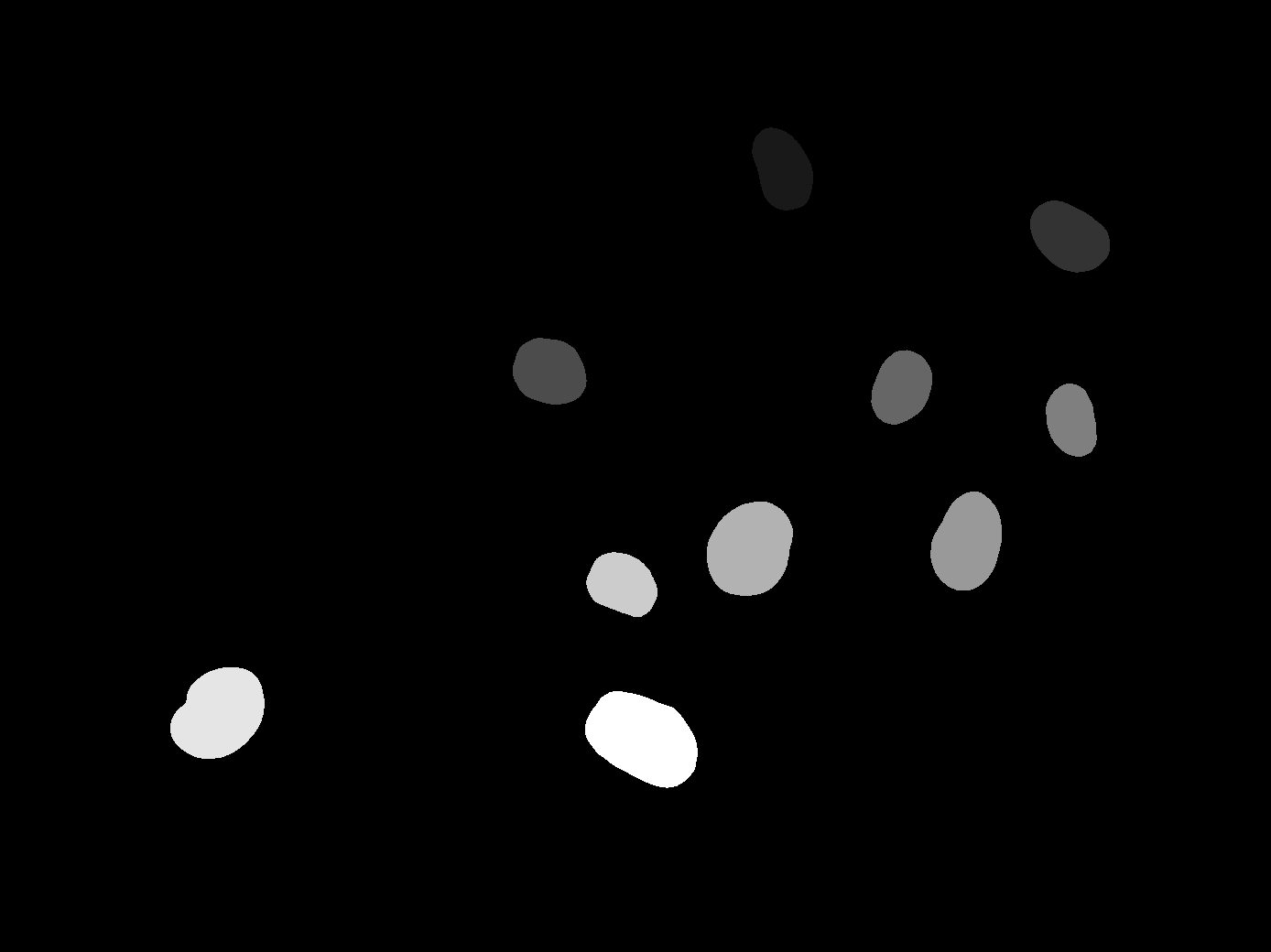

Supplement: S1 File — This file contains all scripts (CellProfiler v2.1.1 and MATLAB2016a) and data necessary to reproduce the information shown in Fig 3. (ZIP) [file pone.0180810.s001.zip › vitaminD_eColi_reproducibleResearchArchive/Results2016/A_35_c0_seg.tif]

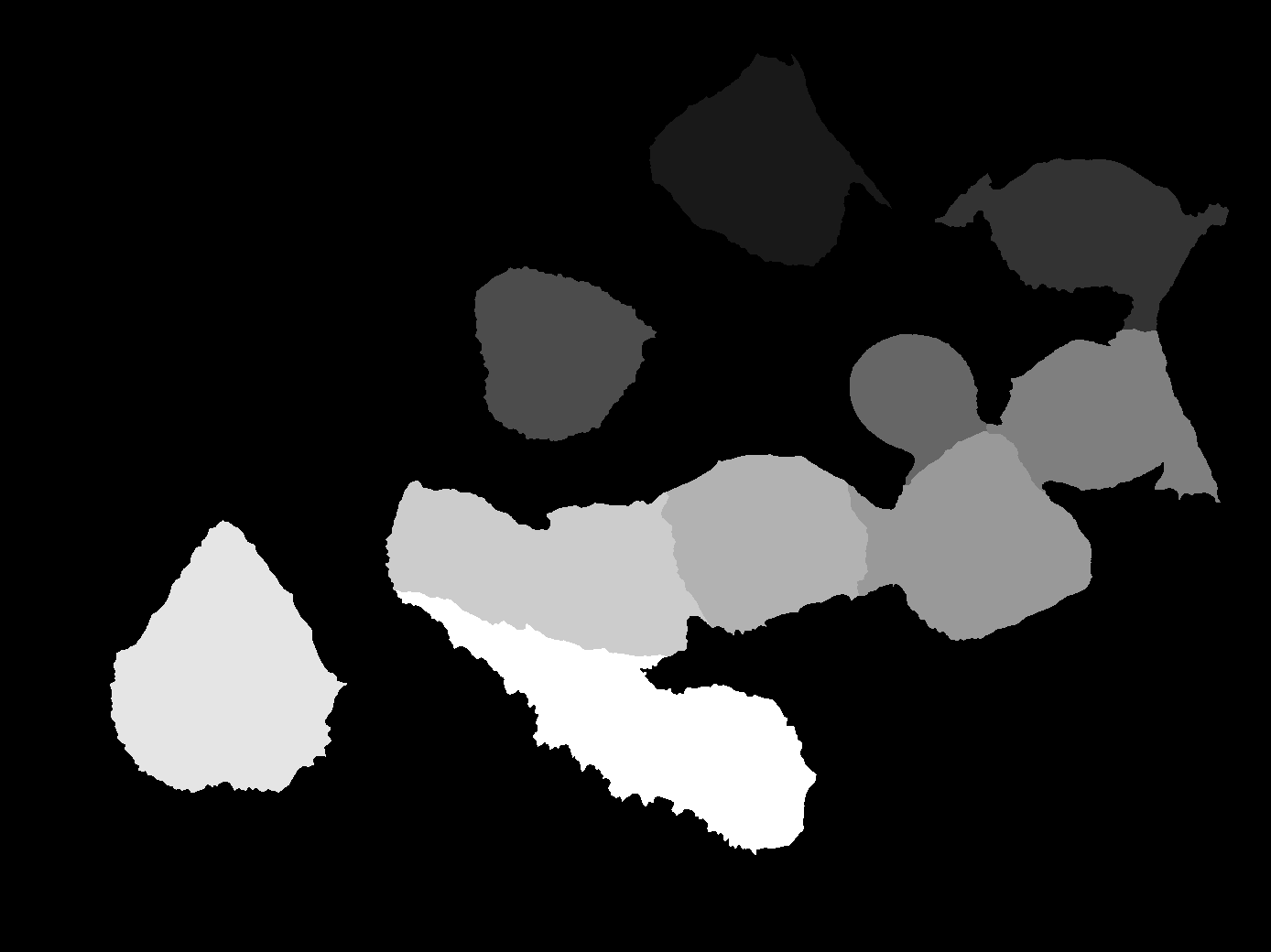

Supplement: S1 File — This file contains all scripts (CellProfiler v2.1.1 and MATLAB2016a) and data necessary to reproduce the information shown in Fig 3. (ZIP) [file pone.0180810.s001.zip › vitaminD_eColi_reproducibleResearchArchive/Results2016/A_35_c2_seg.tif]

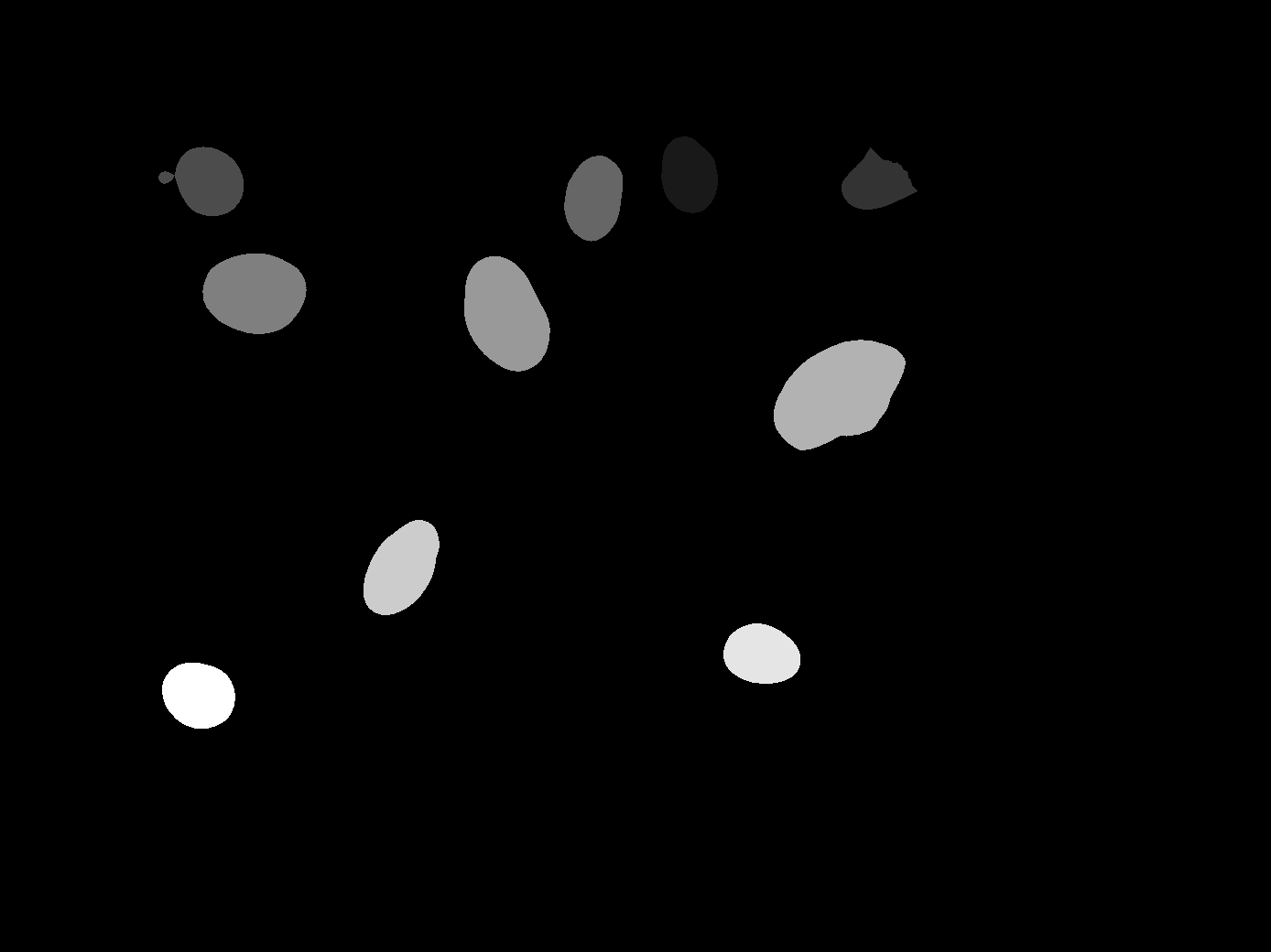

Supplement: S1 File — This file contains all scripts (CellProfiler v2.1.1 and MATLAB2016a) and data necessary to reproduce the information shown in Fig 3. (ZIP) [file pone.0180810.s001.zip › vitaminD_eColi_reproducibleResearchArchive/Results2016/A_36_c0_seg.tif]

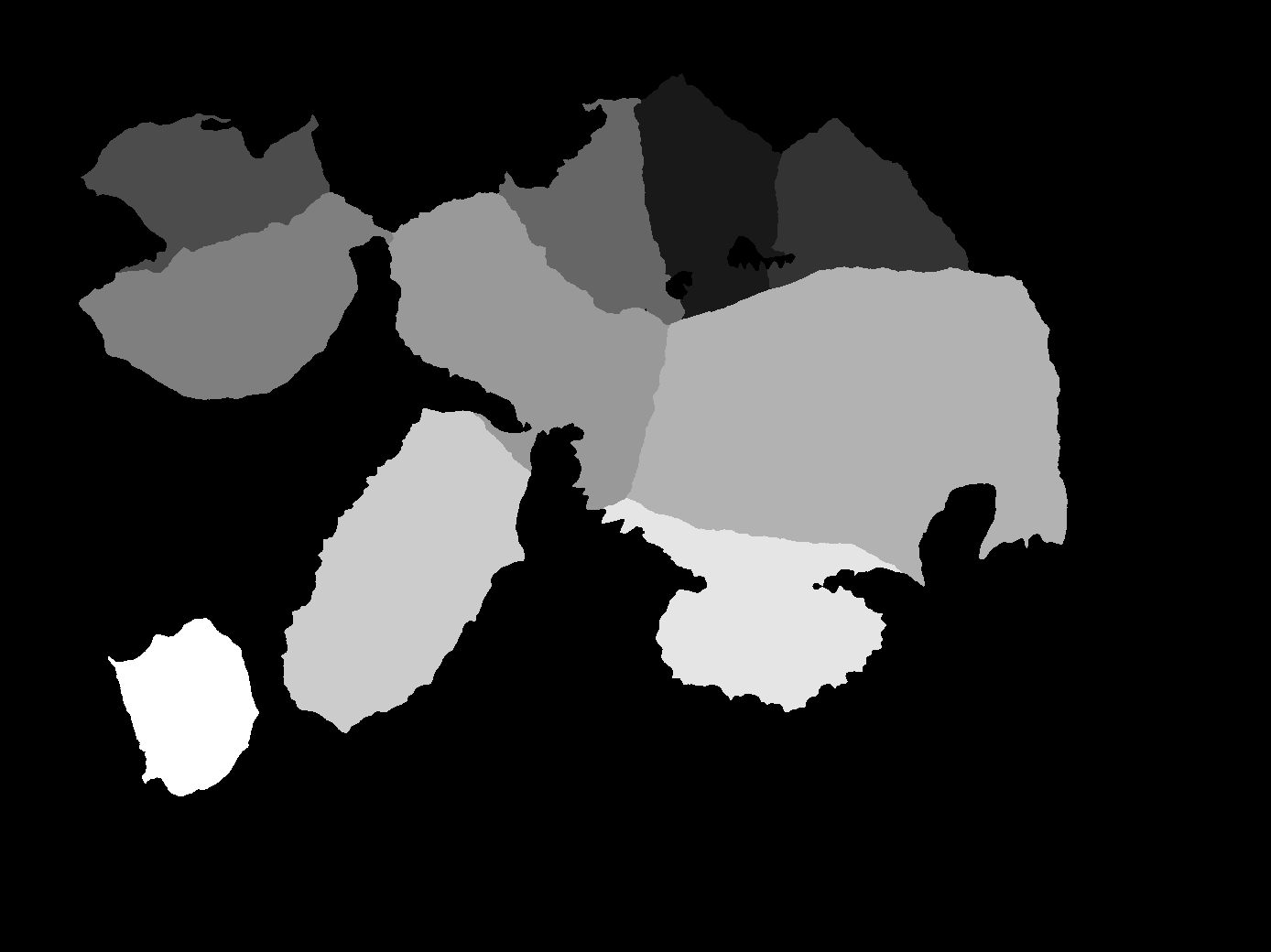

Supplement: S1 File — This file contains all scripts (CellProfiler v2.1.1 and MATLAB2016a) and data necessary to reproduce the information shown in Fig 3. (ZIP) [file pone.0180810.s001.zip › vitaminD_eColi_reproducibleResearchArchive/Results2016/A_36_c2_seg.tif]

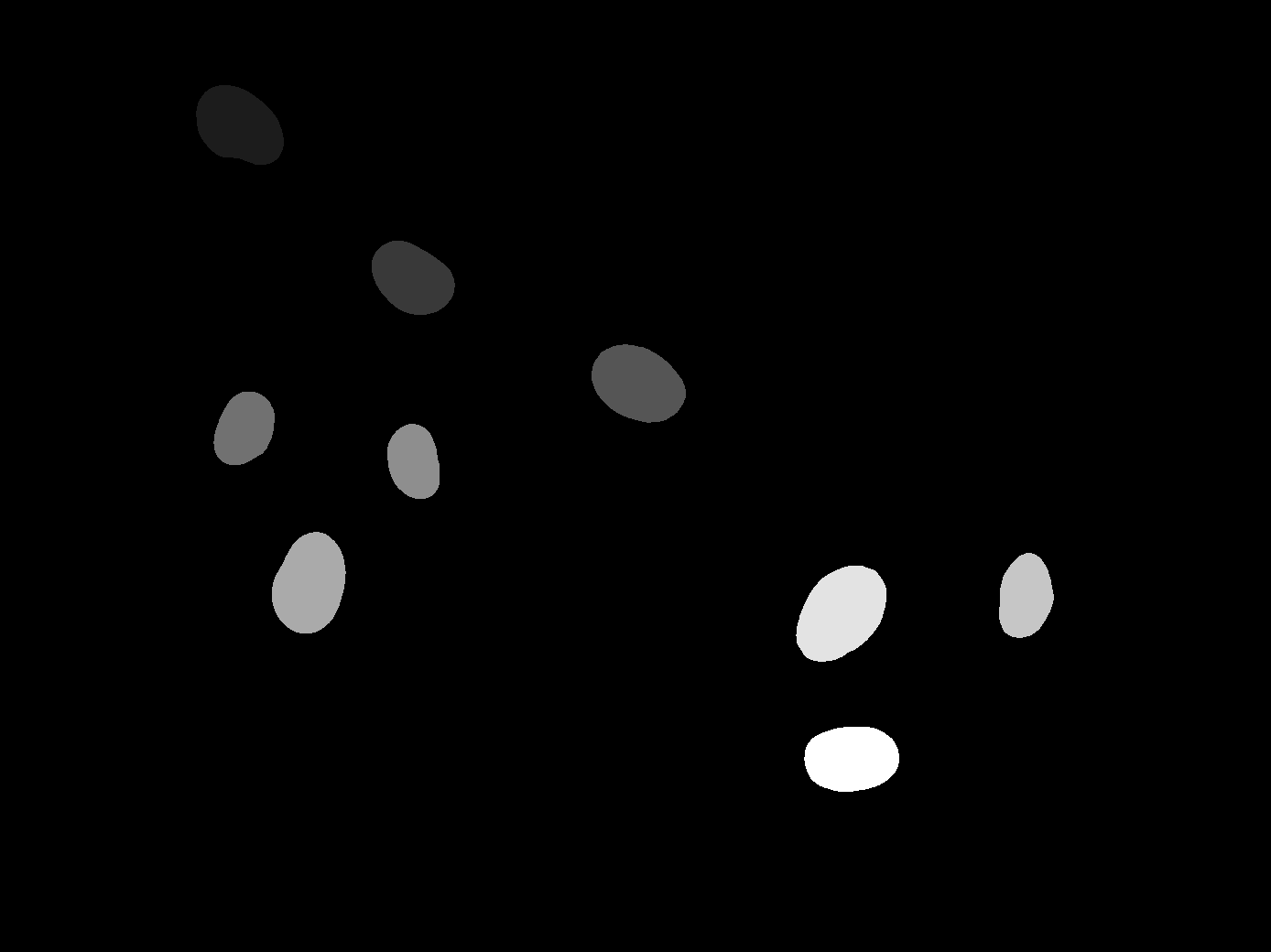

Supplement: S1 File — This file contains all scripts (CellProfiler v2.1.1 and MATLAB2016a) and data necessary to reproduce the information shown in Fig 3. (ZIP) [file pone.0180810.s001.zip › vitaminD_eColi_reproducibleResearchArchive/Results2016/A_37_c0_seg.tif]

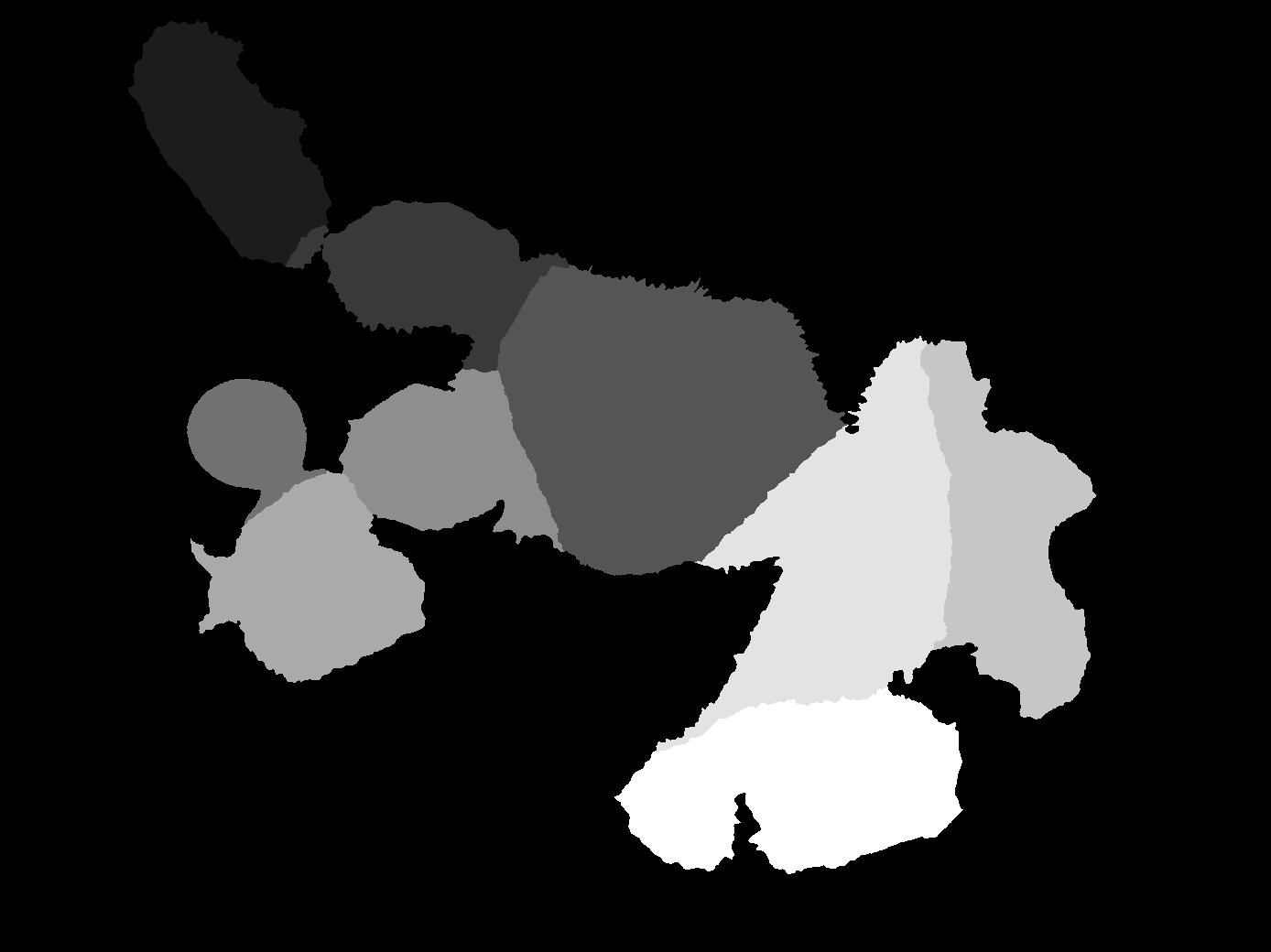

Supplement: S1 File — This file contains all scripts (CellProfiler v2.1.1 and MATLAB2016a) and data necessary to reproduce the information shown in Fig 3. (ZIP) [file pone.0180810.s001.zip › vitaminD_eColi_reproducibleResearchArchive/Results2016/A_37_c2_seg.tif]

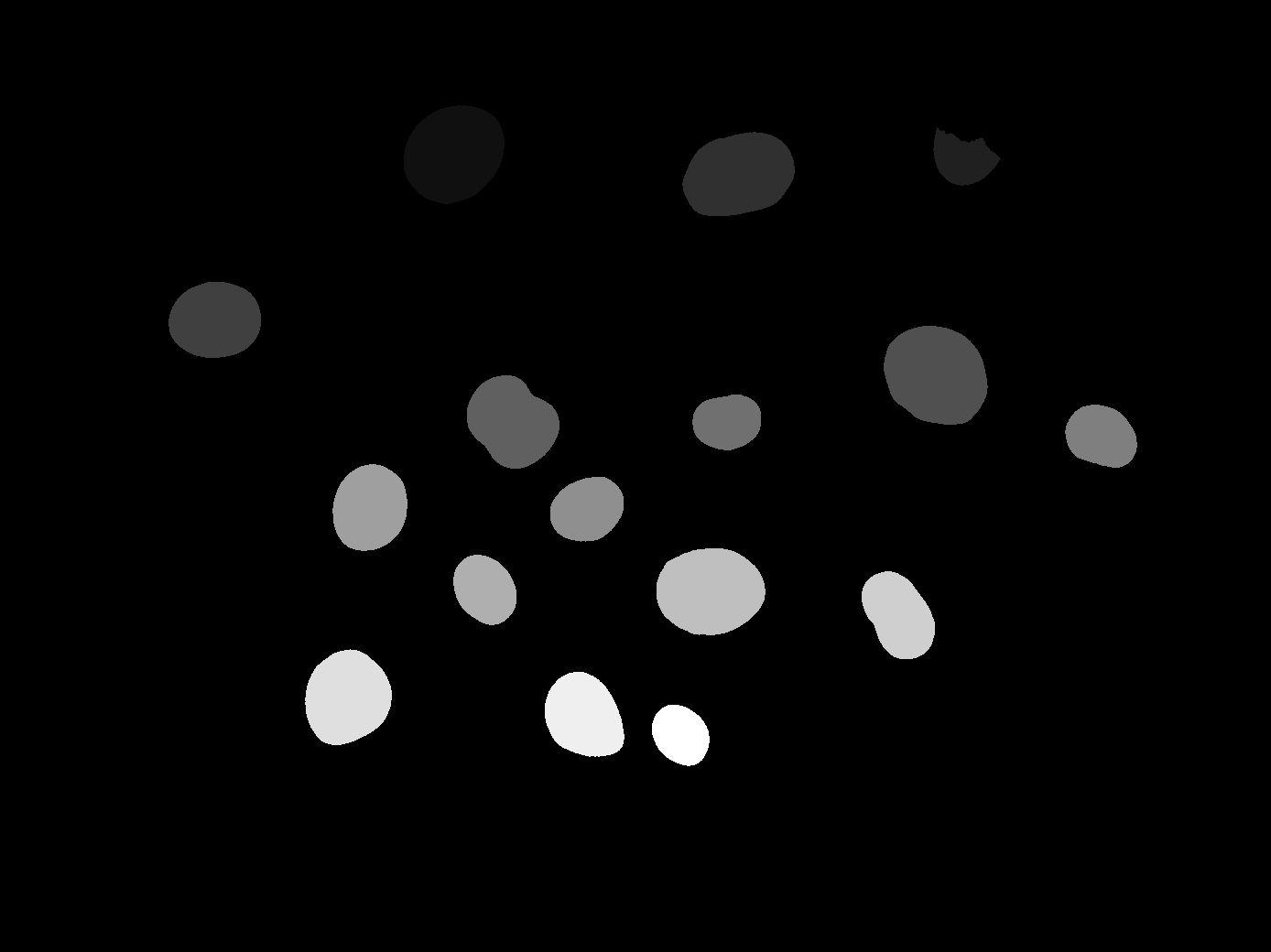

Supplement: S1 File — This file contains all scripts (CellProfiler v2.1.1 and MATLAB2016a) and data necessary to reproduce the information shown in Fig 3. (ZIP) [file pone.0180810.s001.zip › vitaminD_eColi_reproducibleResearchArchive/Results2016/A_38_c0_seg.tif]

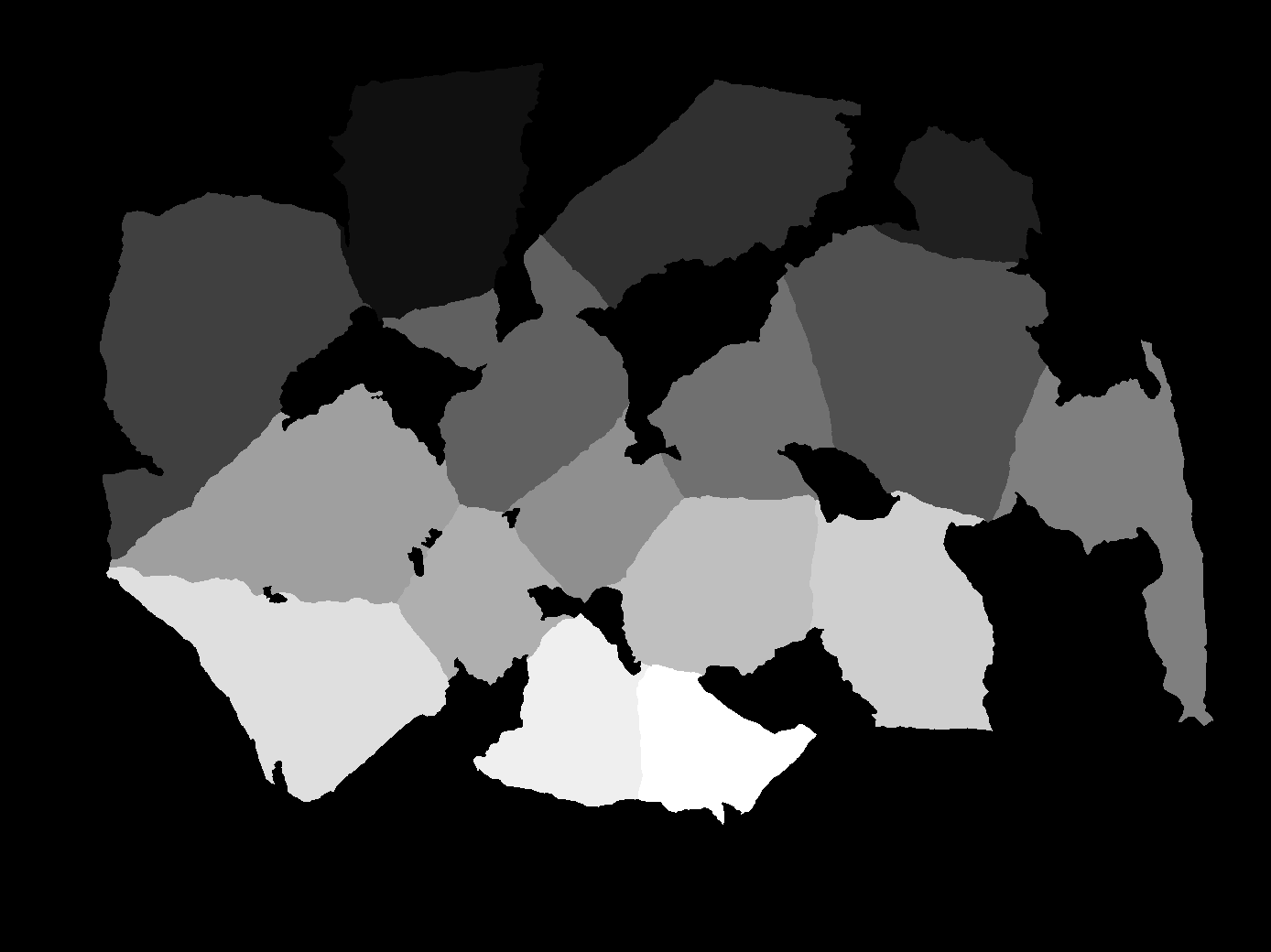

Supplement: S1 File — This file contains all scripts (CellProfiler v2.1.1 and MATLAB2016a) and data necessary to reproduce the information shown in Fig 3. (ZIP) [file pone.0180810.s001.zip › vitaminD_eColi_reproducibleResearchArchive/Results2016/A_38_c2_seg.tif]

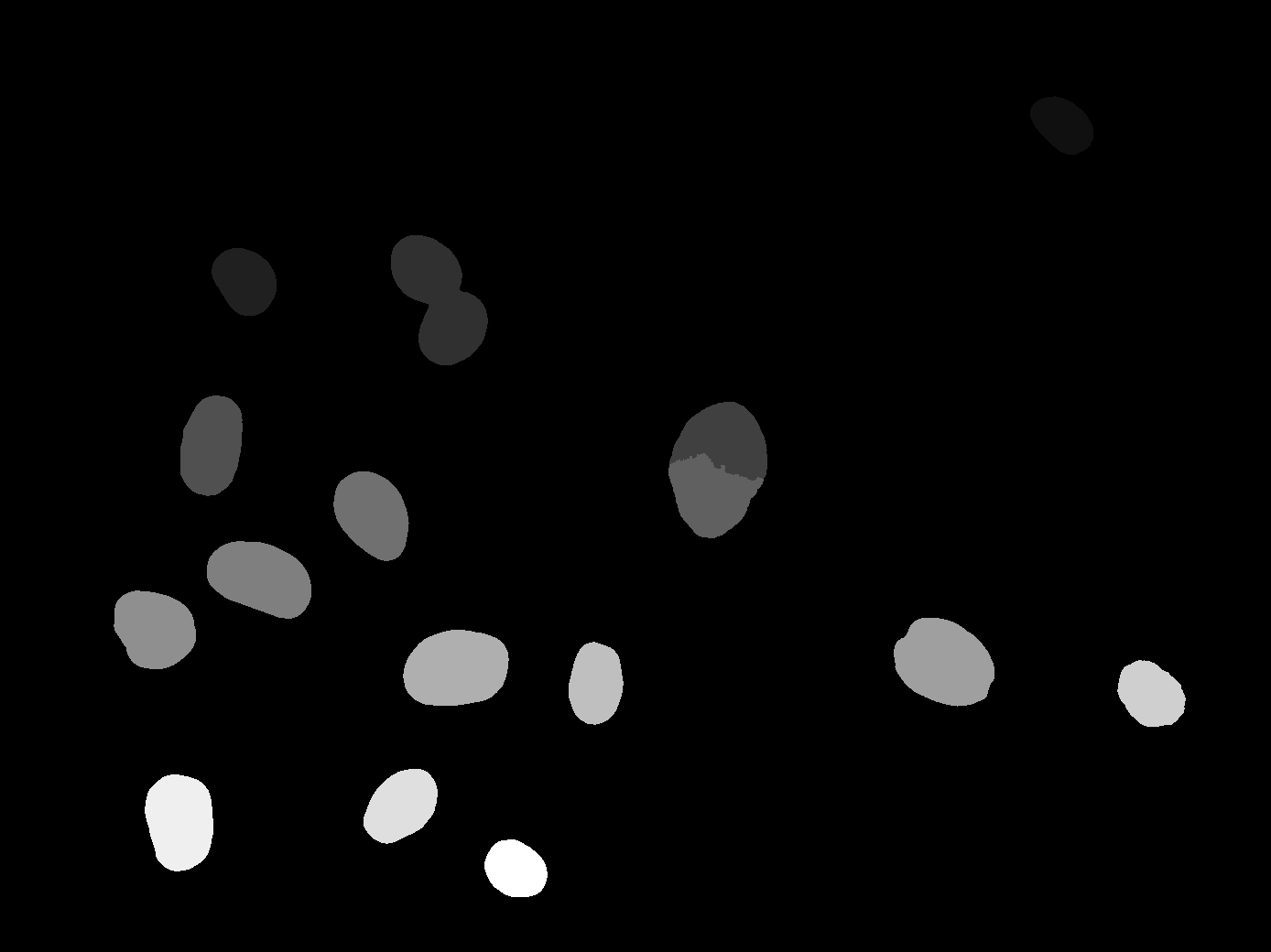

Supplement: S1 File — This file contains all scripts (CellProfiler v2.1.1 and MATLAB2016a) and data necessary to reproduce the information shown in Fig 3. (ZIP) [file pone.0180810.s001.zip › vitaminD_eColi_reproducibleResearchArchive/Results2016/A_39_c0_seg.tif]

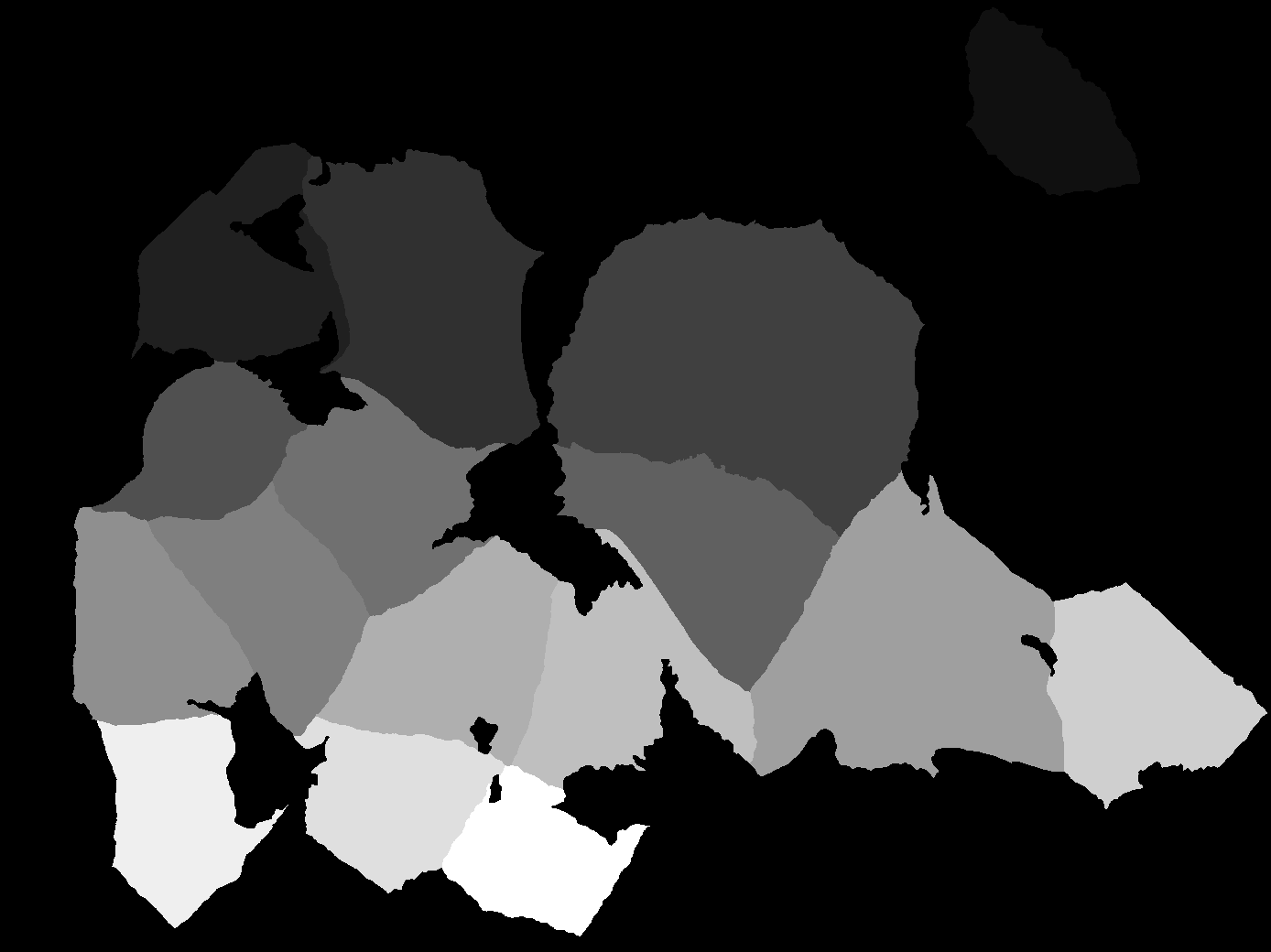

Supplement: S1 File — This file contains all scripts (CellProfiler v2.1.1 and MATLAB2016a) and data necessary to reproduce the information shown in Fig 3. (ZIP) [file pone.0180810.s001.zip › vitaminD_eColi_reproducibleResearchArchive/Results2016/A_39_c2_seg.tif]

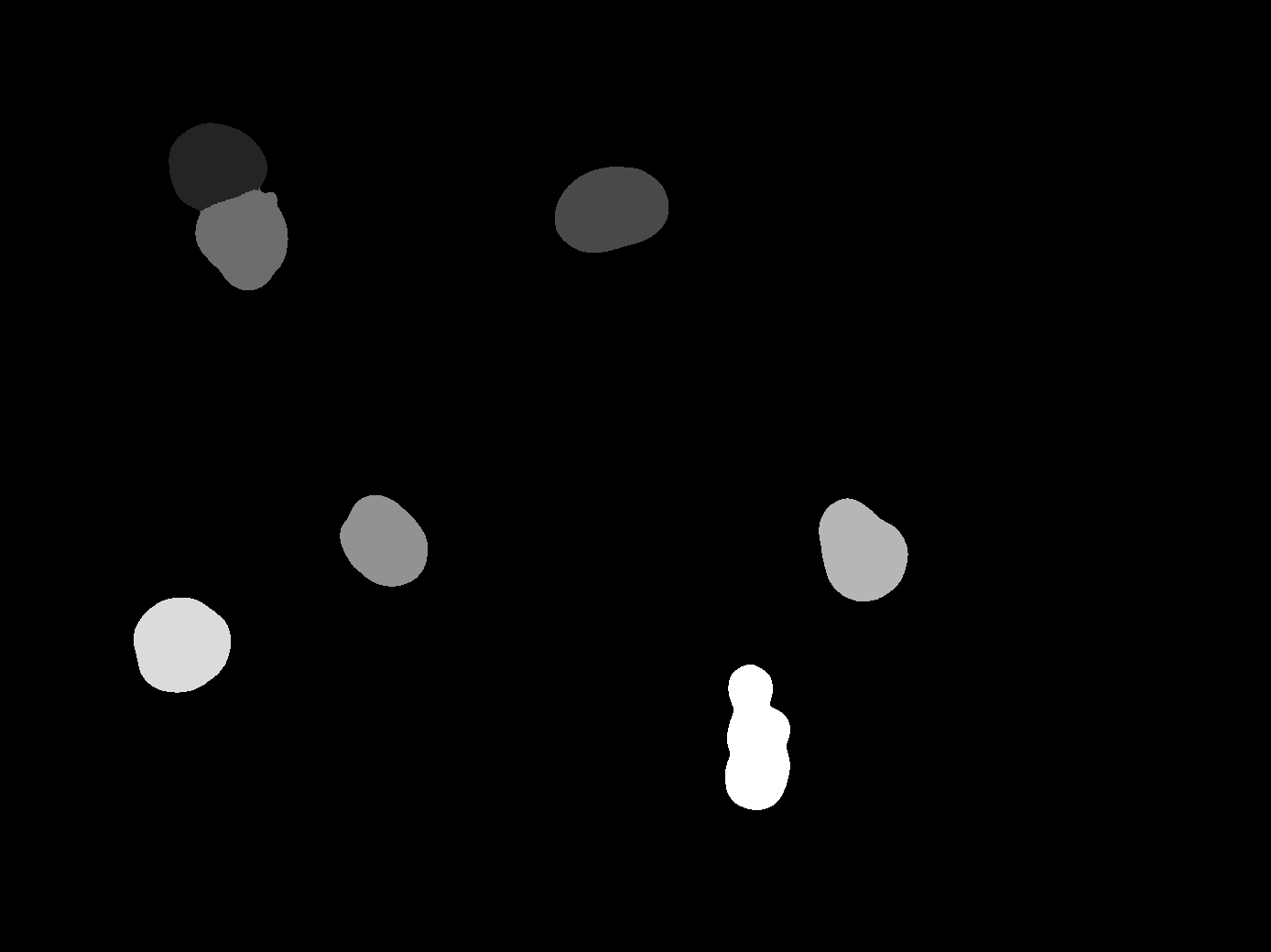

Supplement: S1 File — This file contains all scripts (CellProfiler v2.1.1 and MATLAB2016a) and data necessary to reproduce the information shown in Fig 3. (ZIP) [file pone.0180810.s001.zip › vitaminD_eColi_reproducibleResearchArchive/Results2016/A_3_c0_seg.tif]

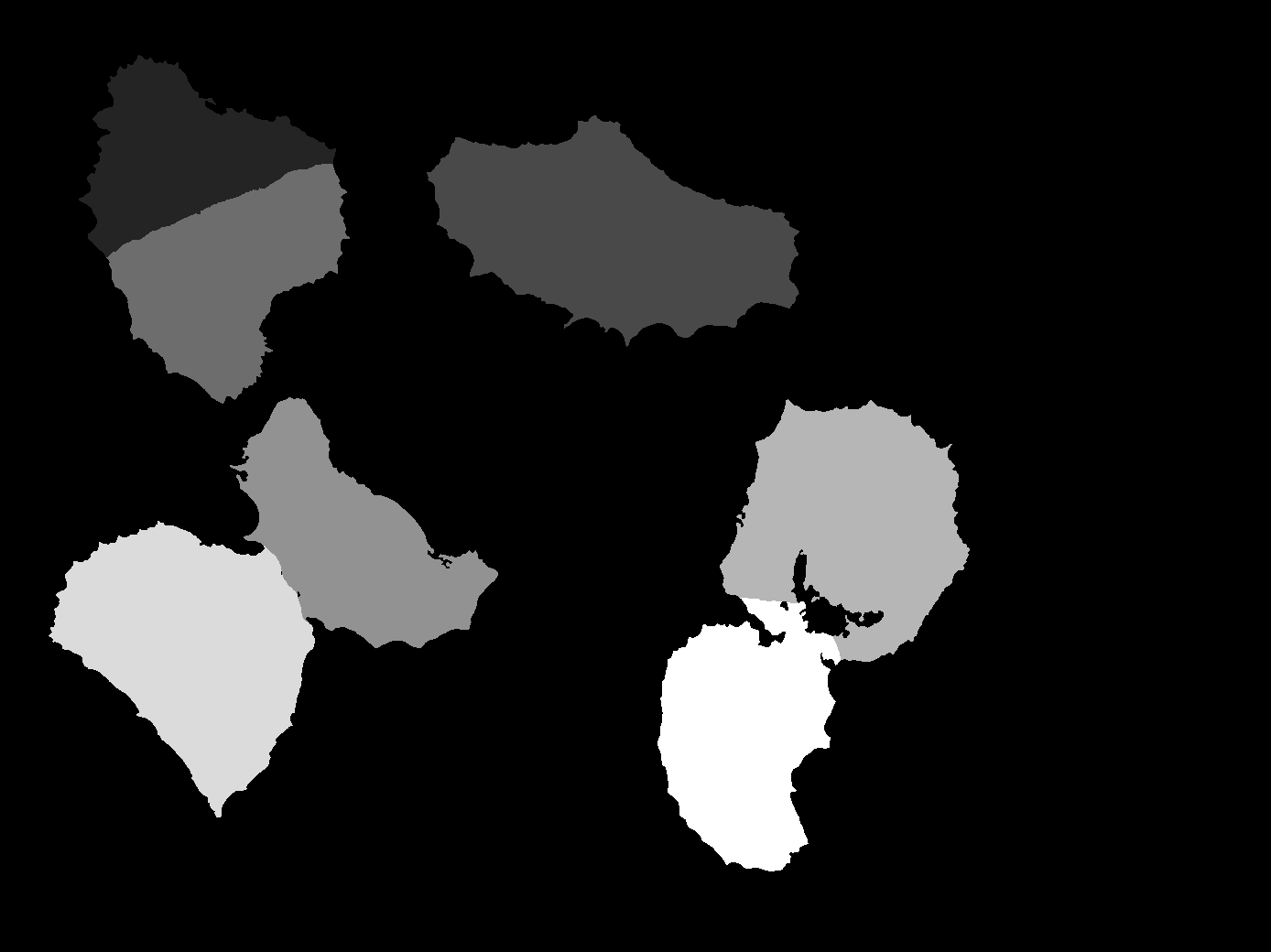

Supplement: S1 File — This file contains all scripts (CellProfiler v2.1.1 and MATLAB2016a) and data necessary to reproduce the information shown in Fig 3. (ZIP) [file pone.0180810.s001.zip › vitaminD_eColi_reproducibleResearchArchive/Results2016/A_3_c2_seg.tif]

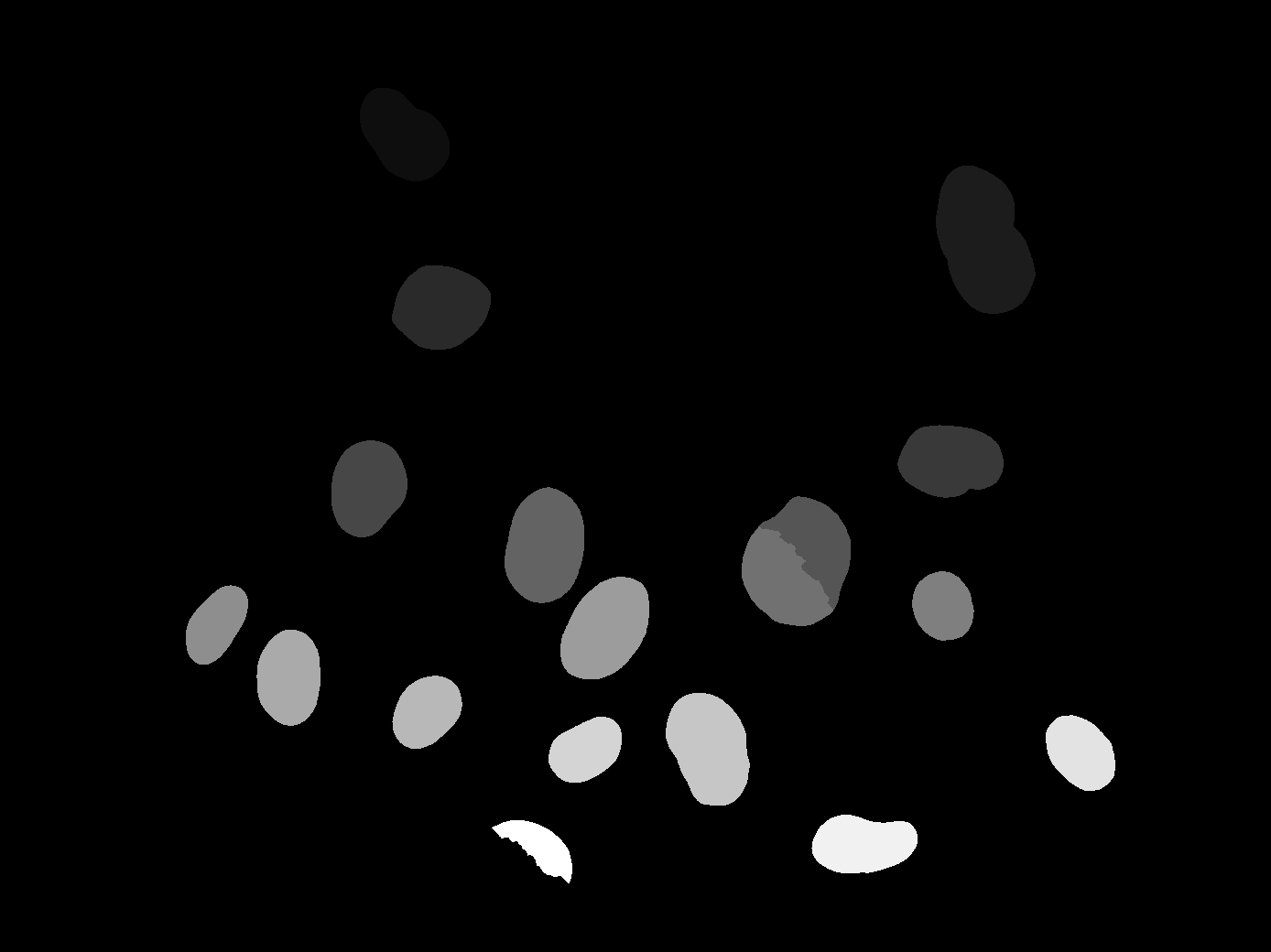

Supplement: S1 File — This file contains all scripts (CellProfiler v2.1.1 and MATLAB2016a) and data necessary to reproduce the information shown in Fig 3. (ZIP) [file pone.0180810.s001.zip › vitaminD_eColi_reproducibleResearchArchive/Results2016/A_40_c0_seg.tif]

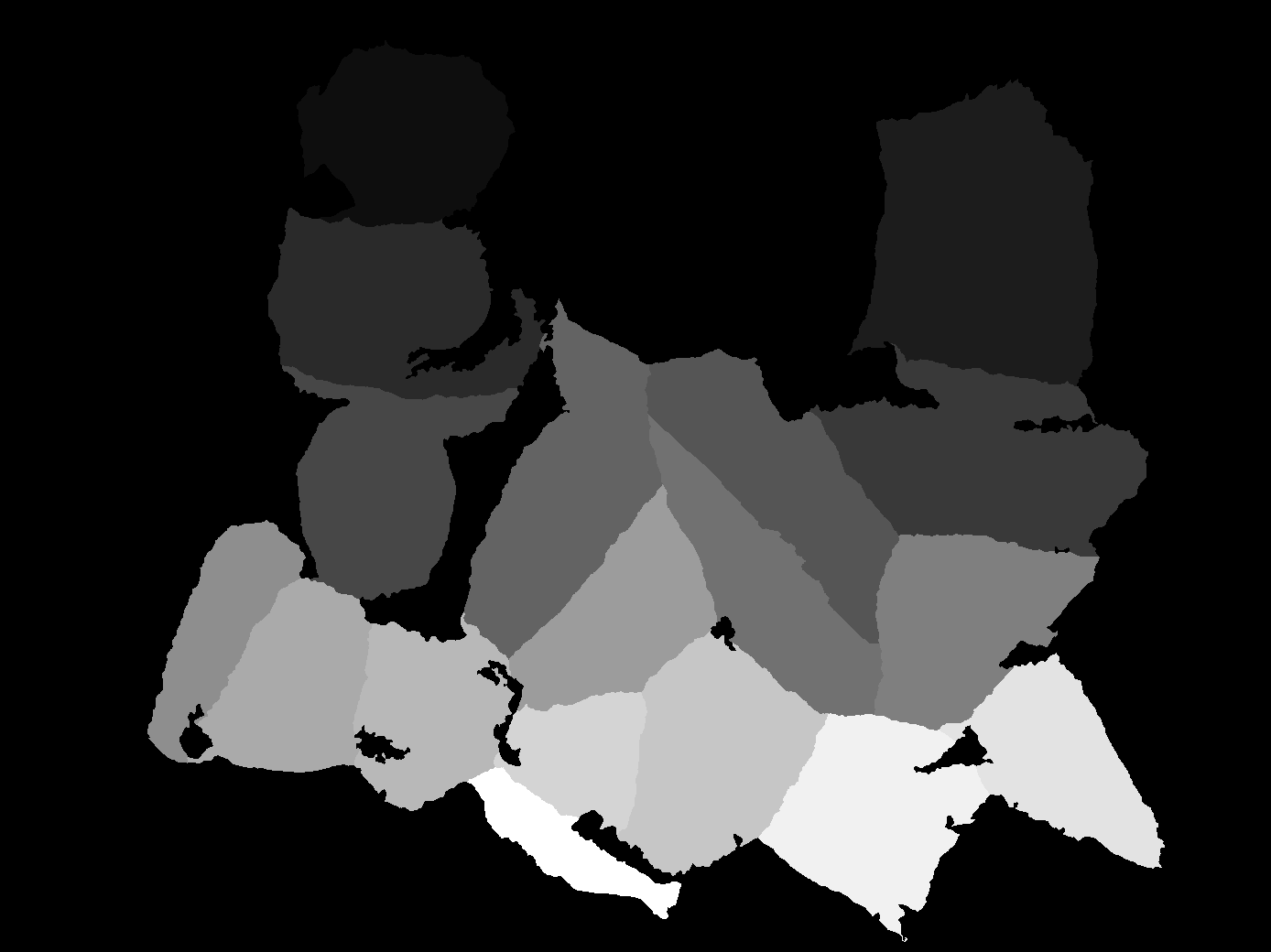

Supplement: S1 File — This file contains all scripts (CellProfiler v2.1.1 and MATLAB2016a) and data necessary to reproduce the information shown in Fig 3. (ZIP) [file pone.0180810.s001.zip › vitaminD_eColi_reproducibleResearchArchive/Results2016/A_40_c2_seg.tif]

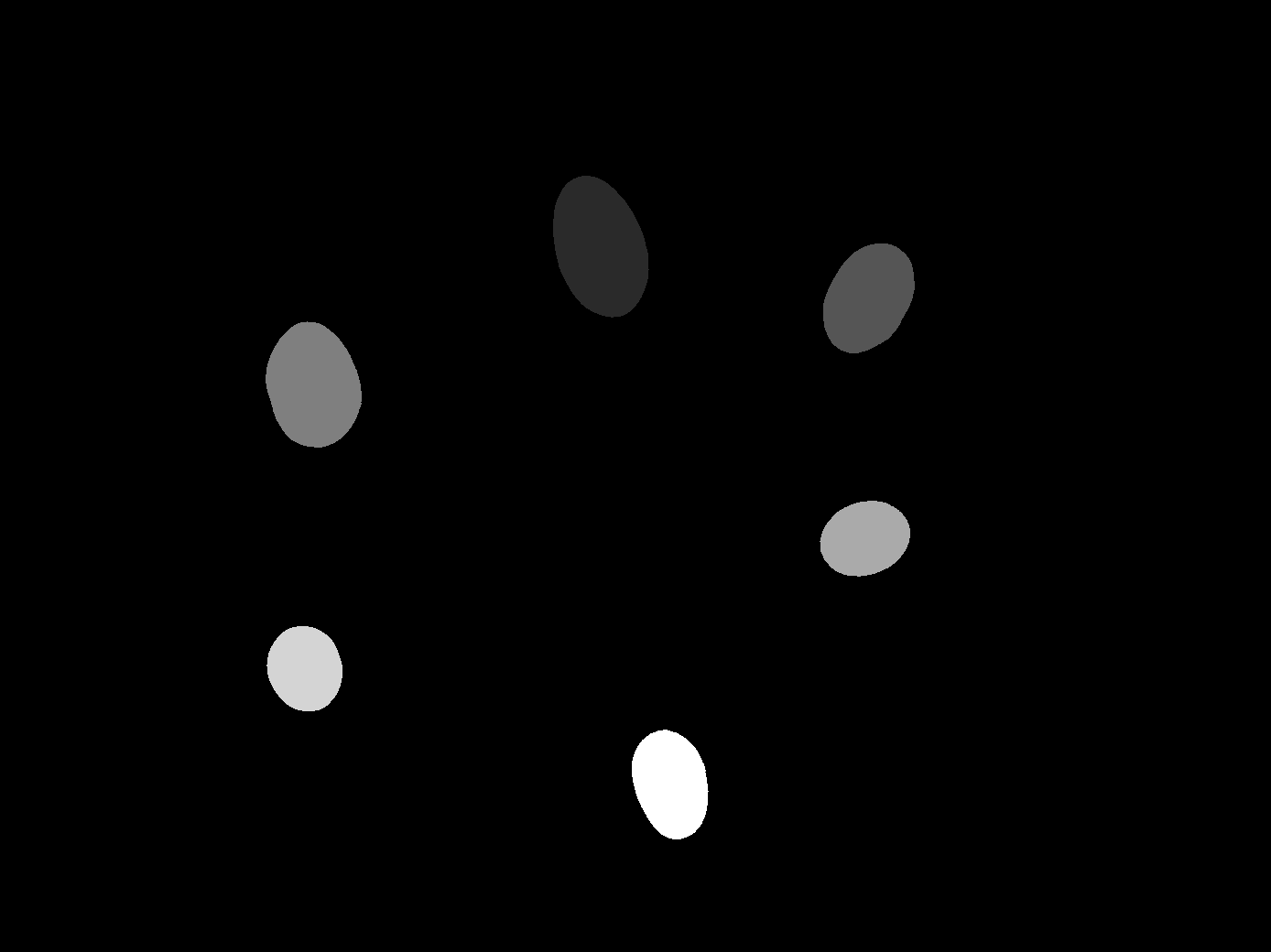

Supplement: S1 File — This file contains all scripts (CellProfiler v2.1.1 and MATLAB2016a) and data necessary to reproduce the information shown in Fig 3. (ZIP) [file pone.0180810.s001.zip › vitaminD_eColi_reproducibleResearchArchive/Results2016/A_4_c0_seg.tif]

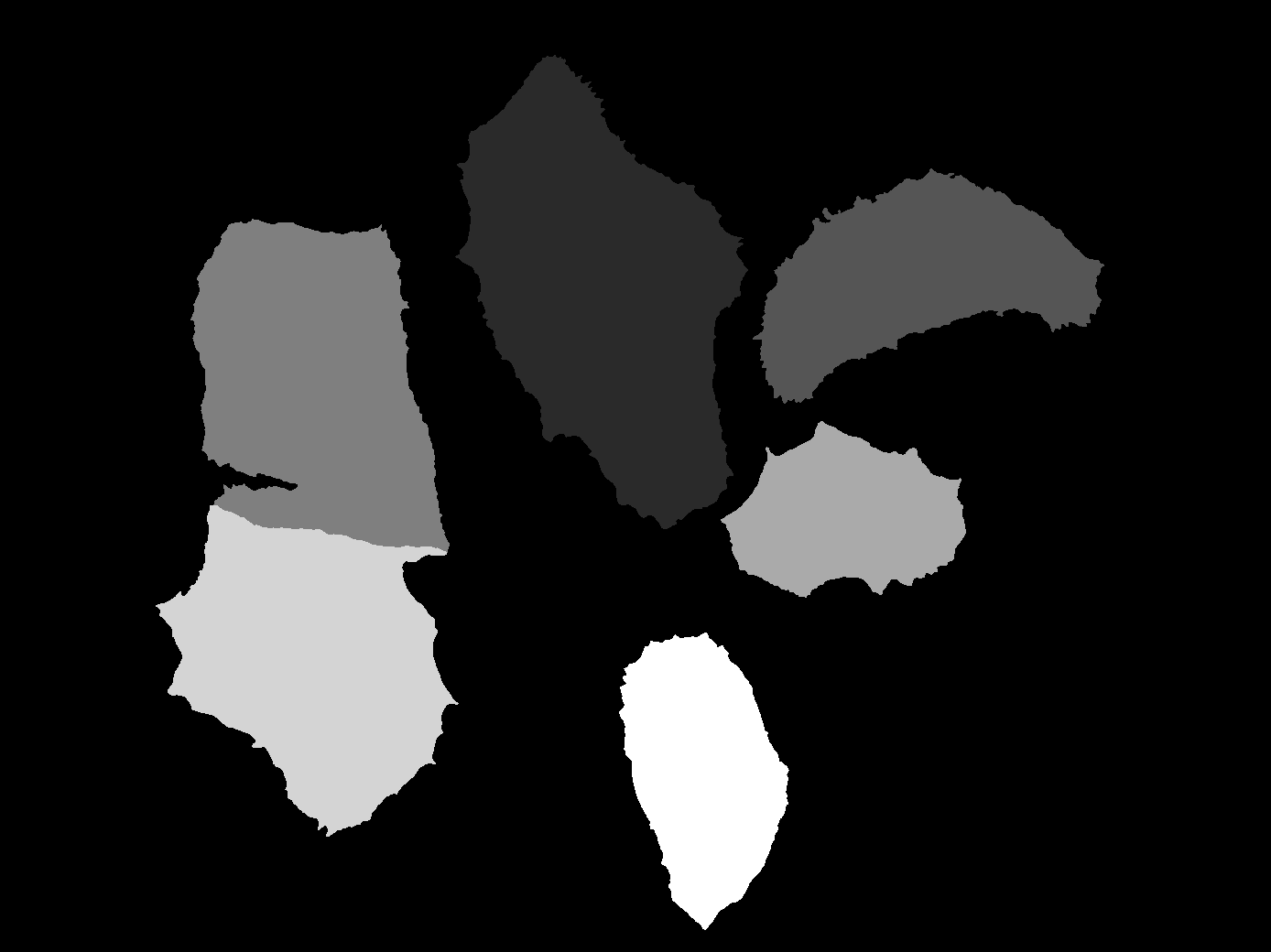

Supplement: S1 File — This file contains all scripts (CellProfiler v2.1.1 and MATLAB2016a) and data necessary to reproduce the information shown in Fig 3. (ZIP) [file pone.0180810.s001.zip › vitaminD_eColi_reproducibleResearchArchive/Results2016/A_4_c2_seg.tif]

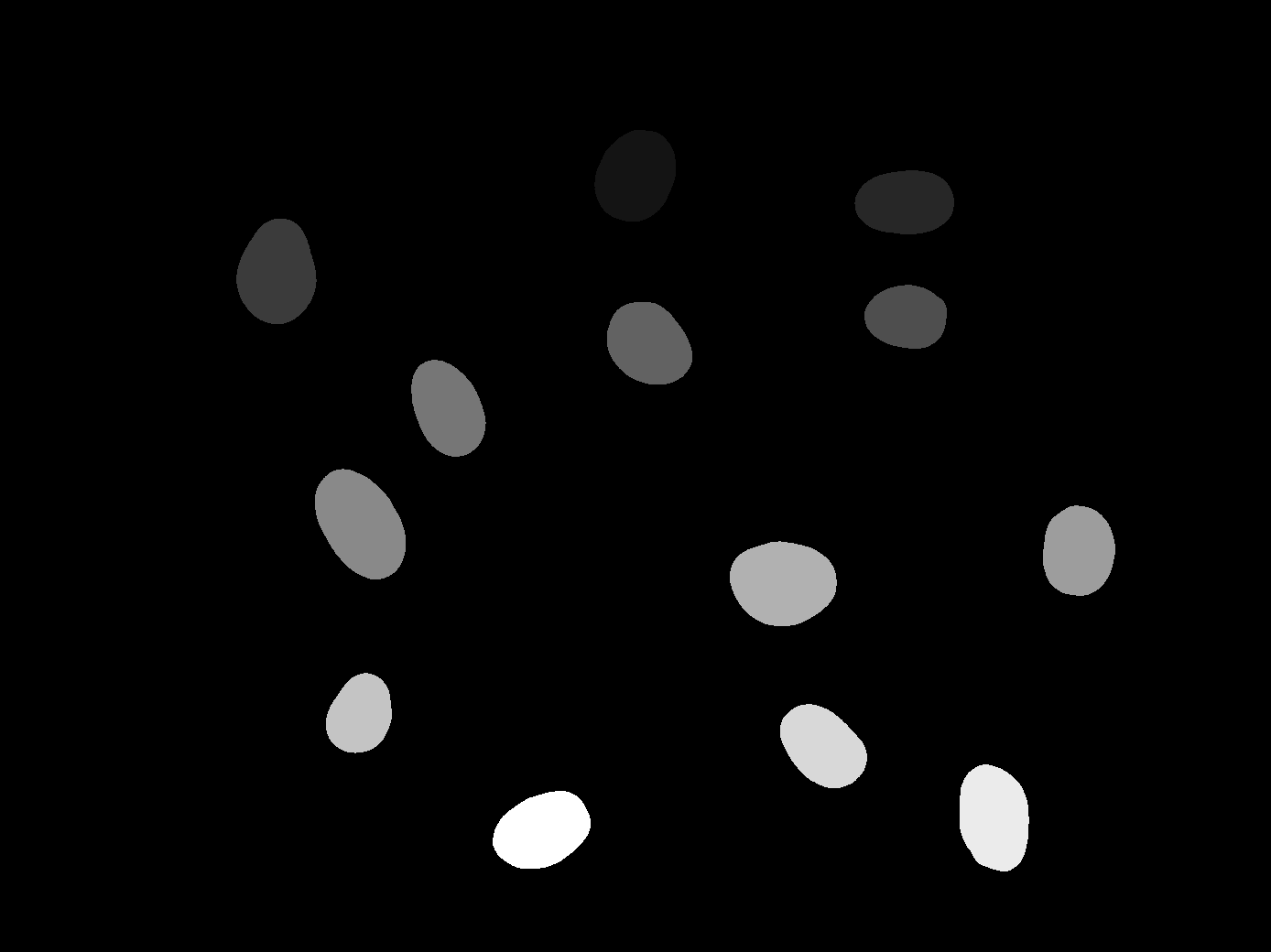

Supplement: S1 File — This file contains all scripts (CellProfiler v2.1.1 and MATLAB2016a) and data necessary to reproduce the information shown in Fig 3. (ZIP) [file pone.0180810.s001.zip › vitaminD_eColi_reproducibleResearchArchive/Results2016/A_5_c0_seg.tif]

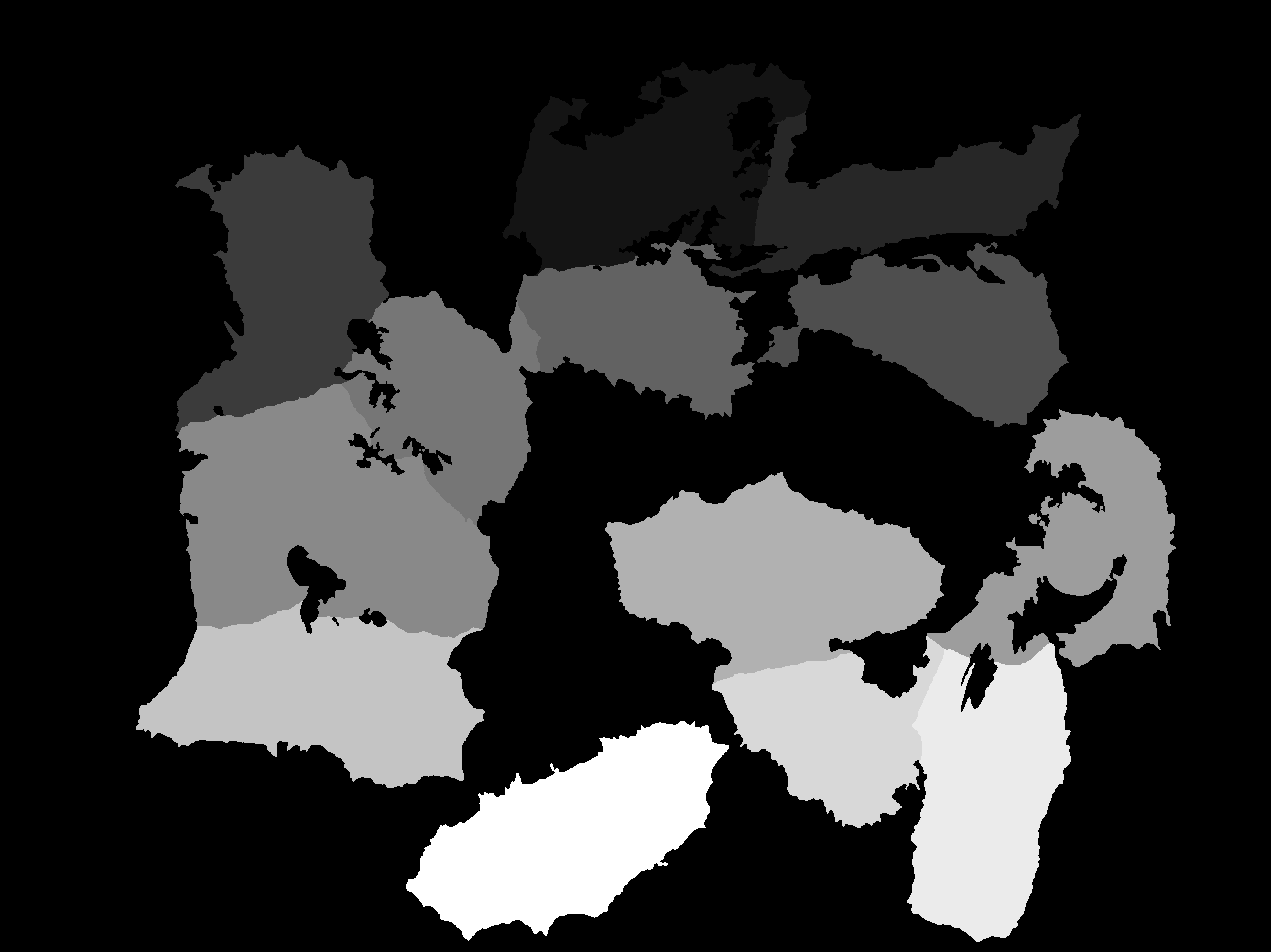

Supplement: S1 File — This file contains all scripts (CellProfiler v2.1.1 and MATLAB2016a) and data necessary to reproduce the information shown in Fig 3. (ZIP) [file pone.0180810.s001.zip › vitaminD_eColi_reproducibleResearchArchive/Results2016/A_5_c2_seg.tif]

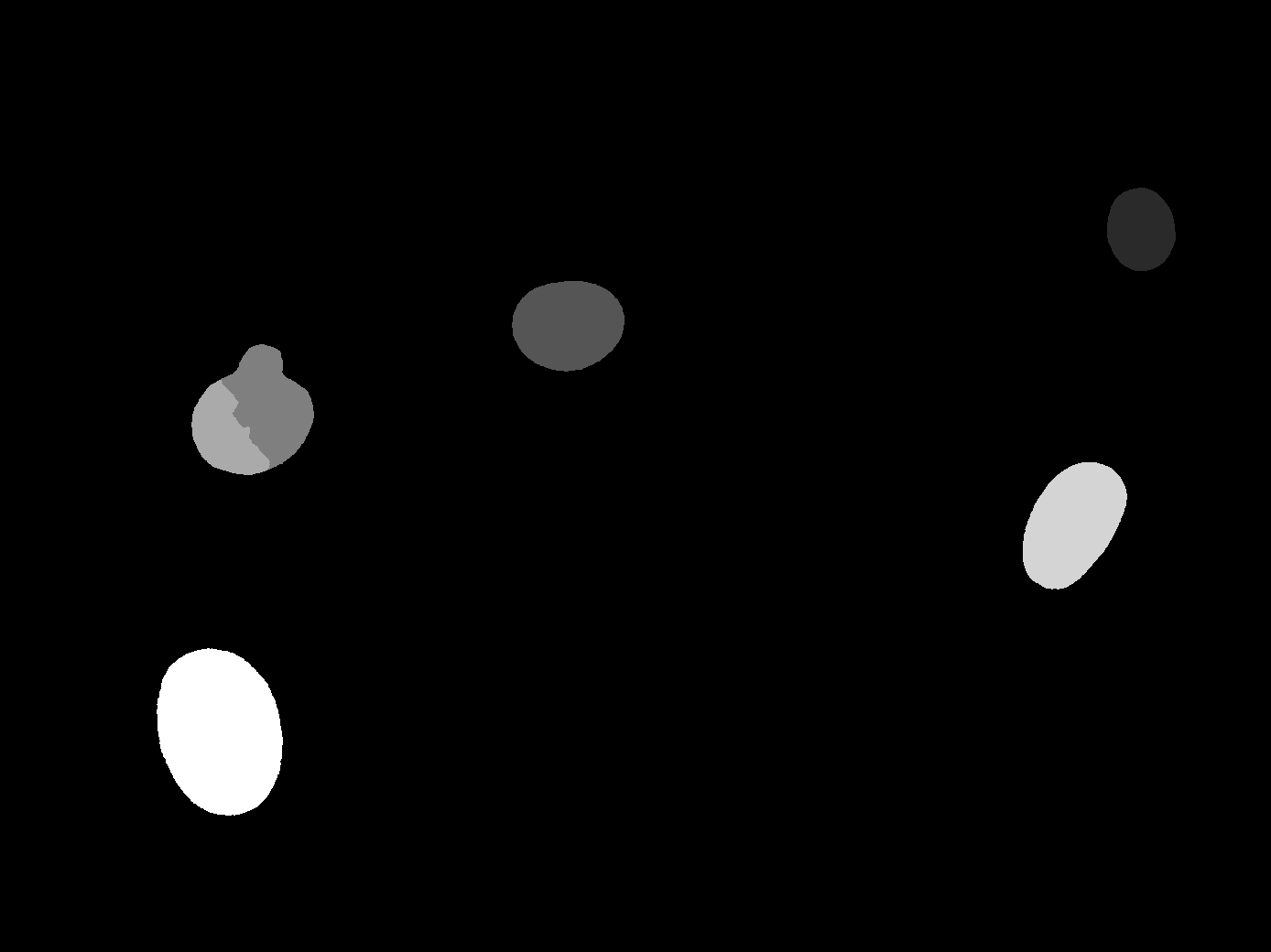

Supplement: S1 File — This file contains all scripts (CellProfiler v2.1.1 and MATLAB2016a) and data necessary to reproduce the information shown in Fig 3. (ZIP) [file pone.0180810.s001.zip › vitaminD_eColi_reproducibleResearchArchive/Results2016/A_6_c0_seg.tif]

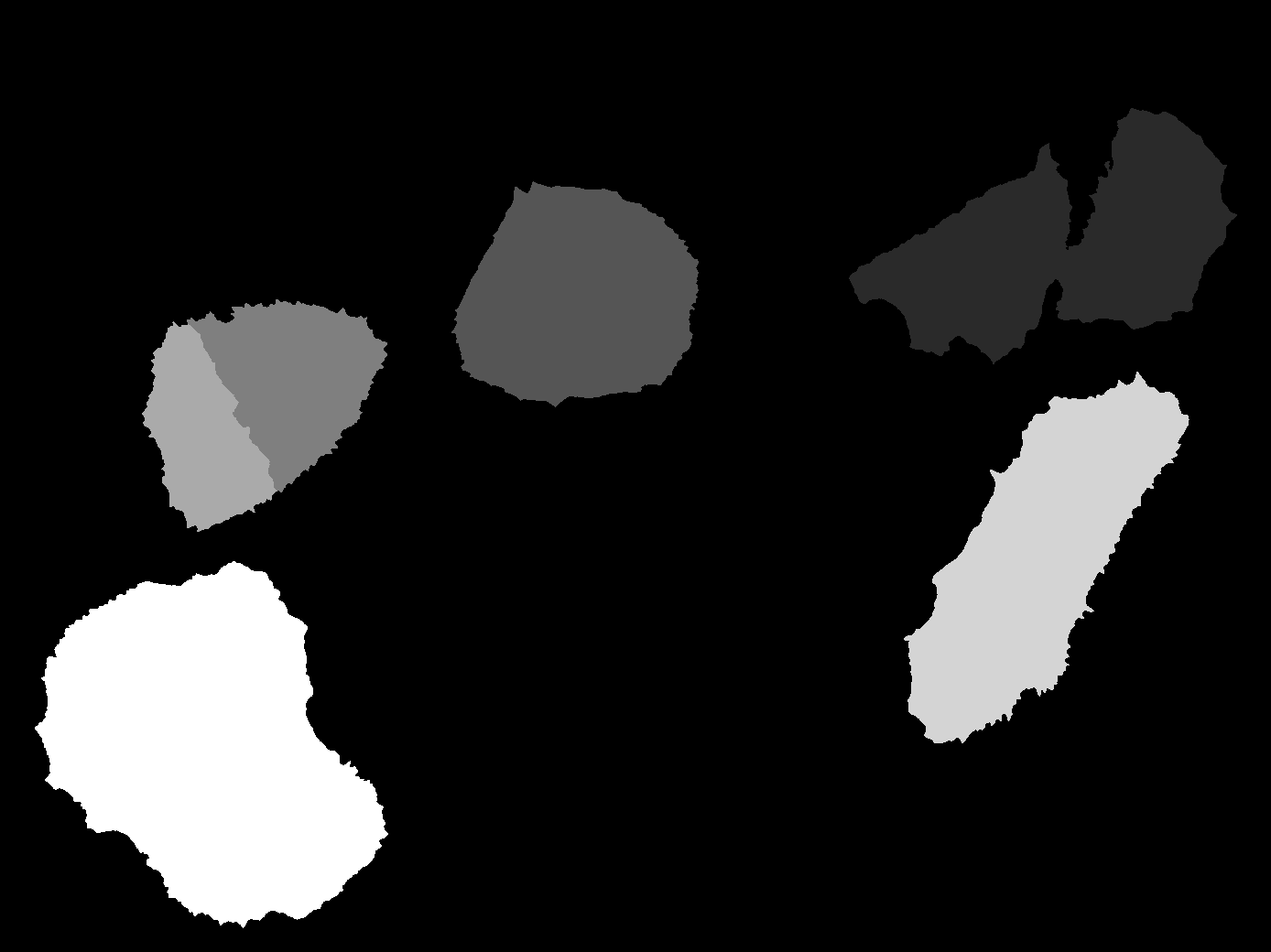

Supplement: S1 File — This file contains all scripts (CellProfiler v2.1.1 and MATLAB2016a) and data necessary to reproduce the information shown in Fig 3. (ZIP) [file pone.0180810.s001.zip › vitaminD_eColi_reproducibleResearchArchive/Results2016/A_6_c2_seg.tif]

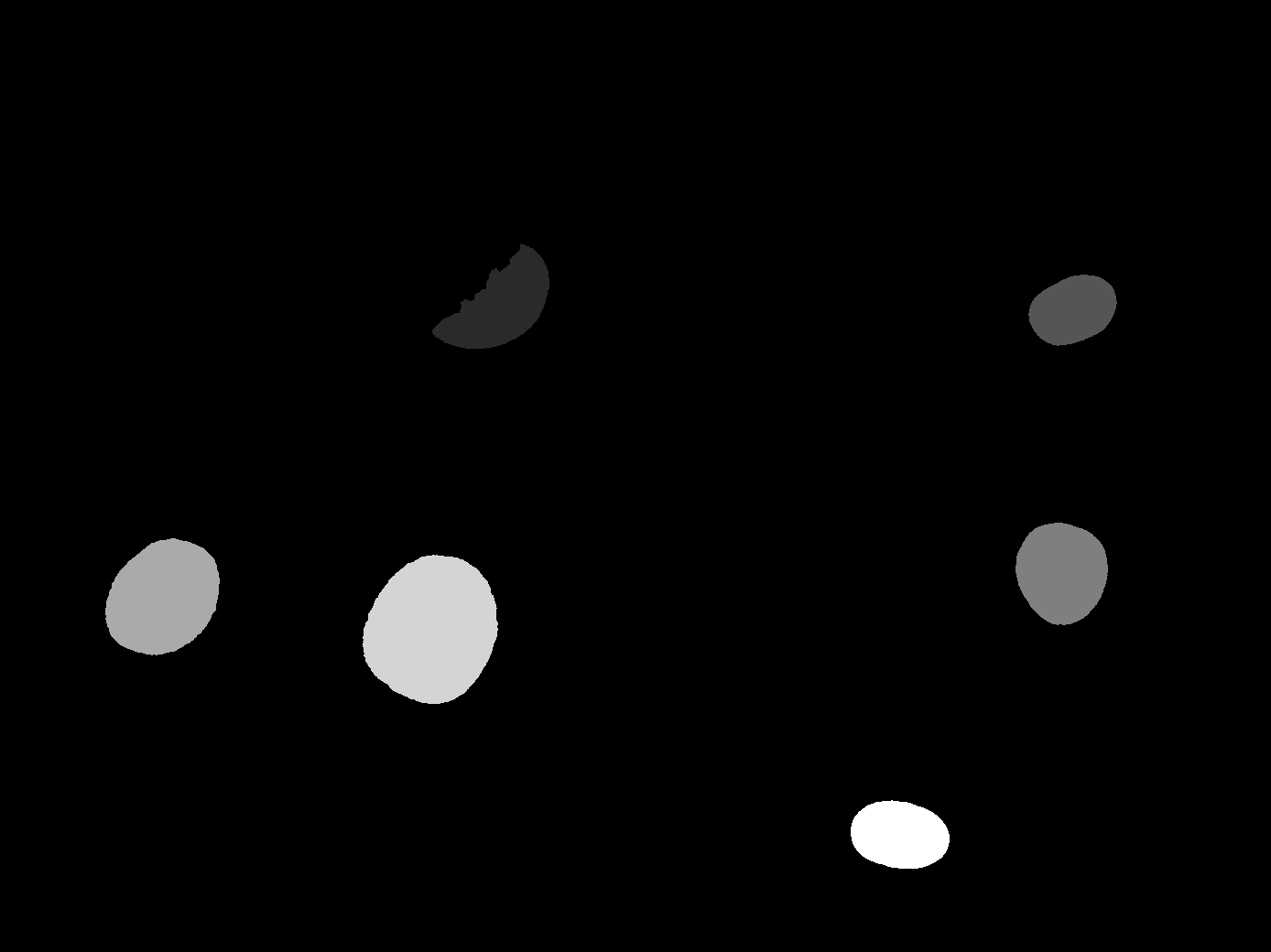

Supplement: S1 File — This file contains all scripts (CellProfiler v2.1.1 and MATLAB2016a) and data necessary to reproduce the information shown in Fig 3. (ZIP) [file pone.0180810.s001.zip › vitaminD_eColi_reproducibleResearchArchive/Results2016/A_7_c0_seg.tif]

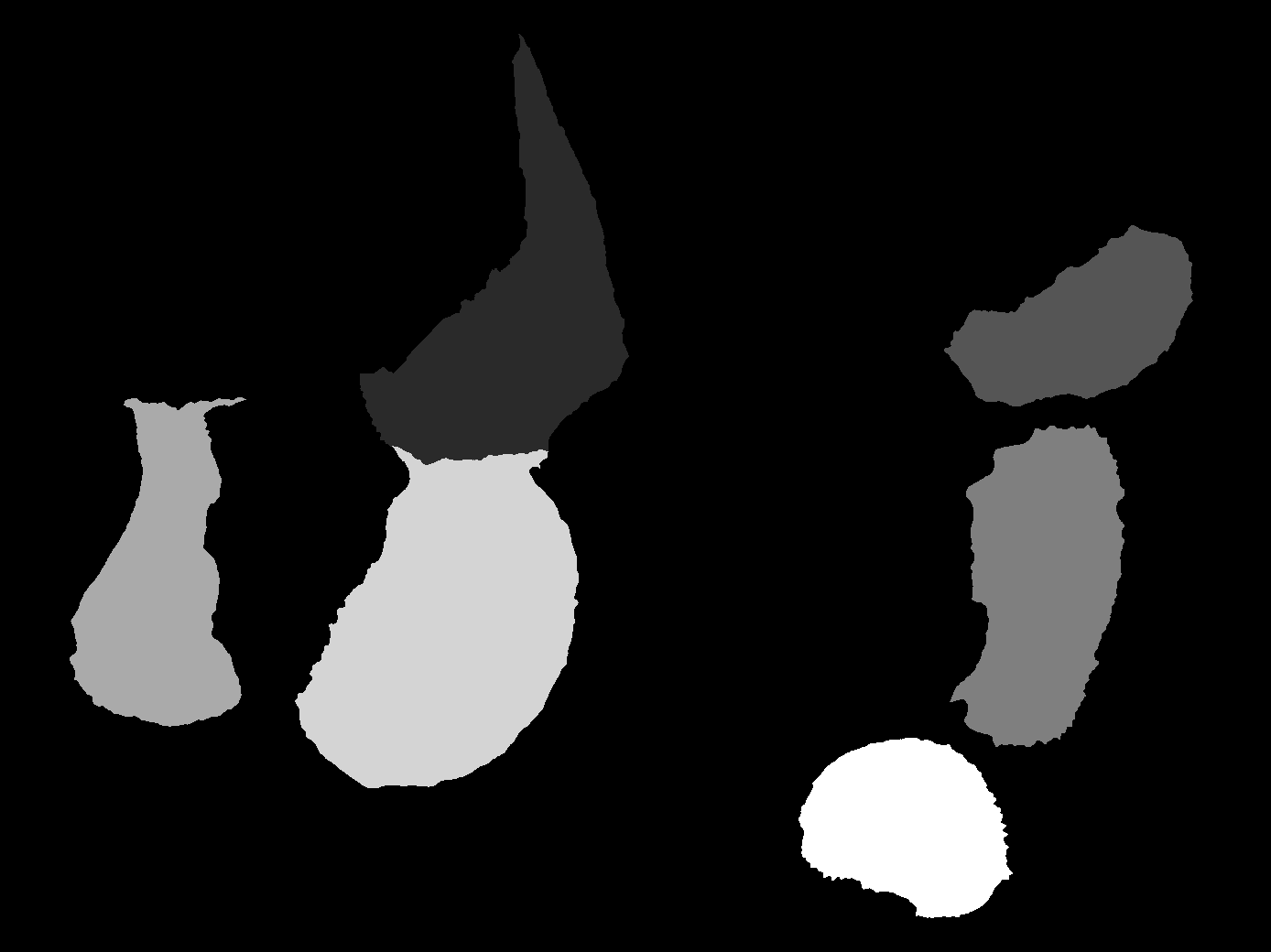

Supplement: S1 File — This file contains all scripts (CellProfiler v2.1.1 and MATLAB2016a) and data necessary to reproduce the information shown in Fig 3. (ZIP) [file pone.0180810.s001.zip › vitaminD_eColi_reproducibleResearchArchive/Results2016/A_7_c2_seg.tif]

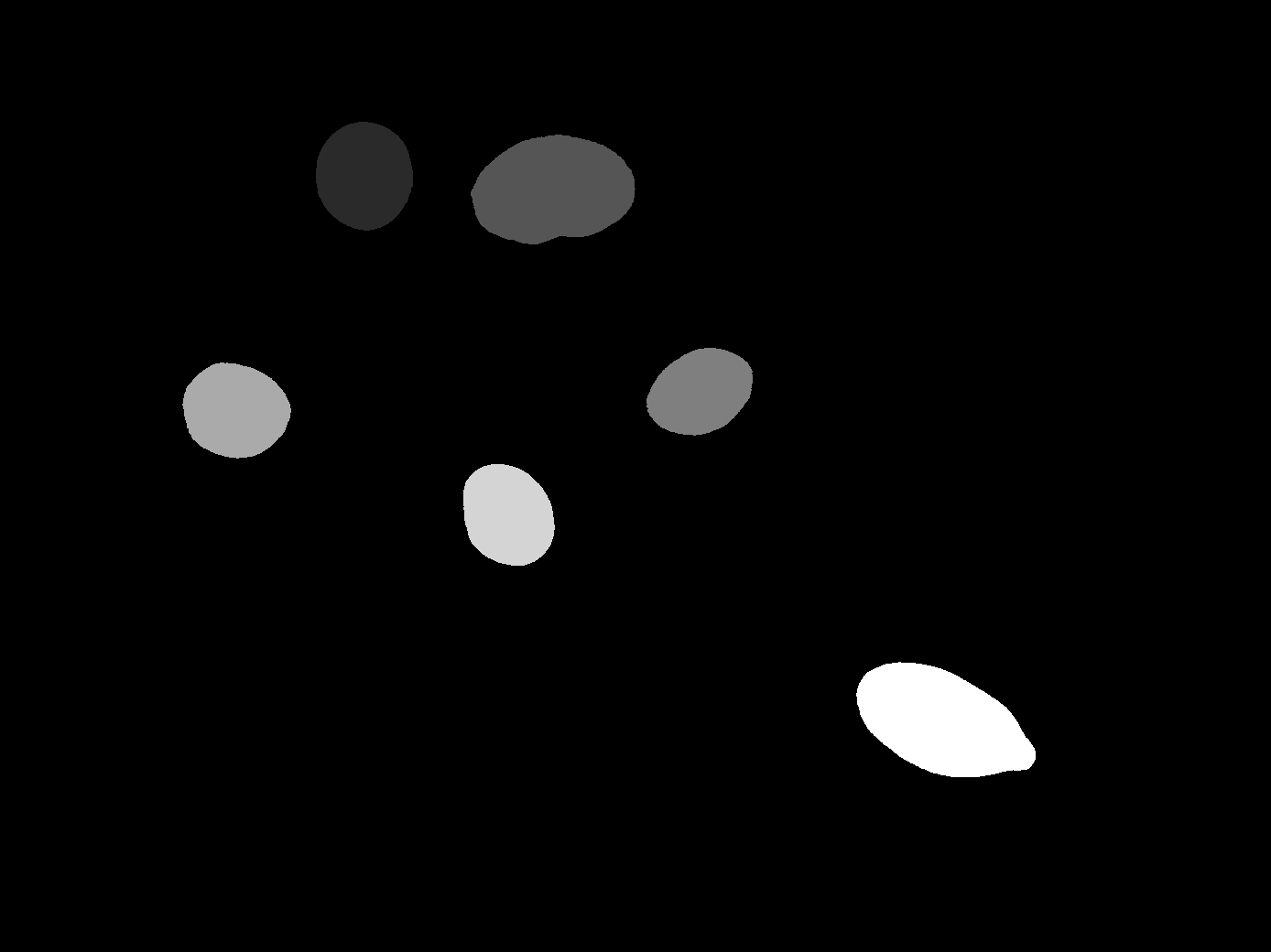

Supplement: S1 File — This file contains all scripts (CellProfiler v2.1.1 and MATLAB2016a) and data necessary to reproduce the information shown in Fig 3. (ZIP) [file pone.0180810.s001.zip › vitaminD_eColi_reproducibleResearchArchive/Results2016/A_8_c0_seg.tif]

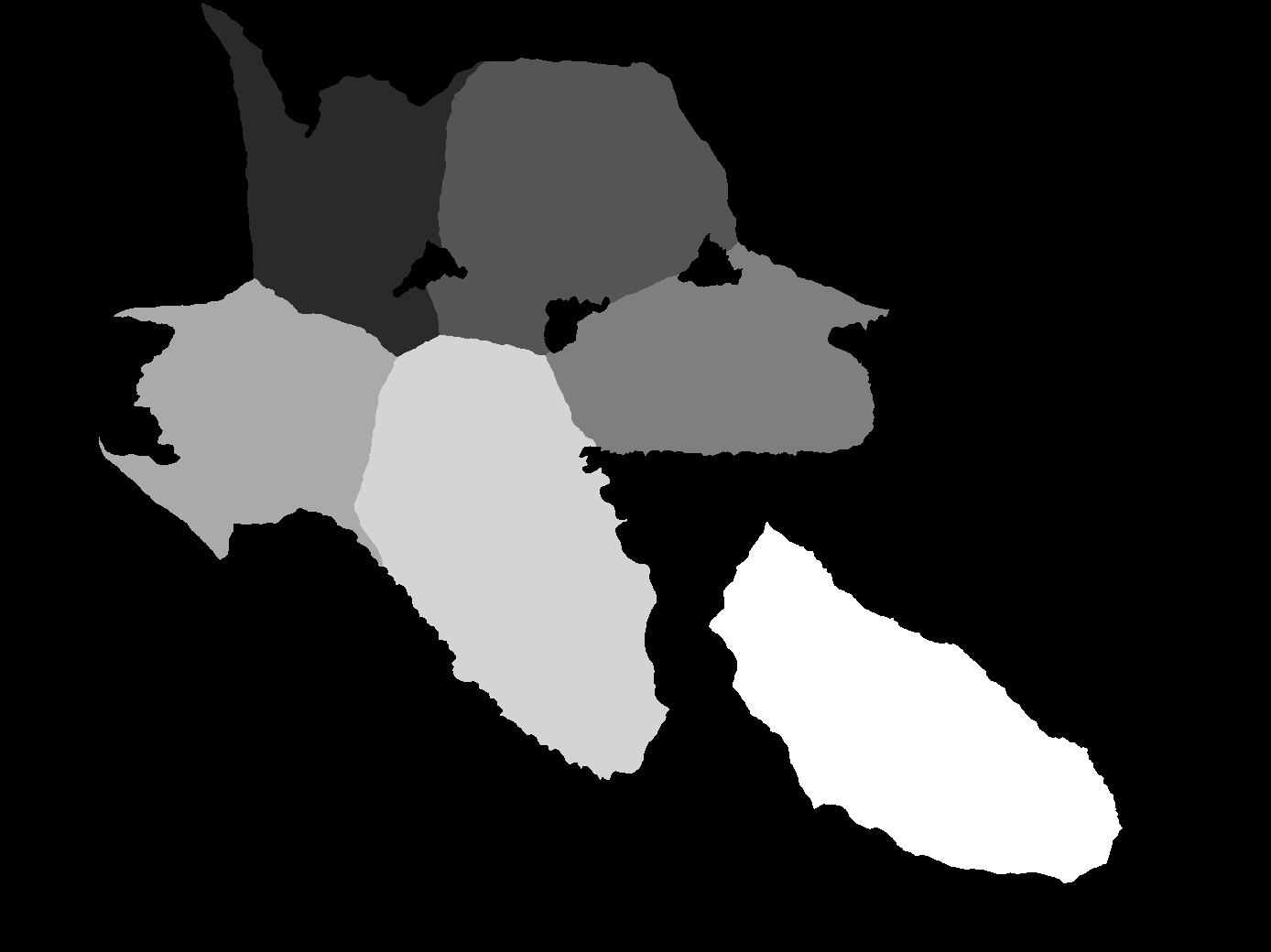

Supplement: S1 File — This file contains all scripts (CellProfiler v2.1.1 and MATLAB2016a) and data necessary to reproduce the information shown in Fig 3. (ZIP) [file pone.0180810.s001.zip › vitaminD_eColi_reproducibleResearchArchive/Results2016/A_8_c2_seg.tif]

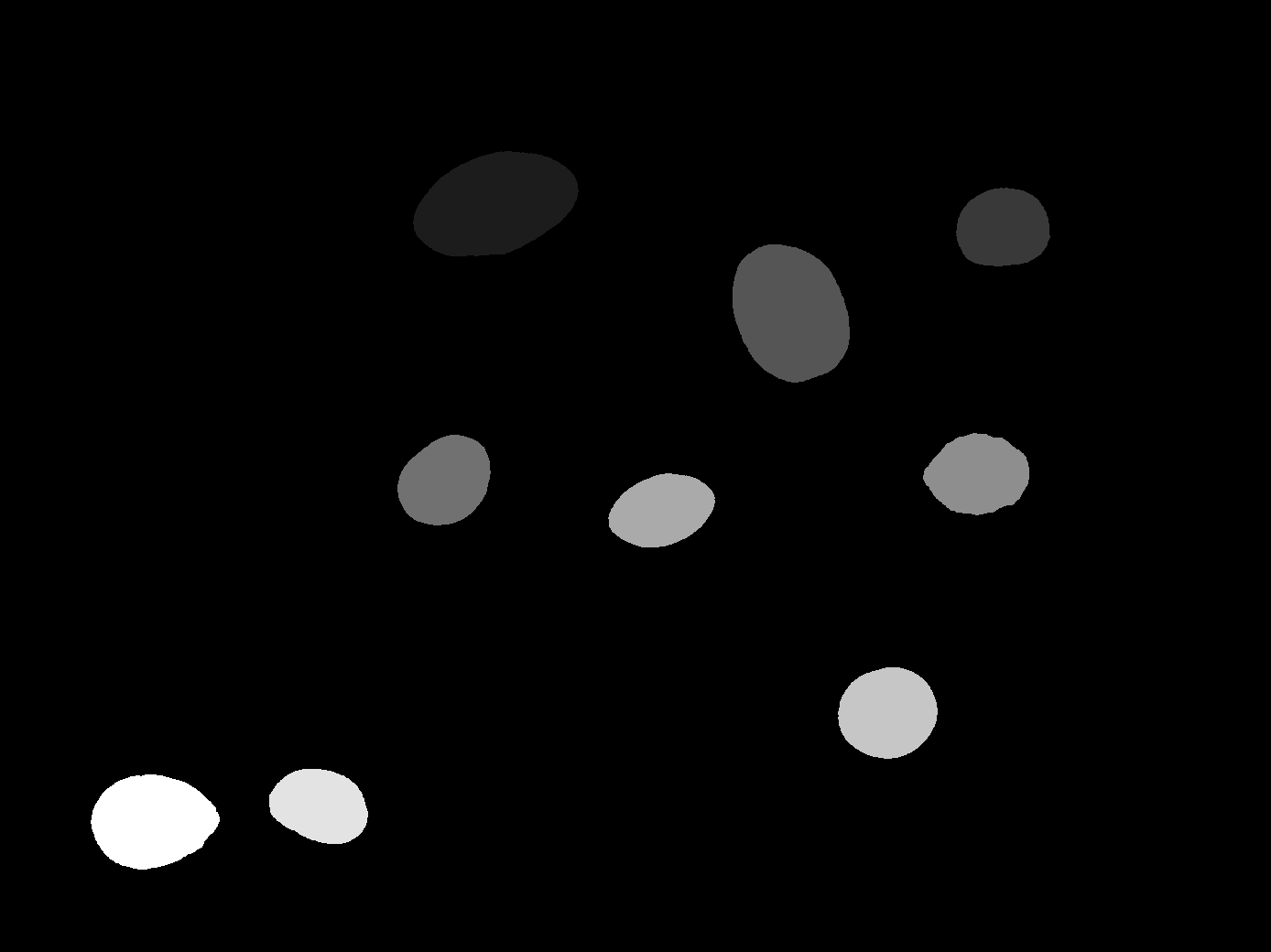

Supplement: S1 File — This file contains all scripts (CellProfiler v2.1.1 and MATLAB2016a) and data necessary to reproduce the information shown in Fig 3. (ZIP) [file pone.0180810.s001.zip › vitaminD_eColi_reproducibleResearchArchive/Results2016/A_9_c0_seg.tif]

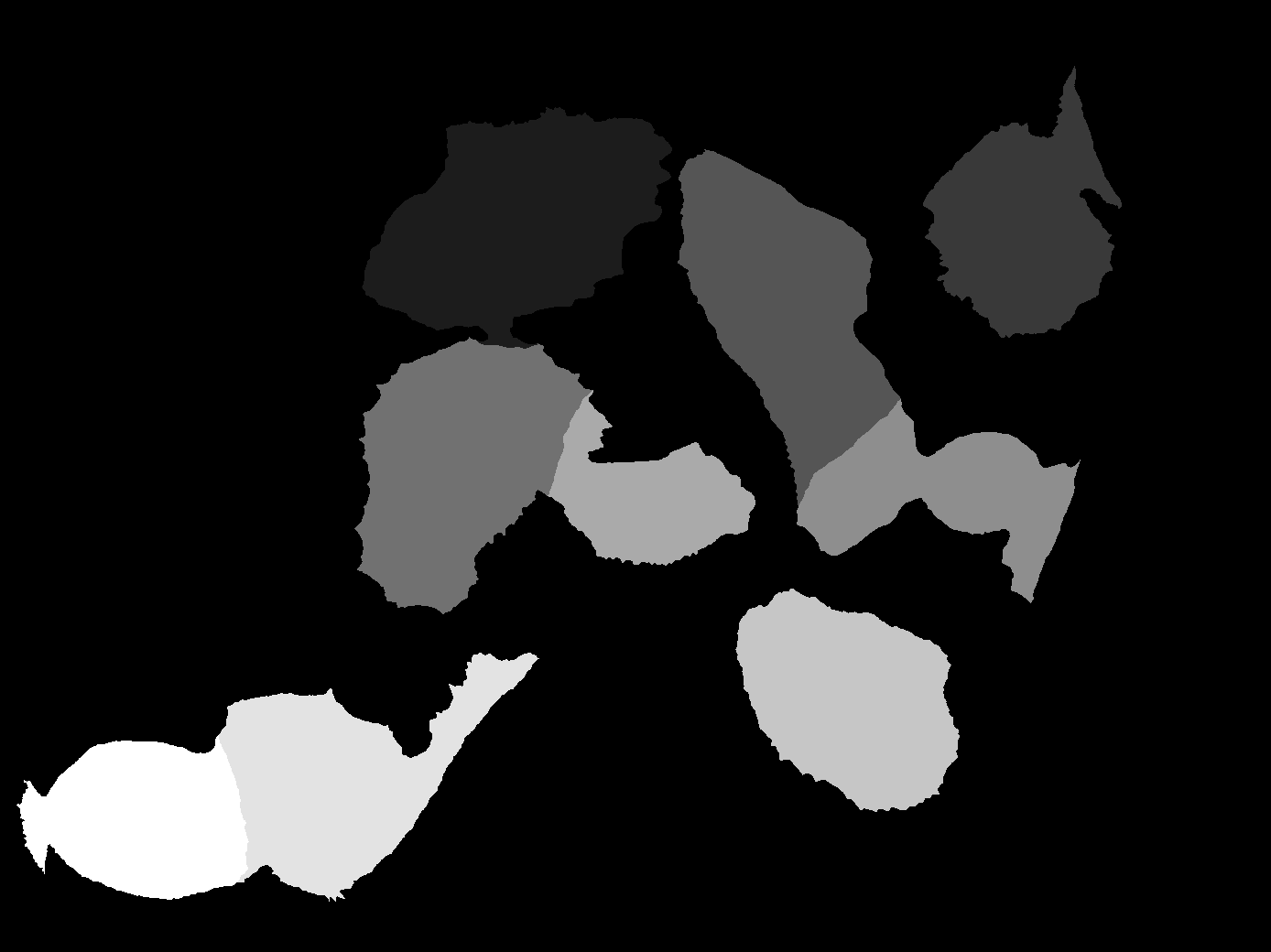

Supplement: S1 File — This file contains all scripts (CellProfiler v2.1.1 and MATLAB2016a) and data necessary to reproduce the information shown in Fig 3. (ZIP) [file pone.0180810.s001.zip › vitaminD_eColi_reproducibleResearchArchive/Results2016/A_9_c2_seg.tif]

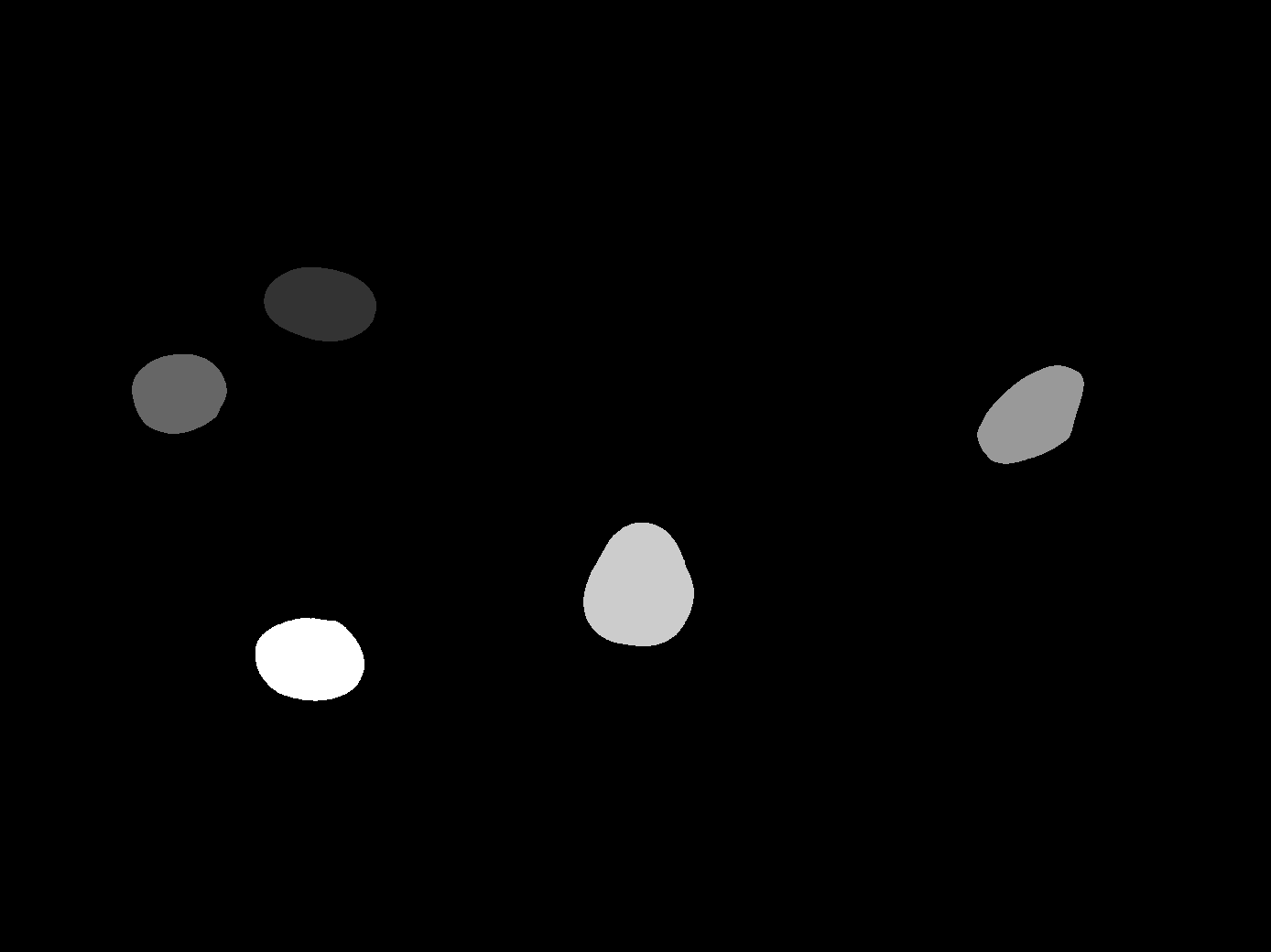

Supplement: S1 File — This file contains all scripts (CellProfiler v2.1.1 and MATLAB2016a) and data necessary to reproduce the information shown in Fig 3. (ZIP) [file pone.0180810.s001.zip › vitaminD_eColi_reproducibleResearchArchive/Results2016/B_10_c0_seg.tif]

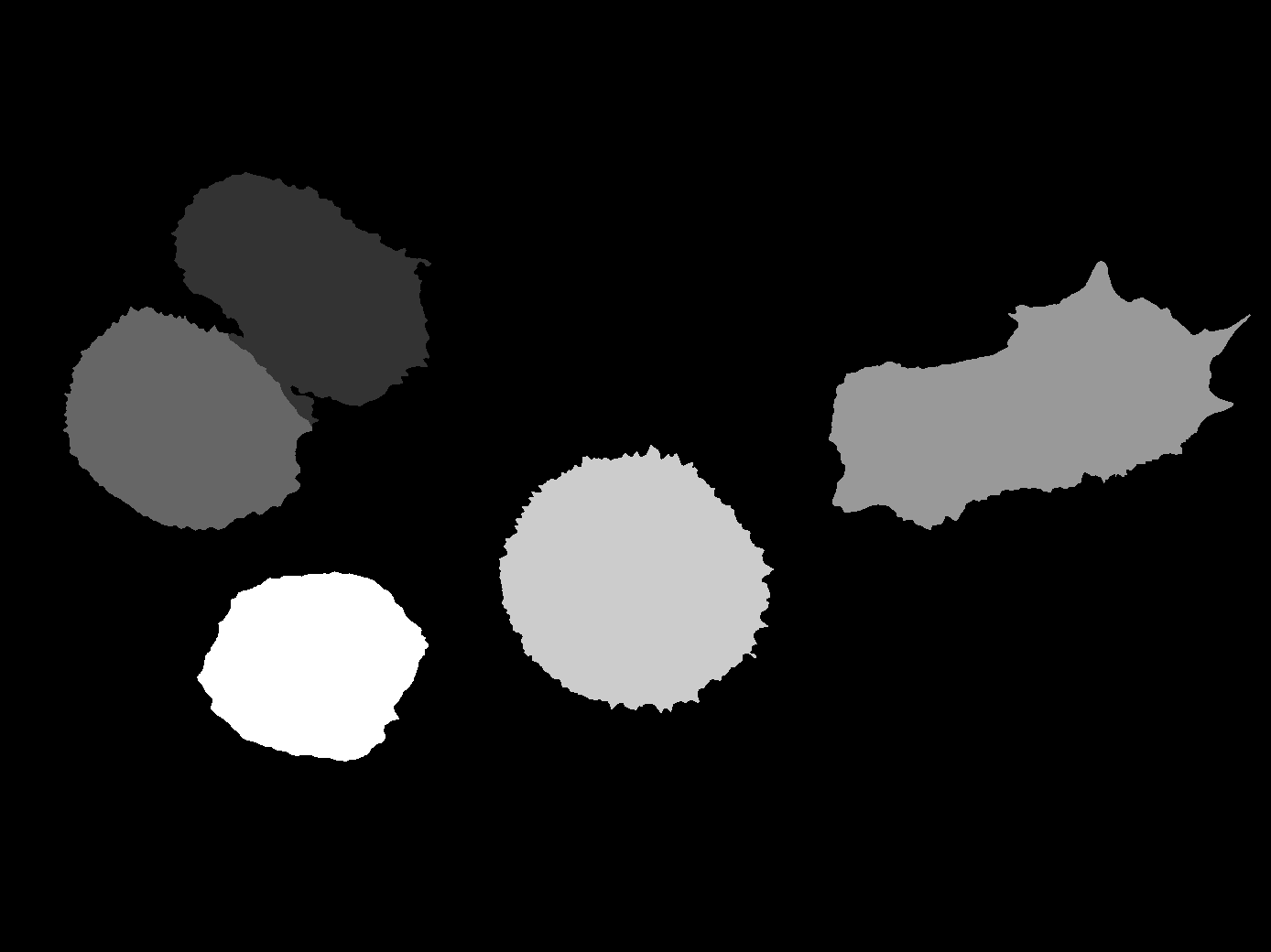

Supplement: S1 File — This file contains all scripts (CellProfiler v2.1.1 and MATLAB2016a) and data necessary to reproduce the information shown in Fig 3. (ZIP) [file pone.0180810.s001.zip › vitaminD_eColi_reproducibleResearchArchive/Results2016/B_10_c2_seg.tif]

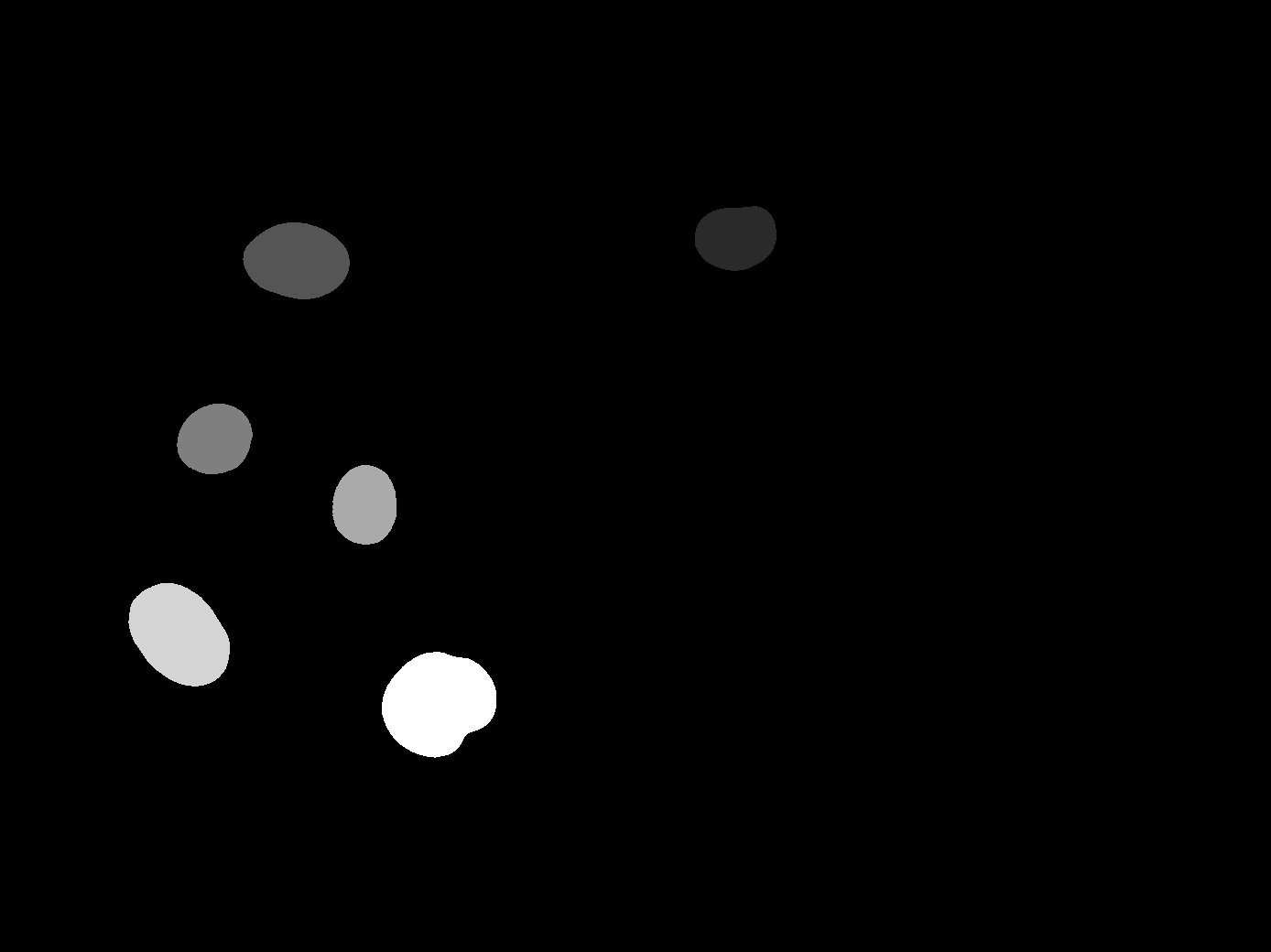

Supplement: S1 File — This file contains all scripts (CellProfiler v2.1.1 and MATLAB2016a) and data necessary to reproduce the information shown in Fig 3. (ZIP) [file pone.0180810.s001.zip › vitaminD_eColi_reproducibleResearchArchive/Results2016/B_11_c0_seg.tif]

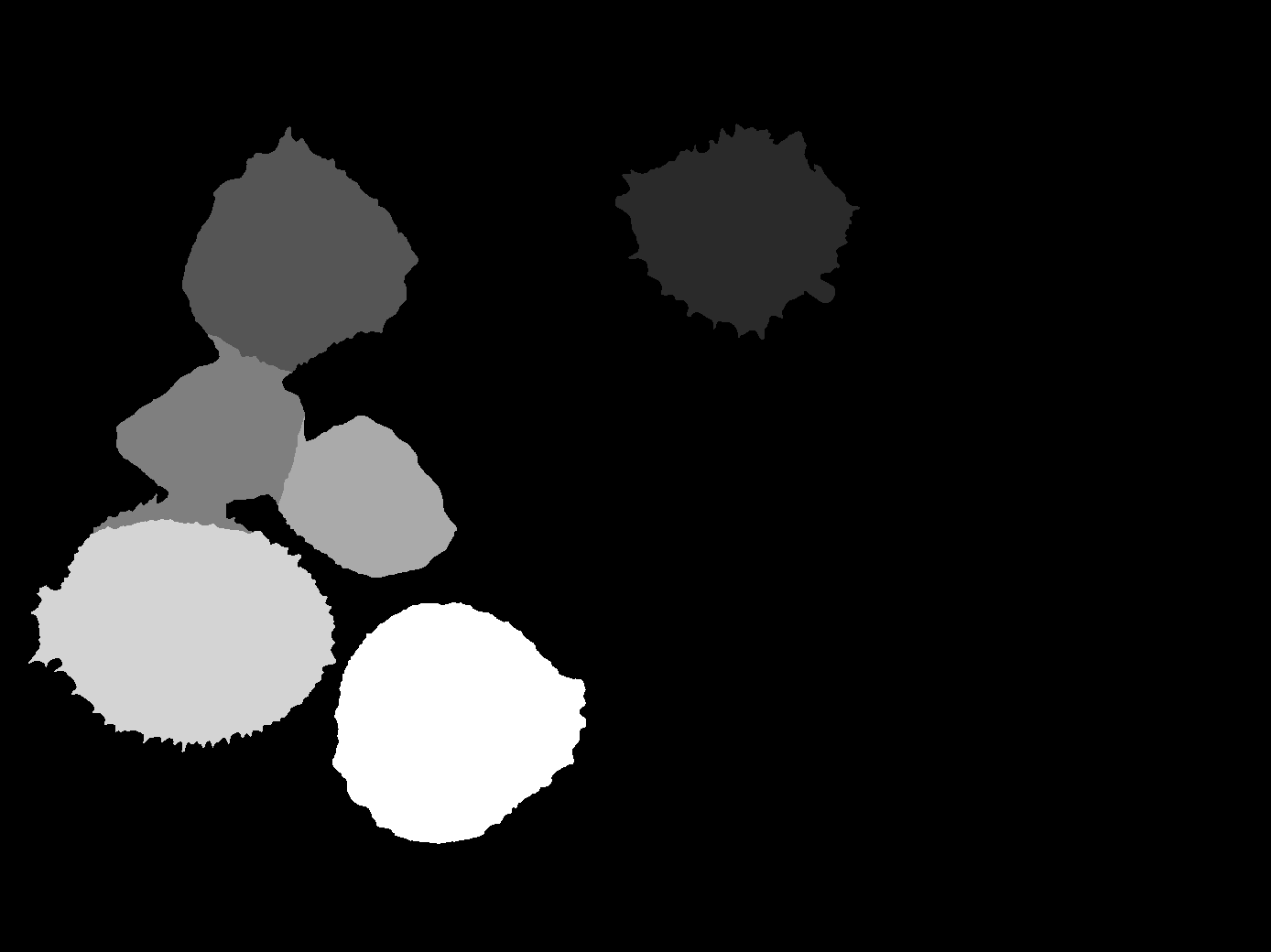

Supplement: S1 File — This file contains all scripts (CellProfiler v2.1.1 and MATLAB2016a) and data necessary to reproduce the information shown in Fig 3. (ZIP) [file pone.0180810.s001.zip › vitaminD_eColi_reproducibleResearchArchive/Results2016/B_11_c2_seg.tif]

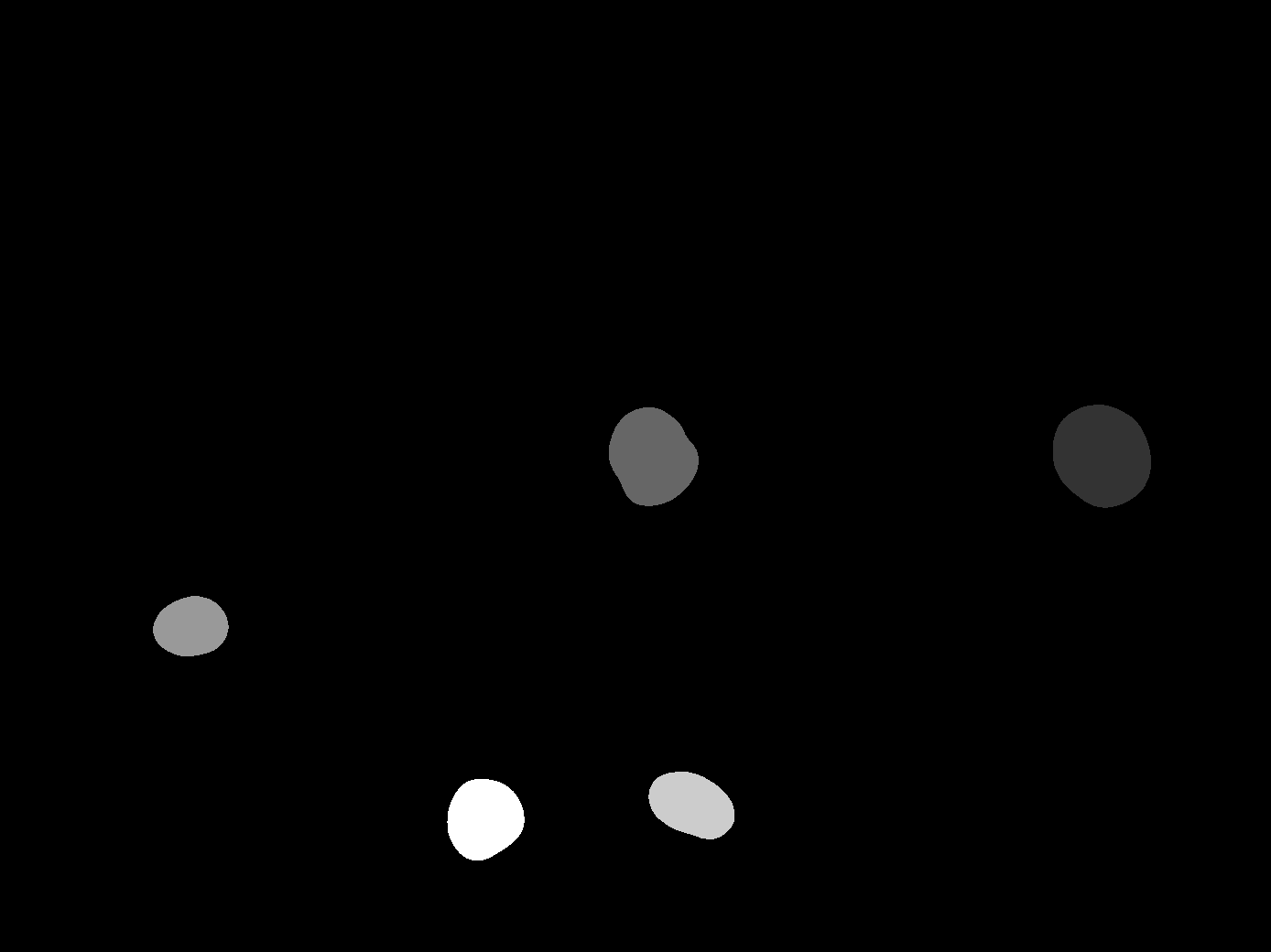

Supplement: S1 File — This file contains all scripts (CellProfiler v2.1.1 and MATLAB2016a) and data necessary to reproduce the information shown in Fig 3. (ZIP) [file pone.0180810.s001.zip › vitaminD_eColi_reproducibleResearchArchive/Results2016/B_12_c0_seg.tif]

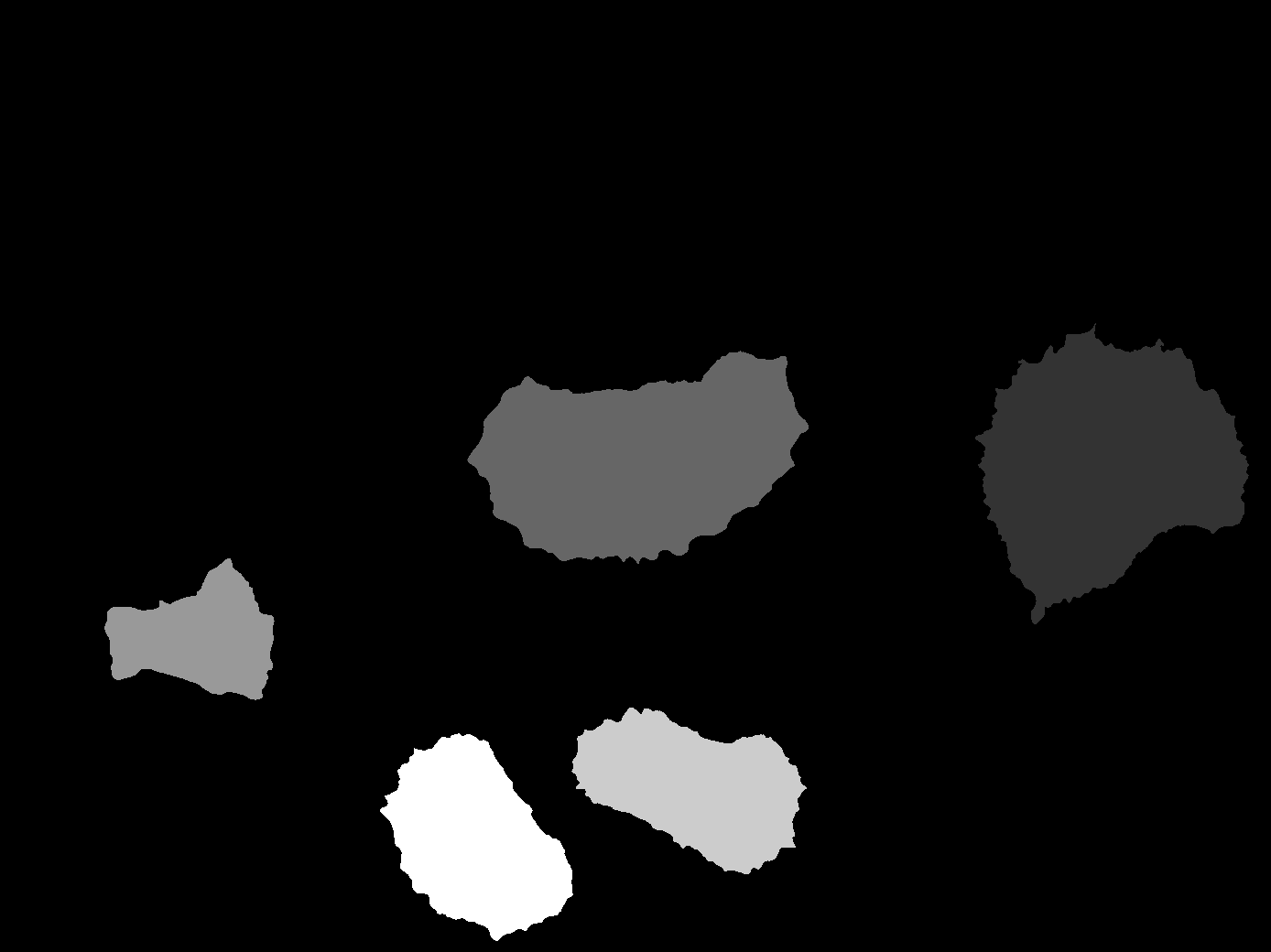

Supplement: S1 File — This file contains all scripts (CellProfiler v2.1.1 and MATLAB2016a) and data necessary to reproduce the information shown in Fig 3. (ZIP) [file pone.0180810.s001.zip › vitaminD_eColi_reproducibleResearchArchive/Results2016/B_12_c2_seg.tif]

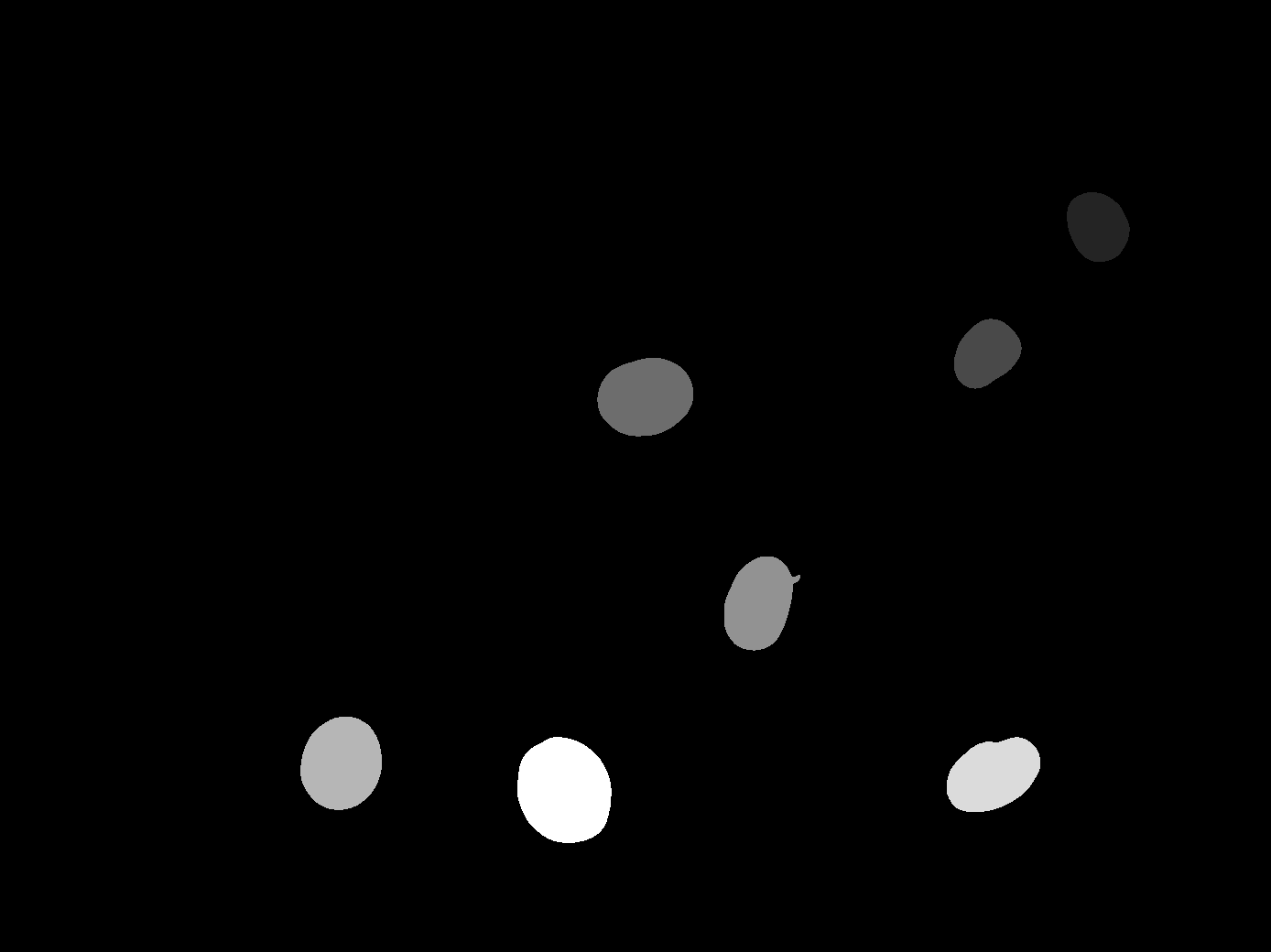

Supplement: S1 File — This file contains all scripts (CellProfiler v2.1.1 and MATLAB2016a) and data necessary to reproduce the information shown in Fig 3. (ZIP) [file pone.0180810.s001.zip › vitaminD_eColi_reproducibleResearchArchive/Results2016/B_13_c0_seg.tif]

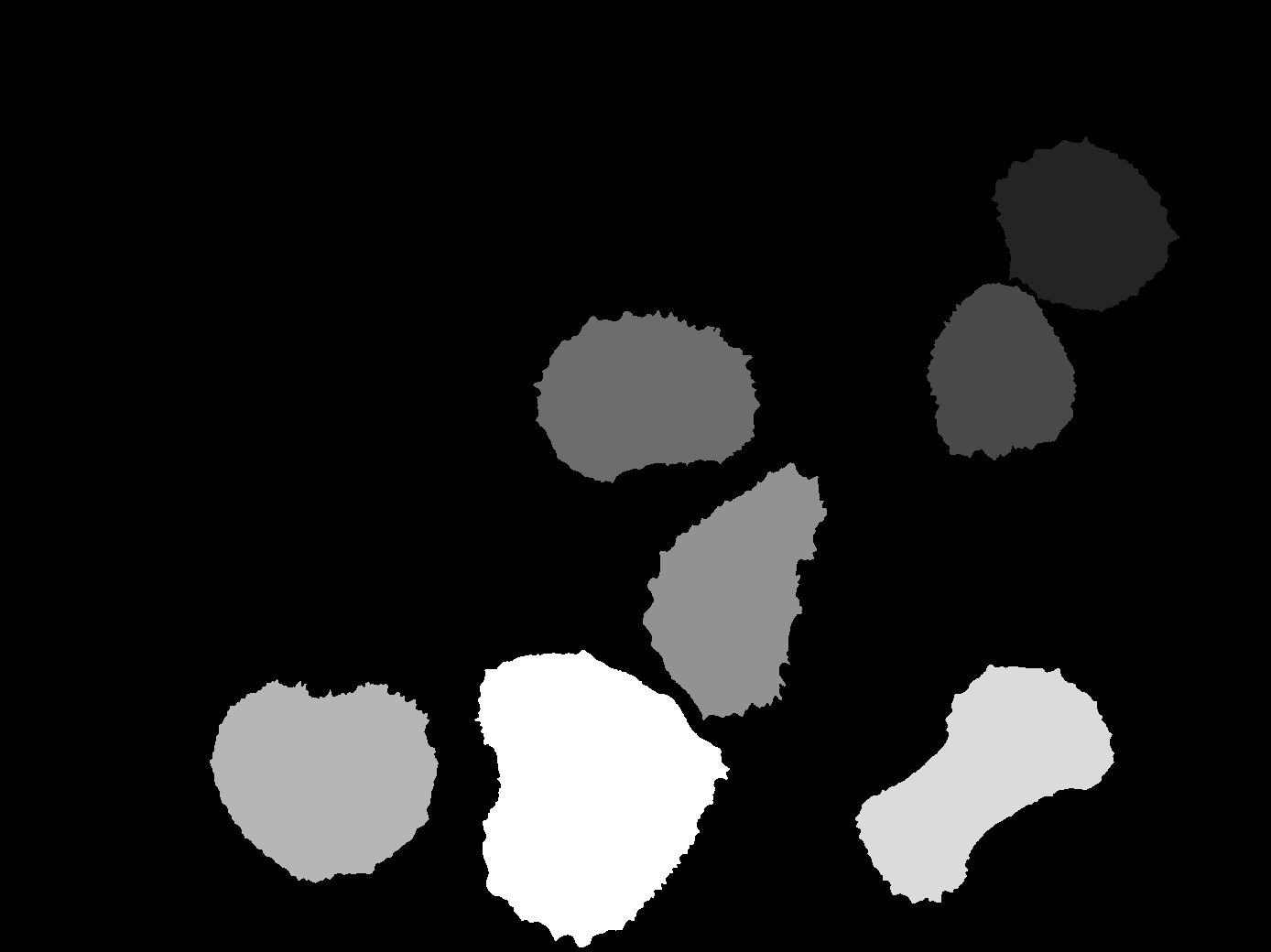

Supplement: S1 File — This file contains all scripts (CellProfiler v2.1.1 and MATLAB2016a) and data necessary to reproduce the information shown in Fig 3. (ZIP) [file pone.0180810.s001.zip › vitaminD_eColi_reproducibleResearchArchive/Results2016/B_13_c2_seg.tif]

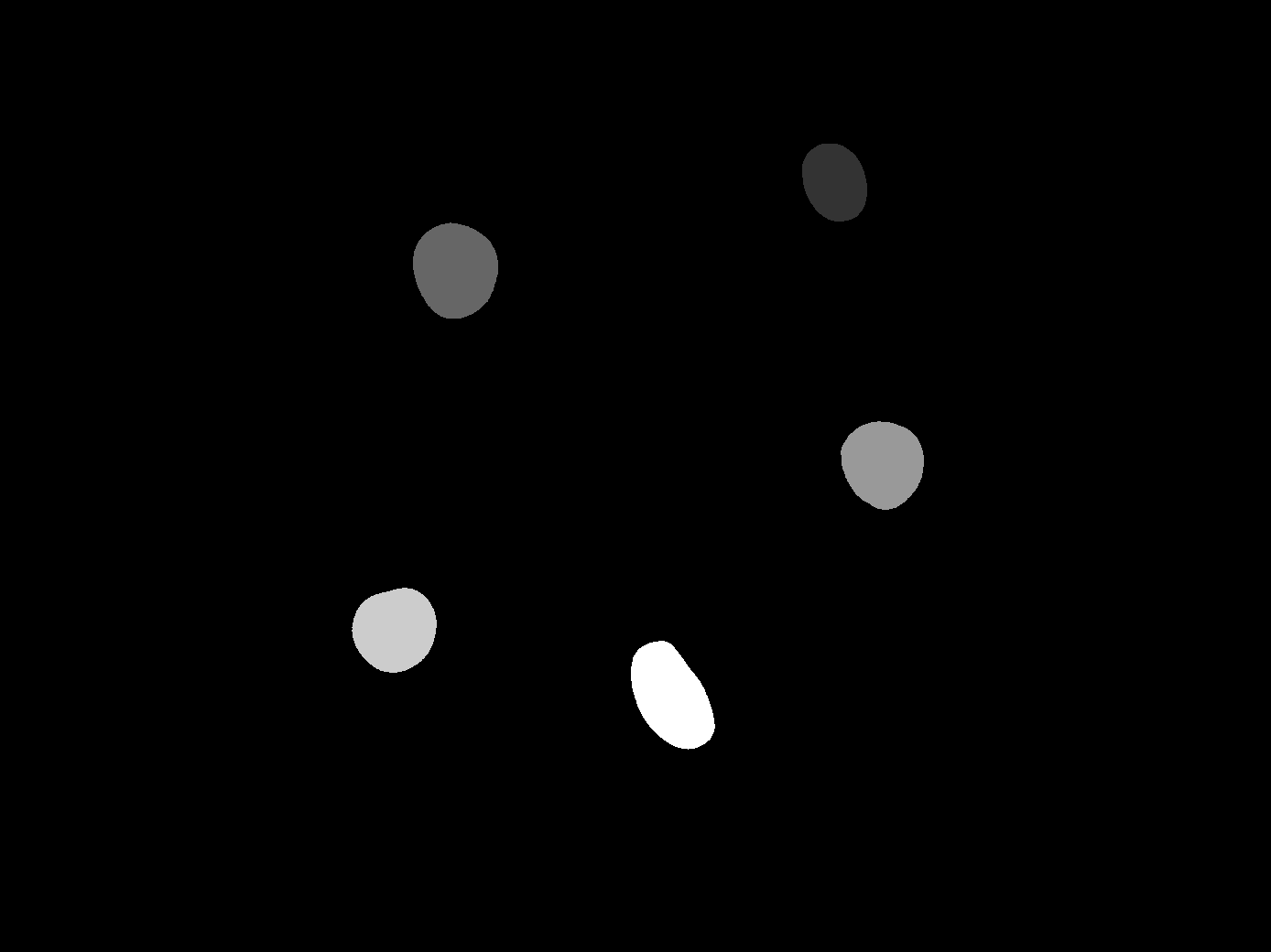

Supplement: S1 File — This file contains all scripts (CellProfiler v2.1.1 and MATLAB2016a) and data necessary to reproduce the information shown in Fig 3. (ZIP) [file pone.0180810.s001.zip › vitaminD_eColi_reproducibleResearchArchive/Results2016/B_14_c0_seg.tif]

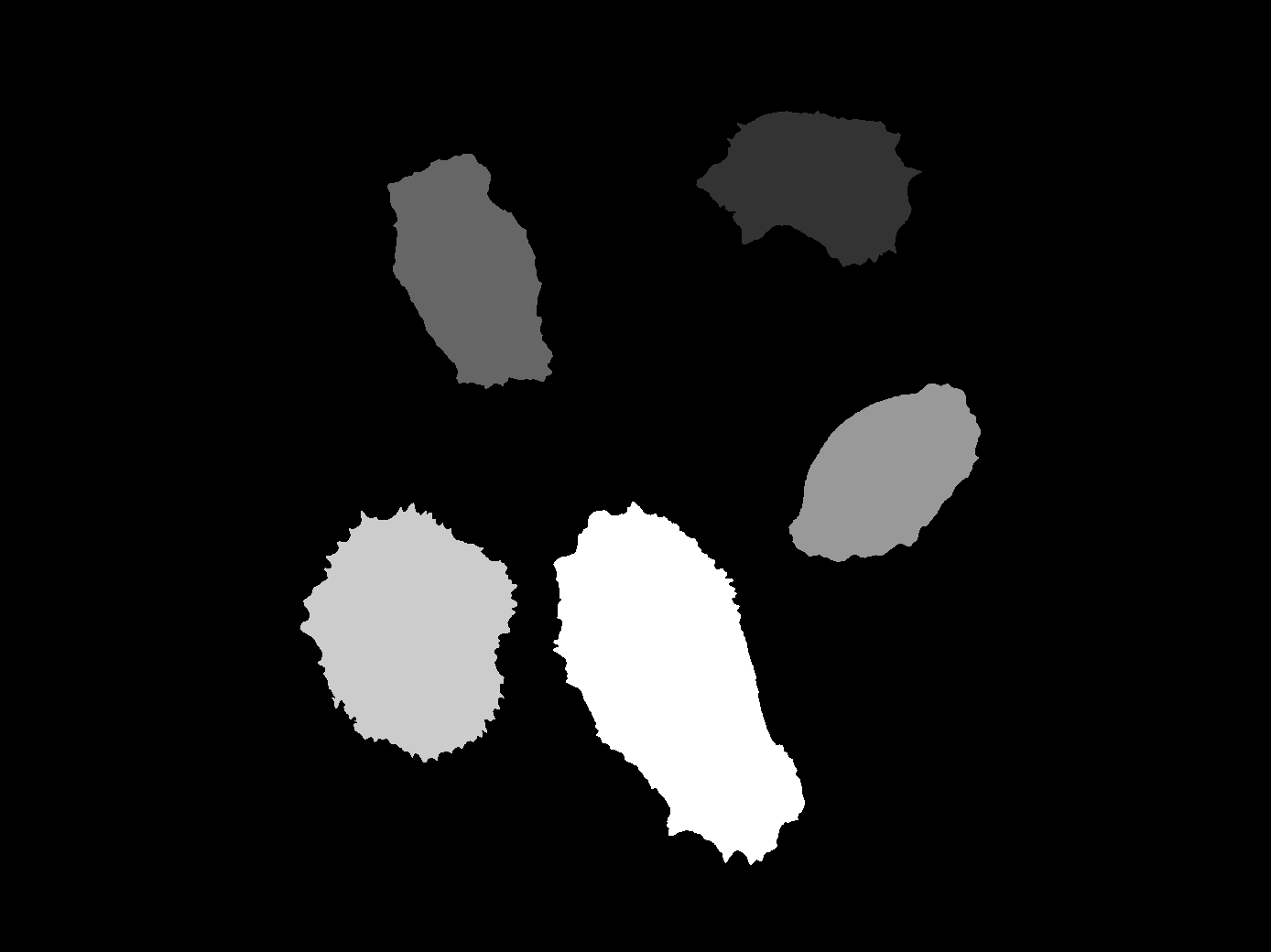

Supplement: S1 File — This file contains all scripts (CellProfiler v2.1.1 and MATLAB2016a) and data necessary to reproduce the information shown in Fig 3. (ZIP) [file pone.0180810.s001.zip › vitaminD_eColi_reproducibleResearchArchive/Results2016/B_14_c2_seg.tif]

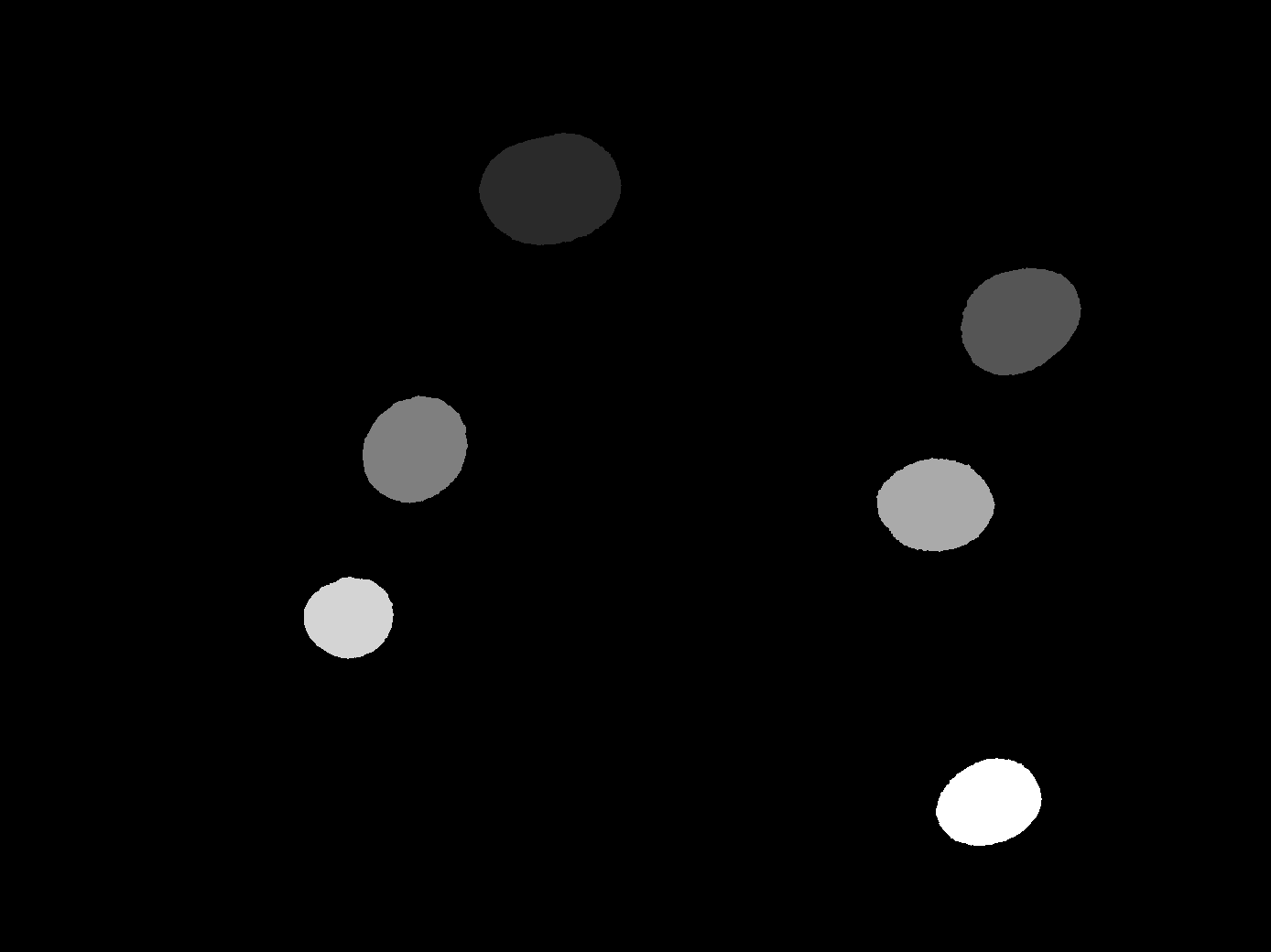

Supplement: S1 File — This file contains all scripts (CellProfiler v2.1.1 and MATLAB2016a) and data necessary to reproduce the information shown in Fig 3. (ZIP) [file pone.0180810.s001.zip › vitaminD_eColi_reproducibleResearchArchive/Results2016/B_15_c0_seg.tif]

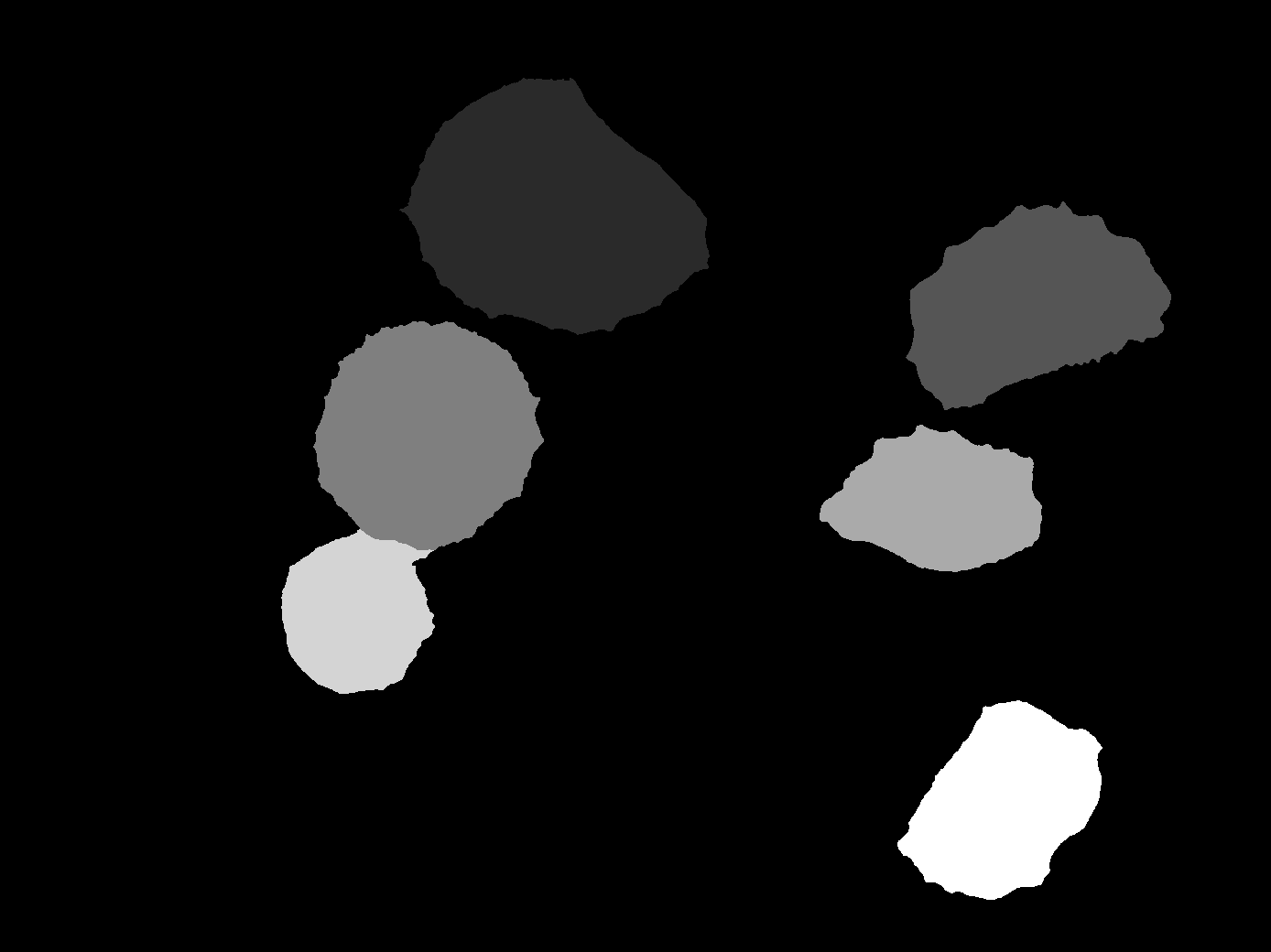

Supplement: S1 File — This file contains all scripts (CellProfiler v2.1.1 and MATLAB2016a) and data necessary to reproduce the information shown in Fig 3. (ZIP) [file pone.0180810.s001.zip › vitaminD_eColi_reproducibleResearchArchive/Results2016/B_15_c2_seg.tif]

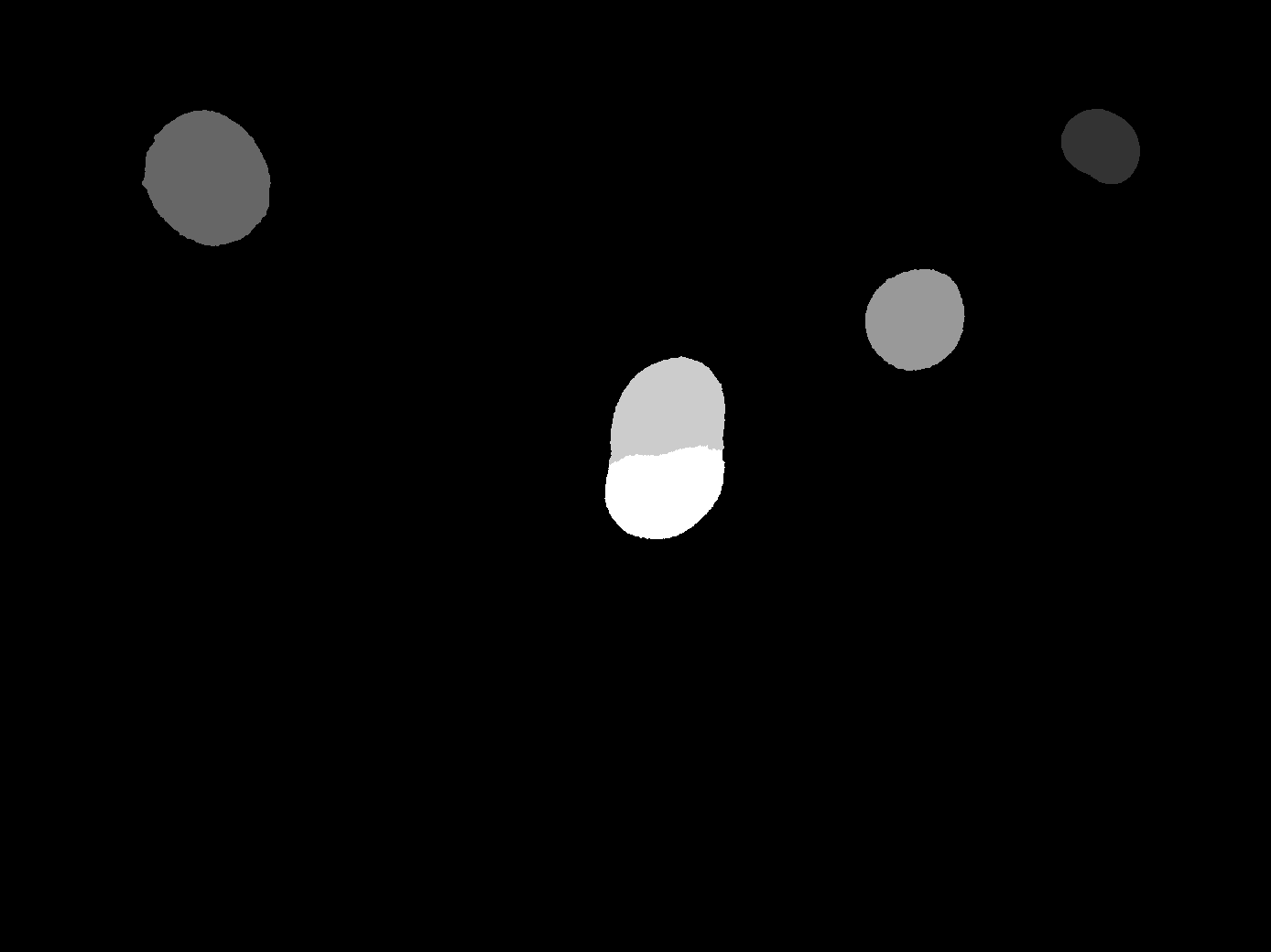

Supplement: S1 File — This file contains all scripts (CellProfiler v2.1.1 and MATLAB2016a) and data necessary to reproduce the information shown in Fig 3. (ZIP) [file pone.0180810.s001.zip › vitaminD_eColi_reproducibleResearchArchive/Results2016/B_16_c0_seg.tif]

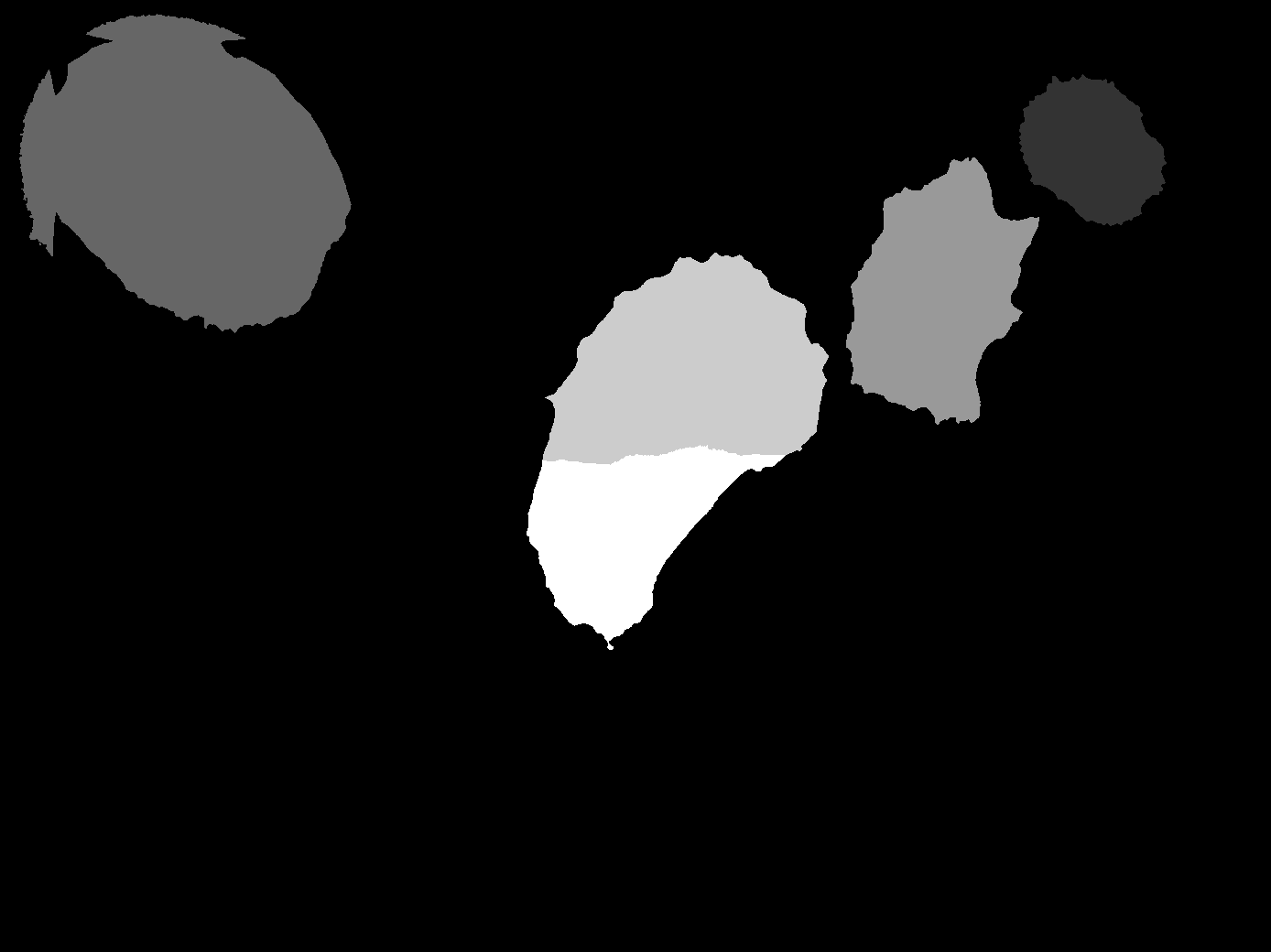

Supplement: S1 File — This file contains all scripts (CellProfiler v2.1.1 and MATLAB2016a) and data necessary to reproduce the information shown in Fig 3. (ZIP) [file pone.0180810.s001.zip › vitaminD_eColi_reproducibleResearchArchive/Results2016/B_16_c2_seg.tif]

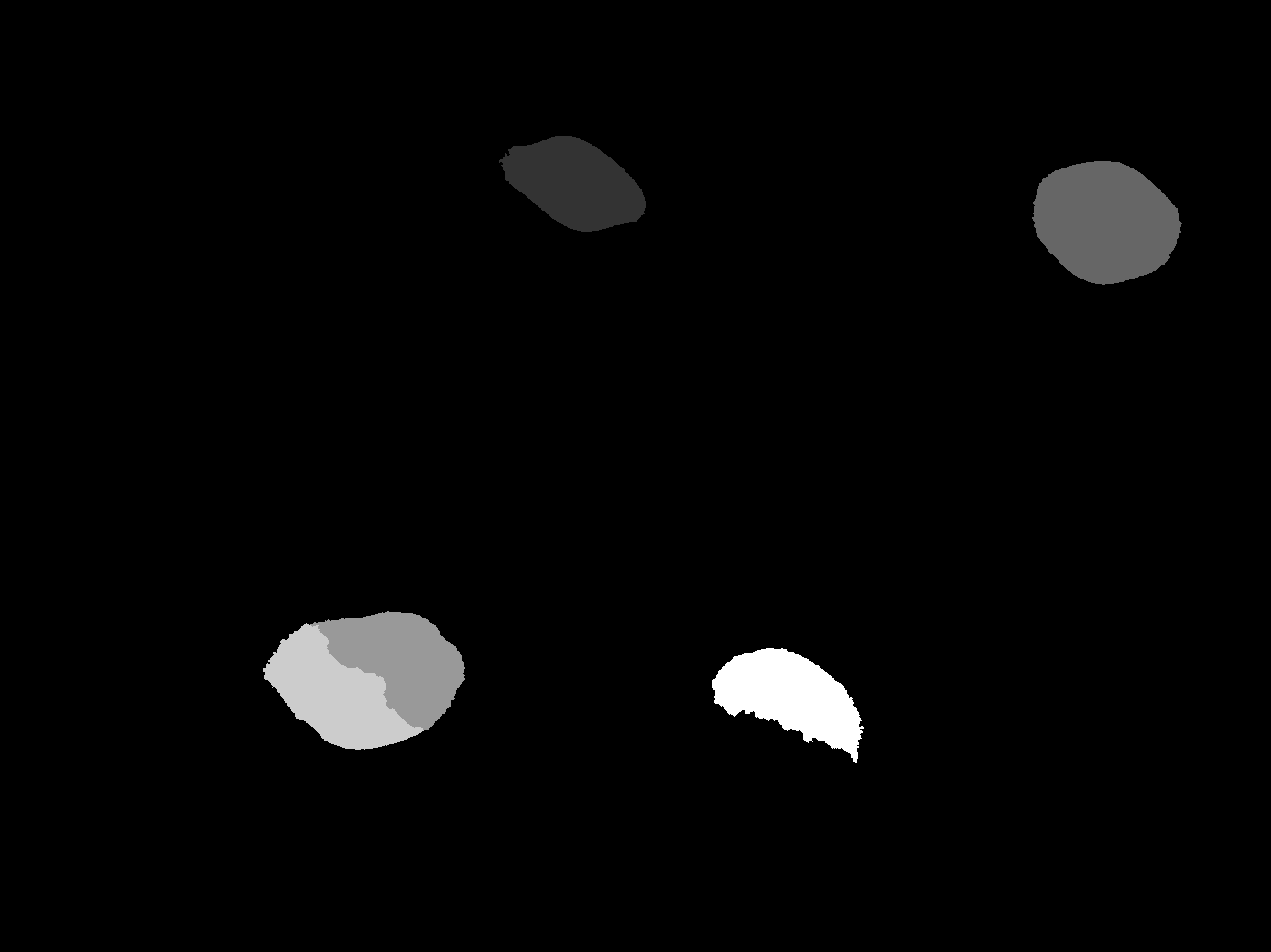

Supplement: S1 File — This file contains all scripts (CellProfiler v2.1.1 and MATLAB2016a) and data necessary to reproduce the information shown in Fig 3. (ZIP) [file pone.0180810.s001.zip › vitaminD_eColi_reproducibleResearchArchive/Results2016/B_17_c0_seg.tif]

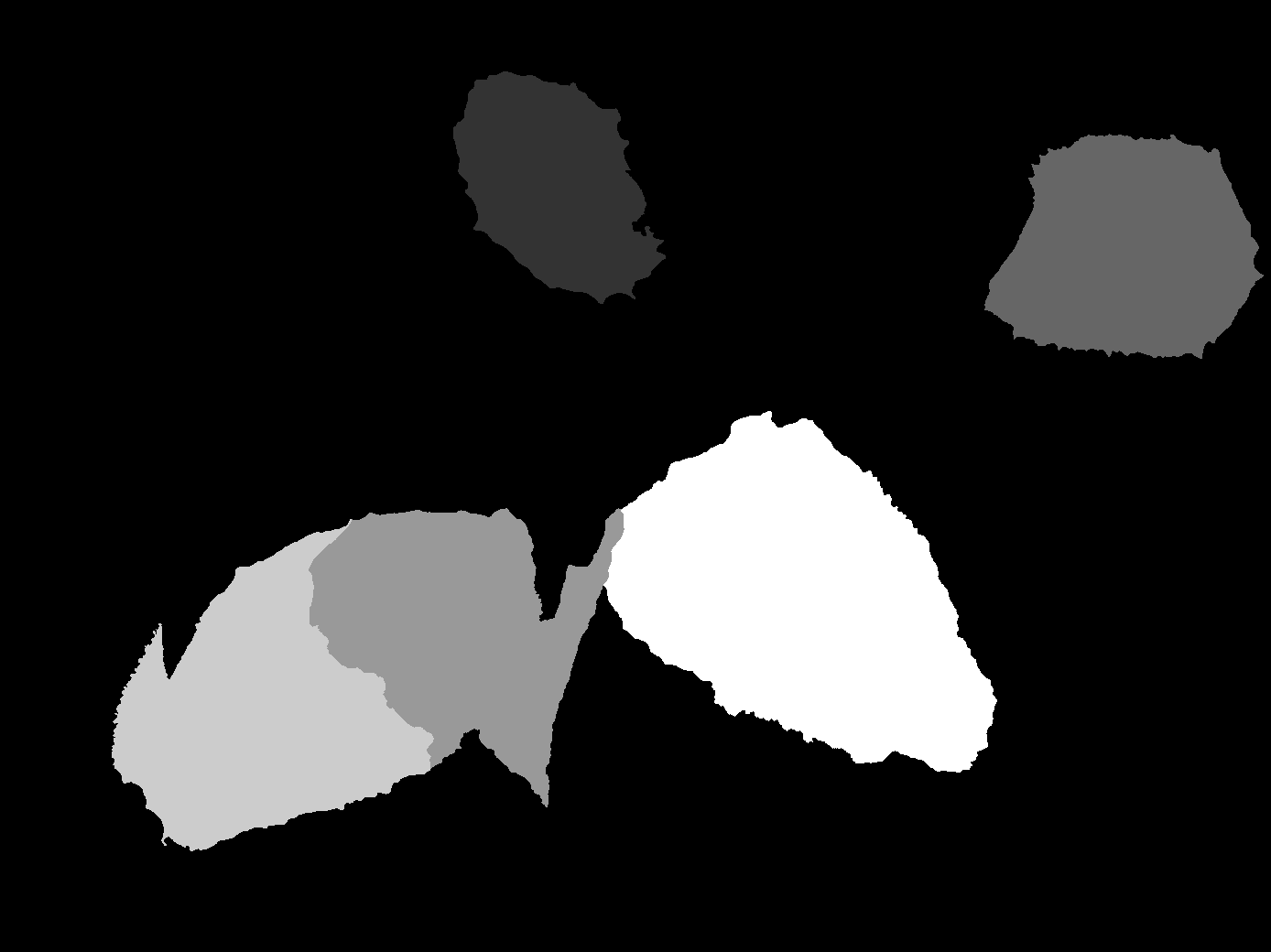

Supplement: S1 File — This file contains all scripts (CellProfiler v2.1.1 and MATLAB2016a) and data necessary to reproduce the information shown in Fig 3. (ZIP) [file pone.0180810.s001.zip › vitaminD_eColi_reproducibleResearchArchive/Results2016/B_17_c2_seg.tif]

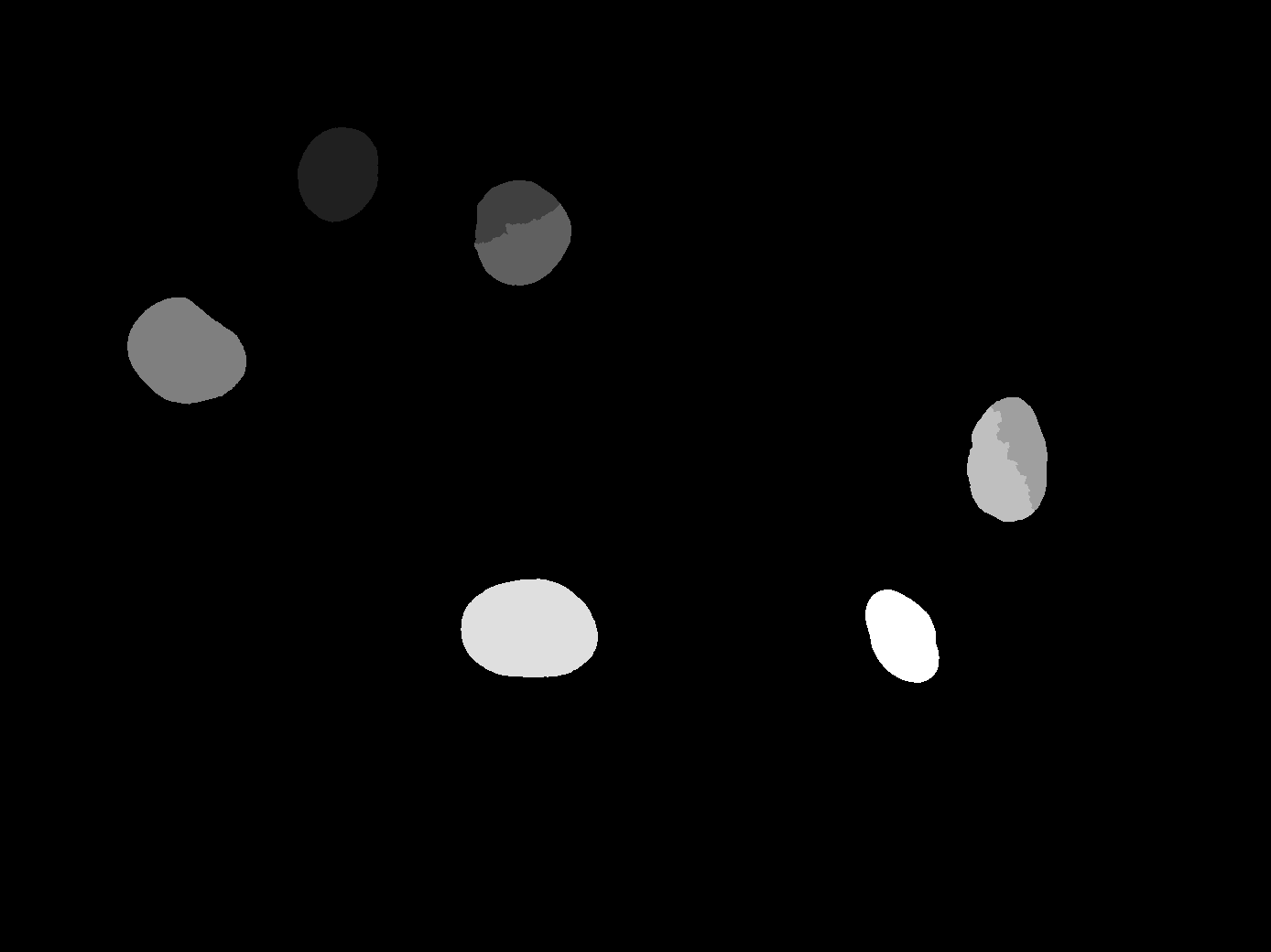

Supplement: S1 File — This file contains all scripts (CellProfiler v2.1.1 and MATLAB2016a) and data necessary to reproduce the information shown in Fig 3. (ZIP) [file pone.0180810.s001.zip › vitaminD_eColi_reproducibleResearchArchive/Results2016/B_18_c0_seg.tif]

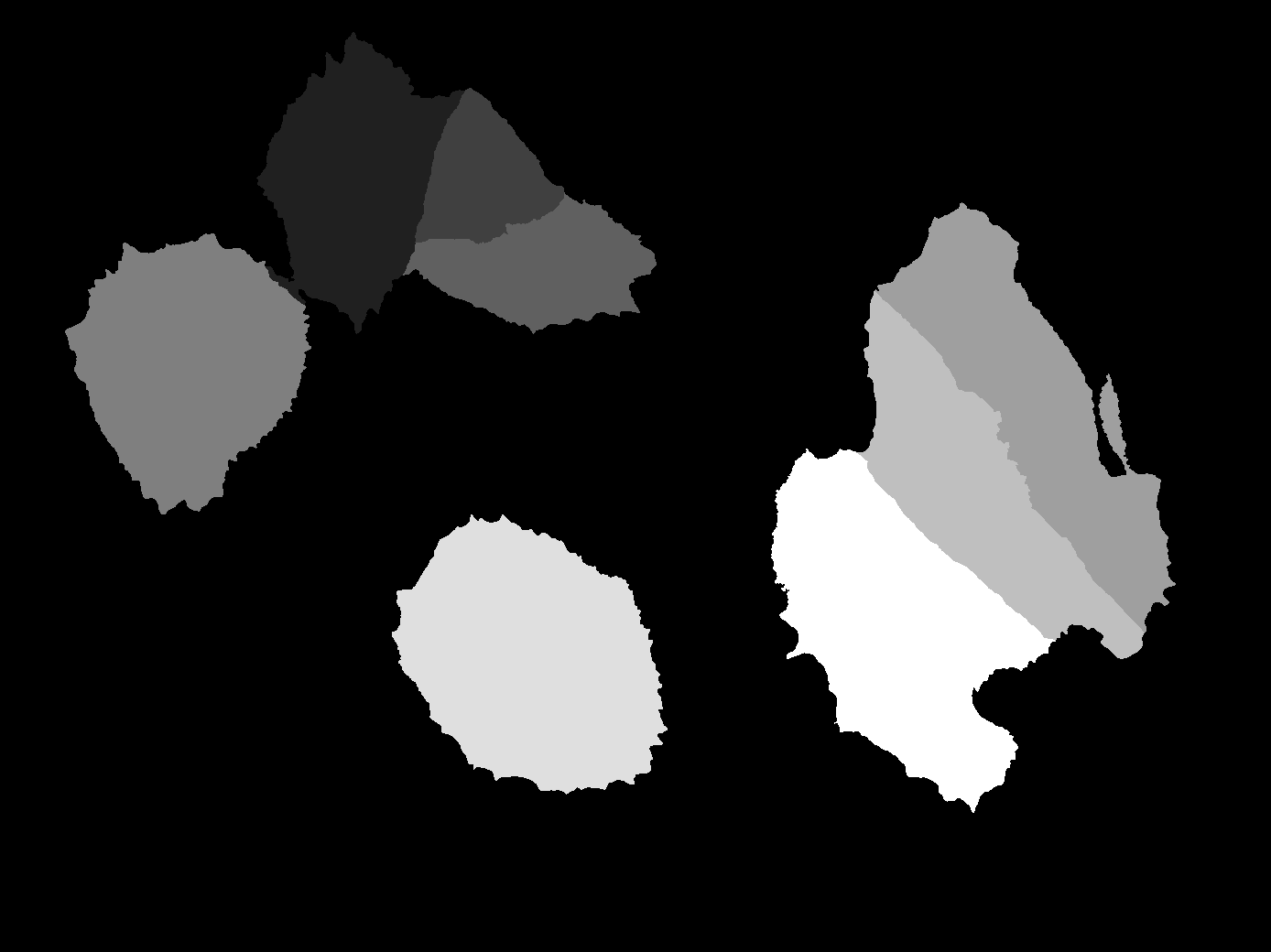

Supplement: S1 File — This file contains all scripts (CellProfiler v2.1.1 and MATLAB2016a) and data necessary to reproduce the information shown in Fig 3. (ZIP) [file pone.0180810.s001.zip › vitaminD_eColi_reproducibleResearchArchive/Results2016/B_18_c2_seg.tif]
